# Supplementary material for: Mechanochemical ligand-controlled regiodivergent hydroarylation of alkenes via iron-catalyzed C−H activation
Source: Nat Commun. 2025 Dec 26;17:150. doi: 10.1038/s41467-025-66806-5 (PMC12774961; doi:10.1038/s41467-025-66806-5)
Supplement: Supplementary file 1 — Supplementary information [file 41467_2025_66806_MOESM1_ESM.pdf]

Supplementary Information

Mechanochemical ligand-controlled regiodivergent hydroarylation of alkenes via iron-catalyzed C−H activation

Zi-Jing Zhang, Ziyue Liu, Xinran Chen and Lutz Ackermann*

**Email: Lutz.Ackermann@chemie.unigoettingen.de*

Table of Contents

1. General information..2

2. Details for condition optimization..3

3. Synthesis of bis(NHC) preligands...7

4. General procedure for iron-catalyzed regioselective hydroarylation........................12

5. Characterization data of products..15

6. Scale-up reaction...54

7. Late-stage transformations...55

8. Mechanistic investigations..64

9. Computational studies……...76

10. NMR spectra...83

11. References...182

1. General information

General data:

NMR spectra were recorded on Bruker-300 MHz spectrometer. Chemical shifts (δ) are given in ppm relative to TMS. The residual solvent signals were used as references and the chemical shifts were converted to the TMS scale ($CDCl_3$: δH = 7.26 ppm, δC = 77.16 ppm).

High resolution mass spectra were recorded on a Thermo LTQ Orbitrap XL (ESI+) or a P-SIMS-Gly of Bruker Daltonics Inc (EI+).

Melting point ranges are measured with a Stuart Melting Point Apparatus SMP3 (Barloworld Scientific, Ltd.).

Materials:

All starting materials, reagents and solvents were purchased from commercial suppliers (Aldrich, Alfa, TCI, Daicel, etc.) and used as supplied unless otherwise stated. Indole substrates[1-3] and *N*-heterocyclic carbene (NHC) preligands (**L1**–**L5**[4], **L6**[5], **L7**[6], **L8**–**L14**[7], **L15**[8]) were synthesized in accordance with similar procedures in the literature. Tetrahydrofuran was dried over Na and distilled prior to use.

2. Details for condition optimization

Table S1. Screening of NHC preligands in the reaction of indole **1a** with 1-octene **2a**

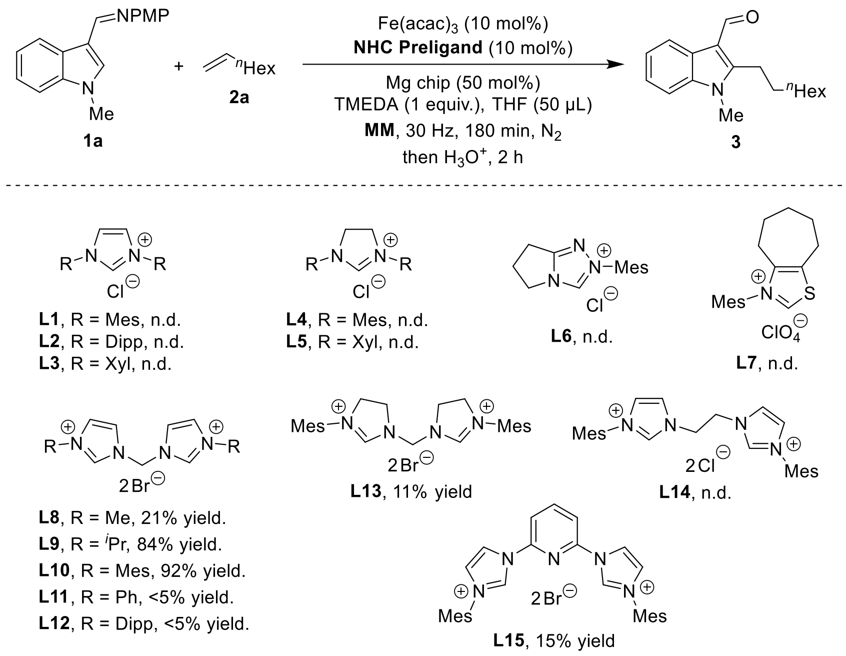

Reaction conditions: **1a** (0.1 mmol), **2a** (0.15 mmol), $Fe(acac)_3$ (10 mol%), **NHC Preligand** (10 mol%), Mg chip (50 mol%), TMEDA (0.1 mmol) and THF (50 µL) were placed in a stainless-steel vessel (5 mL) with stainless-steel ball (d_{MB} = 7 mm), milled in a mixer mill (RETSCH MM 400) at 30 Hz for 180 min under nitrogen atmosphere. Then THF (3 mL) and HCl aq. (3 M, 1 mL) were added and the mixture was stirred for 2 h. The ratio of l:b is >99:1 for all cases. The yield was determined by ^{1}H NMR spectroscopy using 1,3,5-trimethoxybenzene as the internal standard. TMEDA, *N,N,N',N'*-tetramethylethylenediamine; THF, tetrahydrofuran; Mes, 2,4,6-trimethylphenyl; Dipp, 2,6-diisopropylphenyl; Xyl, 2,6-dimethylphenyl; n.d., not detected.

Table S2. Optimization of reaction conditions

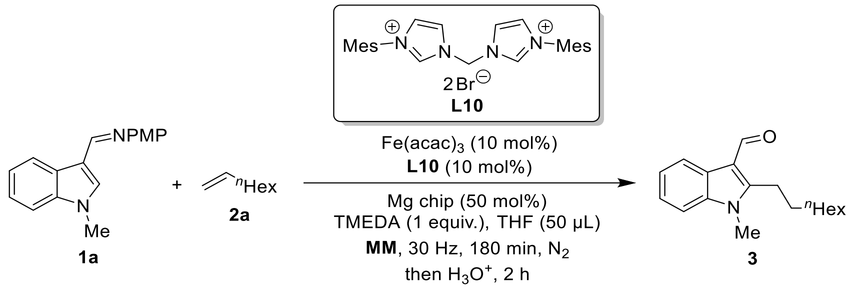

Entry	Deviation from Standard Conditions	Yield (%)
1	None	92 (90)
2	90 min	31
3	$FeCl_2$/$Co(acac)_3$/$Ni(acac)_2$/$Cu(OAc)_2$ instead of $Fe(acac)_3$	56/n.d./n.d./n.d.
4	Without $Fe(acac)_3$	n.d.
5	Without **L10**	n.d.
6	Zn powder/Mn powder instead of Mg chip	n.d./n.d.
7	Mg chip (1 equiv.)	82
8	Without Mg chip	n.d.
9	Without TMEDA	72
10	Toluene/1,4-dioxane/2-MeTHF instead of THF	81/87/91
11	Without solvent	83
12	Under air	n.d.

Reaction conditions: **1a** (0.1 mmol), **2a** (0.15 mmol), $Fe(acac)_3$ (10 mol%), **L10** (10 mol%), Mg chip (50 mol%), TMEDA (0.1 mmol) and THF (50 μL) were placed in a stainless-steel vessel (5 mL) with stainless-steel ball (d_{MB} = 7 mm), milled in a mixer mill (RETSCH MM 400) at 30 Hz for 180 min under nitrogen atmosphere. Then THF (3 mL) and HCl aq. (3 M, 1 mL) were added and the mixture was stirred for 2 h. The ratio of l:b is >99:1 for all cases. The yield was determined by ^{1}H NMR spectroscopy using 1,3,5-trimethoxybenzene as the internal standard (the yield of the isolated product is given within parentheses).

Table S3. Screening of Mechanical Parameters

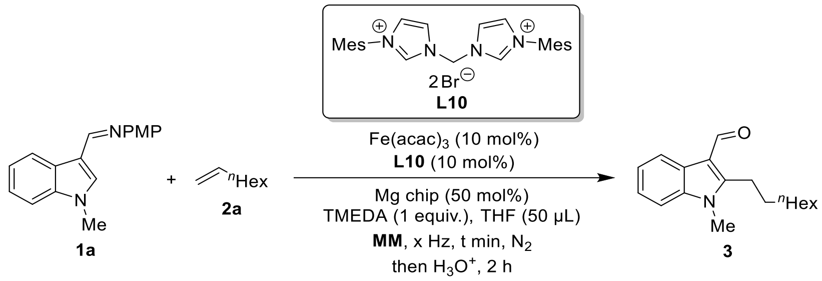

Entry	Milling Time (min)	Milling Frequency (Hz)	Milling Ball (No.)	Yield (%)
1	180	30	7 mm (1)	92 (90)
2	90	30	7 mm (1)	31
3	270	30	7 mm (1)	91
4	180	25	7 mm (1)	74
5	180	20	7 mm (1)	65
6	180	30	10 mm (1)	89
7	180	30	3 mm (20)	85
8	180	30	3 mm (10)	64

Reaction conditions: **1a** (0.1 mmol), **2a** (0.15 mmol), $Fe(acac)_3$ (10 mol%), **L10** (10 mol%), Mg chip (50 mol%), TMEDA (0.1 mmol) and THF (50 μL) were placed in a stainless-steel vessel (5 mL) with stainless-steel ball(s), milled in a mixer mill (RETSCH MM 400) at x Hz for t min under nitrogen atmosphere. Then THF (3 mL) and HCl aq. (3 M, 1 mL) were added and the mixture was stirred for 2 h. The ratio of l:b is >99:1 for all cases. The yield was determined by ^{1}H NMR spectroscopy using 1,3,5-trimethoxybenzene as the internal standard (the yield of the isolated product is given within parentheses).

Table S4. Screening of NHC preligands in the reaction of indole **1a** with styrene **48a**

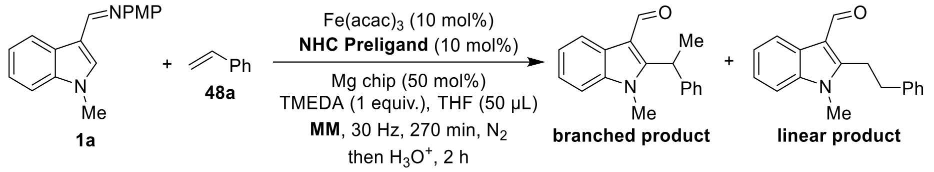

Entry	NHC Preligand	Yield (%)	Branched: Linear (*Markov: anti-Markov*)
1	**L1**	91 (87)	>99:1
2	**L2**	23	>99:1
3	**L3**	85	>99:1
4	**L4**	83	>99:1
5	**L5**	86	>99:1
6	**L6**	80	>99:1
7	**L7**	n.d.	-
8	**L8**	30	53:47
9	**L9**	88	57:43
10	**L10**	73 (72)	6:94
11	**L11**	21	28:72
12	**L12**	14	46:54
13	**L13**	27	93:7
14	**L14**	15	65:35
15	**L15**	10	67:33

Reaction conditions: **1a** (0.1 mmol), **48a** (0.15 mmol), $Fe(acac)_3$ (10 mol%), **NHC Preligand** (10 mol%), Mg chip (50 mol%), TMEDA (0.1 mmol) and THF (50 μL) were placed in a stainless-steel vessel (5 mL) with stainless-steel ball (d_{MB} = 7 mm), milled in a mixer mill (RETSCH MM 400) at 30 Hz for 270 min under nitrogen atmosphere. Then THF (3 mL) and HCl aq. (3 M, 1 mL) were added and the mixture was stirred for 2 h. The yield was determined by ^1H NMR spectroscopy using 1,3,5-trimethoxybenzene as the internal standard (the yield of the isolated product is given within parentheses).

3. Synthesis of bis(NHC) preligands

General Procedure 1: synthesis of bis(NHC) preligands

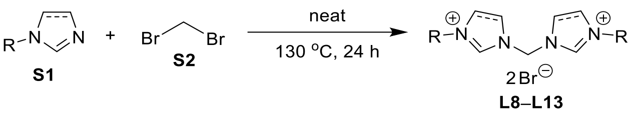

In a 25 mL round-bottomed flask under nitrogen, *N*-substituted imidazole **S1** (10 mmol) was added and the temperature was raised to 130 °C. Dibromomethane **S2** (5 mmol) was then added and the mixture was stirred at 130 °C for 24 hours. The precipitate was filtered and washed with tetrahydrofuran (10 mL). The residue was purified by recrystallization (dichloromethane /diethyl ether) and dried *in vacuo* to afford the desired preligand.

General Procedure 2: synthesis of bis(NHC) preligand L14

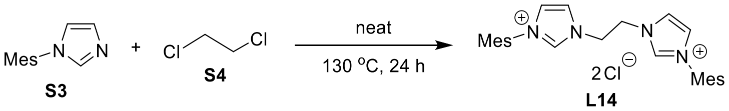

In a 25 mL round-bottomed flask under nitrogen, 1-mesityl-1*H*-imidazole **S3** (10 mmol) was added and the temperature was raised to 130 °C. 1,2-Dichloroethane **S4** (5 mmol) was then added and the mixture was stirred at 130 °C for 24 hours. The precipitate was filtered and washed with tetrahydrofuran (10 mL). The residue was purified by recrystallization (dichloromethane /diethyl ether) and dried *in vacuo* to afford **L14**.

General Procedure 3: synthesis of bis(NHC) preligand L15

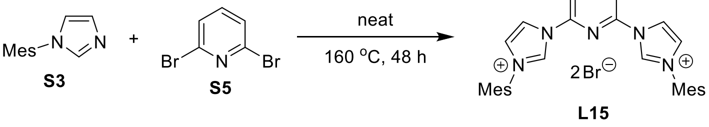

In a 25 mL round-bottomed flask under nitrogen, 1-mesityl-1*H*-imidazole **S3** (10 mmol) was added and the temperature was raised to 160 °C. 2,6-Dibromopyridine **S2** (5 mmol) was then added and the mixture was stirred at 160 °C for 48 hours. The

precipitate was filtered and washed with tetrahydrofuran (10 mL). The residue was purified by recrystallization (dichloromethane /diethyl ether) and dried *in vacuo* to afford **L15**.

1,1'-Methylenebis(3-methyl-1*H*-imidazol-3-ium) dibromide (L8)

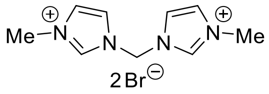

The **general procedure 1** was followed using 1-methyl-1*H*-imidazole (10 mmol, 0.82 g) and dibromomethane (5 mmol, 0.35 mL) to afford **L8** (1.36 g, 81% yield) as a white solid. **^{1}H NMR (300 MHz, DMSO-*d*$_6$)** δ 9.59 (t, *J* = 1.7 Hz, 2H), 8.11 (t, *J* = 1.8 Hz, 2H), 7.82 (t, *J* = 1.8 Hz, 2H), 6.79 (s, 2H), 3.89 (s, 6H). **^{13}C NMR (75 MHz, DMSO-*d*$_6$)** δ 138.0 (CH), 124.3 (CH), 121.9 (CH), 57.8 (CH_2), 36.3 (CH_3). **HRMS** (ESI) m/z (M–2Br–H)$^+$: calculated for $(C_9H_{13}N_4)^+$: 177.1135, found: 177.1134.

1,1'-Methylenebis(3-isopropyl-1*H*-imidazol-3-ium) dibromide (L9)

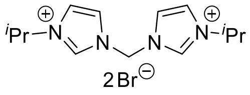

The **general procedure 1** was followed using 1-isopropyl-1*H*-imidazole (10 mmol, 1.10 g) and dibromomethane (5 mmol, 0.35 mL) to afford **L9** (1.47 g, 75% yield) as a white solid. **^{1}H NMR (300 MHz, DMSO-*d*$_6$)** δ 9.83 (t, *J* = 1.6 Hz, 2H), 8.21 (t, *J* = 1.8 Hz, 2H), 8.06 (t, *J* = 1.8 Hz, 2H), 6.74 (s, 2H), 4.69 (hept, *J* = 6.6 Hz, 2H), 1.49 (d, *J* = 6.6 Hz,12H). **^{13}C NMR (75 MHz, DMSO-*d*$_6$)** δ 136.6 (CH), 122.4 (CH), 121.3 (CH), 57.8 (CH_2), 52.8 (CH), 22.1 (CH_3). **HRMS** (ESI) m/z (M–2Br–H)$^+$: calculated for $(C_{13}H_{21}N_4)^+$: 233.1761, found: 233.1762.

1,1'-Methylenebis(3-mesityl-1*H*-imidazol-3-ium) dibromide (L10)

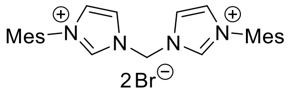

The **general procedure 1** was followed using 1-mesityl-1*H*-imidazole (10 mmol, 1.86 g) and dibromomethane (5 mmol, 0.35 mL) to afford **L10** (1.68 g, 62% yield) as a

white solid. **^{1}H NMR (300 MHz, CDCl$_3$)** δ 11.19 (t, *J* = 1.6 Hz, 2H), 9.67 (t, *J* = 1.7 Hz, 2H), 7.87 (s, 2H), 7.28 (t, *J* = 1.8 Hz, 2H), 6.96 (s, 4H), 2.29 (s, 6H), 2.01 (s, 12H). **^{13}C NMR (75 MHz, CDCl$_3$)** δ 141.8 (CH), 138.9 (C$_q$), 133.8 (C$_q$), 130.3 (C$_q$), 130.1 (CH), 124.7 (CH), 124.0 (CH), 57.0 (CH$_2$), 21.1 (CH$_3$), 17.7 (CH$_3$). **HRMS** (ESI) m/z (M–2Br–H)$^+$: calculated for $(C_{25}H_{29}N_4)^+$: 385.2387, found: 385.2388.

1,1'-Methylenebis(3-phenyl-1*H*-imidazol-3-ium) dibromide (L11)

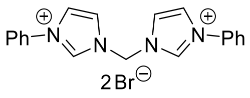

The **general procedure 1** was followed using 1-phenyl-1*H*-imidazole (10 mmol, 1.44 g) and dibromomethane (5 mmol, 0.35 mL) to afford **L11** (1.34 g, 58% yield) as a white solid. **^{1}H NMR (300 MHz, DMSO-*d*$_6$)** δ 10.47 (t, *J* = 1.7 Hz, 2H), 8.47 (dt, *J* = 8.9, 1.9 Hz, 4H), 7.89 – 7.76 (m, 4H), 7.72 – 7.65 (m, 4H), 7.64 – 7.56 (m, 2H), 6.96 (s, 2H). **^{13}C NMR (75 MHz, DMSO-*d*$_6$)** δ 137.4 (CH), 134.5 (C$_q$), 130.3 (CH), 130.2 (CH), 123.1 (CH), 122.0 (CH), 121.5 (CH), 58.3 (CH$_2$). **HRMS** (ESI) m/z (M–2Br–H)$^+$: calculated for $(C_{19}H_{17}N_4)^+$: 301.1448, found: 301.1448.

1,1'-Methylenebis(3-(2,6-diisopropylphenyl)-1*H*-imidazol-3-ium) dibromide (L12)

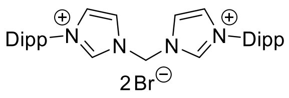

The **general procedure 1** was followed using 1-(2,6-diisopropylphenyl)-1*H*-imidazole (10 mmol, 2.28 g) and dibromomethane (5 mmol, 0.35 mL) to afford **L12** (1.52 g, 48% yield) as a pale-yellow solid. **^{1}H NMR (300 MHz, DMSO-*d*$_6$)** δ 10.40 (d, *J* = 1.6 Hz, 2H), 8.70 (t, *J* = 1.7 Hz, 2H), 8.29 (t, *J* = 1.8 Hz, 2H), 7.65 (t, *J* = 7.8 Hz, 2H), 7.47 (d, *J* = 7.8 Hz, 4H), 7.11 (s, 2H), 2.28 (hept, *J* = 6.7 Hz, 4H), 1.13 (d, *J* = 3.0 Hz, 12H), 1.11 (d, *J* = 3.0 Hz, 12H). **^{13}C NMR (75 MHz, DMSO-*d*$_6$)** δ 144.9 (C$_q$), 139.1 (CH), 131.8 (CH), 130.2 (C$_q$), 125.7 (CH), 124.6 (CH), 123.3 (CH), 58.7 (CH$_2$), 28.0 (CH), 23.8 (CH$_3$), 23.8 (CH$_3$). **HRMS** (ESI) m/z (M–2Br–H)$^+$: calculated for $(C_{31}H_{41}N_4)^+$: 469.3326, found: 469.3327.

1,1'-Methylenebis(3-mesityl-4,5-dihydro-1*H*-imidazol-3-ium) dibromide (L13)

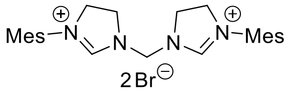

The **general procedure 1** was followed using 1-mesityl-4,5-dihydro-1*H*-imidazole (10 mmol, 1.88 g) and dibromomethane (5 mmol, 0.35 mL) to afford **L13** (1.46 g, 53% yield) as a white solid. **^{1}H NMR (300 MHz, DMSO-*d*$_6$)** δ 9.31 (s, 2H), 7.04 (s, 4H), 5.53 (s, 2H), 4.31 (s, 8H), 2.32 (s, 12H), 2.26 (s, 6H). **^{13}C NMR (75 MHz, DMSO-*d*$_6$)** δ 160.4 (CH), 139.6 (C_q), 135.3 (C_q), 130.8 (C_q), 129.4 (CH), 58.9 (CH_2), 51.4 (CH_2), 47.5 (CH_2), 20.6 (CH_3), 17.5 (CH_3). **HRMS** (ESI) m/z $(M–2Br–H)^+$: calculated for $(C_{25}H_{33}N_4)^+$: 389.2700, found: 389.2705.

1,1'-(Ethane-1,2-diyl)bis(3-mesityl-1*H*-imidazol-3-ium) dichloride (L14)

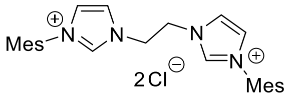

The **general procedure 2** was followed using 1-mesityl-1*H*-imidazole (10 mmol, 1.86 g) and 1,2-dichloroethane (5 mmol, 0.39 mL) to afford **L14** (0.88 g, 37% yield) as a white solid. **^{1}H NMR (300 MHz, CDCl$_3$)** δ 10.26 (s, 2H), 9.10 (s, 2H), 7.10 (t, J = 1.4 Hz, 2H), 6.98 (s, 4H), 5.53 (s, 4H), 2.32 (s, 6H), 2.01 (s, 12H). **^{13}C NMR (75 MHz, CDCl$_3$)** δ 141.6 (CH), 138.0 (C_q), 134.1 (C_q), 130.6 (C_q), 130.0 (CH), 125.5 (CH), 122.9 (CH), 48.7 (CH_2), 21.2 (CH_3), 17.5 (CH_3). **HRMS** (ESI) m/z $(M–2Cl–H)^+$: calculated for $(C_{26}H_{31}N_4)^+$: 399.2543, found: 399.2541.

1,1'-(Pyridine-2,6-diyl)bis(3-mesityl-1*H*-imidazol-3-ium) dibromide (L15)

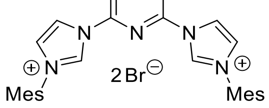

The **general procedure 3** was followed using 1-mesityl-1*H*-imidazole (10 mmol, 1.86 g) and 2,6-dibromopyridine (5 mmol, 1.17 g) to afford **L15** (2.25 g, 74% yield) as a brown solid. **^{1}H NMR (300 MHz, CDCl$_3$)** δ 11.76 (t, J = 1.6 Hz, 2H), 9.77 (t, J =

1.9 Hz, 2H), 9.04 (d, J = 8.1 Hz, 2H), 8.29 (t, J = 8.1 Hz, 1H), 7.32 (t, J = 1.8 Hz, 2H), 6.95 (s, 4H), 2.26 (s, 6H), 2.12 (s, 12H). **^{13}C NMR (75 MHz, $CDCl_3$)** δ 145.4 (C_q), 145.1 (CH), 141.4 (CH), 137.5 (C_q), 134.0 (C_q), 130.5 (C_q), 129.9 (CH), 124.7 (CH), 122.2 (CH), 116.9 (CH), 21.1 (CH_3), 18.0 (CH_3). **HRMS** (ESI) m/z $(M–2Br–H)^+$: calculated for $(C_{29}H_{30}N_5)^+$: 448.2496, found: 448.2498.

4. General procedure for iron-catalyzed regioselective hydroarylation

General Procedure 4: iron-catalyzed regioselective hydroarylation of unactivated alkenes

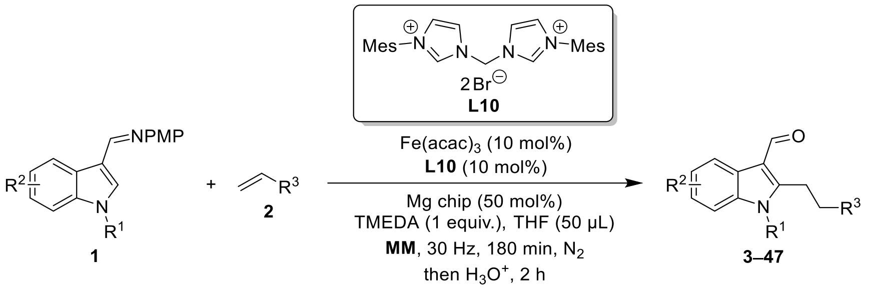

In the glove box, a mixture of indole substrate **1** (0.2 mmol), alkyl alkene **2** (0.3 mmol), $Fe(acac)_3$ (10 mol%, 0.02 mmol, 7.1 mg), **L10** (10 mol%, 0.02 mmol, 10.9 mg), magnesium chip (50 mol%, 0.1 mmol, 2.4 mg), TMEDA (0.2 mmol, 30 μL) and tetrahydrofuran (50 μL) were placed in a nitrogen-purged stainless-steel vessel (5 mL) with a stainless-steel ball (d_{MB} = 7 mm). Then, the vessel was sealed and milled in a mixer mill (RETSCH MM 400) at 30 Hz for 180 min under nitrogen atmosphere. Then, the reaction mixture was diluted with tetrahydrofuran (3 mL) and quenched with HCl aqueous solution (3 M, 1 mL). The resulting mixture was stirred at room temperature for 2 hours. The phases were then separated, the aqueous layer was extracted with ethyl acetate (5 mL ×3). The combined organic layer was washed with saturated $NaHCO_3$ solution and brine, dried over Na_2SO_4, filtered and concentrated *in vacuo*. The linear and branched ratio was determined by ^{1}H NMR analysis of the crude reaction mixture. The residue was purified by column chromatography on silica gel (*n*-hexane: ethyl acetate = 10:1) to afford the desired product.

General Procedure 5: iron-catalyzed regioselective hydroarylation of aryl alkenes

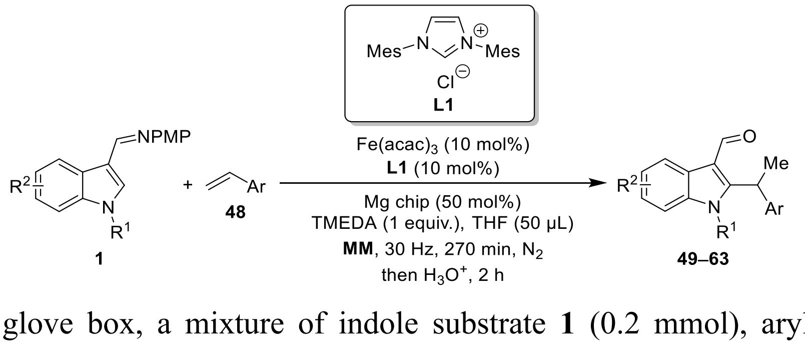

In the glove box, a mixture of indole substrate **1** (0.2 mmol), aryl alkene **48**

(0.3 mmol), $Fe(acac)_3$ (10 mol%, 0.02 mmol, 7.1 mg), **L1** (10 mol%, 0.02 mmol, 6.8 mg), magnesium chip (50 mol%, 0.1 mmol, 2.4 mg), TMEDA (0.2 mmol, 30 μL) and tetrahydrofuran (50 μL) were placed in a nitrogen-purged stainless-steel vessel (5 mL) with a stainless-steel ball (d_{MB} = 7 mm). Then, the vessel was sealed and milled in a mixer mill (RETSCH MM 400) at 30 Hz for 270 min under nitrogen atmosphere. Then, the reaction mixture was diluted with tetrahydrofuran (3 mL) and quenched with HCl aqueous solution (3 M, 1 mL). The resulting mixture was stirred at room temperature for 2 hours. The phases were then separated, the aqueous layer was extracted with ethyl acetate (5 mL ×3). The combined organic layer was washed with saturated $NaHCO_3$ solution and brine, dried over Na_2SO_4, filtered and concentrated *in vacuo*. The linear and branched ratio was determined by ^{1}H NMR analysis of the crude reaction mixture. The residue was purified by column chromatography on silica gel (*n*-hexane: ethyl acetate = 5:1) to afford the desired product.

General Procedure 6: iron-catalyzed regioselective hydroarylation of aryl alkenes

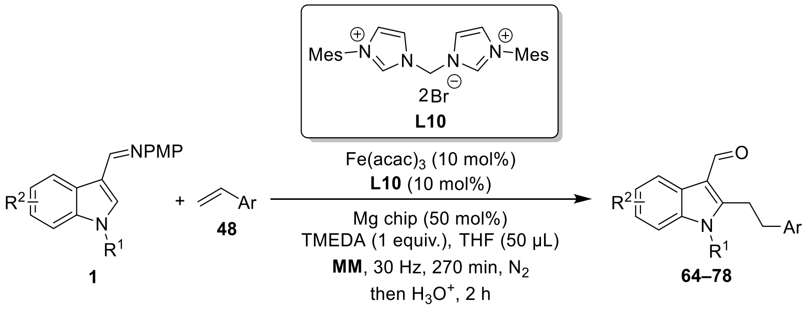

In the glove box, a mixture of indole substrate **1** (0.2 mmol), aryl alkene **48** (0.3 mmol), $Fe(acac)_3$ (10 mol%, 0.02 mmol, 7.1 mg), **L10** (10 mol%, 0.02 mmol, 10.9 mg), magnesium chip (50 mol%, 0.1 mmol, 2.4 mg), TMEDA (0.2 mmol, 30 μL) and tetrahydrofuran (50 μL) were placed in a nitrogen-purged stainless-steel vessel (5 mL) with a stainless-steel ball (d_{MB} = 7 mm). Then, the vessel was sealed and milled in a mixer mill (RETSCH MM 400) at 30 Hz for 270 min under nitrogen atmosphere. Then, the reaction mixture was diluted with tetrahydrofuran (3 mL) and quenched with HCl aqueous solution (3 M, 1 mL). The resulting mixture was stirred at room temperature for 2 hours. The phases were then separated, the aqueous layer was

extracted with ethyl acetate (5 mL ×3). The combined organic layer was washed with saturated $NaHCO_3$ solution and brine, dried over Na_2SO_4, filtered and concentrated *in vacuo*. The linear and branched ratio was determined by ^1H NMR analysis of the crude reaction mixture. The residue was purified by column chromatography on silica gel (*n*-hexane: ethyl acetate = 5:1) to afford the desired product.

5. Characterization data of products

1-Methyl-2-octyl-1*H*-indole-3-carbaldehyde (3)

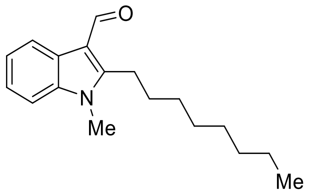

The **general procedure 4** was followed using indole substrate **1a** (0.2 mmol, 52.8 mg) and 1-octene (**2a**) (0.3 mmol, 47 μL) to afford **3** (48.9 mg, 90% yield, l:b >99:1) as a colorless oil. **^{1}H NMR (300 MHz, CDCl$_3$)** δ 10.14 (s, 1H), 8.39 – 8.17 (m, 1H), 7.34 – 7.20 (m, 3H), 3.69 (s, 3H), 3.09 – 3.00 (m, 1H), 1.73 – 1.60 (m, 2H), 1.48 – 1.36 (m, 2H), 1.38 – 1.22 (m, 8H), 0.94 – 0.84 (m, 1H). **^{13}C NMR (75 MHz, CDCl$_3$)** δ 184.2 (CH), 152.2 (C$_q$), 137.2 (C$_q$), 125.8 (C$_q$), 123.2 (CH), 122.9 (CH), 121.1 (CH), 114.0 (C$_q$), 109.4 (CH), 31.9 (CH$_2$), 30.2 (CH$_2$), 29.8 (CH$_3$), 29.5 (CH$_2$), 29.4 (CH$_2$), 29.2 (CH$_2$), 24.5 (CH$_2$), 22.7 (CH$_2$), 14.2 (CH$_3$). **HRMS** (ESI) m/z (M+H)$^+$: calculated for ($C_{18}H_{26}NO$)$^+$: 272.2009, found: 272.2003.

1-Ethyl-2-octyl-1*H*-indole-3-carbaldehyde (4)

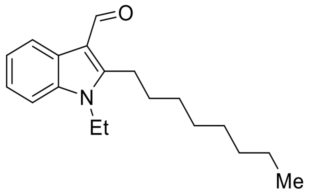

The **general procedure 4** was followed using indole substrate **1b** (0.2 mmol, 55.6 mg) and 1-octene (**2a**) (0.3 mmol, 47 μL) to afford **4** (42.5 mg, 75% yield, l:b >99:1) as a colorless oil. **^{1}H NMR (300 MHz, CDCl$_3$)** δ 10.18 (s, 1H), 8.38 – 8.23 (m, 1H), 7.41 – 7.22 (m, 3H), 4.20 (q, J = 7.3 Hz, 2H), 3.13 – 3.01 (m, 2H), 1.79 – 1.62 (m, 2H), 1.53 – 1.37 (m, 5H), 1.39 – 1.19 (m, 8H), 0.94 – 0.82 (m, 3H). **^{13}C NMR (75 MHz, CDCl$_3$)** δ 184.3 (CH), 151.5 (C$_q$), 136.0 (C$_q$), 126.2 (C$_q$), 123.2 (CH), 122.9 (CH), 121.3 (CH), 114.1 (C$_q$), 109.7 (CH), 38.3 (CH$_2$), 31.9 (CH$_2$), 31.0 (CH$_2$), 29.7 (CH$_2$), 29.4 (CH$_2$), 29.3 (CH$_2$), 24.7 (CH$_2$), 22.8 (CH$_2$), 15.3 (CH$_3$), 14.2 (CH$_3$). **HRMS** (ESI) m/z (M+H)$^+$: calculated for ($C_{19}H_{28}NO$)$^+$: 286.2165, found: 286.2160.

1-(Methoxymethyl)-2-octyl-1*H*-indole-3-carbaldehyde (5)

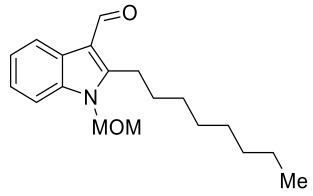

The **general procedure 4** was followed using indole substrate **1c** (0.2 mmol, 58.8 mg) and 1-octene (**2a**) (0.3 mmol, 47 μL) to afford **5** (36.8 mg, 61% yield, l:b >99:1) as a pale-yellow oil. **^{1}H NMR (300 MHz, CDCl$_3$)** δ 10.23 (s, 1H), 8.35 – 8.23 (m, 1H), 7.50 – 7.41 (m, 1H), 7.33 – 7.26 (m, 2H), 5.49 (s, 2H), 3.32 (s, 3H), 3.20 – 3.08 (m, 2H), 1.80 – 1.63 (m, 2H), 1.51 – 1.38 (m, 2H), 1.38 – 1.20 (m, 8H), 0.94 – 0.81 (m, 3H). **^{13}C NMR (75 MHz, CDCl$_3$)** δ 184.9 (CH), 152.5 (C_q), 137.1 (C_q), 125.8 (C_q), 123.7 (CH), 123.3 (CH), 121.4 (CH), 115.2 (C_q), 109.9 (CH), 74.1 (CH_2), 56.4 (CH_3), 31.9 (CH_2), 31.2 (CH_2), 29.7 (CH_2), 29.4 (CH_2), 29.3 (CH_2), 24.6 (CH_2), 22.8 (CH_2), 14.2 (CH_3). **HRMS** (ESI) m/z $(M+H)^+$: calculated for $(C_{19}H_{28}NO_2)^+$: 302.2115, found: 302.2108.

1-Benzyl-2-octyl-1*H*-indole-3-carbaldehyde (6)

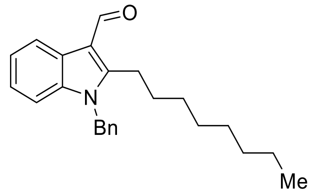

The **general procedure 4** was followed using indole substrate **1d** (0.2 mmol, 68.0 mg) and 1-octene (**2a**) (0.3 mmol, 47 μL) at 30 Hz for 270 min to afford **6** (32.8 mg, 47% yield, l:b >99:1) as a pale-yellow oil. **^{1}H NMR (300 MHz, CDCl$_3$)** δ 10.23 (s, 1H), 8.33 (d, J = 7.8 Hz, 1H), 7.35 – 7.23 (m, 4H), 7.25 – 7.18 (m, 2H), 7.04 – 6.96 (m, 2H), 5.38 (s, 2H), 3.11 – 3.01 (m, 2H), 1.65 – 1.51 (m, 2H), 1.44 – 1.31 (m, 2H), 1.31 – 1.17 (m, 8H), 0.91 – 0.82 (m, 3H). **^{13}C NMR (75 MHz, CDCl$_3$)** δ 184.6 (CH), 152.2 (C_q), 136.9 (C_q), 136.2 (C_q), 129.2 (CH), 128.0 (CH), 126.0 (CH), 126.0 (C_q), 123.5 (CH), 123.1 (CH), 121.3 (CH), 114.6 (C_q), 110.2 (CH), 46.9 (CH_2), 31.9 (CH_2), 30.7 (CH_2), 29.6 (CH_2), 29.3 (CH_2), 29.2 (CH_2), 24.8 (CH_2), 22.7 (CH_2), 14.2 (CH_3). **HRMS** (ESI) m/z $(M+H)^+$: calculated for $(C_{24}H_{30}NO)^+$: 348.2322, found: 348.2323.

1-(4-Methoxybenzyl)-2-octyl-1*H*-indole-3-carbaldehyde (7)

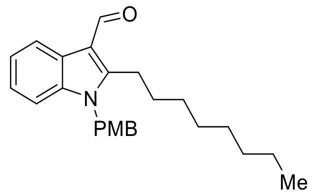

The **general procedure 4** was followed using indole substrate **1e** (0.2 mmol, 74.0 mg) and 1-octene (**2a**) (0.3 mmol, 47 µL) at 30 Hz for 270 min to afford **7** (40.5 mg, 54% yield, l:b >99:1) as a colorless oil. **^{1}H NMR (300 MHz, $CDCl_3$)** δ 10.21 (s, 1H), 8.32 (d, *J* = 7.6 Hz, 1H), 7.34 – 7.24 (m, 1H), 7.27 – 7.18 (m, 2H), 6.93 (d, *J* = 8.8 Hz, 2H), 6.82 (d, *J* = 8.8 Hz, 2H), 5.32 (s, 2H), 3.76 (s, 3H), 3.15 – 2.98 (m, 2H), 1.70 – 1.50 (m, 2H), 1.45 – 1.29 (m, 2H), 1.33 – 1.15 (m, 8H), 0.92 – 0.81 (m, 3H). **^{13}C NMR (75 MHz, $CDCl_3$)** δ 184.5 (CH), 159.4 (C_q), 152.2 (C_q), 136.9 (C_q), 128.1 (C_q), 127.3 (CH), 126.0 (C_q), 123.5 (CH), 123.1 (CH), 121.3 (CH), 114.5 (CH), 114.5 (C_q), 110.2 (CH), 55.4 (CH_3), 46.4 (CH_2), 31.9 (CH_2), 30.7 (CH_2), 29.6 (CH_2), 29.4 (CH_2), 29.3 (CH_2), 24.9 (CH_2), 22.7 (CH_2), 14.2 (CH_3). **HRMS** (ESI) m/z $(M+H)^+$: calculated for $(C_{25}H_{32}NO_2)^+$: 378.2428, found: 378.2422.

1-Methyl-2-octyl-1*H*-pyrrolo[2,3-*b*]pyridine-3-carbaldehyde (8)

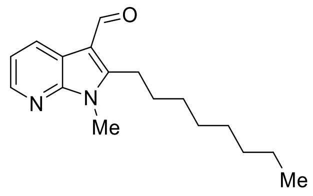

The **general procedure 4** was followed using indole substrate **1f** (0.2 mmol, 53.0 mg) and 1-octene (**2a**) (0.3 mmol, 47 µL) to afford **8** (46.6 mg, 86% yield, l:b >99:1) as a pale-yellow oil. **^{1}H NMR (300 MHz, $CDCl_3$)** δ 10.14 (s, 1H), 8.50 (dd, *J* = 7.8, 1.6 Hz, 1H), 8.34 (dd, *J* = 4.7, 1.6 Hz, 1H), 7.21 (dd, *J* = 7.8, 4.7 Hz, 1H), 3.85 (s, 3H), 3.18 – 3.03 (m, 2H), 1.76 – 1.66 (m, 2H), 1.50 – 1.38 (m, 2H), 1.38 – 1.20 (m, 8H), 0.90 – 0.83 (m, 3H). **^{13}C NMR (75 MHz, $CDCl_3$)** δ 184.1 (CH), 152.8 (C_q), 148.5 (C_q), 144.0 (CH), 129.4 (CH), 118.9 (CH), 118.4 (C_q), 112.5 (C_q), 31.9 (CH_2), 30.1 (CH_2), 29.5 (CH_2), 29.4 (CH_2), 29.3 (CH_2), 28.5 (CH_3), 24.8 (CH_2), 22.7 (CH_2), 14.2 (CH_3).

HRMS (ESI) m/z $(M+H)^+$: calculated for $(C_{17}H_{25}N_2O)^+$: 273.1961, found: 273.1961.

4-Fluoro-1-methyl-2-octyl-1*H*-indole-3-carbaldehyde (9)

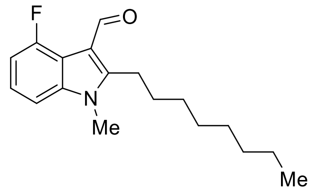

The **general procedure 4** was followed using indole substrate **1g** (0.2 mmol, 56.4 mg) and 1-octene (**2a**) (0.3 mmol, 47 μL) to afford **9** (42.4 mg, 73% yield, l:b >99:1) as a yellow oil. **^{1}H NMR (300 MHz, $CDCl_3$)** δ 10.38 (d, J = 1.5 Hz, 1H), 7.23 – 7.08 (m, 2H), 6.95 (ddd, J = 10.7, 7.5, 1.0 Hz, 1H), 3.73 (s, 3H), 3.27 – 3.13 (m, 2H), 1.73 – 1.56 (m, 2H), 1.52 – 1.36 (m, 2H), 1.39 – 1.19 (m, 8H), 0.93 – 0.81 (m, 3H). **^{13}C NMR (75 MHz, $CDCl_3$)** δ 186.3 (d, J = 5.1 Hz, CH), 156.7 (d, J = 247.7 Hz, C_q), 149.8 (C_q), 139.5 (d, J = 12.3 Hz, C_q), 123.0 (d, J = 7.8 Hz, CH), 115.7 (d, J = 21.5 Hz, C_q), 112.6 (d, J = 5.7 Hz, C_q), 108.0 (d, J = 20.1 Hz, CH), 106.0 (d, J = 3.5 Hz, CH), 32.0 (CH_2), 30.0 (CH_3), 29.7 (CH_2), 29.5 (CH_2), 29.3 (CH_2), 29.2 (CH_2), 25.9 (CH_2), 22.8 (CH_2), 14.2 (CH_3). **^{19}F NMR (282 MHz, $CDCl_3$)** δ -116.43. **HRMS** (ESI) m/z $(M+H)^+$: calculated for $(C_{18}H_{25}FNO)^+$: 290.1915, found: 290.1916.

5-Fluoro-1-methyl-2-octyl-1*H*-indole-3-carbaldehyde (10)

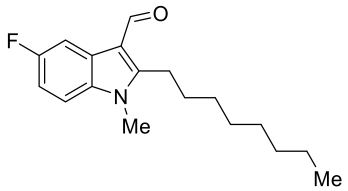

The **general procedure 4** was followed using indole substrate **1h** (0.2 mmol, 56.4 mg) and 1-octene (**2a**) (0.3 mmol, 47 μL) to afford **10** (48.0 mg, 83% yield, l:b >99:1) as a yellow oil. **^{1}H NMR (300 MHz, $CDCl_3$)** δ 10.08 (s, 1H), 7.95 (dd, J = 9.4, 2.5 Hz, 1H), 7.20 (dd, J = 8.8, 4.2 Hz, 1H), 6.99 (td, J = 9.0, 2.6 Hz, 1H), 3.70 (s, 3H), 3.08 – 2.99 (m, 2H), 1.73 – 1.58 (m, 2H), 1.50 – 1.34 (m, 2H), 1.38 – 1.21 (m, 8H), 0.91 – 0.81 (m, 3H). **^{13}C NMR (75 MHz, $CDCl_3$)** δ 184.0 (CH), 160.0 (d, J = 238.1 Hz, C_q), 153.23 (C_q), 133.67 (C_q), 126.4 (d, J = 11.3 Hz, C_q), 114.1 (d, J = 4.2 Hz, C_q), 111.2 (d,

J = 26.2 Hz, CH), 110.1 (d, *J* = 9.7 Hz, CH), 106.9 (d, *J* = 24.8 Hz, CH), 31.9 (CH_2), 30.3 (CH_2), 30.1 (CH_3), 29.5 (CH_2), 29.4 (CH_2), 29.3 (CH_2), 24.7 (CH_2), 22.7 (CH_2), 14.2 (CH_2). **^{19}F NMR (282 MHz, $CDCl_3$)** δ -120.77. **HRMS** (ESI) m/z $(M+H)^+$: calculated for $(C_{18}H_{25}FNO)^+$: 290.1915, found: 290.1911.

1-Methyl-2-octyl-5-(trifluoromethyl)-1*H*-indole-3-carbaldehyde (11)

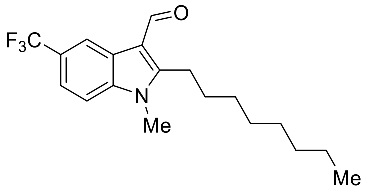

The **general procedure 4** was followed using indole substrate **1i** (0.2 mmol, 66.4 mg) and 1-octene (**2a**) (0.3 mmol, 47 μL) at 30 Hz for 270 min to afford **11** (23.9 mg, 35% yield, l:b >99:1) as a yellow oil. **^{1}H NMR (300 MHz, $CDCl_3$)** δ 10.15 (s, 1H), 8.58 (s, 1H), 7.51 (dd, *J* = 8.7, 1.8 Hz, 1H), 7.37 (d, *J* = 8.6 Hz, 1H), 3.76 (s, 3H), 3.17 – 2.97 (m, 2H), 1.75 – 1.63 (m, 2H), 1.51 – 1.35 (m, 2H), 1.37 – 1.20 (m, 8H), 0.91 – 0.84 (m, 3H). **^{13}C NMR (75 MHz, $CDCl_3$)** δ 184.1 (CH), 153.7 (C_q), 138.6 (C_q), 125.3 (q, *J* = 32.0 Hz, C_q), 125.3 (C_q), 125.1 (q, *J* = 271.8 Hz, C_q), 120.1 (q, *J* = 3.6 Hz, CH), 119.0 (q, *J* = 4.0 Hz, CH), 114.4 (C_q), 109.7 (CH), 31.9 (CH_2), 30.2 (CH_2), 30.1 (CH_3), 29.5 (CH_2), 29.4 (CH_2), 29.3 (CH_2), 24.6 (CH_2), 22.7 (CH_2), 14.2 (CH_3). **^{19}F NMR (282 MHz, $CDCl_3$)** δ -60.60. **HRMS** (ESI) m/z $(M+H)^+$: calculated for $(C_{19}H_{25}F_3NO)^+$: 340.1883, found: 340.1875.

3-Formyl-1-methyl-2-octyl-1*H*-indole-5-carbonitrile (12)

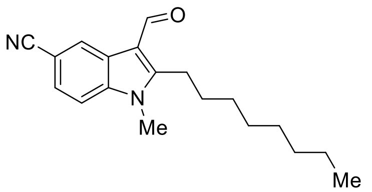

The **general procedure 4** was followed using indole substrate **1j** (0.2 mmol, 57.8 mg) and 1-octene (**2a**) (0.3 mmol, 47 μL) at 30 Hz for 270 min to afford **12** (31.0 mg, 52% yield, l:b >99:1) as a pale-yellow oil. **^{1}H NMR (300 MHz, $CDCl_3$)** δ 10.16 (s, 1H), 8.64 (d, *J* = 1.5 Hz, 1H), 7.53 (dd, *J* = 8.5, 1.6 Hz, 1H), 7.38 (d, *J* = 8.5 Hz, 1H),

3.78 (s, 3H), 3.20 – 2.98 (m, 2H), 1.77 – 1.62 (m, 2H), 1.49 – 1.38 (m, 2H), 1.38 – 1.17 (m, 8H), 0.92 – 0.84 (m, 3H). **^{13}C NMR (75 MHz, CDCl$_3$)** δ 184.1 (CH), 154.0 (C$_q$), 138.8 (C$_q$), 126.6 (CH), 126.5 (CH), 125.6 (C$_q$), 120.1 (C$_q$), 114.3 (C$_q$), 110.4 (CH), 106.2 (C$_q$), 31.9 (CH$_2$), 30.2 (CH$_3$), 30.2 (CH$_2$), 29.5 (CH$_2$), 29.4 (CH$_2$), 29.3 (CH$_2$), 24.7 (CH$_2$), 22.7 (CH$_2$), 14.2 (CH$_3$). **HRMS** (ESI) m/z (M+H)$^+$: calculated for ($C_{19}H_{25}N_2O$)$^+$: 297.1961, found: 297.1956.

Methyl 3-formyl-1-methyl-2-octyl-1*H*-indole-5-carboxylate (13)

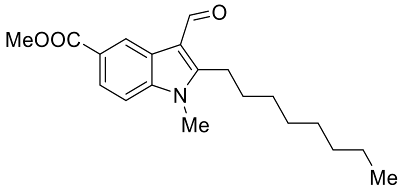

The **general procedure 4** was followed using indole substrate **1k** (0.2 mmol, 64.4 mg) and 1-octene (**2a**) (0.3 mmol, 47 μL) to afford **13** (51.7 mg, 79% yield, l:b >99:1) as a pale-yellow solid. **M.p.**: 60-62 °C. **^{1}H NMR (300 MHz, CDCl$_3$)** δ 10.18 (s, 1H), 8.95 (d, J = 1.6 Hz, 1H), 8.00 (dd, J = 8.6, 1.7 Hz, 1H), 7.33 (d, J = 8.6 Hz, 1H), 3.94 (s, 3H), 3.75 (s, 3H), 3.14 – 3.03 (m, 2H), 1.75 – 1.61 (m, 2H), 1.51 – 1.35 (m, 2H), 1.39 – 1.21 (m, 8H), 0.90 – 0.83 (m, 3H). **^{13}C NMR (75 MHz, CDCl$_3$)** δ 184.2 (CH), 167.9 (C$_q$), 153.4 (C$_q$), 139.7 (C$_q$), 125.4 (C$_q$), 124.9 (C$_q$), 124.9 (CH), 123.4 (CH), 114.6 (C$_q$), 109.3 (CH), 52.1 (CH$_3$), 31.9 (CH$_2$), 30.2 (CH$_2$), 30.1 (CH$_3$), 29.5 (CH$_2$), 29.4 (CH$_2$), 29.3 (CH$_2$), 24.7 (CH$_2$), 22.7 (CH$_2$), 14.2 (CH$_3$). **HRMS** (ESI) m/z (M+H)$^+$: calculated for ($C_{20}H_{28}NO_3$)$^+$: 330.2064, found: 330.2064.

1,5-Dimethyl-2-octyl-1*H*-indole-3-carbaldehyde (14)

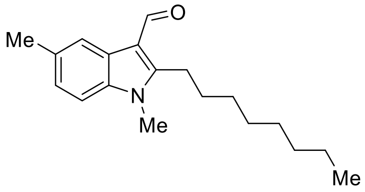

The **general procedure 4** was followed using indole substrate **1l** (0.2 mmol, 55.6 mg) and 1-octene (**2a**) (0.3 mmol, 47 μL) to afford **14** (47.9 mg, 84% yield, l:b >99:1) as a pale-yellow solid. **M.p.**: 98-99 °C. **^{1}H NMR (300 MHz, CDCl$_3$)** δ 10.11 (s, 1H), 8.10

(s, 1H), 7.18 (d, J = 8.3 Hz, 1H), 7.10 (dd, J = 8.4, 1.6 Hz, 1H), 3.68 (s, 3H), 3.11 – 2.97 (m, 2H), 2.48 (s, 3H), 1.73 – 1.58 (m, 2H), 1.50 – 1.37 (m, 2H), 1.38 – 1.22 (m, 8H), 0.94 – 0.81 (m, 3H). **^{13}C NMR (75 MHz, $CDCl_3$)** δ 184.2 (CH), 152.2 (C_q), 135.5 (C_q), 132.6 (C_q), 125.9 (C_q), 124.6 (CH), 121.1 (CH), 113.7 (C_q), 109.1 (CH), 31.9 (CH_2), 30.3 (CH_2), 29.9 (CH_3), 29.5 (CH_2), 29.4 (CH_2), 29.3 (CH_2), 24.5 (CH_2), 22.7 (CH_2), 21.5 (CH_3), 14.2 (CH_3). **HRMS** (ESI) m/z $(M+H)^+$: calculated for $(C_{19}H_{28}NO)^+$: 286.2165, found: 286.2158.

1-Methyl-2-octyl-5-vinyl-1*H*-indole-3-carbaldehyde (15)

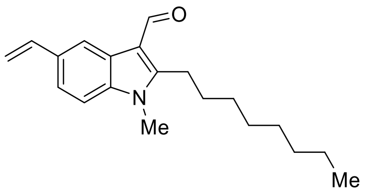

The **general procedure 4** was followed using indole substrate **1m** (0.2 mmol, 58.0 mg) and 1-octene (**2a**) (0.3 mmol, 47 μL) to afford **15** (37.2 mg, 63% yield, l:b >99:1) as a pale-yellow oil. **^{1}H NMR (300 MHz, $CDCl_3$)** δ 10.13 (s, 1H), 8.31 (d, J = 1.5 Hz, 1H), 7.40 (dd, J = 8.5, 1.7 Hz, 1H), 7.24 (d, J = 8.4 Hz, 1H), 6.85 (dd, J = 17.5, 10.9 Hz, 1H), 5.78 (dd, J = 17.6, 1.0 Hz, 1H), 5.21 (dd, J = 10.9, 1.0 Hz, 1H), 3.70 (s, 3H), 3.14 – 2.93 (m, 2H), 1.73 – 1.60 (m, 2H), 1.48 – 1.36 (m, 2H), 1.37 – 1.22 (m, 8H), 0.92 – 0.83 (m, 3H). **^{13}C NMR (75 MHz, $CDCl_3$)** δ 184.2 (CH), 152.7 (C_q), 137.5 (CH), 137.0 (C_q), 132.9 (C_q), 126.0 (C_q), 121.5 (CH), 119.4 (CH), 114.2 (C_q), 112.6 (CH_2), 109.5 (CH), 31.9 (CH_2), 30.3 (CH_2), 30.0 (CH_3), 29.5 (CH_2), 29.4 (CH_2), 29.3 (CH_2), 24.6 (CH_2), 22.7 (CH_2), 14.2 (CH_3). **HRMS** (ESI) m/z $(M+H)^+$: calculated for $(C_{20}H_{28}NO)^+$: 298.2165, found: 298.2152.

5-Methoxy-1-methyl-2-octyl-1*H*-indole-3-carbaldehyde (16)

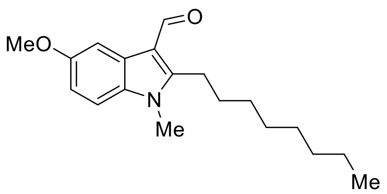

The **general procedure 4** was followed using indole substrate **1n** (0.2 mmol, 58.8 mg)

and 1-octene (**2a**) (0.3 mmol, 47 μL) to afford **16** (40.6 mg, 67% yield, l:b >99:1) as a yellow oil. **^{1}H NMR (300 MHz, CDCl$_3$)** δ 10.10 (s, 1H), 7.81 (d, *J* = 2.5 Hz, 1H), 7.18 (d, *J* = 8.8 Hz, 1H), 6.90 (dd, *J* = 8.9, 2.5 Hz, 1H), 3.89 (s, 3H), 3.68 (s, 3H), 3.08 – 2.97 (m, 2H), 1.70 – 1.60 (m, 2H), 1.48 – 1.35 (m, 2H), 1.36 – 1.20 (m, 8H), 0.91 – 0.83 (m, 3H). **^{13}C NMR (75 MHz, CDCl$_3$)** δ 184.1 (CH), 156.8 (C_q), 152.3 (C_q), 132.0 (C_q), 126.4 (C_q), 114.0 (C_q), 113.2 (CH), 110.2 (CH), 103.1 (CH), 56.0 (CH_3), 31.9 (CH_2), 30.4 (CH_2), 30.0 (CH_3), 29.5 (CH_2), 29.4 (CH_2), 29.3 (CH_2), 24.6 (CH_2), 22.7 (CH_2), 14.2 (CH_3). **HRMS** (ESI) m/z $(M+H)^+$: calculated for $(C_{19}H_{28}NO_2)^+$: 302.2115, found: 302.2118.

6-Chloro-1-methyl-2-octyl-1*H*-indole-3-carbaldehyde (17)

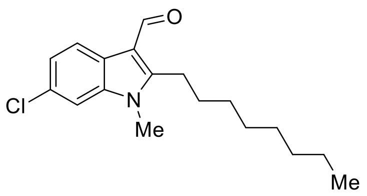

The **general procedure 4** was followed using indole substrate **1o** (0.2 mmol, 59.6 mg) and 1-octene (**2a**) (0.3 mmol, 47 μL) to afford **17** (49.2 mg, 81% yield, l:b >99:1) as a pale-yellow oil. **^{1}H NMR (300 MHz, CDCl$_3$)** δ 10.12 (s, 1H), 8.19 (d, *J* = 8.4 Hz, 1H), 7.30 (d, *J* = 1.8 Hz, 1H), 7.24 (dd, *J* = 8.4, 1.8 Hz, 1H), 3.69 (s, 3H), 3.12 – 3.01 (m, 2H), 1.76 – 1.59 (m, 2H), 1.50 – 1.34 (m, 2H), 1.37 – 1.22 (m, 8H), 0.92 – 0.80 (m, 3H). **^{13}C NMR (75 MHz, CDCl$_3$)** δ 184.2 (CH), 152.8 (C_q), 137.8 (C_q), 129.2 (C_q), 124.2 (C_q), 123.5 (CH), 122.2 (CH), 114.1 (C_q), 109.7 (CH), 31.9 (CH_2), 30.3 (CH_2), 30.0 (CH_3), 29.5 (CH_2), 29.4 (CH_2), 29.3 (CH_2), 24.6 (CH_2), 22.8 (CH_2), 14.2 (CH_3). **HRMS** (ESI) m/z $(M+H)^+$: calculated for $(C_{18}H_{25}ClNO)^+$: 306.1619, found: 306.1609.

7-Fluoro-1-methyl-2-octyl-1*H*-indole-3-carbaldehyde (18)

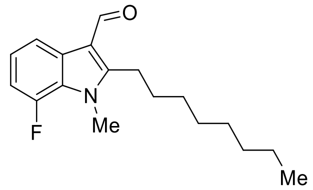

The **general procedure 4** was followed using indole substrate **1p** (0.2 mmol, 56.4 mg)

and 1-octene (**2a**) (0.3 mmol, 47 µL) to afford **18** (48.9 mg, 85% yield, l:b >99:1) as a yellow oil. **^{1}H NMR (300 MHz, CDCl$_3$)** δ 10.13 (d, *J* = 1.0 Hz, 1H), 8.04 (dd, *J* = 7.9, 1.0 Hz, 1H), 7.14 (td, *J* = 8.0, 4.6 Hz, 1H), 6.93 (ddd, *J* = 12.8, 8.0, 1.0 Hz, 1H), 3.92 (d, *J* = 1.5 Hz, 3H), 3.14 – 2.95 (m, 2H), 1.73 – 1.61 (m, 2H), 1.49 – 1.37 (m, 2H), 1.38 – 1.21 (m, 8H), 0.91 – 0.84 (m, 3H). **^{13}C NMR (75 MHz, CDCl$_3$)** δ 184.3 (CH), 152.9 (C_q), 149.7 (d, *J* = 244.7 Hz, C_q), 129.3 (d, *J* = 3.9 Hz, C_q), 124.8 (d, *J* = 8.5 Hz, C_q), 123.2 (d, *J* = 6.6 Hz, CH), 116.9 (d, *J* = 3.7 Hz, CH), 114.5 (d, *J* = 1.5 Hz, C_q), 109.3 (d, *J* = 18.2 Hz, CH), 32.5 (d, *J* = 7.4 Hz, CH_3), 31.9 (CH_2), 30.1 (CH_2), 29.5 (CH_2), 29.4 (CH_2), 29.2 (CH_2), 24.3 (CH_2), 22.7 (CH_2), 14.2 (CH_3). **^{19}F NMR (282 MHz, CDCl$_3$)** δ -135.31. **HRMS** (ESI) m/z $(M+H)^+$: calculated for $(C_{18}H_{25}FNO)^+$: 290.1915, found: 290.1909.

7-Ethyl-1-methyl-2-octyl-1*H*-indole-3-carbaldehyde (19)

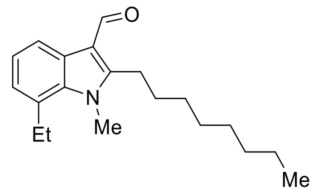

The **general procedure 4** was followed using indole substrate **1q** (0.2 mmol, 58.4 mg) and 1-octene (**2a**) (0.3 mmol, 47 µL) to afford **19** (49.7 mg, 83% yield, l:b >99:1) as a yellow oil. **^{1}H NMR (300 MHz, CDCl$_3$)** δ 10.15 (s, 1H), 8.21 (d, *J* = 7.8 Hz, 1H), 7.19 (t, *J* = 7.6 Hz, 1H), 7.05 (d, *J* = 7.2 Hz, 1H), 3.93 (s, 3H), 3.11 (q, *J* = 7.5 Hz, 2H), 3.07 – 3.01 (m, 2H), 1.71 – 1.59 (m, 2H), 1.50 – 1.24 (m, 13H), 0.93 – 0.85 (m, 3H). **^{13}C NMR (75 MHz, CDCl$_3$)** δ 184.3 (CH), 152.6 (C_q), 135.2 (C_q), 127.7 (C_q), 126.9 (C_q), 124.8 (CH), 122.9 (CH), 119.0 (CH), 113.9 (C_q), 32.8 (CH_3), 31.9 (CH_2), 30.1 (CH_2), 29.4 (CH_2), 29.4 (CH_2), 29.2 (CH_2), 25.9 (CH_2), 24.4 (CH_2), 22.7 (CH_2), 16.9 (CH_3), 14.2 (CH_3). **HRMS** (ESI) m/z $(M+H)^+$: calculated for $(C_{20}H_{30}NO)^+$: 300.2322, found: 300.2318.

2-Hexyl-1-methyl-1*H*-indole-3-carbaldehyde (20)

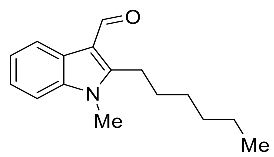

The **general procedure 4** was followed using indole substrate **1a** (0.2 mmol, 52.8 mg) and 1-hexene (**2b**) (0.3 mmol, 37 µL) to afford **20** (42.8 mg, 88% yield, l:b >99:1) as a yellow oil. **^{1}H NMR (300 MHz, CDCl$_3$)** δ 10.14 (s, 1H), 8.33 – 8.21 (m, 1H), 7.35 – 7.20 (m, 3H), 3.70 (s, 3H), 3.11 – 2.98 (m, 2H), 1.72 – 1.61 (m, 2H), 1.48 – 1.37 (m, 2H), 1.38 – 1.25 (m, 4H), 0.92 – 0.86 (m, 3H). **^{13}C NMR (75 MHz, CDCl$_3$)** δ 184.2 (CH), 152.2 (C_q), 137.2 (C_q), 125.8 (C_q), 123.2 (CH), 122.9 (CH), 121.1 (CH), 114.0 (C_q), 109.4 (CH), 31.6 (CH_2), 30.2 (CH_2), 29.8 (CH_3), 29.1 (CH_2), 24.5 (CH_2), 22.6 (CH_2), 14.1 (CH_3). **HRMS** (ESI) m/z $(M+H)^+$: calculated for $(C_{16}H_{22}NO)^+$: 244.1696, found: 244.1696. The analytical data are in accordance with those reported in the literature[9].

2-Decyl-1-methyl-1*H*-indole-3-carbaldehyde (21)

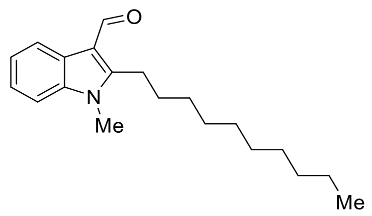

The **general procedure 4** was followed using indole substrate **1a** (0.2 mmol, 52.8 mg) and 1-decene (**2c**) (0.3 mmol, 57 µL) to afford **21** (55.2 mg, 92% yield, l:b >99:1) as a yellow oil. **^{1}H NMR (300 MHz, CDCl$_3$)** δ 10.14 (s, 1H), 8.33 – 8.25 (m, 1H), 7.36 – 7.20 (m, 3H), 3.70 (s, 3H), 3.16 – 2.93 (m, 2H), 1.74 – 1.57 (m, 2H), 1.51 – 1.36 (m, 2H), 1.38 – 1.17 (m, 12H), 0.95 – 0.82 (m, 3H). **^{13}C NMR (75 MHz, CDCl$_3$)** δ 184.2 (CH), 152.2 (C_q), 137.2 (C_q), 125.8 (C_q), 123.2 (CH), 122.9 (CH), 121.1 (CH), 114.0 (C_q), 109.4 (CH), 32.0 (CH_2), 30.2 (CH_2), 29.8 (CH_3), 29.6 (CH_2), 29.6 (CH_2), 29.5 (CH_2), 29.4 (CH_2), 29.4 (CH_2), 24.5 (CH_2), 22.8 (CH_2), 14.2 (CH_3). **HRMS** (ESI) m/z $(M+H)^+$: calculated for $(C_{20}H_{30}NO)^+$: 300.2322, found: 300.2323.

1-Methyl-2-(4-methylpentyl)-1*H*-indole-3-carbaldehyde (22)

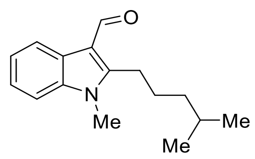

The **general procedure 4** was followed using indole substrate **1a** (0.2 mmol, 52.8 mg) and 4-methylpent-1-ene (**2d**) (0.3 mmol, 38 μL) to afford **22** (41.6 mg, 86% yield, l:b >99:1) as a yellow oil. **^{1}H NMR (300 MHz, $CDCl_3$)** δ 10.14 (s, 1H), 8.39 – 8.19 (m, 1H), 7.36 – 7.18 (m, 3H), 3.70 (s, 3H), 3.09 – 2.96 (m, 2H), 1.74 – 1.61 (m, 2H), 1.64 – 1.50 (m, 1H), 1.35 – 1.27 (m, 2H), 0.89 (s, 3H), 0.88 (s, 3H). **^{13}C NMR (75 MHz, $CDCl_3$)** δ 184.2 (CH), 152.2 (C_q), 137.1 (C_q), 125.7 (C_q), 123.2 (CH), 122.9 (CH), 121.1 (CH), 114.0 (C_q), 109.4 (CH), 38.6 (CH_2), 29.8 (CH_3), 28.1 (CH_2), 27.9 (CH), 24.7 (CH_2), 22.6 (CH_3). **HRMS** (ESI) m/z $(M+H)^+$: calculated for $(C_{16}H_{22}NO)^+$: 244.1696, found: 244.1698.

2-(2-Cyclohexylethyl)-1-methyl-1*H*-indole-3-carbaldehyde (23)

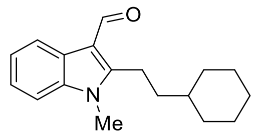

The **general procedure 4** was followed using indole substrate **1a** (0.2 mmol, 52.8 mg) and vinylcyclohexane (**2e**) (0.3 mmol, 41 μL) to afford **23** (44.8 mg, 83% yield, l:b >99:1) as an orange solid. **M.p.**: 80-82 °C. **^{1}H NMR (300 MHz, $CDCl_3$)** δ 10.13 (s, 1H), 8.32 – 8.15 (m, 1H), 7.37 – 7.18 (m, 3H), 3.68 (s, 3H), 3.09 – 2.96 (m, 2H), 1.85 – 1.62 (m, 5H), 1.57 – 1.47 (m, 2H), 1.48 – 1.27 (m, 1H), 1.28 – 1.12 (m, 3H), 1.06 – 0.88 (m, 2H). **^{13}C NMR (75 MHz, $CDCl_3$)** δ 184.1 (CH), 152.7 (C_q), 137.2 (C_q), 125.8 (C_q), 123.2 (CH), 122.9 (CH), 121.1 (CH), 113.7 (C_q), 109.4 (CH), 38.0 (CH_2), 37.7 (CH), 33.2 (CH_2), 29.8 (CH_3), 26.6 (CH_2), 26.3 (CH_2), 22.1 (CH_2). **HRMS** (ESI) m/z $(M+H)^+$: calculated for $(C_{18}H_{24}NO)^+$: 270.1852, found: 270.1850.

2-(3-Cyclohexylpropyl)-1-methyl-1*H*-indole-3-carbaldehyde (24)

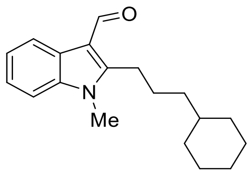

The **general procedure 4** was followed using indole substrate **1a** (0.2 mmol, 52.8 mg) and allylcyclohexane (**2f**) (0.3 mmol, 46 μL) to afford **24** (50.3 mg, 89% yield, l:b >99:1) as a white solid. **M.p.**: 101-104 °C. **^{1}H NMR (300 MHz, CDCl$_3$)** δ 10.13 (s, 1H), 8.36 – 8.18 (m, 1H), 7.33 – 7.18 (m, 3H), 3.69 (s, 3H), 3.08 – 2.97 (m, 2H), 1.75 – 1.58 (m, 7H), 1.36 – 1.06 (m, 6H), 0.93 – 0.79 (m, 2H). **^{13}C NMR (75 MHz, CDCl$_3$)** δ 184.2 (CH), 152.2 (C$_q$), 137.1 (C$_q$), 125.7 (C$_q$), 123.2 (CH), 122.9 (CH), 121.1 (CH), 114.0 (C$_q$), 109.4 (CH), 37.5 (CH), 37.1 (CH$_2$), 33.3 (CH$_2$), 29.8 (CH$_3$), 27.6 (CH$_2$), 26.7 (CH$_2$), 26.4 (CH$_2$), 24.7 (CH$_2$). **HRMS** (ESI) m/z (M+H)$^+$: calculated for ($C_{19}H_{26}NO$)$^+$: 284.2009, found: 284.2001.

2-Ethyl-1-methyl-1*H*-indole-3-carbaldehyde (25)

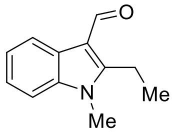

The **general procedure 4** was followed using indole substrate **1a** (0.2 mmol, 52.8 mg) and ethoxyethene (**2g**) (0.3 mmol, 29 μL) to afford **25** (24.8 mg, 66% yield) as a pale-yellow oil. **^{1}H NMR (300 MHz, CDCl$_3$)** δ 10.18 (s, 1H), 8.34 – 8.19 (m, 1H), 7.40 – 7.21 (m, 3H), 3.74 (s, 3H), 3.13 (q, *J* = 7.6 Hz, 2H), 1.34 (t, *J* = 7.6 Hz, 3H). **^{13}C NMR (75 MHz, CDCl$_3$)** δ 184.1 (CH), 153.3 (C$_q$), 137.2 (C$_q$), 125.9 (C$_q$), 123.3 (CH), 123.0 (CH), 121.2 (CH), 113.4 (C$_q$), 109.5 (CH), 29.7 (CH$_3$), 18.0 (CH$_2$), 14.8 (CH$_3$). **HRMS** (ESI) m/z (M+H)$^+$: calculated for ($C_{12}H_{14}NO$)$^+$: 188.1070, found: 188.1062.

1-Methyl-2-(3-phenylpropyl)-1*H*-indole-3-carbaldehyde (26)

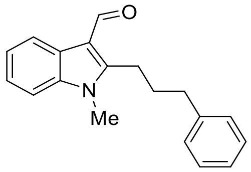

The **general procedure 4** was followed using indole substrate **1a** (0.2 mmol, 52.8 mg) and allylbenzene (**2h**) (0.3 mmol, 40 μL) to afford **26** (50.5 mg, 91% yield, l:b >99:1) as a pale-yellow solid. **M.p.**: 112-113 °C. **^{1}H NMR (300 MHz, $CDCl_3$)** δ 10.08 (s, 1H), 8.37 – 8.18 (m, 1H), 7.35 – 7.27 (m, 5H), 7.25 – 7.18 (m, 3H), 3.59 (s, 3H), 3.09 – 3.01 (m, 2H), 2.77 (t, *J* = 7.5 Hz, 2H), 2.06 – 1.92 (m, 2H). **^{13}C NMR (75 MHz, $CDCl_3$)** δ 184.1 (CH), 151.5 (C_q), 140.9 (C_q), 137.1 (C_q), 128.6 (CH), 128.5 (CH), 126.3 (CH), 125.8 (C_q), 123.2 (CH), 122.9 (CH), 121.0 (CH), 114.0 (C_q), 109.4 (CH), 35.4 (CH_2), 31.5 (CH_2), 29.7 (CH_3), 23.8 (CH_2). **HRMS** (ESI) m/z $(M+H)^+$: calculated for $(C_{19}H_{20}NO)^+$: 278.1539, found: 278.1542. The analytical data are in accordance with those reported in the literature[10].

2-(3-(4-Fluorophenyl)propyl)-1-methyl-1*H*-indole-3-carbaldehyde (27)

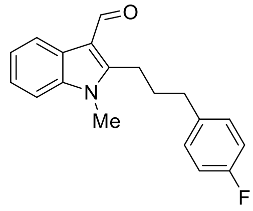

The **general procedure 4** was followed using indole substrate **1a** (0.2 mmol, 52.8 mg) and 1-allyl-4-fluorobenzene (**2i**) (0.3 mmol, 40 μL) at 30 Hz for 270 min to afford **27** (33.1 mg, 56% yield, l:b >99:1) as a pale-yellow solid. **M.p.**: 115-117 °C. **^{1}H NMR (300 MHz, $CDCl_3$)** δ 10.11 (s, 1H), 8.34 – 8.16 (m, 1H), 7.33 – 7.27 (m, 3H), 7.19 – 7.11 (m, 2H), 7.03 – 6.95 (m, 2H), 3.64 (s, 3H), 3.14 – 3.01 (m, 2H), 2.74 (t, *J* = 7.5 Hz, 2H), 2.07 – 1.90 (m, 2H). **^{13}C NMR (75 MHz, $CDCl_3$)** δ 184.1 (CH), 161.6 (d, *J* = 244.2 Hz, C_q), 151.1 (C_q), 137.2 (C_q), 136.6 (d, *J* = 3.3 Hz, C_q), 129.8 (d, *J* = 7.8 Hz, CH), 125.9 (C_q), 123.2 (d, *J* = 23.8 Hz, CH), 121.0 (CH), 115.6 (CH), 115.3 (CH), 114.1 (C_q), 109.5 (CH), 34.6 (CH_2), 31.7 (CH_2), 29.8 (CH_3), 24.0 (CH_2). **^{19}F NMR (282 MHz, $CDCl_3$)** δ -116.93. **HRMS** (ESI) m/z $(M+H)^+$: calculated for $(C_{19}H_{19}FNO)^+$: 296.1445, found: 296.1436.

2-(3-([1,1'-Biphenyl]-4-yl)propyl)-1-methyl-1*H*-indole-3-carbaldehyde (28)

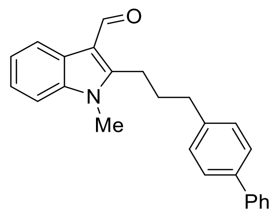

The **general procedure 4** was followed using indole substrate **1a** (0.2 mmol, 52.8 mg) and 4-allyl-1,1'-biphenyl (**2j**) (0.3 mmol, 58.3 mg) at 30 Hz for 270 min to afford **28** (59.1 mg, 84% yield, l:b >99:1) as a pale-yellow oil. **^{1}H NMR (300 MHz, CDCl$_3$)** δ 10.07 (s, 1H), 8.25 – 8.15 (m, 1H), 7.56 – 7.42 (m, 4H), 7.42 – 7.30 (m, 2H), 7.32 – 7.15 (m, 6H), 3.57 (s, 3H), 3.12 – 2.96 (m, 2H), 2.74 (t, J = 7.5 Hz, 2H), 2.07 – 1.90 (m, 2H). **^{13}C NMR (75 MHz, CDCl$_3$)** δ 184.2 (CH), 151.3 (C$_q$), 141.0 (C$_q$), 140.1 (C$_q$), 139.3 (C$_q$), 137.2 (C$_q$), 128.9 (CH), 128.9 (CH), 127.4 (CH), 127.3 (CH), 127.1 (CH), 125.9 (C$_q$), 123.3 (CH), 123.0 (CH), 121.1 (CH), 114.1 (C$_q$), 109.5 (CH), 35.1 (CH$_2$), 31.5 (CH$_2$), 29.8 (CH$_3$), 24.1 (CH$_2$). **HRMS** (ESI) m/z (M+H)$^+$: calculated for ($C_{25}H_{24}NO$)$^+$: 354.1852, found: 354.1848.

2-(3-(4-Methoxyphenyl)propyl)-1-methyl-1*H*-indole-3-carbaldehyde (29)

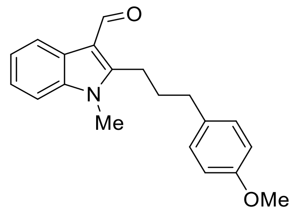

The **general procedure 4** was followed using indole substrate **1a** (0.2 mmol, 52.8 mg) and 1-allyl-4-methoxybenzene (**2k**) (0.3 mmol, 46 µL) to afford **29** (58.3 mg, 95% yield, l:b >99:1) as a pale-yellow solid. **M.p.**: 123-124 °C. **^{1}H NMR (300 MHz, CDCl$_3$)** δ 10.10 (s, 1H), 8.33 – 8.23 (m, 1H), 7.34 – 7.25 (m, 3H), 7.11 (d, J = 8.6 Hz, 2H), 6.85 (d, J = 8.6 Hz, 2H), 3.80 (s, 3H), 3.62 (s, 3H), 3.13 – 3.01 (m, 2H), 2.71 (t, J = 7.4 Hz, 2H), 2.08 – 1.90 (m, 2H). **^{13}C NMR (75 MHz, CDCl$_3$)** δ 184.2 (CH), 158.2 (C$_q$), 151.6 (C$_q$), 137.2 (C$_q$), 133.0 (C$_q$), 129.4 (CH), 125.8 (C$_q$), 123.3 (CH), 123.0 (CH), 121.1 (CH), 114.1 (CH), 109.5 (CH), 55.4 (CH$_3$), 34.5 (CH$_2$), 31.8 (CH$_2$), 29.8 (CH$_3$), 23.9 (CH$_2$). **HRMS** (ESI) m/z (M+H)$^+$: calculated for ($C_{20}H_{22}NO_2$)$^+$: 308.1645, found: 308.1649.

1-Methyl-2-(3-(naphthalen-1-yl)propyl)-1*H*-indole-3-carbaldehyde (30)

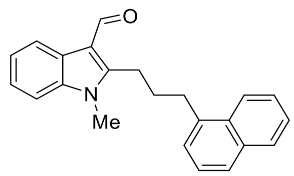

The **general procedure 4** was followed using indole substrate **1a** (0.2 mmol, 52.8 mg) and 1-allylnaphthalene (**2l**) (0.3 mmol, 49 μL) to afford **30** (52.4 mg, 80% yield, l:b >99:1) as a yellow oil. **^{1}H NMR (300 MHz, CDCl$_3$)** δ 10.16 (s, 1H), 8.33 – 8.21 (m, 1H), 8.00 – 7.89 (m, 1H), 7.92 – 7.81 (m, 1H), 7.74 (d, J = 8.1 Hz, 1H), 7.55 – 7.42 (m, 2H), 7.47 – 7.35 (m, 1H), 7.38 – 7.23 (m, 4H), 3.53 (s, 3H), 3.29 – 3.12 (m, 4H), 2.23 – 2.06 (m, 2H). **^{13}C NMR (75 MHz, CDCl$_3$)** δ 184.2 (CH), 151.3 (C$_q$), 137.2 (C$_q$), 137.0 (C$_q$), 134.1 (C$_q$), 131.8 (C$_q$), 129.1 (CH), 127.2 (CH), 126.3 (CH), 126.2 (CH), 125.9 (C$_q$), 125.8 (CH), 125.6 (CH), 123.5 (CH), 123.3 (CH), 123.0 (CH), 121.0 (CH), 114.1 (C$_q$), 109.5 (CH), 32.6 (CH$_2$), 30.9 (CH$_2$), 29.7 (CH$_3$), 24.4 (CH$_2$). **HRMS** (ESI) m/z (M+H)$^+$: calculated for ($C_{23}H_{22}NO$)$^+$: 328.1696, found: 328.1691.

1-Methyl-2-(2-(trimethylsilyl)ethyl)-1*H*-indole-3-carbaldehyde (31)

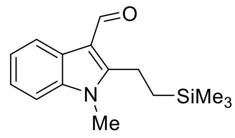

The **general procedure 4** was followed using indole substrate **1a** (0.2 mmol, 52.8 mg) and trimethyl(vinyl)silane (**2m**) (0.3 mmol, 44 μL) to afford **31** (42.7 mg, 82% yield, l:b >99:1) as a pale-yellow solid. **M.p.**: 120-122 °C. **^{1}H NMR (300 MHz, CDCl$_3$)** δ 10.16 (s, 1H), 8.31 – 8.21 (m, 1H), 7.39 – 7.19 (m, 3H), 3.71 (s, 3H), 3.17 – 2.90 (m, 2H), 0.98 – 0.78 (m, 2H), 0.11 (s, 9H). **^{13}C NMR (75 MHz, CDCl$_3$)** δ 183.9 (CH), 154.7 (C$_q$), 137.2 (C$_q$), 125.9 (C$_q$), 123.2 (CH), 123.0 (CH), 121.2 (CH), 112.8 (C$_q$), 109.4 (CH), 29.7 (CH$_3$), 19.0 (CH$_2$), 18.2 (CH$_2$), -1.8 (CH$_3$). **HRMS** (ESI) m/z (M+H)$^+$: calculated for ($C_{15}H_{22}NOSi$)$^+$: 260.1465, found: 260.1463.

1-Methyl-2-(2-(triethylsilyl)ethyl)-1*H*-indole-3-carbaldehyde (32)

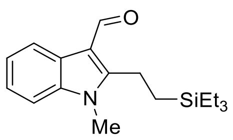

The **general procedure 4** was followed using indole substrate **1a** (0.2 mmol, 52.8 mg) and triethyl(vinyl)silane (**2n**) (0.3 mmol, 55 μL) to afford **32** (46.5 mg, 77% yield, l:b >99:1) as a white solid. **M.p.**: 111-113 °C. **^{1}H NMR (300 MHz, CDCl$_3$)** δ 10.17 (s, 1H), 8.33 – 8.17 (m, 1H), 7.36 – 7.25 (m, 3H), 3.73 (s, 3H), 3.14 – 2.94 (m, 2H), 1.01 (t, J = 7.9 Hz, 9H), 0.94 – 0.80 (m, 2H), 0.64 (q, J = 7.9 Hz, 6H). **^{13}C NMR (75 MHz, CDCl$_3$)** δ 183.9 (CH), 154.9 (C$_q$), 137.2 (C$_q$), 125.9 (C$_q$), 123.2 (CH), 123.0 (CH), 121.2 (CH), 112.8 (C$_q$), 109.4 (CH), 29.7 (CH$_3$), 19.0 (CH$_2$), 13.3 (CH$_2$), 7.6 (CH$_3$), 3.2 (CH$_2$). **HRMS** (ESI) m/z (M+H)$^+$: calculated for $(C_{18}H_{28}NOSi)^+$: 302.1935, found: 302.1932.

1-Methyl-2-(2-(triphenylsilyl)ethyl)-1*H*-indole-3-carbaldehyde (33)

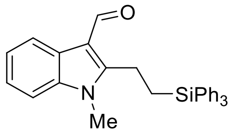

The **general procedure 4** was followed using indole substrate **1a** (0.2 mmol, 52.8 mg) and triphenyl(vinyl)silane (**2o**) (0.3 mmol, 85.9 mg) at 30 Hz for 270 min to afford **33** (38.5 mg, 43% yield, l:b >99:1) as a white solid. **M.p.**: 180-182 °C. **^{1}H NMR (300 MHz, CDCl$_3$)** δ 10.03 (s, 1H), 8.30 – 8.19 (m, 1H), 7.65 – 7.58 (m, 6H), 7.51 – 7.38 (m, 9H), 7.31 – 7.23 (m, 3H), 3.52 (s, 3H), 3.23 – 3.09 (m, 2H), 1.81 – 1.64 (m, 2H). **^{13}C NMR (75 MHz, CDCl$_3$)** δ 184.0 (CH), 153.9 (C$_q$), 137.2 (C$_q$), 135.6 (CH), 133.9 (C$_q$), 130.1 (CH), 128.4 (CH), 125.9 (C$_q$), 123.3 (CH), 123.0 (CH), 121.2 (CH), 113.1 (C$_q$), 109.4 (CH), 29.6 (CH$_3$), 19.2 (CH$_2$), 15.1 (CH$_2$). **HRMS** (ESI) m/z (M+H)$^+$: calculated for $(C_{30}H_{28}NOSi)^+$: 446.1935, found: 446.1929.

1-Methyl-2-(3-(trimethylsilyl)propyl)-1*H*-indole-3-carbaldehyde (34)

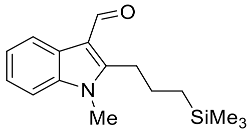

The **general procedure 4** was followed using indole substrate **1a** (0.2 mmol, 52.8 mg) and allyltrimethylsilane (**2p**) (0.3 mmol, 48 μL) to afford **34** (31.0 mg, 57% yield, l:b >99:1) as a white solid. **M.p.**: 102-103 °C. **^{1}H NMR (300 MHz, CDCl$_3$)** δ 10.16 (s, 1H), 8.39 – 8.19 (m, 1H), 7.36 – 7.24 (m, 3H), 3.74 (s, 3H), 3.12 (t, *J* = 7.5 Hz, 2H), 1.78 – 1.61 (m, 2H), 0.69 – 0.54 (m, 2H), -0.02 (s, 9H). **^{13}C NMR (75 MHz, CDCl$_3$)** δ 184.4 (CH), 151.9 (C$_q$), 137.2 (C$_q$), 125.8 (C$_q$), 123.3 (CH), 123.0 (CH), 121.3 (CH), 114.3 (C$_q$), 109.5 (CH), 30.0 (CH$_3$), 28.3 (CH$_2$), 25.1 (CH$_2$), 16.9 (CH$_2$), -1.6 (CH$_3$). **HRMS** (ESI) m/z (M+H)$^+$: calculated for $(C_{16}H_{24}NOSi)^+$: 274.1622, found: 274.1612.

2-(2-(9*H*-Carbazol-9-yl)ethyl)-1-methyl-1*H*-indole-3-carbaldehyde (35)

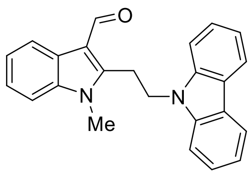

The **general procedure 4** was followed using indole substrate **1a** (0.2 mmol, 52.8 mg) and 9-vinyl-9*H*-carbazole (**2q**) (0.3 mmol, 58.0 mg) to afford **35** (49.7 mg, 71% yield, l:b >99:1) as a white solid. **M.p.**: 178-180 °C. **^{1}H NMR (300 MHz, CDCl$_3$)** δ 10.42 (s, 1H), 8.16 (d, *J* = 7.3 Hz, 1H), 8.12 – 8.02 (m, 2H), 7.36 – 7.26 (m, 3H), 7.24 – 7.17 (m, 5H), 7.06 (dt, *J* = 7.8, 1.0 Hz, 1H), 4.70 (t, *J* = 6.6 Hz, 2H), 3.64 (t, *J* = 6.6 Hz, 2H), 2.99 (s, 3H). **^{13}C NMR (75 MHz, CDCl$_3$)** δ 184.2 (CH), 146.9 (C$_q$), 139.9 (C$_q$), 137.0 (C$_q$), 126.6 (C$_q$), 126.1 (CH), 123.4 (CH), 123.1 (C$_q$), 123.0 (CH), 120.6 (CH), 119.5 (CH), 119.4 (CH), 113.9 (C$_q$), 109.9 (CH), 108.3 (CH), 43.1 (CH$_2$), 29.2 (CH$_3$), 25.0 (CH$_2$). **HRMS** (ESI) m/z (M+H)$^+$: calculated for $(C_{24}H_{21}N_2O)^+$: 353.1648, found: 353.1654.

2-(3-(9*H*-Carbazol-9-yl)propyl)-1-methyl-1*H*-indole-3-carbaldehyde (36)

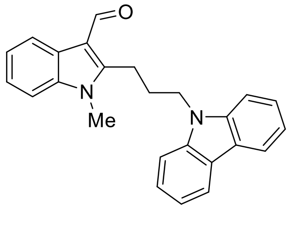

The **general procedure 4** was followed using indole substrate **1a** (0.2 mmol, 52.8 mg) and 9-allyl-9*H*-carbazole (**2r**) (0.3 mmol, 62.2 mg) at 30 Hz for 270 min to afford **36** (47.1 mg, 64% yield, l:b >99:1) as a pale-yellow solid. **M.p.**: 174-176 °C. **^{1}H NMR (300 MHz, $CDCl_3$)** δ 10.12 (s, 1H), 8.22 – 8.11 (m, 1H), 8.11 (d, *J* = 7.8 Hz, 2H), 7.50 – 7.42 (m, 2H), 7.37 (d, *J* = 8.1 Hz, 2H), 7.28 – 7.21 (m, 4H), 7.23 – 7.13 (m, 1H), 4.45 (t, *J* = 6.8 Hz, 2H), 3.22 (s, 3H), 3.06 – 2.95 (m, 2H), 2.30 – 2.14 (m, 2H). **^{13}C NMR (75 MHz, $CDCl_3$)** δ 184.0 (CH), 149.5 (C_q), 140.4 (C_q), 137.0 (C_q), 126.1 (C_q), 126.0 (CH), 123.3 (CH), 123.1 (C_q), 123.0 (CH), 120.7 (CH), 120.3 (CH), 119.4 (CH), 113.7 (C_q), 109.6 (CH), 108.6 (CH), 42.3 (CH_2), 29.3 (CH_3), 29.0 (CH_2), 22.3 (CH_2). **HRMS** (ESI) m/z $(M+H)^+$: calculated for $(C_{25}H_{23}N_2O)^+$: 367.1805, found: 367.1799.

2-(2-(1*H*-Indol-1-yl)ethyl)-1-methyl-1*H*-indole-3-carbaldehyde (37)

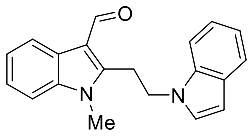

The **general procedure 4** was followed using indole substrate **1a** (0.2 mmol, 52.8 mg) and 1-vinyl-1*H*-indole (**2s**) (0.3 mmol, 42.9 mg) to afford **37** (47.4 mg, 78% yield, l:b >99:1) as a pale-yellow oil. **^{1}H NMR (300 MHz, $CDCl_3$)** δ 10.31 (s, 1H), 8.16 (dd, *J* = 6.5, 1.9 Hz, 1H), 7.66 – 7.56 (m, 1H), 7.38 – 7.20 (m, 3H), 7.21 – 7.04 (m, 3H), 6.66 (d, *J* = 3.2 Hz, 1H), 6.39 (dd, *J* = 3.2, 0.9 Hz, 1H), 4.61 – 4.50 (m, 2H), 3.64 – 3.53 (m, 2H), 2.90 (s, 3H). **^{13}C NMR (75 MHz, $CDCl_3$)** δ 184.1 (CH), 146.9 (C_q), 137.0 (C_q), 135.7 (C_q), 128.9 (C_q), 127.7 (CH), 126.6 (C_q), 123.3 (CH), 123.0 (CH), 122.1 (CH), 121.3 (CH), 119.8 (CH), 119.4 (CH), 113.6 (C_q), 110.1 (CH), 108.9 (CH), 102.1 (CH), 46.7 (CH_2), 29.0 (CH_3), 26.6 (CH_2). **HRMS** (ESI) m/z $(M+H)^+$: calculated for $(C_{20}H_{19}N_2O)^+$: 303.1492, found: 303.1482.

2-(3-(1*H*-Indol-1-yl)propyl)-1-methyl-1*H*-indole-3-carbaldehyde (38)

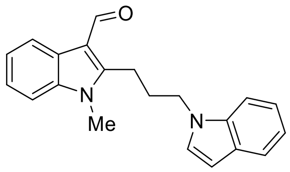

The **general procedure 4** was followed using indole substrate **1a** (0.2 mmol, 52.8 mg) and 1-allyl-1*H*-indole (**2t**) (0.3 mmol, 47.2 mg) at 30 Hz for 270 min to afford **38** (42.1 mg, 67% yield, l:b >99:1) as a yellow oil. **^{1}H NMR (300 MHz, CDCl$_3$)** δ 10.16 (s, 1H), 8.25 – 8.13 (m, 1H), 7.66 (dt, *J* = 7.8, 1.0 Hz, 1H), 7.35 – 7.25 (m, 4H), 7.21 (ddd, *J* = 8.2, 7.1, 1.3 Hz, 1H), 7.17 – 7.08 (m, 2H), 6.55 (dd, *J* = 3.2, 0.9 Hz, 1H), 4.30 (t, *J* = 6.6 Hz, 2H), 3.38 (s, 3H), 3.15 – 2.98 (m, 2H), 2.31 – 2.15 (m, 2H). **^{13}C NMR (75 MHz, CDCl$_3$)** δ 184.0 (CH), 149.5 (C$_q$), 137.1 (C$_q$), 136.1 (C$_q$), 128.9 (C$_q$), 127.7 (CH), 126.1 (C$_q$), 123.4 (CH), 123.0 (CH), 121.9 (CH), 121.4 (CH), 120.4 (CH), 119.7 (CH), 113.9 (C$_q$), 109.7 (CH), 109.3 (CH), 101.9 (CH), 45.7 (CH$_2$), 30.4 (CH$_2$), 29.5 (CH$_3$), 22.1 (CH$_2$). **HRMS** (ESI) m/z (M+H)$^+$: calculated for (C$_{21}$H$_{21}$N$_2$O)$^+$: 317.1648, found: 317.1640.

2-(2-(*tert*-Butoxy)ethyl)-1-methyl-1*H*-indole-3-carbaldehyde (39)

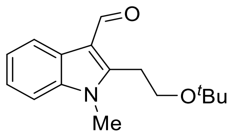

The **general procedure 4** was followed using indole substrate **1a** (0.2 mmol, 52.8 mg) and 2-methyl-2-(vinyloxy)propane (**2u**) (0.3 mmol, 39 μL) at 30 Hz for 270 min to afford **39** (35.1 mg, 68% yield, l:b >99:1) as a pale-yellow oil. **^{1}H NMR (300 MHz, CDCl$_3$)** δ 10.20 (s, 1H), 8.34 – 8.16 (m, 1H), 7.42 – 7.24 (m, 3H), 3.80 (s, 3H), 3.69 (t, *J* = 6.3 Hz, 2H), 3.33 (t, *J* = 6.3 Hz, 2H), 1.08 (s, 9H). **^{13}C NMR (75 MHz, CDCl$_3$)** δ 184.4 (CH), 149.8 (C$_q$), 137.2 (C$_q$), 126.1 (C$_q$), 123.2 (CH), 122.9 (CH), 120.7 (CH), 114.0 (C$_q$), 109.7 (CH), 73.5 (C$_q$), 62.0 (CH$_2$), 30.5 (CH$_3$), 27.5 (CH$_3$), 26.6 (CH$_2$). **HRMS** (ESI) m/z (M+H)$^+$: calculated for (C$_{16}$H$_{22}$NO$_2$)$^+$: 260.1645, found: 260.1638.

5-(3-Formyl-1-methyl-1*H*-indol-2-yl)pentyl acetate (40)

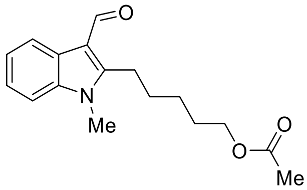

The **general procedure 4** was followed using indole substrate **1a** (0.2 mmol, 52.8 mg)

and pent-4-en-1-yl acetate (**2v**) (0.3 mmol, 42 μL) to afford **40** (49.6 mg, 86% yield, l:b >99:1) as a pale-yellow oil. **^{1}H NMR (300 MHz, CDCl$_3$)** δ 10.09 (s, 1H), 8.25 – 8.13 (m, 1H), 7.30 – 7.16 (m, 3H), 3.98 (t, J = 6.5 Hz, 2H), 3.65 (s, 3H), 3.09 – 2.96 (m, 2H), 1.95 (s, 3H), 1.74 – 1.52 (m, 4H), 1.50 – 1.33 (m, 2H). **^{13}C NMR (75 MHz, CDCl$_3$)** δ 184.1 (CH), 171.3 (C$_q$), 151.3 (C$_q$), 137.2 (C$_q$), 125.9 (C$_q$), 123.3 (CH), 123.0 (CH), 121.0 (CH), 114.1 (C$_q$), 109.5 (CH), 64.1 (CH$_2$), 29.9 (CH$_3$), 29.7 (CH$_2$), 28.5 (CH$_2$), 25.8 (CH$_2$), 24.5 (CH$_2$), 21.1 (CH$_3$). **HRMS** (ESI) m/z (M+H)$^+$: calculated for ($C_{17}H_{22}NO_3$)$^+$: 288.1594, found: 288.1590.

2-(6-Hydroxyhexyl)-1-methyl-1*H*-indole-3-carbaldehyde (41)

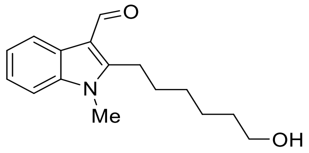

The **general procedure 4** was followed using indole substrate **1a** (0.2 mmol, 52.8 mg) and *tert*-butyl(hex-5-en-1-yloxy)dimethylsilane (**2w**) (0.3 mmol, 64.3 mg) to afford **41** (47.9 mg, 92% yield, l:b >99:1) as a pale-yellow oil. **^{1}H NMR (300 MHz, CDCl$_3$)** δ 10.13 (s, 1H), 8.31 – 8.21 (m, 1H), 7.32 – 7.24 (m, 3H), 3.70 (s, 3H), 3.63 (t, J = 6.4 Hz, 2H), 3.14 – 2.98 (m, 2H), 1.77 (br, 1H), 1.74 – 1.62 (m, 2H), 1.64 – 1.49 (m, 2H), 1.50 – 1.36 (m, 4H). **^{13}C NMR (75 MHz, CDCl$_3$)** δ 184.3 (CH), 152.0 (C$_q$), 137.2 (C$_q$), 125.8 (C$_q$), 123.3 (CH), 123.0 (CH), 121.0 (CH), 114.0 (C$_q$), 109.5 (CH), 62.8 (CH$_2$), 32.6 (CH$_2$), 30.1 (CH$_2$), 29.9 (CH$_3$), 29.1 (CH$_2$), 25.6 (CH$_2$), 24.5 (CH$_2$). **HRMS** (ESI) m/z (M+H)$^+$: calculated for ($C_{16}H_{22}NO_2$)$^+$: 260.1645, found: 260.1641.

2-(5-(((1*R*,2*S*,5*R*)-2-Isopropyl-5-methylcyclohexyl)oxy)pentyl)-1-methyl-1*H*-indole-3-carbaldehyde (42)

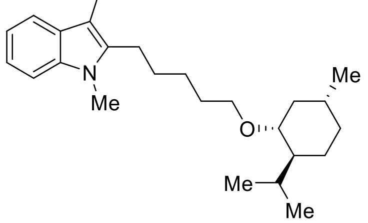

The **general procedure 4** was followed using indole substrate **1a** (0.2 mmol, 52.8 mg)

and (1*S*,2*R*,4*R*)-1-isopropyl-4-methyl-2-(pent-4-en-1-yloxy)cyclohexane (**2x**) (0.3 mmol, 67.3 mg) to afford **42** (52.2 mg, 68% yield, l:b >99:1) as a colorless oil. **^{1}H NMR (300 MHz, CDCl$_3$)** δ 10.15 (s, 1H), 8.35 – 8.17 (m, 1H), 7.34 – 7.25 (m, 3H), 3.71 (s, 3H), 3.69 – 3.53 (m, 1H), 3.32 – 3.18 (m, 1H), 3.13 – 3.05 (m, 2H), 2.98 (td, *J* = 10.6, 4.1 Hz, 1H), 2.25 – 2.08 (m, 1H), 2.14 – 2.00 (m, 1H), 1.77 – 1.45 (m, 8H), 1.42 – 1.27 (m, 1H), 1.25 – 1.14 (m, 1H), 1.03 – 0.78 (m, 9H), 0.75 (d, *J* = 6.9 Hz, 3H). **^{13}C NMR (75 MHz, CDCl$_3$)** δ 184.1 (CH), 151.9 (C$_q$), 137.2 (C$_q$), 125.8 (C$_q$), 123.2 (CH), 123.0 (CH), 121.1 (CH), 114.0 (C$_q$), 109.4 (CH), 79.3 (CH), 68.2 (CH$_2$), 48.4 (CH), 40.6 (CH$_2$), 34.7 (CH$_2$), 31.7 (CH), 30.2 (CH$_2$), 30.2 (CH$_2$), 29.9 (CH$_3$), 26.3 (CH$_2$), 25.8 (CH), 24.6 (CH$_2$), 23.5 (CH$_2$), 22.5 (CH$_3$), 21.1 (CH$_3$), 16.4 (CH$_3$). **HRMS** (ESI) m/z (M+H)$^+$: calculated for $(C_{25}H_{38}NO_2)^+$: 384.2897, found: 384.2891.

1-Methyl-2-(6-(((4*R*)-1,7,7-trimethylbicyclo[2.2.1]heptan-2-yl)oxy)hexyl)-1*H*-indole-3-carbaldehyde (43)

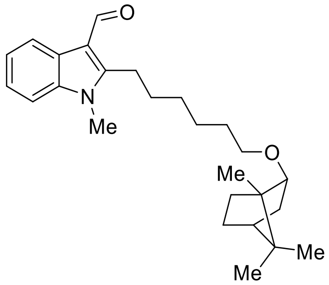

The **general procedure 4** was followed using indole substrate **1a** (0.2 mmol, 52.8 mg) and (4*R*)-2-(hex-5-en-1-yloxy)-1,7,7-trimethylbicyclo[2.2.1]heptane (**2y**) (0.3 mmol, 70.9 mg) to afford **43** (53.9 mg, 68% yield, l:b >99:1) as a pale-yellow oil. **^{1}H NMR (300 MHz, CDCl$_3$)** δ 10.16 (s, 1H), 8.34 – 8.22 (m, 1H), 7.37 – 7.24 (m, 3H), 3.72 (s, 3H), 3.52 (ddd, *J* = 9.4, 3.3, 1.8 Hz, 1H), 3.49 – 3.35 (m, 1H), 3.39 – 3.25 (m, 1H), 3.12 – 3.05 (m, 2H), 2.17 – 2.00 (m, 1H), 2.04 – 1.88 (m, 1H), 1.75 – 1.36 (m, 10H), 1.28 – 1.10 (m, 2H), 0.96 (dd, *J* = 12.9, 3.3 Hz, 1H), 0.85 (s, 3H), 0.83 (s, 6H). **^{13}C NMR (75 MHz, CDCl$_3$)** δ 184.2 (CH), 152.1 (C$_q$), 137.2 (C$_q$), 125.8 (C$_q$), 123.2 (CH), 123.0 (CH), 121.2 (CH), 114.1 (C$_q$), 109.4 (CH), 84.7 (CH), 69.9 (CH$_2$), 49.3 (C$_q$), 47.9 (C$_q$), 45.1 (CH), 36.5 (CH$_2$), 30.3 (CH$_2$), 30.0 (CH$_2$), 29.9 (CH$_3$), 29.3 (CH$_2$), 28.4 (CH$_2$), 26.8 (CH$_2$), 26.2 (CH$_2$), 24.6 (CH$_2$), 19.9 (CH$_3$), 19.0 (CH$_3$), 14.2 (CH$_3$).

HRMS (ESI) m/z $(M+H)^+$: calculated for $(C_{26}H_{38}NO_2)^+$: 396.2897, found: 396.2899.

(*E*)-2-(6-((3,7-dimethylocta-2,6-dien-1-yl)oxy)hexyl)-1-methyl-1*H*-indole-3-carbaldehyde (44)

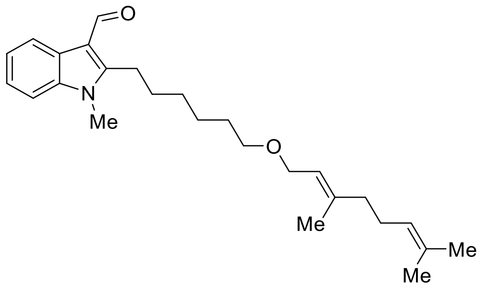

The **general procedure 4** was followed using indole substrate **1a** (0.2 mmol, 52.8 mg) and (*E*)-1-(hex-5-en-1-yloxy)-3,7-dimethylocta-2,6-diene (**2z**) (0.3 mmol, 70.9 mg) to afford **44** (55.3 mg, 70% yield, l:b >99:1) as a pale-yellow oil. **^{1}H NMR (300 MHz, CDCl$_3$)** δ 10.14 (s, 1H), 8.33 – 8.21 (m, 1H), 7.34 – 7.24 (m, 3H), 5.40 – 5.28 (m, 1H), 5.15 – 5.03 (m, 1H), 4.01 – 3.91 (m, 2H), 3.70 (s, 3H), 3.40 (t, J = 6.5 Hz, 2H), 3.13 – 3.00 (m, 2H), 2.16 – 1.98 (m, 4H), 1.73 – 1.53 (m, 13H), 1.51 – 1.34 (m, 4H). **^{13}C NMR (75 MHz, CDCl$_3$)** δ 184.2 (CH), 152.0 (C_q), 140.0 (C_q), 137.2 (C_q), 131.7 (C_q), 125.8 (C_q), 124.1 (CH), 123.2 (CH), 122.9 (CH), 121.1 (CH), 121.1 (CH), 114.0 (C_q), 109.4 (CH), 70.1 (CH_2), 67.4 (CH_2), 39.7 (CH_2), 30.2 (CH_2), 29.8 (CH_3), 29.8 (CH_2), 29.3 (CH_2), 26.5 (CH_2), 26.1 (CH_2), 25.8 (CH_3), 24.5 (CH_2), 17.8 (CH_3), 16.6 (CH_3). **HRMS** (ESI) m/z $(M+H)^+$: calculated for $(C_{26}H_{38}NO_2)^+$: 396.2897, found: 396.2895.

2-(3-(3,4-Dimethoxyphenyl)propyl)-1-methyl-1*H*-indole-3-carbaldehyde (45)

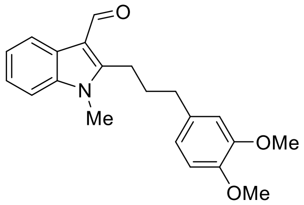

The **general procedure 4** was followed using indole substrate **1a** (0.2 mmol, 52.8 mg) and 4-allyl-1,2-dimethoxybenzene (**2aa**) (0.3 mmol, 52 μL) at 30 Hz for 270 min to afford **45** (58.4 mg, 87% yield, l:b >99:1) as a colorless oil. **^{1}H NMR (300 MHz, CDCl$_3$)** δ 10.11 (s, 1H), 8.31 – 8.24 (m, 1H), 7.31 – 7.27 (m, 3H), 6.81 (d, J = 8.1 Hz, 1H), 6.74 (dd, J = 8.1, 2.0 Hz, 1H), 6.70 (d, J = 2.0 Hz, 1H), 3.87 (s, 3H), 3.86 (s, 3H),

3.63 (s, 3H), 3.13 – 3.03 (m, 2H), 2.71 (t, *J* = 7.5 Hz, 2H), 2.06 – 1.92 (m, 2H). **^{13}C NMR (75 MHz, CDCl$_3$)** δ 184.2 (CH), 151.5 (C_q), 149.0 (C_q), 147.5 (C_q), 137.2 (C_q), 133.5 (C_q), 125.8 (C_q), 123.3 (CH), 123.0 (CH), 121.0 (CH), 120.3 (CH), 114.1 (C_q), 111.7 (CH), 111.4 (CH), 109.5 (CH), 56.0 (CH_3), 56.0 (CH_3), 35.0 (CH_2), 31.6 (CH_2), 29.8 (CH_3), 23.9 (CH_2). **HRMS** (ESI) m/z $(M+H)^+$: calculated for $(C_{21}H_{24}NO_3)^+$: 338.1751, found: 338.1751.

1-Methyl-2-(3-((8*R*,9*S*,13*S*,14*S*)-13-methyl-17-oxo-7,8,9,11,12,13,14,15,16,17-decahydro-6*H*-cyclopenta[*a*]phenanthren-2-yl)propyl)-1*H*-indole-3-carbaldehyde (46)

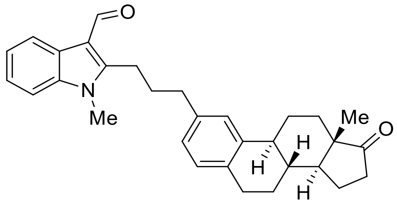

The **general procedure 4** was followed using indole substrate **1a** (0.2 mmol, 52.8 mg) and (8*R*,9*S*,13*S*,14*S*)-2-allyl-13-methyl-6,7,8,9,11,12,13,14,15,16-decahydro-17*H*-cyclopenta[*a*]phenanthren-17-one (**2ab**) (0.3 mmol, 88.3 mg) at 30 Hz for 270 min to afford **46** (56.9 mg, 63% yield, l:b >99:1) as a pale-yellow oil. **^{1}H NMR (300 MHz, CDCl$_3$)** δ 10.10 (s, 1H), 8.33 – 8.22 (m, 1H), 7.34 – 7.24 (m, 3H), 7.22 (d, *J* = 8.0 Hz, 1H), 6.99 (dd, *J* = 8.0, 1.9 Hz, 1H), 6.92 (d, *J* = 1.9 Hz, 1H), 3.67 (s, 3H), 3.18 – 3.07 (m, 2H), 2.88 (dd, *J* = 8.7, 4.1 Hz, 2H), 2.71 (t, *J* = 7.6 Hz, 2H), 2.57 – 2.22 (m, 3H), 2.21 – 1.91 (m, 6H), 1.71 – 1.40 (m, 6H), 0.92 (s, 3H). **^{13}C NMR (75 MHz, CDCl$_3$)** δ 221.0 (C_q), 184.2 (CH), 151.5 (C_q), 138.3 (C_q), 137.8 (C_q), 137.2 (C_q), 136.7 (C_q), 129.1 (CH), 125.8 (CH), 125.8 (CH), 125.6 (CH), 123.3 (CH), 123.0 (CH), 121.1 (CH), 114.1 (C_q), 109.4 (CH), 50.6 (CH), 48.1 (C_q), 44.4 (CH), 38.3 (CH), 36.0 (CH_2), 34.9 (CH_2), 31.7 (CH_2), 31.4 (CH_2), 29.9 (CH_3), 29.5 (CH_2), 26.6 (CH_2), 25.9 (CH_2), 24.1 (CH_2), 21.7 (CH_2), 14.0 (CH_3). **HRMS** (ESI) m/z $(M+H)^+$: calculated for $(C_{31}H_{36}NO_2)^+$: 454.2741, found: 454.2743.

2-(2-((*R*)-2,8-dimethyl-2-((4*R*,8*R*)-4,8,12-trimethyltridecyl)chroman-6-yl)ethyl)-1-

methyl-1*H*-indole-3-carbaldehyde (47)

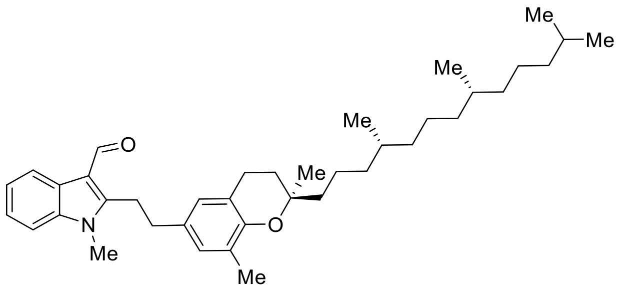

The **general procedure 4** was followed using indole substrate **1a** (0.2 mmol, 52.8 mg) and (*R*)-2,8-dimethyl-2-((4*R*,8*R*)-4,8,12-trimethyltridecyl)-6-vinylchromane (**2ac**) (0.3 mmol, 123.7 mg) at 30 Hz for 270 min to afford **47** (92.5 mg, 81% yield, l:b >99:1) as a pale-yellow oil. **^{1}H NMR (300 MHz, $CDCl_3$)** δ 10.17 (s, 1H), 8.34 – 8.23 (m, 1H), 7.34 – 7.27 (m, 3H), 6.76 (d, *J* = 2.2 Hz, 1H), 6.62 (d, *J* = 2.2 Hz, 1H), 3.55 (s, 3H), 3.33 (dd, *J* = 8.9, 6.6 Hz, 2H), 2.86 (dd, *J* = 8.9, 6.6 Hz, 2H), 2.65 (t, *J* = 6.7 Hz, 2H), 2.13 (s, 3H), 1.86 – 1.70 (m, 2H), 1.61 – 1.02 (m, 24H), 0.92 – 0.81 (m, 12H). **^{13}C NMR (75 MHz, $CDCl_3$)** δ 184.2 (CH), 151.3 (C_q), 151.0 (C_q), 137.1 (C_q), 130.1 (C_q), 128.4 (CH), 126.7 (CH), 126.6 (C_q), 125.9 (C_q), 123.2 (CH), 123.0 (CH), 121.0 (CH), 120.8 (C_q), 113.8 (C_q), 109.5 (CH), 76.1 (C_q), 40.2 (CH_2), 39.5 (CH_2), 37.6 (CH_2), 37.6 (CH_2), 37.4 (CH_2), 36.3 (CH_2), 32.9 (CH), 32.8 (CH), 31.4 (CH_2), 29.7 (CH_3), 28.1 (CH), 27.4 (CH_2), 24.9 (CH_2), 24.6 (CH_2), 24.3 (CH_3), 22.9 (CH_3), 22.8 (CH_3), 22.4 (CH_2), 21.1 (CH_2), 19.9 (CH_3), 19.8 (CH_3), 16.1 (CH_3). **HRMS** (ESI) m/z $(M+H)^+$: calculated for $(C_{39}H_{58}NO_2)^+$: 572.4462, found: 572.4462.

1-Methyl-2-(1-phenylethyl)-1*H*-indole-3-carbaldehyde (49)

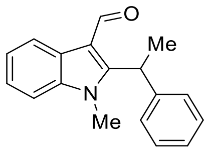

The **general procedure 5** was followed using indole substrate **1a** (0.2 mmol, 52.8 mg) and styrene (**48a**) (0.3 mmol, 34 μL) to afford **49** (45.9 mg, 87% yield, b:l >99:1) as a pale-yellow solid. **M.p.**: 94-96 °C. **^{1}H NMR (300 MHz, $CDCl_3$)** δ 10.25 (s, 1H), 8.47 – 8.25 (m, 1H), 7.37 – 7.16 (m, 8H), 5.20 (q, *J* = 7.4 Hz, 1H), 3.43 (s, 3H), 1.86 (d, *J* = 7.4 Hz, 3H). **^{13}C NMR (75 MHz, $CDCl_3$)** δ 184.9 (CH), 153.6 (C_q), 141.1 (C_q),

137.5 (C_q), 129.0 (CH), 127.1 (CH), 127.0 (CH), 125.9 (C_q), 123.6 (CH), 123.2 (CH), 121.5 (CH), 114.7 (C_q), 109.4 (CH), 34.6 (CH_3), 31.3 (CH), 18.7 (CH_3). **HRMS** (ESI) m/z $(M+H)^+$: calculated for $(C_{18}H_{18}NO)^+$: 264.1383, found: 264.1383. The analytical data are in accordance with those reported in the literature[1].

1-Methyl-2-(1-phenylethyl)-1*H*-pyrrolo[2,3-*b*]pyridine-3-carbaldehyde (50)

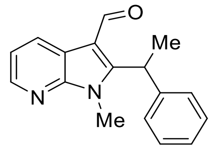

The **general procedure 5** was followed using indole substrate **1f** (0.2 mmol, 53.0 mg) and styrene (**48a**) (0.3 mmol, 34 µL) to afford **50** (45.4 mg, 86% yield, b:l >99:1) as a red oil. **^{1}H NMR (300 MHz, $CDCl_3$)** δ 10.24 (s, 1H), 8.62 (dd, *J* = 7.8, 1.6 Hz, 1H), 8.37 (dd, *J* = 4.8, 1.7 Hz, 1H), 7.39 – 7.32 (m, 2H), 7.31 – 7.22 (m, 4H), 5.18 (q, *J* = 7.4 Hz, 1H), 3.63 (s, 3H), 1.93 (d, *J* = 7.4 Hz, 3H). **^{13}C NMR (75 MHz, $CDCl_3$)** δ 184.9 (CH), 154.3 (C_q), 148.5 (C_q), 144.4 (CH), 140.7 (C_q), 130.0 (CH), 129.1 (CH), 127.3 (CH), 127.2 (CH), 119.2 (CH), 118.6 (C_q), 113.1 (C_q), 35.2 (CH_3), 29.7 (CH), 19.0 (CH_3). **HRMS** (ESI) m/z $(M+H)^+$: calculated for $(C_{17}H_{17}N_2O)^+$: 265.1335, found: 265.1329.

3-Formyl-1-methyl-2-(1-phenylethyl)-1*H*-indole-5-carbonitrile (51)

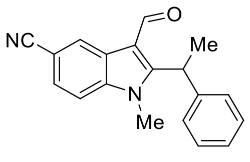

The **general procedure 5** was followed using indole substrate **1j** (0.2 mmol, 57.8 mg) and styrene (**48a**) (0.3 mmol, 34 µL) to afford **51** (40.7 mg, 71% yield, b:l >99:1) as a pale-yellow solid. **M.p.**: 130-132 °C. **^{1}H NMR (300 MHz, $CDCl_3$)** δ 10.25 (s, 1H), 8.74 (d, *J* = 1.0 Hz, 1H), 7.53 (dd, *J* = 8.5, 1.6 Hz, 1H), 7.39 – 7.28 (m, 4H), 7.28 – 7.20 (m, 2H), 5.17 (q, *J* = 7.4 Hz, 1H), 3.51 (s, 3H), 1.91 (d, *J* = 7.4 Hz, 3H). **^{13}C NMR (75 MHz, $CDCl_3$)** δ 184.8 (CH), 155.5 (C_q), 140.4 (C_q), 139.1 (C_q), 129.2 (CH), 127.4 (CH), 127.1 (CH), 127.0 (CH), 126.8 (CH), 125.7 (C_q), 120.1 (C_q), 114.9 (C_q),

110.4 (CH), 106.4 (C_q), 34.9 (CH_3), 31.6 (CH), 18.9 (CH_3). **HRMS** (ESI) m/z $(M+H)^+$: calculated for $(C_{19}H_{17}N_2O)^+$: 289.1335, found: 289.1329.

Methyl 3-formyl-1-methyl-2-(1-phenylethyl)-1*H*-indole-5-carboxylate (52)

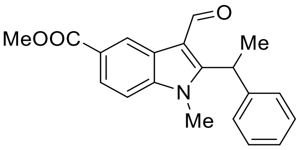

The **general procedure 5** was followed using indole substrate **1k** (0.2 mmol, 64.4 mg) and styrene (**48a**) (0.3 mmol, 34 µL) to afford **52** (48.8 mg, 76% yield, b:l >99:1) as a pale-yellow solid. **M.p.**: 154-155 °C. **^{1}H NMR (300 MHz, CDCl$_3$)** δ 10.30 (s, 1H), 9.05 (dd, *J* = 1.6, 0.6 Hz, 1H), 8.01 (dd, *J* = 8.6, 1.7 Hz, 1H), 7.48 – 7.16 (m, 6H), 5.24 (q, *J* = 7.4 Hz, 1H), 3.95 (s, 3H), 3.48 (s, 3H), 1.89 (d, *J* = 7.4 Hz, 3H). **^{13}C NMR (75 MHz, CDCl$_3$)** δ 184.8 (CH), 167.8 (C_q), 154.8 (C_q), 140.7 (C_q), 140.0 (C_q), 129.1 (CH), 127.2 (CH), 127.1 (CH), 125.5 (C_q), 125.1 (CH), 125.1 (C_q), 123.8 (CH), 115.3 (C_q), 109.3 (CH), 52.1 (CH_3), 34.7 (CH_3), 31.5 (CH), 18.7 (CH_3). **HRMS** (ESI) m/z $(M+H)^+$: calculated for $(C_{20}H_{20}NO_3)^+$: 322.1438, found: 322.1441.

5-Methoxy-1-methyl-2-(1-phenylethyl)-1*H*-indole-3-carbaldehyde (53)

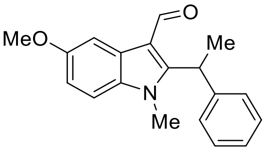

The **general procedure 5** was followed using indole substrate **1n** (0.2 mmol, 58.8 mg) and styrene (**48a**) (0.3 mmol, 34 µL) to afford **53** (37.7 mg, 64% yield, b:l >99:1) as a pale-yellow oil. **^{1}H NMR (300 MHz, CDCl$_3$)** δ 10.21 (s, 1H), 7.91 (d, *J* = 2.5 Hz, 1H), 7.40 – 7.29 (m, 2H), 7.28 – 7.22 (m, 3H), 7.16 (d, *J* = 8.9 Hz, 1H), 6.93 (dd, *J* = 8.9, 2.5 Hz, 1H), 5.13 (q, *J* = 7.4 Hz, 1H), 3.91 (s, 3H), 3.44 (s, 3H), 1.88 (d, *J* = 7.4 Hz, 3H). **^{13}C NMR (75 MHz, CDCl$_3$)** δ 184.8 (CH), 157.0 (C_q), 153.7 (C_q), 141.2 (C_q), 132.3 (C_q), 129.0 (CH), 127.1 (CH), 127.0 (C_q), 126.5 (C_q), 114.7 (CH), 113.8 (CH), 110.2 (CH), 103.2 (CH), 56.1 (CH_3), 34.7 (CH_3), 31.4 (CH), 18.9 (CH_3). **HRMS** (ESI) m/z $(M+H)^+$: calculated for $(C_{19}H_{20}NO_2)^+$: 294.1489, found: 294.1496.

6-Chloro-1-methyl-2-(1-phenylethyl)-1*H*-indole-3-carbaldehyde (54)

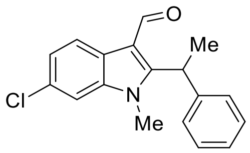

The **general procedure 5** was followed using indole substrate **1o** (0.2 mmol, 59.6 mg) and styrene (**48a**) (0.3 mmol, 34 μL) to afford **54** (43.2 mg, 73% yield, b:l >99:1) as a pale-yellow oil. **^{1}H NMR (300 MHz, CDCl$_3$)** δ 10.23 (s, 1H), 8.29 (d, *J* = 8.9 Hz, 1H), 7.46 – 7.17 (m, 7H), 5.17 (q, *J* = 7.4 Hz, 1H), 3.42 (s, 3H), 1.89 (d, *J* = 7.4 Hz, 3H). **^{13}C NMR (75 MHz, CDCl$_3$)** δ 184.8 (CH), 154.2 (C_q), 140.8 (C_q), 138.1 (C_q), 129.6 (C_q), 129.1 (CH), 127.2 (CH), 127.1 (CH), 124.3 (C_q), 123.7 (CH), 122.6 (CH), 114.7 (C_q), 109.7 (CH), 34.7 (CH_3), 31.4 (CH), 18.8 (CH_3). **HRMS** (ESI) m/z $(M+H)^+$: calculated for $(C_{18}H_{17}ClNO)^+$: 298.0993, found: 298.0990.

7-Ethyl-1-methyl-2-(1-phenylethyl)-1*H*-indole-3-carbaldehyde (55)

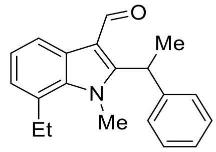

The **general procedure 5** was followed using indole substrate **1q** (0.2 mmol, 58.4 mg) and styrene (**48a**) (0.3 mmol, 34 μL) to afford **55** (39.0 mg, 67% yield, b:l >99:1) as a yellow oil. **^{1}H NMR (300 MHz, CDCl$_3$)** δ 10.28 (s, 1H), 8.30 (dd, *J* = 7.9, 1.3 Hz, 1H), 7.39 – 7.30 (m, 2H), 7.31 – 7.21 (m, 3H), 7.22 (d, *J* = 7.7 Hz, 1H), 7.08 (dd, *J* = 7.4, 1.4 Hz, 1H), 5.30 (q, *J* = 7.4 Hz, 1H), 3.68 (s, 3H), 3.06 (q, *J* = 7.5 Hz, 2H), 1.88 (d, *J* = 7.4 Hz, 3H), 1.31 (t, *J* = 7.5 Hz, 3H). **^{13}C NMR (75 MHz, CDCl$_3$)** δ 184.9 (CH), 153.9 (C_q), 141.3 (C_q), 135.6 (C_q), 129.0 (CH), 128.0 (C_q), 127.2 (C_q), 127.0 (CH), 126.9 (CH), 125.4 (CH), 123.2 (CH), 119.3 (CH), 114.7 (C_q), 34.5 (CH_3), 34.2 (CH), 26.0 (CH_2), 18.5 (CH_3), 16.9 (CH_3). **HRMS** (ESI) m/z $(M+H)^+$: calculated for $(C_{20}H_{22}NO)^+$: 292.1696, found: 292.1697.

2-(1-(4-Fluorophenyl)ethyl)-1-methyl-1*H*-indole-3-carbaldehyde (56)

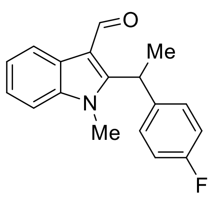

The **general procedure 5** was followed using indole substrate **1a** (0.2 mmol, 52.8 mg) and 1-fluoro-4-vinylbenzene (**48b**) (0.3 mmol, 36 μL) to afford **56** (45.4 mg, 81% yield, b:l >99:1) as an orange oil. **^{1}H NMR (300 MHz, CDCl$_3$)** δ 10.26 (s, 1H), 8.42 – 8.27 (m, 1H), 7.37 – 7.27 (m, 3H), 7.27 – 7.18 (m, 2H), 7.09 – 6.96 (m, 2H), 5.21 (q, *J* = 7.3 Hz, 1H), 3.46 (s, 3H), 1.87 (d, *J* = 7.4 Hz, 3H). **^{13}C NMR (75 MHz, CDCl$_3$)** δ 184.8 (CH), 161.8 (d, *J* = 246.2 Hz, C_q), 153.0 (C_q), 137.5 (C_q), 136.9 (d, *J* = 3.3 Hz, C_q), 128.7 (d, *J* = 8.0 Hz, CH), 126.0 (C_q), 123.7 (CH), 123.2 (CH), 121.4 (CH), 115.9 (d, *J* = 21.4 Hz, CH), 114.6 (C_q), 109.5 (CH), 34.0 (CH_3), 31.3 (CH), 18.9 (CH_3). **^{19}F NMR (282 MHz, CDCl$_3$)** δ -115.70. **HRMS** (ESI) m/z $(M+H)^+$: calculated for $(C_{18}H_{17}FNO)^+$: 282.1289, found: 282.1295.

2-(1-([1,1'-Biphenyl]-4-yl)ethyl)-1-methyl-1*H*-indole-3-carbaldehyde (57)

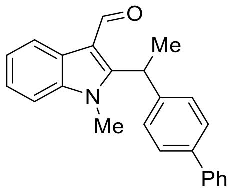

The **general procedure 5** was followed using indole substrate **1a** (0.2 mmol, 52.8 mg) and 4-vinyl-1,1'-biphenyl (**48c**) (0.3 mmol, 54.1 mg) to afford **57** (61.0 mg, 90% yield, b:l >99:1) as a yellow oil. **^{1}H NMR (300 MHz, CDCl$_3$)** δ 10.33 (s, 1H), 8.55 – 8.34 (m, 1H), 7.65 – 7.53 (m, 4H), 7.50 – 7.43 (m, 2H), 7.40 – 7.28 (m, 6H), 5.27 (q, *J* = 7.4 Hz, 1H), 3.52 (s, 3H), 1.93 (d, *J* = 7.4 Hz, 3H). **^{13}C NMR (75 MHz, CDCl$_3$)** δ 184.8 (CH), 153.4 (C_q), 140.4 (C_q), 140.2 (C_q), 139.8 (C_q), 137.5 (C_q), 128.9 (CH), 127.5 (CH), 127.5 (CH), 127.1 (CH), 125.9 (C_q), 123.6 (CH), 123.1 (CH), 121.5 (CH), 114.6 (C_q), 109.4 (CH), 34.3 (CH_3), 31.3 (CH), 18.8 (CH_3). **HRMS** (ESI) m/z $(M+H)^+$: calculated for $(C_{24}H_{22}NO)^+$: 340.1696, found: 340.1691.

2-(1-(4-Methoxyphenyl)ethyl)-1-methyl-1*H*-indole-3-carbaldehyde (58)

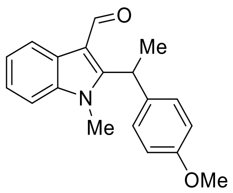

The **general procedure 5** was followed using indole substrate **1a** (0.2 mmol, 52.8 mg) and 1-methoxy-4-vinylbenzene (**48d**) (0.3 mmol, 40 μL) to afford **58** (51.8 mg, 88% yield, b:l >99:1) as a white solid. **M.p.**: 164-166 °C. **^{1}H NMR (300 MHz, CDCl$_3$)** δ 10.26 (s, 1H), 8.47 – 8.26 (m, 1H), 7.39 – 7.23 (m, 3H), 7.17 (d, *J* = 8.0 Hz, 2H), 6.86 (d, *J* = 8.8 Hz, 2H), 5.15 (q, *J* = 7.4 Hz, 1H), 3.79 (s, 3H), 3.47 (s, 3H), 1.86 (d, *J* = 7.4 Hz, 3H). **^{13}C NMR (75 MHz, CDCl$_3$)** δ 184.9 (CH), 158.5 (C_q), 154.0 (C_q), 137.5 (C_q), 133.1 (C_q), 128.2 (CH), 125.9 (C_q), 123.5 (CH), 123.1 (CH), 121.6 (CH), 114.6 (C_q), 114.3 (CH), 109.4 (CH), 55.4 (CH_3), 33.9 (CH_3), 31.2 (CH), 19.0 (CH_3). **HRMS** (ESI) m/z $(M+H)^+$: calculated for $(C_{19}H_{20}NO_2)^+$: 294.1489, found: 294.1499.

2-(1-(4-(Dimethylamino)phenyl)ethyl)-1-methyl-1*H*-indole-3-carbaldehyde (59)

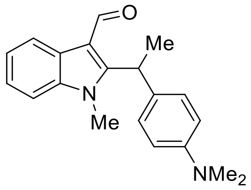

The **general procedure 5** was followed using indole substrate **1a** (0.2 mmol, 52.8 mg) and *N*,*N*-dimethyl-4-vinylaniline (**48e**) (0.3 mmol, 44.2 mg) to afford **59** (38.2 mg, 62% yield, b:l >99:1) as a colorless oil. **^{1}H NMR (300 MHz, CDCl$_3$)** δ 10.26 (s, 1H), 8.47 – 8.30 (m, 1H), 7.36 – 7.23 (m, 3H), 7.11 (d, *J* = 8.0 Hz, 2H), 6.68 (d, *J* = 8.8 Hz, 2H), 5.10 (q, *J* = 7.4 Hz, 1H), 3.49 (s, 3H), 2.93 (s, 6H), 1.84 (d, *J* = 7.4 Hz, 3H). **^{13}C NMR (75 MHz, CDCl$_3$)** δ 185.1 (CH), 154.8 (C_q), 149.5 (C_q), 137.6 (C_q), 128.6 (C_q), 127.8 (CH), 125.9 (C_q), 123.4 (CH), 123.1 (CH), 121.7 (CH), 114.7 (C_q), 112.9 (CH), 109.3 (CH), 40.7 (CH_3), 33.7 (CH_3), 31.2 (CH), 19.0 (CH_3). **HRMS** (ESI) m/z $(M+H)^+$: calculated for $(C_{20}H_{23}N_2O)^+$: 307.1805, found: 307.1811.

1-Methyl-2-(1-(*m*-tolyl)ethyl)-1*H*-indole-3-carbaldehyde (60)

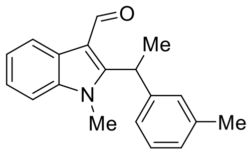

The **general procedure 5** was followed using indole substrate **1a** (0.2 mmol, 52.8 mg) and 1-methyl-3-vinylbenzene (**48f**) (0.3 mmol, 40 μL) to afford **60** (44.3 mg, 80% yield, b:l >99:1) as a yellow oil. **^{1}H NMR (300 MHz, CDCl$_3$)** δ 10.27 (s, 1H), 8.58 – 8.25 (m, 1H), 7.37 – 7.27 (m, 3H), 7.22 (d, *J* = 7.5 Hz, 1H), 7.07 (d, *J* = 8.3 Hz, 3H), 5.17 (q, *J* = 7.4 Hz, 1H), 3.48 (s, 3H), 2.31 (s, 3H), 1.87 (d, *J* = 7.4 Hz, 3H). **^{13}C NMR (75 MHz, CDCl$_3$)** δ 185.0 (CH), 153.9 (C_q), 141.0 (C_q), 138.7 (C_q), 137.5 (C_q), 128.9 (CH), 128.0 (CH), 127.8 (CH), 125.9 (C_q), 124.0 (CH), 123.5 (CH), 123.1 (CH), 121.6 (CH), 114.7 (C_q), 109.4 (CH), 34.5 (CH_3), 31.3 (CH), 21.6 (CH_3), 18.8 (CH_3). **HRMS** (ESI) m/z $(M+H)^+$: calculated for $(C_{19}H_{20}NO)^+$: 278.1539, found: 278.1545.

2-(1-(2-Methoxyphenyl)ethyl)-1-methyl-1*H*-indole-3-carbaldehyde (61)

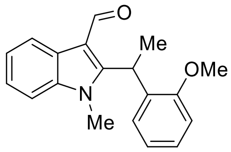

The **general procedure 5** was followed using indole substrate **1a** (0.2 mmol, 52.8 mg) and 1-methoxy-2-vinylbenzene (**48g**) (0.3 mmol, 40 μL) to afford **61** (46.2 mg, 79% yield, b:l >99:1) as a yellow solid. **M.p.**: 165-167 °C. **^{1}H NMR (300 MHz, CDCl$_3$)** δ 10.19 (s, 1H), 8.45 – 8.28 (m, 1H), 7.43 (d, *J* = 7.6 Hz, 1H), 7.31 – 7.21 (m, 4H), 7.00 (td, *J* = 7.5, 1.2 Hz, 1H), 6.81 (dd, *J* = 8.2, 1.2 Hz, 1H), 5.00 (q, *J* = 7.4 Hz, 1H), 3.71 (s, 3H), 3.63 (s, 3H), 1.84 (d, *J* = 7.4 Hz, 3H). **^{13}C NMR (75 MHz, CDCl$_3$)** δ 185.9 (CH), 157.2 (C_q), 154.9 (C_q), 137.0 (C_q), 130.0 (C_q), 128.6 (CH), 127.1 (CH), 126.0 (C_q), 123.1 (CH), 122.9 (CH), 122.1 (CH), 120.9 (CH), 114.1 (C_q), 110.8 (CH), 109.3 (CH), 55.5 (CH_3), 31.2 (CH_3), 30.5 (CH), 20.1 (CH_3). **HRMS** (ESI) m/z $(M+H)^+$: calculated for $(C_{19}H_{20}NO_2)^+$: 294.1489, found: 294.1497.

1-Methyl-2-(1-(naphthalen-2-yl)ethyl)-1*H*-indole-3-carbaldehyde (62)

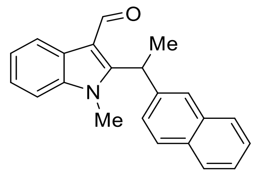

The **general procedure 5** was followed using indole substrate **1a** (0.2 mmol, 52.8 mg) and 2-vinylnaphthalene (**48h**) (0.3 mmol, 46.3 mg) to afford **62** (53.3 mg, 85% yield, b:l >99:1) as a colorless oil. **^{1}H NMR (300 MHz, CDCl$_3$)** δ 10.31 (s, 1H), 8.54 – 8.26 (m, 1H), 7.85 – 7.70 (m, 4H), 7.54 – 7.38 (m, 2H), 7.35 – 7.18 (m, 4H), 5.36 (q, *J* = 7.3 Hz, 1H), 3.44 (s, 3H), 1.97 (d, *J* = 7.3 Hz, 3H). **^{13}C NMR (75 MHz, CDCl$_3$)** δ 184.9 (CH), 153.3 (C_q), 138.6 (C_q), 137.5 (C_q), 133.5 (C_q), 132.4 (C_q), 128.8 (CH), 127.9 (CH), 127.8 (CH), 126.6 (CH), 126.2 (CH), 126.0 (C_q), 125.9 (CH), 125.1 (CH), 123.6 (CH), 123.2 (CH), 121.5 (CH), 114.8 (C_q), 109.5 (CH), 34.8 (CH_3), 31.3 (CH), 18.8 (CH_3). **HRMS** (ESI) m/z $(M+H)^+$: calculated for $(C_{22}H_{20}NO)^+$: 314.1539, found: 314.1540.

1-Methyl-2-(1-ferrocenylethyl)-1*H*-indole-3-carbaldehyde (63)

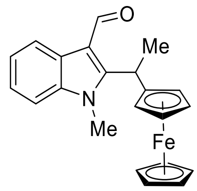

The **general procedure 5** was followed using indole substrate **1a** (0.2 mmol, 52.8 mg) and vinylferrocene (**48i**) (0.3 mmol, 63.6 mg) to afford **63** (44.5 mg, 60% yield, b:l >99:1) as a red solid. **M.p.**: 157-159 °C. **^{1}H NMR (300 MHz, CDCl$_3$)** δ 10.25 (s, 1H), 8.41 – 8.26 (m, 1H), 7.34 – 7.14 (m, 3H), 5.17 (q, *J* = 7.4 Hz, 1H), 4.44 (dt, *J* = 2.6, 1.4 Hz, 1H), 4.19 (s, 5H), 4.16 (td, *J* = 2.5, 1.3 Hz, 1H), 4.13 (td, *J* = 2.5, 1.3 Hz, 1H), 4.03 (dt, *J* = 2.6, 1.4 Hz, 1H), 3.55 (s, 3H), 1.77 (d, *J* = 7.4 Hz, 3H). **^{13}C NMR (75 MHz, CDCl$_3$)** δ 184.7 (CH), 154.1 (C_q), 137.4 (C_q), 125.9 (C_q), 123.4 (CH), 123.0 (CH), 121.4 (CH), 113.4 (C_q), 109.4 (CH), 89.7 (C_q), 69.2 (CH), 68.9 (CH), 68.8 (CH), 67.2 (CH), 66.9 (CH), 31.3 (CH_3), 31.1 (CH), 19.0 (CH_3). **HRMS** (ESI) m/z $(M+H)^+$: calculated for $(C_{22}H_{22}FeNO)^+$: 372.1045, found: 372.1041.

1-Methyl-2-phenethyl-1*H*-indole-3-carbaldehyde (64)

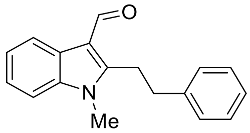

The **general procedure 6** was followed using indole substrate **1a** (0.2 mmol, 52.8 mg) and styrene (**48a**) (0.3 mmol, 34 μL) to afford **64** (38.1 mg, 72% yield, l:b = 94:6) as a pale-yellow oil. **^{1}H NMR (300 MHz, CDCl$_3$)** δ 10.06 (s, 1H), 8.20 (ddd, *J* = 5.4, 3.1, 1.3 Hz, 1H), 7.28 – 7.14 (m, 6H), 7.04 (dd, *J* = 7.6, 1.9 Hz, 2H), 3.41 (s, 3H), 3.33 (t, *J* = 7.5 Hz, 2H), 2.96 (t, *J* = 7.5 Hz, 2H). **^{13}C NMR (75 MHz, CDCl$_3$)** δ 184.1 (CH), 150.4 (C_q), 139.9 (C_q), 137.1 (C_q), 128.8 (CH), 128.6 (CH), 126.9 (CH), 126.0 (C_q), 123.3 (CH), 123.0 (CH), 120.8 (CH), 113.9 (C_q), 109.6 (CH), 36.8 (CH_2), 29.6 (CH_3), 27.1 (CH_2). **HRMS** (ESI) m/z $(M+H)^+$: calculated for $(C_{18}H_{18}NO)^+$: 264.1383, found: 264.1379. The analytical data are in accordance with those reported in the literature[11].

1-Methyl-2-phenethyl-1*H*-pyrrolo[2,3-*b*]pyridine-3-carbaldehyde (65)

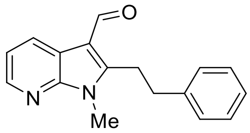

The **general procedure 6** was followed using indole substrate **1f** (0.2 mmol, 53.0 mg) and styrene (**48a**) (0.3 mmol, 34 μL) to afford **65** (42.8 mg, 81% yield, l:b = 92:8) as a pale-yellow oil. **^{1}H NMR (300 MHz, CDCl$_3$)** δ 10.06 (s, 1H), 8.51 (dd, *J* = 7.8, 1.6 Hz, 1H), 8.38 (dd, *J* = 4.8, 1.6 Hz, 1H), 7.34 – 7.20 (m, 4H), 7.13 (dd, *J* = 7.5, 1.9 Hz, 2H), 3.69 (s, 3H), 3.46 (t, *J* = 7.5 Hz, 2H), 3.08 (t, *J* = 7.5 Hz, 2H). **^{13}C NMR (75 MHz, CDCl$_3$)** δ 183.9 (CH), 151.0 (C_q), 148.3 (C_q), 144.1 (CH), 139.5 (C_q), 129.3 (CH), 128.9 (CH), 128.6 (CH), 127.1 (CH), 119.0 (CH), 118.5 (C_q), 112.5 (C_q), 36.6 (CH_2), 28.3 (CH_3), 27.3 (CH_2). **HRMS** (ESI) m/z $(M+H)^+$: calculated for $(C_{17}H_{17}N_2O)^+$: 265.1335, found: 265.1337.

3-Formyl-1-methyl-2-phenethyl-1*H*-indole-5-carbonitrile (66)

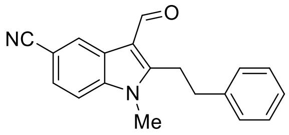

The **general procedure 6** was followed using indole substrate **1j** (0.2 mmol, 57.8 mg) and styrene (**48a**) (0.3 mmol, 34 µL) to afford **66** (36.6 mg, 64% yield, l:b = 95:5) as a pale-yellow solid. **M.p.**: 145-146 °C. **^{1}H NMR (300 MHz, $CDCl_3$)** δ 10.03 (s, 1H), 8.59 (dd, *J* = 1.6, 0.7 Hz, 1H), 7.50 (dd, *J* = 8.5, 1.6 Hz, 1H), 7.32 (dd, *J* = 8.5, 0.7 Hz, 1H), 7.29 – 7.22 (m, 3H), 7.08 – 7.02 (m, 2H), 3.49 (s, 3H), 3.41 (t, *J* = 7.3 Hz, 2H), 3.04 (t, *J* = 7.3 Hz, 2H). **^{13}C NMR (75 MHz, $CDCl_3$)** δ 183.9 (CH), 152.3 (C_q), 139.3 (C_q), 138.6 (C_q), 129.0 (CH), 128.6 (CH), 127.1 (CH), 126.5 (CH), 126.3 (CH), 125.6 (C_q), 120.1 (C_q), 114.2 (C_q), 110.5 (CH), 106.2 (C_q), 36.7 (CH_2), 29.9 (CH_3), 27.1 (CH_2). **HRMS** (ESI) m/z $(M+H)^+$: calculated for $(C_{19}H_{17}N_2O)^+$: 289.1335, found: 289.1342.

Methyl 3-formyl-1-methyl-2-phenethyl-1*H*-indole-5-carboxylate (67)

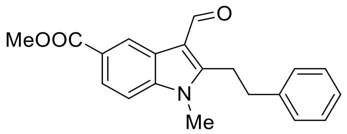

The **general procedure 6** was followed using indole substrate **1k** (0.2 mmol, 64.4 mg) and styrene (**48a**) (0.3 mmol, 34 µL) to afford **67** (45.3 mg, 71% yield, l:b = 94:6) as a white solid. **M.p.**: 146-149 °C. **^{1}H NMR (300 MHz, $CDCl_3$)** δ 10.12 (s, 1H), 8.94 (dd, *J* = 1.7, 0.7 Hz, 1H), 8.01 (dd, *J* = 8.7, 1.7 Hz, 1H), 7.34 – 7.20 (m, 4H), 7.14 – 7.00 (m, 2H), 3.96 (s, 3H), 3.49 (s, 3H), 3.40 (t, *J* = 7.4 Hz, 2H), 3.03 (t, *J* = 7.4 Hz, 2H). **^{13}C NMR (75 MHz, $CDCl_3$)** δ 184.0 (CH), 167.8 (C_q), 151.6 (C_q), 139.6 (C_q), 139.5 (C_q), 128.9 (CH), 128.6 (CH), 126.9 (CH), 125.5 (C_q), 124.9 (C_q), 124.8 (CH), 123.1 (CH), 114.5 (C_q), 109.4 (CH), 52.1 (CH_3), 36.6 (CH_2), 29.8 (CH_3), 27.2 (CH_2). **HRMS** (ESI) m/z $(M+H)^+$: calculated for $(C_{20}H_{20}NO_3)^+$: 322.1438, found: 322.1438.

5-Methoxy-1-methyl-2-phenethyl-1*H*-indole-3-carbaldehyde (68)

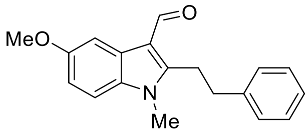

The **general procedure 6** was followed using indole substrate **1n** (0.2 mmol, 58.8 mg) and styrene (**48a**) (0.3 mmol, 34 μL) to afford **68** (40.5 mg, 69% yield, l:b = 97:3) as a pale-yellow oil. **^{1}H NMR (300 MHz, CDCl$_3$)** δ 10.06 (s, 1H), 7.78 (d, *J* = 2.5 Hz, 1H), 7.30 – 7.20 (m, 3H), 7.16 (d, *J* = 8.9 Hz, 1H), 7.10 (d, *J* = 2.2 Hz, 1H), 7.07 (d, *J* = 1.6 Hz, 1H), 6.91 (dd, *J* = 8.9, 2.5 Hz, 1H), 3.90 (s, 3H), 3.43 (s, 3H), 3.35 (t, *J* = 7.5 Hz, 2H), 3.01 (t, *J* = 7.5 Hz, 2H). **^{13}C NMR (75 MHz, CDCl$_3$)** δ 184.0 (CH), 156.8 (C_q), 150.5 (C_q), 139.9 (C_q), 132.0 (C_q), 128.9 (CH), 128.6 (CH), 126.9 (CH), 126.6 (C_q), 113.9 (C_q), 113.3 (CH), 110.4 (CH), 102.8 (CH), 56.0 (CH_3), 37.0 (CH_2), 29.7 (CH_3), 27.1 (CH_2). **HRMS** (ESI) m/z $(M+H)^+$: calculated for $(C_{19}H_{20}NO_2)^+$: 294.1489, found: 294.1491.

6-Chloro-1-methyl-2-phenethyl-1*H*-indole-3-carbaldehyde (69)

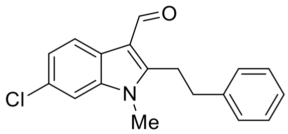

The **general procedure 6** was followed using indole substrate **1o** (0.2 mmol, 59.6 mg) and styrene (**48a**) (0.3 mmol, 34 μL) to afford **69** (37.5 mg, 63% yield, l:b = 94:6) as a pale-yellow oil. **^{1}H NMR (300 MHz, CDCl$_3$)** δ 10.05 (s, 1H), 8.16 (d, *J* = 9.0 Hz, 1H), 7.32 – 7.18 (m, 5H), 7.10 – 7.00 (m, 2H), 3.41 (s, 3H), 3.37 (t, *J* = 7.4 Hz, 2H), 3.01 (t, *J* = 7.4 Hz, 2H). **^{13}C NMR (75 MHz, CDCl$_3$)** δ 184.0 (CH), 151.0 (C_q), 139.6 (C_q), 137.6 (C_q), 129.3 (C_q), 128.9 (CH), 128.6 (CH), 127.0 (CH), 124.4 (C_q), 123.5 (CH), 122.0 (CH), 114.0 (C_q), 109.9 (CH), 36.9 (CH_2), 29.7 (CH_3), 27.1 (CH_2). **HRMS** (ESI) m/z $(M+H)^+$: calculated for $(C_{18}H_{17}ClNO)^+$: 298.0993, found: 298.0988.

7-Ethyl-1-methyl-2-phenethyl-1*H*-indole-3-carbaldehyde (70)

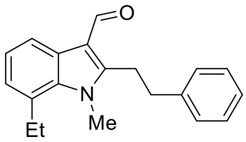

The **general procedure 6** was followed using indole substrate **1q** (0.2 mmol, 58.4 mg) and styrene (**48a**) (0.3 mmol, 34 µL) to afford **70** (28.2 mg, 48% yield, l:b = 90:10) as a red oil. **^{1}H NMR (300 MHz, CDCl$_3$)** δ 10.14 (s, 1H), 8.17 (dd, *J* = 7.8, 1.3 Hz, 1H), 7.32 – 7.24 (m, 3H), 7.20 (t, *J* = 7.6 Hz, 1H), 7.12 (dd, *J* = 7.6, 1.6 Hz, 2H), 7.06 (dd, *J* = 7.4, 1.2 Hz, 1H), 3.73 (s, 3H), 3.40 (t, *J* = 7.6 Hz, 2H), 3.08 (q, *J* = 7.5 Hz, 2H), 3.00 (t, *J* = 7.6 Hz, 2H), 1.32 (t, *J* = 7.5 Hz, 3H). **^{13}C NMR (75 MHz, CDCl$_3$)** δ 184.3 (CH), 150.8 (C_q), 140.0 (C_q), 135.2 (C_q), 128.9 (CH), 128.7 (CH), 128.0 (C_q), 127.2 (C_q), 126.9 (CH), 125.0 (CH), 123.1 (CH), 118.7 (CH), 113.8 (C_q), 36.8 (CH_2), 32.6 (CH_3), 27.0 (CH_2), 26.1 (CH_2), 17.0 (CH_3). **HRMS** (ESI) m/z $(M+H)^+$: calculated for $(C_{20}H_{22}NO)^+$: 292.1696, found: 292.1694.

2-(4-Fluorophenethyl)-1-methyl-1*H*-indole-3-carbaldehyde (71)

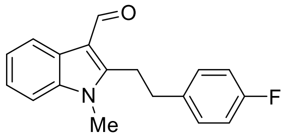

The **general procedure 6** was followed using indole substrate **1a** (0.2 mmol, 52.8 mg) and 1-fluoro-4-vinylbenzene (**48b**) (0.3 mmol, 36 µL) to afford **71** (41.3 mg, 73% yield, l:b = 85:15) as a colorless oil. **^{1}H NMR (300 MHz, CDCl$_3$)** δ 10.13 (s, 1H), 8.29 – 8.14 (m, 1H), 7.37 – 7.27 (m, 3H), 7.07 – 7.01 (m, 2H), 6.99 – 6.90 (m, 2H), 3.49 (s, 3H), 3.38 (t, *J* = 7.4 Hz, 2H), 2.99 (t, *J* = 7.5 Hz, 2H). **^{13}C NMR (75 MHz, CDCl$_3$)** δ 184.1 (CH), 161.9 (d, *J* = 245.0 Hz, C_q), 149.9 (C_q), 137.0 (C_q), 135.6 (d, *J* = 3.4 Hz, C_q), 130.1 (d, *J* = 7.9 Hz, CH), 126.1 (C_q), 123.3 (CH), 123.1 (CH), 120.7 (CH), 115.7 (d, *J* = 21.3 Hz, CH), 113.9 (C_q), 109.7 (CH), 35.9 (CH_2), 29.7 (CH_3), 27.3 (CH_2). **^{19}F NMR (282 MHz, CDCl$_3$)** δ -116.05. **HRMS** (ESI) m/z $(M+H)^+$: calculated for $(C_{18}H_{17}FNO)^+$: 282.1289, found: 282.1285.

2-(2-([1,1'-Biphenyl]-4-yl)ethyl)-1-methyl-1*H*-indole-3-carbaldehyde (72)

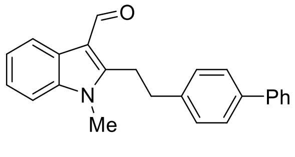

The **general procedure 6** was followed using indole substrate **1a** (0.2 mmol, 52.8 mg) and 4-vinyl-1,1'-biphenyl (**48c**) (0.3 mmol, 54.1 mg) to afford **72** (42.1 mg, 62% yield, l:b = 92:8) as a yellow oil. **^{1}H NMR (300 MHz, $CDCl_3$)** δ 10.16 (s, 1H), 8.29 – 8.17 (m, 1H), 7.58 – 7.52 (m, 2H), 7.48 (d, *J* = 8.1 Hz, 2H), 7.44 – 7.36 (m, 2H), 7.35 – 7.26 (m, 3H), 7.22 (s, 1H), 7.15 (d, *J* = 8.2 Hz, 2H), 3.47 (s, 3H), 3.39 (t, *J* = 7.6 Hz, 2H), 3.02 (t, *J* = 7.6 Hz, 2H). **^{13}C NMR (75 MHz, $CDCl_3$)** δ 184.1 (CH), 150.3 (C_q), 140.8 (C_q), 139.8 (C_q), 139.0 (C_q), 137.1 (C_q), 129.0 (CH), 128.9 (CH), 127.5 (CH), 127.4 (CH), 127.1 (CH), 126.1 (C_q), 123.3 (CH), 123.0 (CH), 120.8 (CH), 113.9 (C_q), 109.7 (CH), 36.5 (CH_2), 29.7 (CH_3), 27.1 (CH_2). **HRMS** (ESI) m/z $(M+H)^+$: calculated for $(C_{24}H_{22}NO)^+$: 340.1696, found: 340.1703.

2-(4-Methoxyphenethyl)-1-methyl-1*H*-indole-3-carbaldehyde (73)

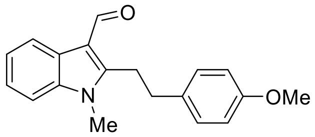

The **general procedure 6** was followed using indole substrate **1a** (0.2 mmol, 52.8 mg) and 1-methoxy-4-vinylbenzene (**48d**) (0.3 mmol, 40 μL) to afford **73** (50.2 mg, 86% yield, l:b = 98:2) as a colorless oil. **^{1}H NMR (300 MHz, $CDCl_3$)** δ 10.12 (s, 1H), 8.32 – 8.17 (m, 1H), 7.35 – 7.24 (m, 3H), 7.00 (d, *J* = 8.6 Hz, 2H), 6.79 (d, *J* = 8.6 Hz, 2H), 3.78 (s, 3H), 3.47 (s, 3H), 3.35 (t, *J* = 7.4 Hz, 2H), 2.96 (t, *J* = 7.4 Hz, 2H). **^{13}C NMR (75 MHz, $CDCl_3$)** δ 184.2 (CH), 158.5 (C_q), 150.6 (C_q), 137.1 (C_q), 131.9 (C_q), 129.6 (CH), 126.0 (C_q), 123.2 (CH), 123.0 (CH), 120.9 (CH), 114.2 (CH), 113.9 (C_q), 109.6 (CH), 55.4 (CH_3), 36.0 (CH_2), 29.7 (CH_3), 27.3 (CH_2). **HRMS** (ESI) m/z $(M+H)^+$: calculated for $(C_{19}H_{20}NO_2)^+$: 294.1489, found: 294.1486.

2-(4-(Dimethylamino)phenethyl)-1-methyl-1*H*-indole-3-carbaldehyde (74)

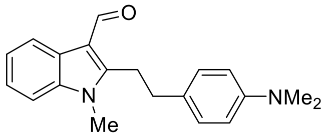

The **general procedure 6** was followed using indole substrate **1a** (0.2 mmol, 52.8 mg)

and *N*,*N*-dimethyl-4-vinylaniline (**48e**) (0.3 mmol, 44.2 mg) to afford **74** (31.5 mg, 51% yield, l:b = 94:6) as a yellow solid. **M.p.**: 92-93 °C. **^{1}H NMR (300 MHz, CDCl$_3$)** δ 10.14 (s, 1H), 8.41 – 8.16 (m, 1H), 7.38 – 7.23 (m, 3H), 6.96 (d, *J* = 8.6 Hz, 2H), 6.64 (d, *J* = 8.7 Hz, 2H), 3.49 (s, 3H), 3.34 (t, *J* = 7.5 Hz, 2H), 2.93 (t, *J* = 7.4 Hz, 2H), 2.91 (s, 6H). **^{13}C NMR (75 MHz, CDCl$_3$)** δ 184.3 (CH), 151.2 (C$_q$), 149.7 (C$_q$), 137.1 (C$_q$), 129.2 (CH), 127.8 (C$_q$), 126.0 (C$_q$), 123.2 (CH), 123.0 (CH), 121.0 (CH), 113.9 (C$_q$), 113.1 (CH), 109.6 (CH), 40.9 (CH$_3$), 36.1 (CH$_2$), 29.7 (CH$_3$), 27.5 (CH$_2$). **HRMS** (ESI) m/z (M+H)$^+$: calculated for $(C_{20}H_{23}N_2O)^+$: 307.1805, found: 307.1809.

1-Methyl-2-(3-methylphenethyl)-1*H*-indole-3-carbaldehyde (75)

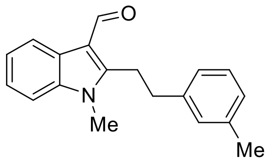

The **general procedure 6** was followed using indole substrate **1a** (0.2 mmol, 52.8 mg) and 1-methyl-3-vinylbenzene (**48f**) (0.3 mmol, 40 µL) to afford **75** (44.8 mg, 81% yield, l:b = 95:5) as a yellow solid. **M.p.**: 81-83 °C. **^{1}H NMR (300 MHz, CDCl$_3$)** δ 10.14 (s, 1H), 8.38 – 8.19 (m, 1H), 7.35 – 7.24 (m, 3H), 7.17 (t, *J* = 7.5 Hz, 1H), 7.05 (d, *J* = 7.6 Hz, 1H), 6.94 (s, 1H), 6.91 (d, *J* = 7.8 Hz, 1H), 3.51 (s, 3H), 3.37 (dd, *J* = 8.4, 7.0 Hz, 2H), 2.96 (dd, *J* = 8.5, 6.8 Hz, 2H), 2.29 (s, 3H). **^{13}C NMR (75 MHz, CDCl$_3$)** δ 184.2 (CH), 150.6 (C$_q$), 139.9 (C$_q$), 138.5 (C$_q$), 137.1 (C$_q$), 129.3 (CH), 128.7 (CH), 127.6 (CH), 126.0 (C$_q$), 125.6 (CH), 123.3 (CH), 123.0 (CH), 120.9 (CH), 113.9 (C$_q$), 109.6 (CH), 36.8 (CH$_2$), 29.6 (CH$_3$), 27.1 (CH$_2$), 21.4 (CH$_3$). **HRMS** (ESI) m/z (M+H)$^+$: calculated for $(C_{19}H_{20}NO)^+$: 278.1539, found: 278.1548.

2-(2-Methoxyphenethyl)-1-methyl-1*H*-indole-3-carbaldehyde (76)

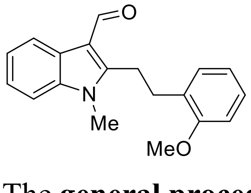

The **general procedure 6** was followed using indole substrate **1a** (0.2 mmol, 52.8 mg) and 1-methoxy-2-vinylbenzene (**48g**) (0.3 mmol, 40 µL) to afford **76** (42.7 mg,

73% yield, l:b = 96:4) as a yellow solid. **M.p.**: 133-136 °C. **^{1}H NMR (300 MHz, CDCl$_3$)** δ 10.06 (s, 1H), 8.36 – 8.22 (m, 1H), 7.34 – 7.23 (m, 3H), 7.29 – 7.17 (m, 1H), 7.04 (dd, *J* = 7.6, 1.6 Hz, 1H), 6.92 – 6.81 (m, 2H), 3.76 (s, 3H), 3.67 (s, 3H), 3.35 (dd, *J* = 8.6, 6.8 Hz, 2H), 2.99 (dd, *J* = 8.7, 6.7 Hz, 2H). **^{13}C NMR (75 MHz, CDCl$_3$)** δ 184.3 (CH), 157.5 (C_q), 151.7 (C_q), 137.3 (C_q), 130.3 (CH), 128.3 (CH), 128.2 (C_q), 125.8 (C_q), 123.2 (CH), 122.9 (CH), 121.3 (CH), 120.9 (CH), 114.2 (C_q), 110.4 (CH), 109.4 (CH), 55.4 (CH_3), 31.9 (CH_2), 29.6 (CH_3), 25.1 (CH_2). **HRMS** (ESI) m/z $(M+H)^+$: calculated for $(C_{19}H_{20}NO_2)^+$: 294.1489, found: 294.1491.

1-Methyl-2-(2-(naphthalen-2-yl)ethyl)-1*H*-indole-3-carbaldehyde (77)

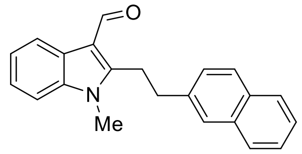

The **general procedure 6** was followed using indole substrate **1a** (0.2 mmol, 52.8 mg) and 2-vinylnaphthalene (**48h**) (0.3 mmol, 46.3 mg) to afford **77** (45.0 mg, 72% yield, l:b = 89:11) as a white solid. **M.p.**: 104-105 °C. **^{1}H NMR (300 MHz, CDCl$_3$)** δ 10.22 (s, 1H), 8.33 – 8.20 (m, 1H), 7.86 – 7.69 (m, 3H), 7.59 (s, 1H), 7.52 – 7.39 (m, 2H), 7.38 – 7.19 (m, 4H), 3.48 (dd, *J* = 8.4, 6.6 Hz, 2H), 3.48 (s, 3H), 3.17 (dd, *J* = 8.5, 6.8 Hz, 2H). **^{13}C NMR (75 MHz, CDCl$_3$)** δ 184.1 (CH), 150.4 (C_q), 137.5 (C_q), 137.1 (C_q), 133.7 (C_q), 132.4 (C_q), 128.5 (CH), 127.8 (CH), 127.6 (CH), 127.0 (CH), 126.9 (CH), 126.4 (CH), 126.1 (C_q), 125.8 (CH), 123.3 (CH), 123.0 (CH), 120.8 (CH), 113.8 (C_q), 109.7 (CH), 37.0 (CH_2), 29.7 (CH_3), 27.1 (CH_2). **HRMS** (ESI) m/z $(M+H)^+$: calculated for $(C_{22}H_{20}NO)^+$: 314.1539, found: 314.1540.

1-Methyl-2-ferrocenylethyl-1*H*-indole-3-carbaldehyde (78)

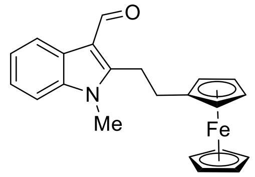

The **general procedure 6** was followed using indole substrate **1a** (0.2 mmol, 52.8 mg) and vinylferrocene (**48i**) (0.3 mmol, 63.6 mg) to afford **78** (49.7 mg, 67% yield, l:b =

95:5) as a red solid. **M.p.**: 127-129 °C. **^{1}H NMR (300 MHz, $CDCl_3$)** δ 10.14 (s, 1H), 8.34 – 8.15 (m, 1H), 7.34 – 7.22 (m, 3H), 4.10 (s, 5H), 4.05 (t, J = 1.8 Hz, 2H), 3.94 (t, J = 1.8 Hz, 2H), 3.48 (s, 3H), 3.25 (t, J = 7.4 Hz, 2H), 2.76 (t, J = 7.4 Hz, 2H). **^{13}C NMR (75 MHz, $CDCl_3$)** δ 184.2 (CH), 151.0 (C_q), 137.1 (C_q), 126.0 (C_q), 123.2 (CH), 123.0 (CH), 120.9 (CH), 113.7 (C_q), 109.6 (CH), 86.5 (C_q), 68.7 (CH), 68.4 (CH), 68.0 (CH), 31.2 (CH_2), 29.9 (CH_3), 27.0 (CH_2). **HRMS** (ESI) m/z $(M+H)^+$: calculated for $(C_{22}H_{22}FeNO)^+$: 372.1045, found: 372.1047.

6. Scale-up reaction

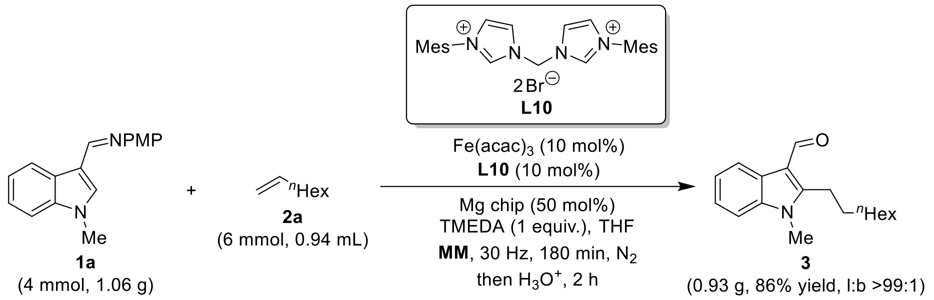

In the glove box, a mixture of indole substrate **1a** (4 mmol, 1.06 g), 1-octene (**2a**) (6 mmol, 0.94 mL), $Fe(acac)_3$ (10 mol%, 0.4 mmol, 141.3 mg), **L10** (10 mol%, 0.4 mmol, 217.6 mg), magnesium chip (50 mol%, 2 mmol, 48.0 mg), TMEDA (4 mmol, 0.6 mL) and tetrahydrofuran (1.0 mL) were placed in a nitrogen-purged zirconia vessel (25 mL) with zirconia ball (d_{MB} = 15 mm). Then, the vessel was sealed and milled in a mixer mill (RETSCH MM 400) at 30 Hz for 180 min under nitrogen atmosphere. Then, the reaction mixture was diluted with tetrahydrofuran (10 mL) and quenched with HCl aqueous solution (3 M, 10 mL). The resulting mixture was stirred at room temperature for 2 hours. The phases were then separated, the aqueous layer was extracted with ethyl acetate (50 mL ×3). The combined organic layer was washed with saturated $NaHCO_3$ solution and brine, dried over Na_2SO_4, filtered and concentrated *in vacuo*. The linear and branched ratio was determined by ^1H NMR analysis of the crude reaction mixture. The residue was purified by column chromatography on silica gel (*n*-hexane: ethyl acetate = 10:1) to afford the desired product **3** (931.7 mg, 86% yield, l:b >99:1).

7. Late-stage transformations

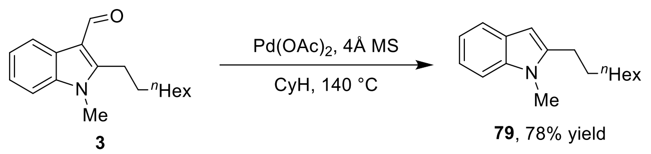

To a solution of **3** (0.1 mmol, 27.1 mg) and 4Å molecular sieves (50 mg) in anhydrous cyclohexane (0.5 mL), was added Pd(OAc)$_2$ (15 mol%, 0.015 mmol, 3.4 mg). After stirring at 140 °C for 24 hours, the reaction mixture was cooled to room temperature, filtered through a pad of Celite and washed with ethyl acetate (10 mL). The filtrate was concentrated and the crude product was purified by column chromatography on silica gel (*n*-hexane: ethyl acetate = 50:1) to afford the desired product **79** (19.0 mg, 78% yield).

1-Methyl-2-octyl-1*H*-indole (79)

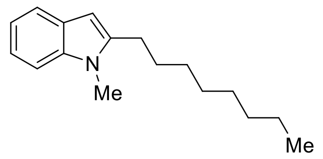

Pale-yellow oil. **^{1}H NMR (300 MHz, CDCl$_3$)** δ 7.53 (dt, *J* = 7.5, 1.0 Hz, 1H), 7.27 (dd, *J* = 8.0, 1.1 Hz, 1H), 7.15 (ddd, *J* = 8.2, 7.0, 1.3 Hz, 1H), 7.06 (ddd, *J* = 8.1, 7.0, 1.2 Hz, 1H), 6.25 (d, *J* = 0.9 Hz, 1H), 3.67 (s, 3H), 2.80 – 2.63 (m, 2H), 1.80 – 1.63 (m, 2H), 1.51 – 1.38 (m, 2H), 1.38 – 1.24 (m, 8H), 0.95 – 0.83 (m, 3H). **^{13}C NMR (75 MHz, CDCl$_3$)** δ 141.7 (C$_q$), 137.5 (C$_q$), 128.1 (C$_q$), 120.6 (CH), 119.8 (CH), 119.3 (CH), 108.8 (CH), 98.8 (CH), 32.0 (CH$_2$), 29.6 (CH$_2$), 29.6 (CH$_2$), 29.6 (CH$_3$), 29.4 (CH$_2$), 28.8 (CH$_2$), 27.0 (CH$_2$), 22.8 (CH$_2$), 14.3 (CH$_3$). **HRMS** (ESI) m/z (M+H)$^+$: calculated for (C$_{17}$H$_{26}$N)$^+$: 244.2060, found: 244.2063.

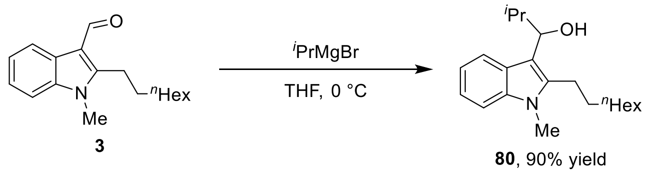

To a solution of **3** (0.1 mmol, 27.1 mg) in anhydrous THF (2 mL), was added the isopropyl magnesium bromide (3 M in Et$_2$O, 0.15 mmol, 50 μL) dropwise at 0 °C. After

stirring at 0 °C for 3 hours, the reaction mixture was quenched by a saturated solution of NH_4Cl (1 mL). The phases were separated and the aqueous layer was extracted with diethyl ether (3 × 5 mL). The combined organic layer was washed with brine, dried over Na_2SO_4, filtered and concentrated *in vacuo*. The crude product was purified by column chromatography on silica gel (*n*-hexane: ethyl acetate = 10:1) to afford the desired product **80** (28.3 mg, 90% yield).

2-Methyl-1-(1-methyl-2-octyl-1*H*-indol-3-yl)propan-1-ol (80)

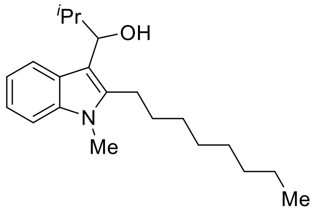

Pale-yellow oil. **^{1}H NMR (300 MHz, $CDCl_3$)** δ 7.79 (dt, *J* = 7.8, 1.0 Hz, 1H), 7.28 (dt, *J* = 8.2, 1.0 Hz, 1H), 7.18 (ddd, *J* = 8.2, 6.9, 1.2 Hz, 1H), 7.09 (ddd, *J* = 8.0, 6.9, 1.2 Hz, 1H), 4.51 (dd, *J* = 9.3, 2.0 Hz, 1H), 3.68 (s, 3H), 2.86 – 2.73 (m, 2H), 2.54 – 2.34 (m, 1H), 1.74 (br, 1H), 1.59 (dt, *J* = 15.2, 7.6 Hz, 2H), 1.50 – 1.28 (m, 10H), 1.23 (d, *J* = 6.4 Hz, 3H), 0.94 – 0.87 (m, 3H), 0.73 (d, *J* = 6.7 Hz, 3H). **^{13}C NMR (75 MHz, $CDCl_3$)** δ 138.8 (C_q), 137.2 (C_q), 125.8 (C_q), 121.0 (CH), 120.1 (CH), 119.2 (CH), 113.2 (C_q), 108.9 (CH), 75.2 (CH), 34.5 (CH), 32.0 (CH_2), 30.6 (CH_2), 29.9 (CH_2), 29.7 (CH_3), 29.5 (CH_2), 29.4 (CH_2), 24.9 (CH_2), 22.8 (CH_2), 20.3 (CH_3), 20.1 (CH_3), 14.2 (CH_3). **HRMS** (ESI) m/z $(M+H)^+$: calculated for $(C_{21}H_{34}NO)^+$: 316.2635, found: 316.2635.

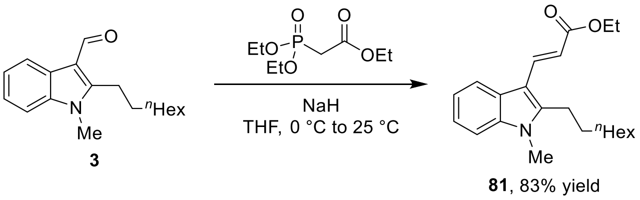

To a solution of ethyl 2-(diethoxyphosphoryl)acetate (0.2 mmol, 44.8 mg) in anhydrous THF (1 mL), was added portionwise NaH (60% dispersion in mineral oil, 0.4 mmol, 16.0 mg) at 0 °C. After stirring for 1 hour, **3** (0.1 mmol, 27.1 mg) was added and the resulting mixture was allowed to warm up to 25 °C and stirred overnight. The

reaction mixture was quenched by saturated NH_4Cl aqueous solution (1 mL). Then, diethyl ether (5 mL) and water (5 mL) were added. The phases were separated and the aqueous layer was extracted with diethyl ether (3 × 5 mL). The combined organic layer was washed with brine, dried over Na_2SO_4, filtered and concentrated *in vacuo*. The crude product was purified by column chromatography on silica gel (*n*-hexane: ethyl acetate = 10:1) to afford the desired product **81** (28.4 mg, 83% yield).

ethyl (*E*)-3-(1-methyl-2-octyl-1*H*-indol-3-yl)acrylate (81)

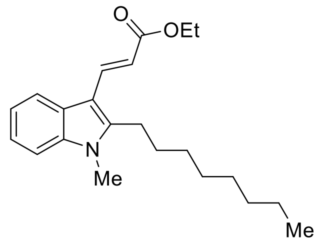

Colorless oil. **^{1}H NMR (300 MHz, $CDCl_3$)** δ 7.96 (d, J = 15.8 Hz, 1H), 7.96 – 7.85 (m, 1H), 7.36 – 7.17 (m, 3H), 6.44 (d, J = 15.8 Hz, 1H), 4.28 (q, J = 7.1 Hz, 2H), 3.71 (s, 3H), 3.03 – 2.82 (m, 2H), 1.66 – 1.55 (m, 2H), 1.47 – 1.23 (m, 13H), 0.92 – 0.83 (m, 3H). **^{13}C NMR (75 MHz, $CDCl_3$)** δ 169.0 (C_q), 146.3 (C_q), 137.8 (CH), 125.9 (C_q), 122.3 (CH), 121.5 (CH), 120.4 (CH), 111.7 (CH), 109.6 (CH), 108.9 (C_q), 60.1 (CH_2), 32.0 (CH_2), 30.3 (CH_2), 30.0 (CH_3), 29.6 (CH_2), 29.5 (CH_2), 29.3 (CH_2), 24.8 (CH_2), 22.8 (CH_2), 14.7 (CH_3), 14.2 (CH_3). **HRMS** (ESI) m/z $(M+H)^+$: calculated for $(C_{22}H_{32}NO_2)^+$: 342.2428, found: 342.2431.

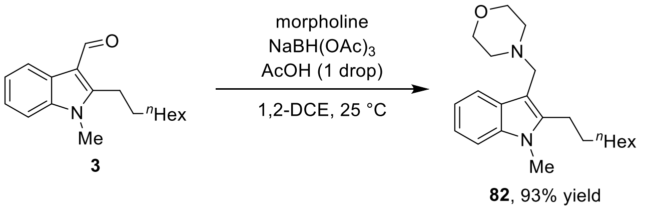

A solution of **3** (0.1 mmol, 27.1 mg), morpholine (0.2 mmol, 18 μL), $NaBH(OAc)_3$ (0.4 mmol, 84.8 mg) and AcOH (1 drop) in 1,2-dichloroethane (1 mL) was stirred at 25 °C for 12 hours. The reaction mixture was diluted with CH_2Cl_2 (5 mL), quenched by saturated $NaHCO_3$ aqueous solution (5 mL). The phases were separated and the aqueous layer was extracted with dichloromethane (3 × 5 mL). The combined organic

layer was washed with brine, dried over Na_2SO_4, filtered and concentrated *in vacuo*. The crude product was purified by column chromatography on silica gel (*n*-hexane: ethyl acetate = 10:1) to afford the desired product **82** (31.9 mg, 93% yield).

4-((1-Methyl-2-octyl-1*H*-indol-3-yl)methyl)morpholine (82)

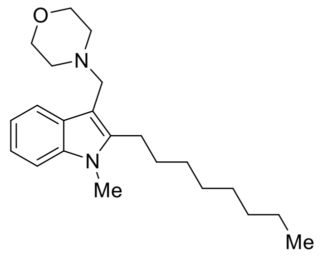

Pale-yellow oil. **^{1}H NMR (300 MHz, $CDCl_3$)** δ 7.71 (dq, *J* = 7.6, 0.8 Hz, 1H), 7.32 – 7.22 (m, 1H), 7.18 (ddd, *J* = 8.1, 6.9, 1.3 Hz, 1H), 7.10 (ddd, *J* = 8.1, 6.9, 1.2 Hz, 1H), 3.74 – 3.60 (m, 9H), 2.85 – 2.75 (m, 2H), 2.53 – 2.43 (m, 4H), 1.65 – 1.52 (m, 2H), 1.49 – 1.24 (m, 10H), 0.98 – 0.84 (m, 3H). **^{13}C NMR (75 MHz, $CDCl_3$)** δ 139.9 (C_q), 136.7 (C_q), 128.6 (C_q), 120.7 (CH), 119.1 (CH), 119.0 (CH), 108.7 (CH), 107.1 (C_q), 67.4 (CH_2), 53.8 (CH_2), 53.4 (CH_2), 32.0 (CH_2), 30.2 (CH_2), 29.8 (CH_2), 29.7 (CH_3), 29.6 (CH_2), 29.4 (CH_2), 24.8 (CH_2), 22.8 (CH_2), 14.2 (CH_3). **HRMS** (ESI) m/z $(M+H)^+$: calculated for $(C_{22}H_{35}N_2O)^+$: 343.2744, found: 343.2746.

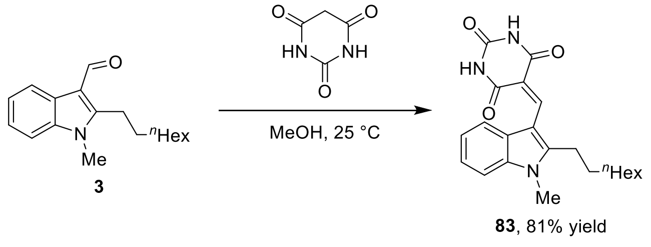

To a flame-dried and N_2-purged Schlenk tube were added **3** (0.1 mmol, 27.1 mg), barbituric acid (0.15 mmol, 19.2 mg) and MeOH (1 mL). The resulting mixture was stirred at 25 °C for 48 hours and then the yellow solution was concentrated *in vacuo*. The crude product was purified by column chromatography on silica gel (*n*-hexane: ethyl acetate = 1:1) to afford the desired product **83** (30.8 mg, 81% yield).

5-((1-Methyl-2-octyl-1*H*-indol-3-yl)methylene)pyrimidine-2,4,6(1*H*,3*H*,5*H*)-trione (83)

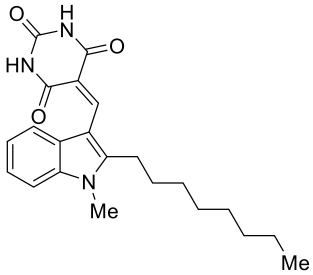

Yellow solid. **M.p.**: 211-213 °C. **^{1}H NMR (300 MHz, DMSO-*d*$_6$)** δ 11.03 (s, 1H), 10.87 (s, 1H), 8.47 (s, 1H), 7.58 (d, *J* = 8.2 Hz, 1H), 7.27 (ddd, *J* = 8.2, 6.9, 1.5 Hz, 1H), 7.19 (td, *J* = 7.4, 6.9, 1.1 Hz, 1H), 7.13 (dd, *J* = 8.1, 1.4 Hz, 1H), 3.85 (s, 3H), 3.03 (t, *J* = 7.5 Hz, 2H), 1.58 (q, *J* = 6.9 Hz, 2H), 1.35 – 1.15 (m, 10H), 0.88 – 0.76 (m, 3H). **^{13}C NMR (75 MHz, DMSO-*d*$_6$)** δ 164.5 (C_q), 161.5 (C_q), 155.2 (C_q), 150.6 (C_q), 145.9 (CH), 138.1 (C_q), 125.3 (C_q), 124.4 (CH), 122.6 (CH), 121.5 (CH), 111.5 (C_q), 110.6 (CH), 109.0 (C_q), 31.2 (CH_2), 30.6 (CH_3), 29.3 (CH_2), 28.6 (CH_2), 28.5 (CH_2), 24.7 (CH_2), 22.1 (CH_2), 13.9 (CH_3). **HRMS** (ESI) m/z $(M+H)^+$: calculated for $(C_{22}H_{28}N_3O_3)^+$: 382.2125, found: 382.2108.

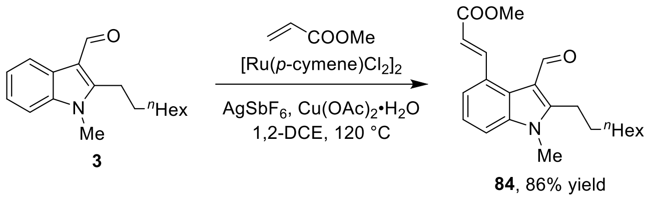

To a flame-dried and N_2-purged Schlenk tube were added **3** (0.1 mmol, 27.1 mg), $[Ru(p\text{-cymene})Cl_2]_2$ (5 mol%, 0.005 mmol, 3.1 mg), $AgSbF_6$ (20 mol%, 0.02 mmol, 6.8 mg) and $Cu(OAc)_2{\cdot}H_2O$ (0.05 mmol, 9.9 mg). The Schlenk tube was then sealed, purged and backfilled with N_2 three times. Methyl acrylate (0.3 mmol, 27 μL) and 1,2-DCE (1 mL) were added *via* syringe. The resulting mixture was stirred at 120 °C for 24 hours. After cooling to room temperature, the reaction mixture was diluted with ethyl acetate and filtered through a pad of Celite. The resulting solution was concentrated *in vacuo* and the crude product was purified by column chromatography on silica gel (*n*-hexane: ethyl acetate = 2:1) to afford the desired product **84** (30.5 mg, 86% yield).

Methyl (*E*)-3-(3-formyl-1-methyl-2-octyl-1*H*-indol-4-yl)acrylate (84)

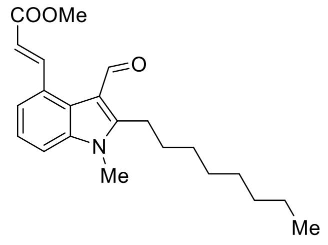

Yellow oil. **^{1}H NMR (300 MHz, $CDCl_3$)** δ 10.20 (s, 1H), 9.03 (d, J = 15.8 Hz, 1H), 7.48 (d, J = 7.3 Hz, 1H), 7.34 (dd, J = 8.2, 1.2 Hz, 1H), 7.30 – 7.23 (m, 1H), 6.38 (d, J = 15.8 Hz, 1H), 3.84 (s, 3H), 3.73 (s, 3H), 3.20 – 3.02 (m, 2H), 1.73 – 1.57 (m, 2H), 1.50 – 1.35 (m, 2H), 1.37 – 1.20 (m, 8H), 0.93 – 0.79 (m, 3H). **^{13}C NMR (75 MHz, $CDCl_3$)** δ 184.2 (CH), 167.6 (C_q), 153.9 (C_q), 145.7 (CH), 138.0 (C_q), 128.9 (C_q), 124.8 (C_q), 123.1 (CH), 121.5 (CH), 118.9 (CH), 114.8 (C_q), 111.1 (CH), 51.8 (CH_3), 31.9 (CH_2), 30.0 (CH_2), 30.0 (CH_3), 29.6 (CH_2), 29.4 (CH_2), 29.3 (CH_2), 25.3 (CH_2), 22.7 (CH_2), 14.2 (CH_3). **HRMS** (ESI) m/z $(M+H)^+$: calculated for $(C_{22}H_{30}NO_3)^+$: 356.2220, found: 356.2222.

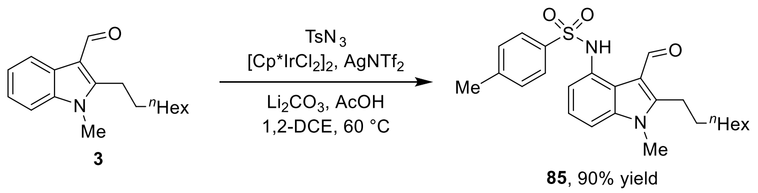

To a flame-dried and N_2-purged Schlenk tube were added **3** (0.1 mmol, 27.1 mg), TsN_3 (0.12 mmol, 23.6 mg), $[Cp^*IrCl_2]_2$ (5 mol%, 0.005 mmol, 4.0 mg), $AgNTf_2$ (20 mol%, 0.02 mmol, 7.8 mg) and Li_2CO_3 (0.1 mmol, 7.4 mg). The Schlenk tube was then sealed, purged and backfilled with N_2 three times. AcOH (0.1 mmol, 6 μL) and 1,2-DCE (1 mL) were added *via* syringe. The resulting mixture was stirred at 60 °C for 4 hours. After cooling to room temperature, the reaction mixture was diluted with ethyl acetate and filtered through a pad of Celite. The resulting solution was concentrated *in vacuo* and the crude product was purified by column chromatography on silica gel (*n*-hexane: ethyl acetate = 1:1) to afford the desired product **85** (39.8 mg, 90% yield).

***N*-(3-Formyl-1-methyl-2-octyl-1*H*-indol-4-yl)-4-methylbenzenesulfonamide (85)**

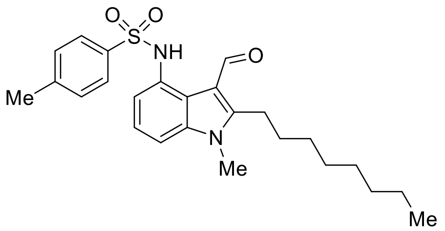

White solid. **M.p.**: 146-148 °C. **^{1}H NMR (300 MHz, $CDCl_3$)** δ 11.85 (s, 1H), 9.67 (s, 1H), 7.75 (d, *J* = 8.3 Hz, 2H), 7.44 (dd, *J* = 8.0, 0.8 Hz, 1H), 7.17 (t, *J* = 8.1 Hz, 1H), 7.12 (d, *J* = 7.8 Hz, 2H), 6.91 (dd, *J* = 8.2, 0.8 Hz, 1H), 3.65 (s, 3H), 3.07 – 2.92 (m, 2H), 2.29 (s, 3H), 1.70 – 1.54 (m, 2H), 1.46 – 1.35 (m, 2H), 1.36 – 1.21 (m, 8H), 0.98 – 0.80 (m, 3H). **^{13}C NMR (75 MHz, $CDCl_3$)** δ 184.4 (CH), 155.1 (C_q), 143.1 (C_q), 139.0 (C_q), 137.5 (C_q), 132.7 (C_q), 129.4 (CH), 127.5 (CH), 125.2 (CH), 116.6 (C_q), 114.2 (C_q), 111.9 (CH), 104.9 (CH), 31.9 (CH_2), 30.6 (CH_2), 30.4 (CH_3), 29.5 (CH_2), 29.4 (CH_2), 29.2 (CH_2), 24.7 (CH_2), 22.7 (CH_2), 21.6 (CH_3), 14.2 (CH_3). **HRMS** (ESI) m/z $(M+H)^+$: calculated for $(C_{25}H_{33}N_2O_3S)^+$: 441.2206, found: 441.2207.

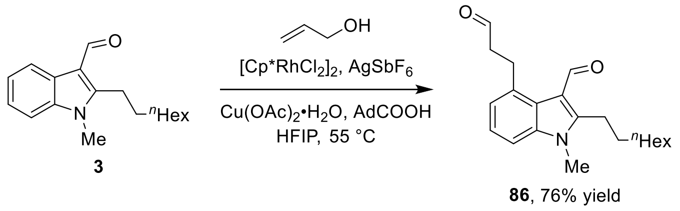

To a flame-dried and N_2-purged Schlenk tube were added **3** (0.1 mmol, 27.1 mg), $[Cp^*RhCl_2]_2$ (5 mol%, 0.005 mmol, 3.1 mg), $AgSbF_6$ (20 mol%, 0.02 mmol, 6.8 mg), $Cu(OAc)_2{\cdot}H_2O$ (0.25 mmol, 49.7 mg) and 1-AdCOOH (0.125 mmol, 22.5 mg). The Schlenk tube was then sealed, purged and backfilled with N_2 three times. Allyl alcohol (0.3 mmol, 20 μL) and HFIP (1 mL) were added *via* syringe. The resulting mixture was stirred at 55 °C for 24 hours. After cooling to room temperature, the reaction mixture was diluted with ethyl acetate and filtered through a pad of Celite. The resulting solution was concentrated *in vacuo* and the crude product was purified by column chromatography on silica gel (*n*-hexane: ethyl acetate = 5:1) to afford the desired product **86** (25.0 mg, 76% yield).

1-Methyl-2-octyl-4-(3-oxopropyl)-1*H*-indole-3-carbaldehyde (86)

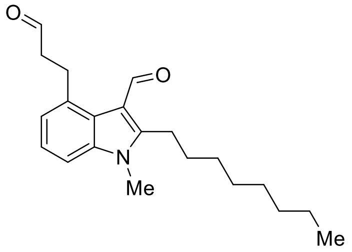

Pale-yellow oil. **^{1}H NMR (300 MHz, $CDCl_3$)** δ 10.17 (s, 1H), 9.86 (t, *J* = 1.8 Hz, 1H), 7.25 – 7.17 (m, 2H), 7.09 (dd, *J* = 5.2, 3.2 Hz, 1H), 3.73 (s, 3H), 3.60 (t, *J* = 7.5 Hz, 2H), 3.20 – 3.07 (m, 2H), 2.79 (td, *J* = 7.5, 1.8 Hz, 2H), 1.74 – 1.60 (m, 2H), 1.53 – 1.38 (m, 2H), 1.39 – 1.22 (m, 8H), 0.97 – 0.81 (m, 3H). **^{13}C NMR (75 MHz, $CDCl_3$)** δ 202.9 (CH), 184.1 (CH), 153.7 (C_q), 138.2 (C_q), 134.7 (C_q), 124.3 (CH), 124.1 (C_q), 123.5 (CH), 114.5 (C_q), 108.1 (CH), 45.9 (CH_2), 31.9 (CH_2), 30.3 (CH_2), 30.0 (CH_3), 29.7 (CH_2), 29.5 (CH_2), 29.3 (CH_2), 29.1 (CH_2), 25.2 (CH_2), 22.8 (CH_2), 14.2 (CH_3). **HRMS** (ESI) m/z $(M+H)^+$: calculated for $(C_{21}H_{30}NO_2)^+$: 328.2271, found: 328.2274.

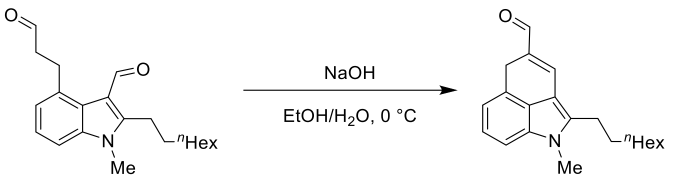

86 **87**, 92% yield

To a solution of 86 (0.05 mmol, 16.4 mg) in EtOH (0.5 mL) at 0 °C was added an aqueous solution of NaOH (2 M, 0.15 mmol, 75 μL). The resulting mixture was stirred at 0 °C for 1 hour, then ethyl acetate (5 mL) and water (5 mL) were added. The phases were separated and the aqueous layer was extracted with ethyl acetate (3 × 5 mL). The combined organic layer was washed with brine, dried over Na_2SO_4 and concentrated *in vacuo* to afford the desired product **87** (14.2 mg, 92% yield).

1-Methyl-2-octyl-1,5-dihydrobenzo[*cd*]indole-4-carbaldehyde (87)

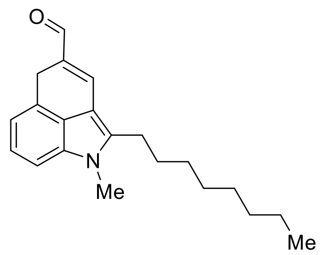

Yellow solid. **M.p.**: 88-89 °C. **^{1}H NMR (300 MHz, CDCl$_3$)** δ 9.51 (s, 1H), 7.41 (t, *J* = 1.5 Hz, 1H), 7.14 (dd, *J* = 8.1, 7.2 Hz, 1H), 6.96 (d, *J* = 7.9 Hz, 1H), 6.91 (dq, *J* = 7.1, 1.2 Hz, 1H), 4.03 (s, 2H), 3.64 (s, 3H), 2.83 (t, *J* = 7.6 Hz, 2H), 1.75 – 1.58 (m, 2H), 1.49 – 1.17 (m, 10H), 0.92 – 0.83 (m, 3H). **^{13}C NMR (75 MHz, CDCl$_3$)** δ 192.5 (CH), 140.8 (CH), 140.6 (C_q), 136.1 (C_q), 134.6 (C_q), 129.0 (C_q), 127.5 (C_q), 124.6 (CH), 118.5 (CH), 110.1 (C_q), 106.5 (CH), 32.0 (CH_2), 30.3 (CH_3), 29.7 (CH_2), 29.5 (CH_2), 29.5 (CH_2), 29.3 (CH_2), 27.2 (CH_2), 25.3 (CH_2), 22.8 (CH_2), 14.2 (CH_3). **HRMS** (ESI) m/z $(M+H)^+$: calculated for $(C_{21}H_{28}NO)^+$: 310.2165, found: 310.2167.

8. Mechanistic investigations

8.1 Reaction with iron(II) complex

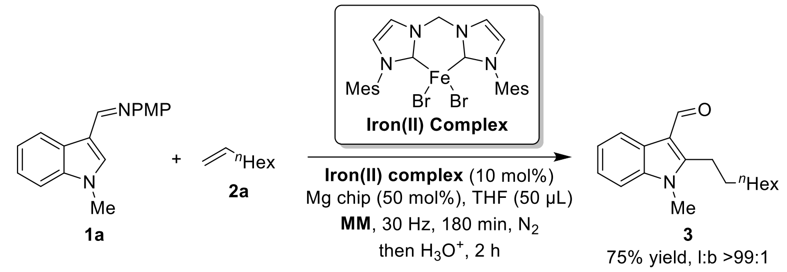

In the glove box, a mixture of indole substrate **1a** (0.2 mmol, 52.8 mg), 1-octene (**2a**) (0.3 mmol, 47 μL), **Iron(II) Complex**[12] (10 mol%, 0.02 mmol, 12.0 mg), magnesium chip (50 mol%, 0.1 mmol, 2.4 mg) and tetrahydrofuran (50 μL) were placed in a nitrogen-purged stainless-steel vessel (5 mL) with a stainless-steel ball (d_{MB} = 7 mm). Then, the vessel was sealed and milled in a mixer mill (RETSCH MM 400) at 30 Hz for 180 min under nitrogen atmosphere. Then, the reaction mixture was diluted with tetrahydrofuran (3 mL) and quenched with HCl aqueous solution (3 M, 1 mL). The resulting mixture was stirred at room temperature for 2 hours. The phases were then separated, the aqueous layer was extracted with ethyl acetate (5 mL ×3). The combined organic layer was washed with saturated $NaHCO_3$ solution and brine, dried over Na_2SO_4, filtered and concentrated *in vacuo*. The linear and branched ratio was determined by ^{1}H NMR analysis of the crude reaction mixture. The residue was purified by column chromatography on silica gel (*n*-hexane: ethyl acetate = 10:1) to afford the desired product **3** (40.8 mg, 75% yield, l:b >99:1).

8.2 Deuterium-labeling experiment

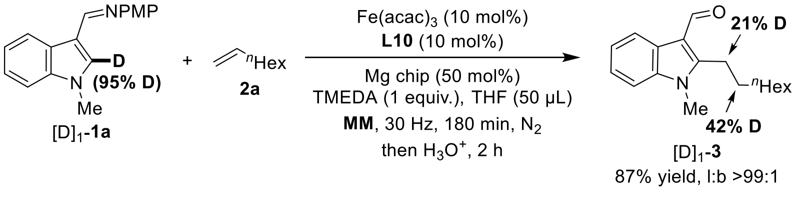

In the glove box, a mixture of indole substrate [D]-**1a** (0.2 mmol, 53.0 mg), 1-octene (**2a**) (0.3 mmol, 47 μL), $Fe(acac)_3$ (10 mol%, 0.02 mmol, 7.1 mg), **L10** (10 mol%, 0.02 mmol, 10.9 mg), magnesium chip (50 mol%, 0.1 mmol, 2.4 mg),

TMEDA (0.2 mmol, 30 μL) and tetrahydrofuran (50 μL) were placed in a nitrogen-purged stainless-steel vessel (5 mL) with a stainless-steel ball (d_{MB} = 7 mm). Then, the vessel was sealed and milled in a mixer mill (RETSCH MM 400) at 30 Hz for 180 min under nitrogen atmosphere. Then, the reaction mixture was diluted with tetrahydrofuran (3 mL) and quenched with HCl aqueous solution (3 M, 1 mL). The resulting mixture was stirred at room temperature for 2 hours. The phases were then separated, the aqueous layer was extracted with ethyl acetate (5 mL ×3). The combined organic layer was washed with saturated $NaHCO_3$ solution and brine, dried over Na_2SO_4, filtered and concentrated *in vacuo*. The linear and branched ratio was determined by ^{1}H NMR analysis of the crude reaction mixture. The residue was purified by column chromatography on silica gel (*n*-hexane: ethyl acetate = 10:1) to afford the product. Deuterium contents were determined by ^{1}H NMR spectroscopic analysis.

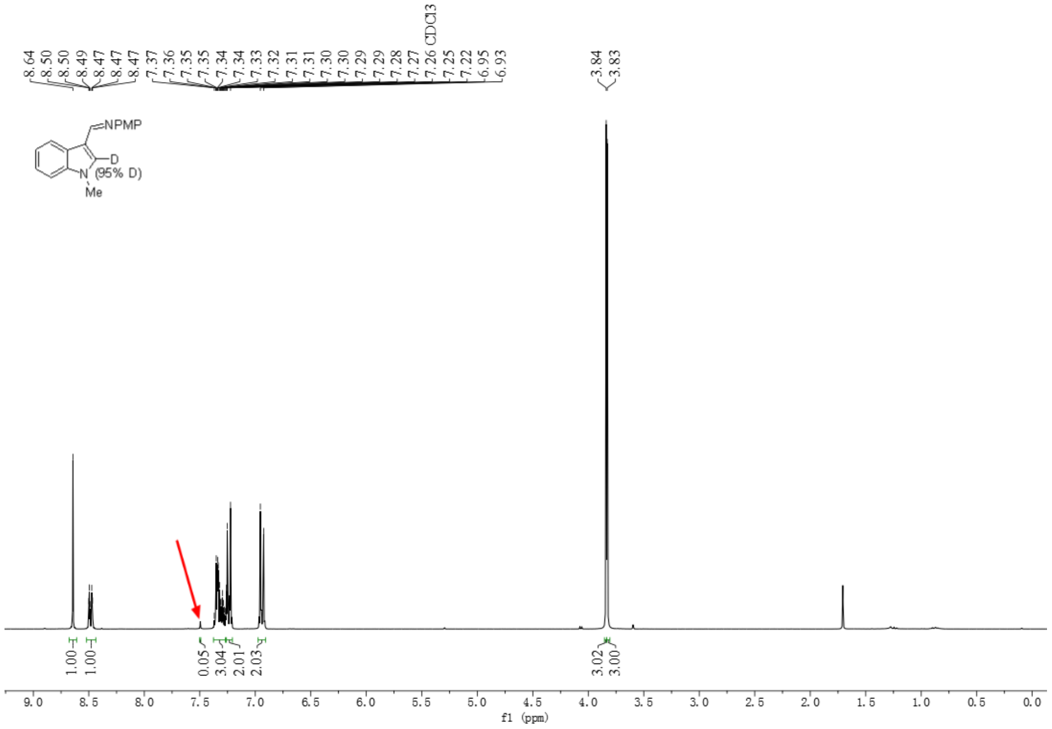

Figure S1. ^{1}H NMR (300 MHz, $CDCl_3$) of [D]$_1$-**1a**.

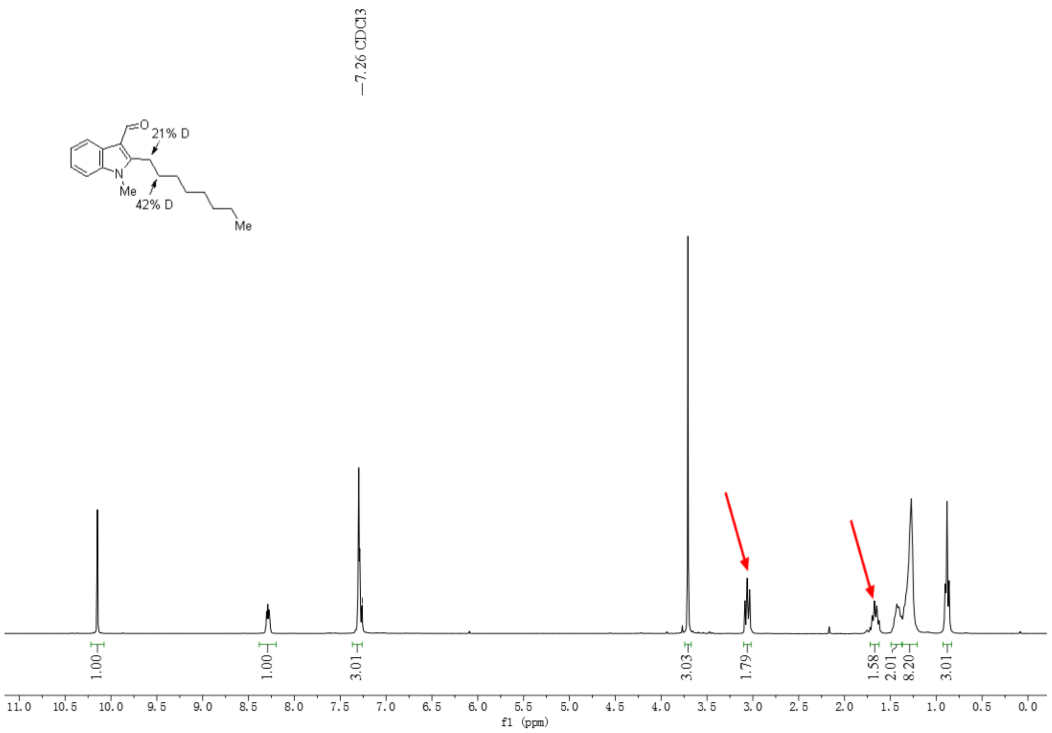

Figure S2. ^{1}H NMR (300 MHz, $CDCl_3$) of [D]$_1$-**3**.

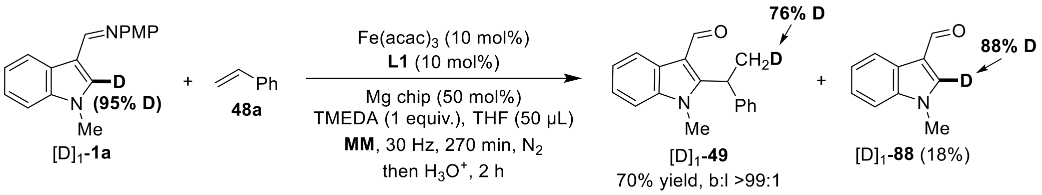

In the glove box, a mixture of indole substrate [D]-**1a** (0.2 mmol, 53.0 mg), styrene (**48a**) (0.3 mmol, 34 μL), $Fe(acac)_3$ (10 mol%, 0.02 mmol, 7.1 mg), **L1** (10 mol%, 0.02 mmol, 6.8 mg), magnesium chip (50 mol%, 0.1 mmol, 2.4 mg), TMEDA (0.2 mmol, 30 μL) and tetrahydrofuran (50 μL) were placed in a nitrogen-purged stainless-steel vessel (5 mL) with a stainless-steel ball (d_{MB} = 7 mm). Then, the vessel was sealed and milled in a mixer mill (RETSCH MM 400) at 30 Hz for 270 min under nitrogen atmosphere. Then, the reaction mixture was diluted with tetrahydrofuran (3 mL) and quenched with HCl aqueous solution (3 M, 1 mL). The resulting mixture was stirred at room temperature for 2 hours. The phases were then separated, the aqueous layer was extracted with ethyl acetate (5 mL ×3). The combined organic layer was washed with saturated $NaHCO_3$ solution and brine, dried over Na_2SO_4, filtered and concentrated *in vacuo*. The linear and branched ratio was determined by ^{1}H NMR analysis of the crude reaction mixture. The residue was purified

by column chromatography on silica gel (*n*-hexane: ethyl acetate = 5:1) to afford the product and hydrolyzed starting material. Deuterium contents were determined by ^{1}H NMR spectroscopic analysis.

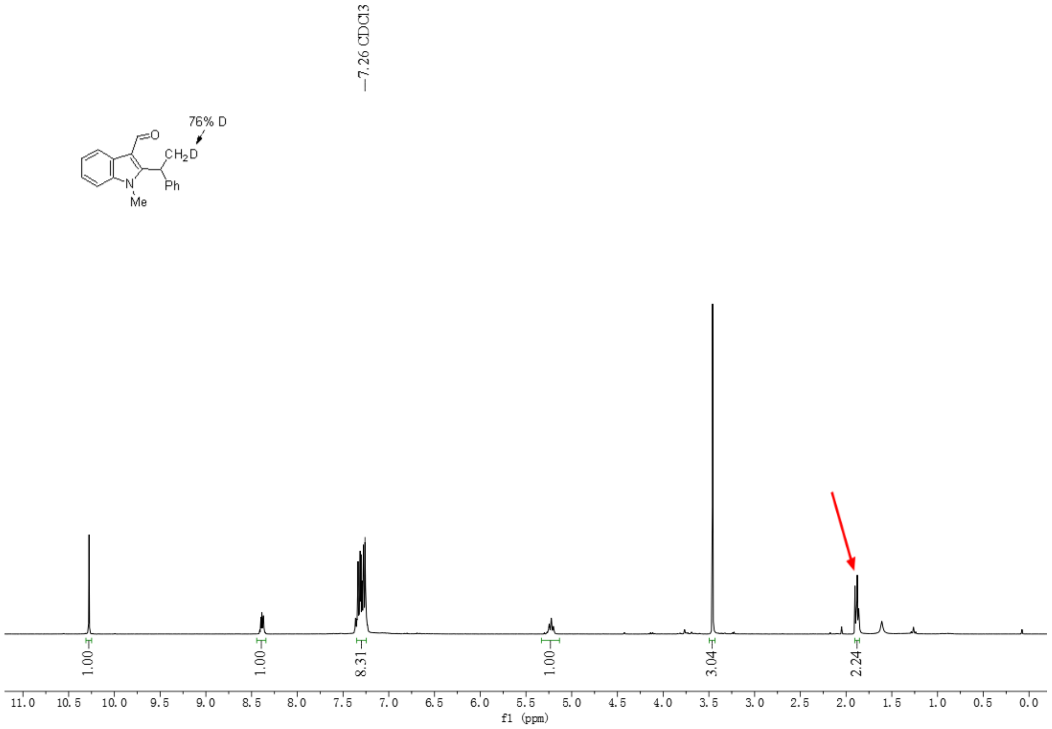

Figure S3. ^{1}H NMR (300 MHz, $CDCl_3$) of [D]$_1$-**49**.

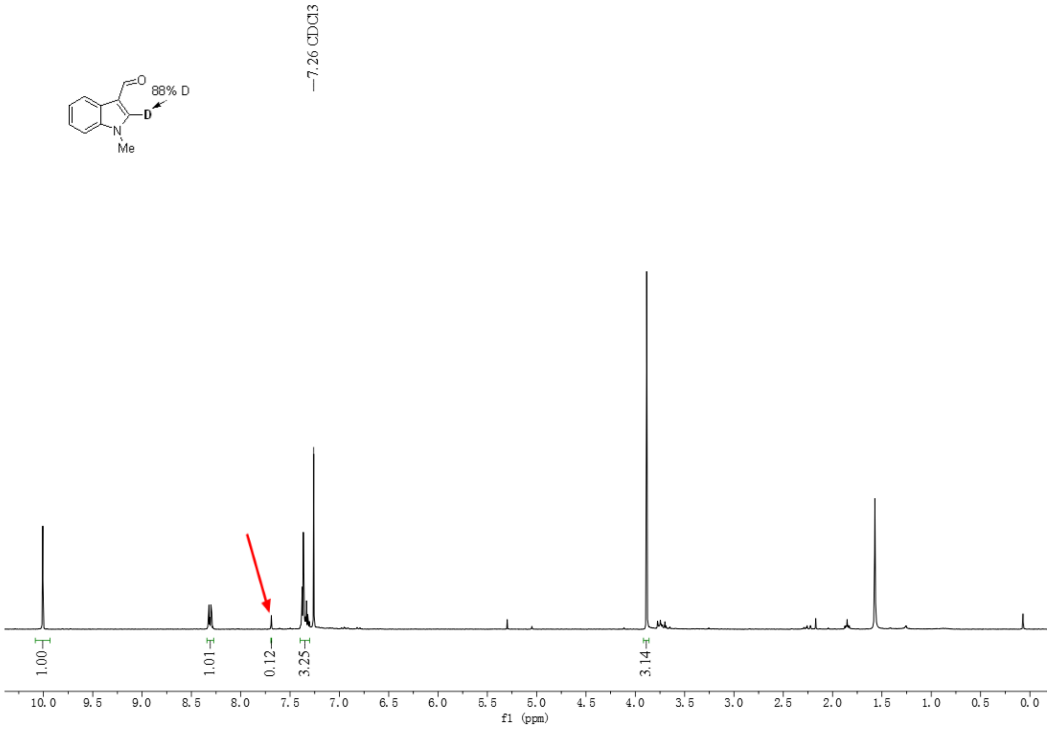

Figure S4. ^{1}H NMR (300 MHz, $CDCl_3$) of [D]$_1$-**88**.

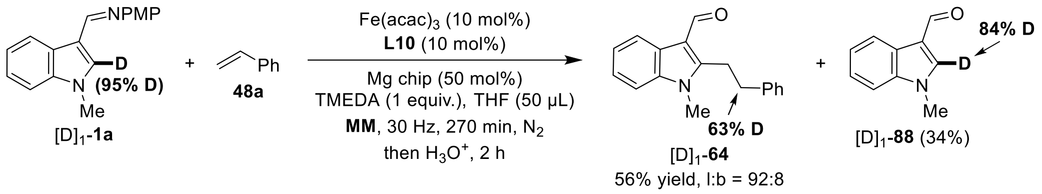

In the glove box, a mixture of indole substrate [D]-**1a** (0.2 mmol, 53.0 mg), styrene (**48a**) (0.3 mmol, 34 μL), Fe(acac)$_3$ (10 mol%, 0.02 mmol, 7.1 mg), **L10** (10 mol%, 0.02 mmol, 10.9 mg), magnesium chip (50 mol%, 0.1 mmol, 2.4 mg), TMEDA (0.2 mmol, 30 μL) and tetrahydrofuran (50 μL) were placed in a nitrogen-purged stainless-steel vessel (5 mL) with a stainless-steel ball (d_{MB} = 7 mm). Then, the vessel was sealed and milled in a mixer mill (RETSCH MM 400) at 30 Hz for 270 min under nitrogen atmosphere. Then, the reaction mixture was diluted with tetrahydrofuran (3 mL) and quenched with HCl aqueous solution (3 M, 1 mL). The resulting mixture was stirred at room temperature for 2 hours. The phases were then separated, the aqueous layer was extracted with ethyl acetate (5 mL ×3). The combined organic layer was washed with saturated $NaHCO_3$ solution and brine, dried over Na_2SO_4, filtered and concentrated *in vacuo*. The linear and branched ratio was determined by ^{1}H NMR analysis of the crude reaction mixture. The residue was purified by column chromatography on silica gel (*n*-hexane: ethyl acetate = 5:1) to afford the product and hydrolyzed starting material. Deuterium contents were determined by ^{1}H NMR spectroscopic analysis.

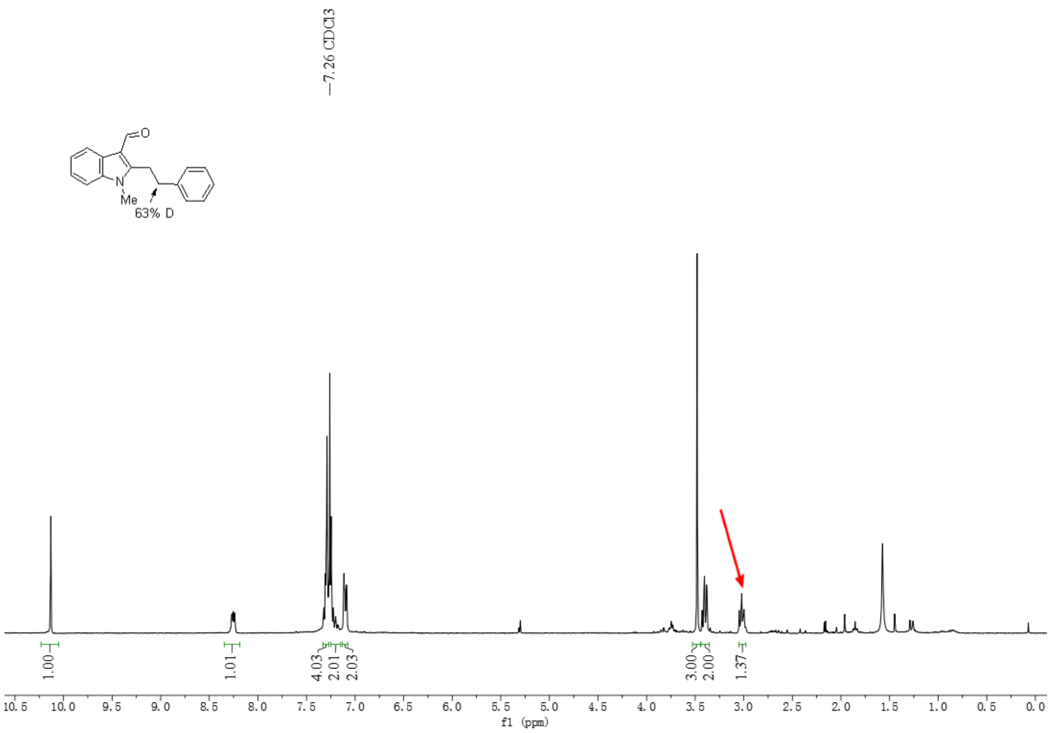

Figure S5. ^{1}H NMR (300 MHz, $CDCl_3$) of $[D]_1$-**64**.

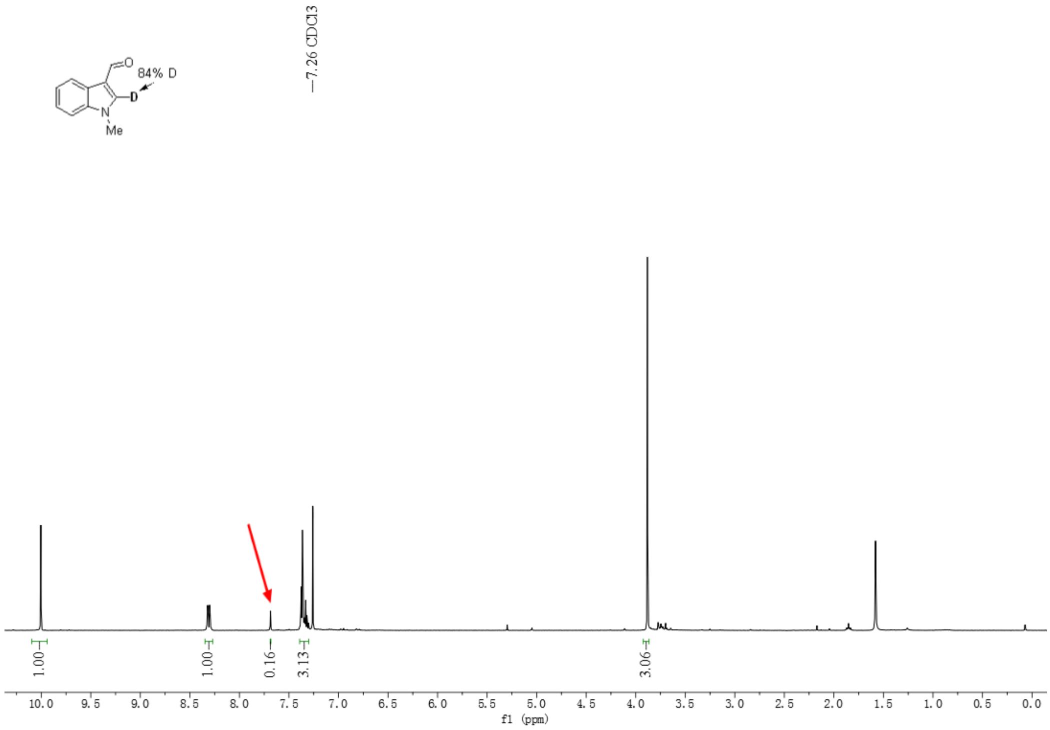

Figure S6. ^{1}H NMR (300 MHz, $CDCl_3$) of $[D]_1$-**88**.

8.3 Deuterium-scrambling experiment

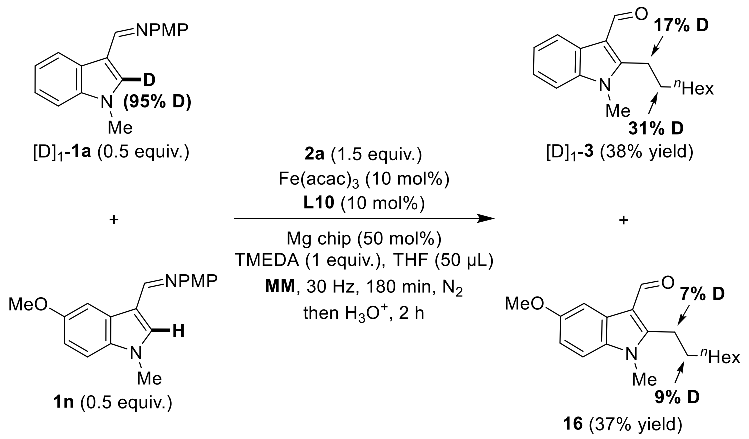

In the glove box, a mixture of indole substrate [D]-**1a** (0.1 mmol, 26.5 mg), indole substrate **1n** (0.1 mmol, 29.4 mg), 1-octene (**2a**) (0.3 mmol, 47 μL), Fe(acac)$_3$ (10 mol%, 0.02 mmol, 7.1 mg), **L10** (10 mol%, 0.02 mmol, 10.9 mg), magnesium chip (50 mol%, 0.1 mmol, 2.4 mg), TMEDA (0.2 mmol, 30 μL) and tetrahydrofuran (50 μL) were placed in a nitrogen-purged stainless-steel vessel (5 mL) with a stainless-steel ball (d_{MB} = 7 mm). Then, the vessel was sealed and milled in a mixer mill (RETSCH MM 400) at 30 Hz for 180 min under nitrogen atmosphere. Then, the reaction mixture was diluted with tetrahydrofuran (3 mL) and quenched with HCl aqueous solution (3 M, 1 mL). The resulting mixture was stirred at room temperature for 2 hours. The phases were then separated, the aqueous layer was extracted with ethyl acetate (5 mL ×3). The combined organic layer was washed with saturated $NaHCO_3$ solution and brine, dried over Na_2SO_4, filtered and concentrated *in vacuo*. The residue was purified by column chromatography on silica gel (*n*-hexane: ethyl acetate = 10:1) to afford the products. Deuterium contents were determined by ^{1}H NMR spectroscopic analysis.

8.4 Test reaction with Grignard reagent system

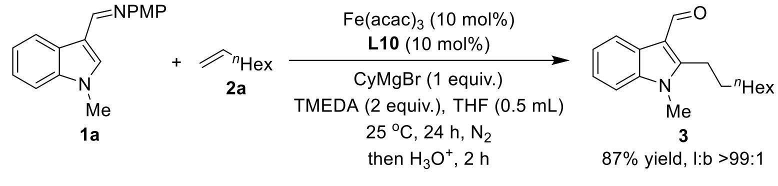

To a flame-dried and N_2-purged Schlenk tube were added indole substrate **1a** (0.2 mmol, 52.8 mg), Fe(acac)$_3$ (10 mol%, 0.02 mmol, 7.1 mg) and **L10** (10 mol%,

0.02 mmol, 10.9 mg). The Schlenk tube was then sealed, purged and backfilled with N_2 three times. 1-octene (**2a**) (0.3 mmol, 47 μL), TMEDA (0.4 mmol, 60 μL) and tetrahydrofuran (0.5 mL) were added *via* syringe. CyMgBr (1 M in THF, 0.2 mmol, 0.2 mL) was then added dropwise and the resulting mixture was stirred at 25 °C for 24 hours. Then, the reaction mixture was diluted with tetrahydrofuran (2 mL) and quenched with HCl aqueous solution (3 M, 1 mL). The resulting mixture was stirred at room temperature for 2 hours. The phases were then separated, the aqueous layer was extracted with ethyl acetate (5 mL ×3). The combined organic layer was washed with brine, dried over Na_2SO_4, filtered and concentrated *in vacuo*. The linear and branched ratio was determined by ^{1}H NMR analysis of the crude reaction mixture. The residue was purified by column chromatography on silica gel (*n*-hexane: ethyl acetate = 10:1) to afford the desired product **3** (47.0 mg, 87% yield, l:b >99:1).

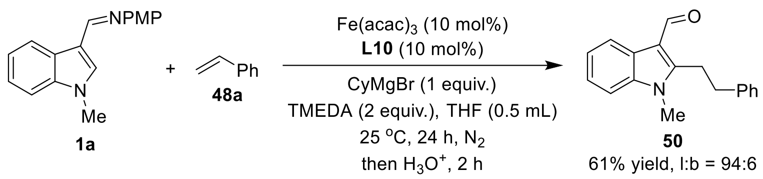

To a flame-dried and N_2-purged Schlenk tube were added indole substrate **1a** (0.2 mmol, 52.8 mg), $Fe(acac)_3$ (10 mol%, 0.02 mmol, 7.1 mg) and **L10** (10 mol%, 0.02 mmol, 10.9 mg). The Schlenk tube was then sealed, purged and backfilled with N_2 three times. styrene (**48a**) (0.3 mmol, 34 μL), TMEDA (0.4 mmol, 60 μL) and tetrahydrofuran (0.5 mL) were added *via* syringe. CyMgBr (1 M in THF, 0.2 mmol, 0.2 mL) was then added dropwise and the resulting mixture was stirred at 25 °C for 24 hours. Then, the reaction mixture was diluted with tetrahydrofuran (2 mL) and quenched with HCl aqueous solution (3 M, 1 mL). The resulting mixture was stirred at room temperature for 2 hours. The phases were then separated, the aqueous layer was extracted with ethyl acetate (5 mL ×3). The combined organic layer was washed with brine, dried over Na_2SO_4, filtered and concentrated *in vacuo*. The linear and branched ratio was determined by ^{1}H NMR analysis of the crude reaction mixture. The residue was purified by column chromatography on silica gel (*n*-hexane: ethyl acetate = 5:1) to

afford the desired product **50** (32.2 mg, 61% yield, l:b = 94:6).

8.5 Kinetic isotope effect experiment

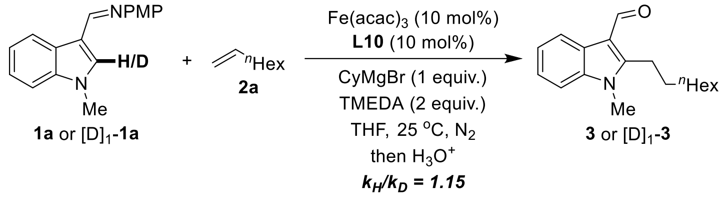

The kinetic isotope effect (KIE) was examined by applying the initial rate method. To a flame-dried and N_2-purged Schlenk tube were added indole substrate **1a** (0.2 mmol, 52.8 mg), $Fe(acac)_3$ (10 mol%, 0.02 mmol, 7.1 mg), **L10** (10 mol%, 0.02 mmol, 10.9 mg) and 1,3,5-trimethoxybenzene (0.067 mmol, 11.2 mg). The Schlenk tube was then sealed, purged and backfilled with N_2 three times. 1-octene (**2a**) (0.3 mmol, 47 μL), TMEDA (0.4 mmol, 60 μL) and tetrahydrofuran (0.5 mL) were added *via* syringe. CyMgBr (1 M in THF, 0.2 mmol, 0.2 mL) was then added dropwise and the resulting mixture was stirred at 25 °C (t = 0 min). Aliquots (50 μL) were removed periodically every 2 min. The conversion was determined by ^{1}H NMR using 1,3,5-trimethoxybenzene as the internal standard. Then the same procedure was applied with indole substrate $[D]_1$-**1a**.

The following results were obtained.

Table S5. Kinetic isotope effect 1

Entry	Time (min)	Yield (%) (with **1a**)	Yield (%) (with $[D]_1$-**1a**)
1	2	0.5	0.5
2	4	4.5	4
3	6	8	7
4	8	11	10
5	10	14	12.5
6	12	17.5	15

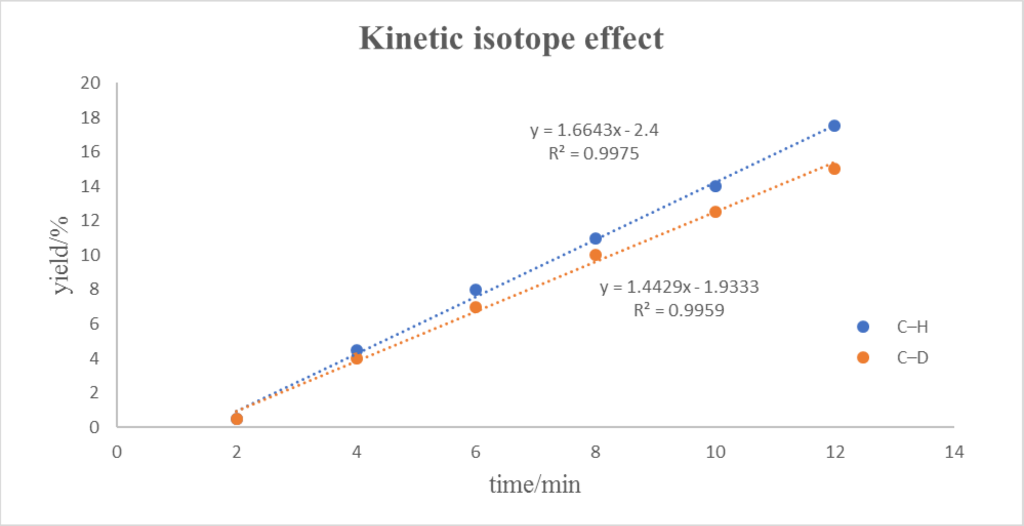

Figure S7. Kinetic isotope effect 1

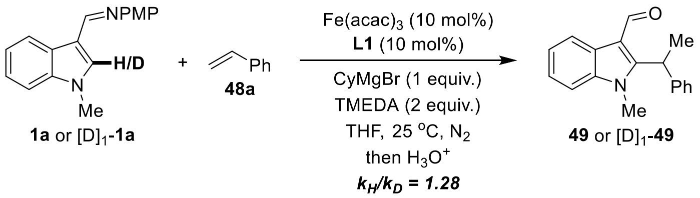

The kinetic isotope effect (KIE) was examined by applying the initial rate method. To a flame-dried and N_2-purged Schlenk tube were added indole substrate **1a** (0.2 mmol, 52.8 mg), $Fe(acac)_3$ (10 mol%, 0.02 mmol, 7.1 mg), **L1** (10 mol%, 0.02 mmol, 6.8 mg) and 1,3,5-trimethoxybenzene (0.067 mmol, 11.2 mg). The Schlenk tube was then sealed, purged and backfilled with N_2 three times. styrene (**48a**) (0.3 mmol, 34 μL), TMEDA (0.4 mmol, 60 μL) and tetrahydrofuran (0.5 mL) were added *via* syringe. CyMgBr (1 M in THF, 0.2 mmol, 0.2 mL) was then added dropwise and the resulting mixture was stirred at 25 °C (t = 0 min). Aliquots (50 μL) were removed periodically every 5 min. The conversion was determined by ^{1}H NMR using 1,3,5-trimethoxybenzene as the internal standard. Then the same procedure was applied with indole substrate $[D]_1$-**1a**.

The following results were obtained.

Table S6. Kinetic isotope effect 2

Entry	Time (min)	Yield (%) (with **1a**)	Yield (%) (with $[D]_1$-**1a**)
1	5	2	0.5
2	10	5	3
3	15	8.5	7
4	20	14	10.5
5	25	18.5	14.5
6	30	23	16.5

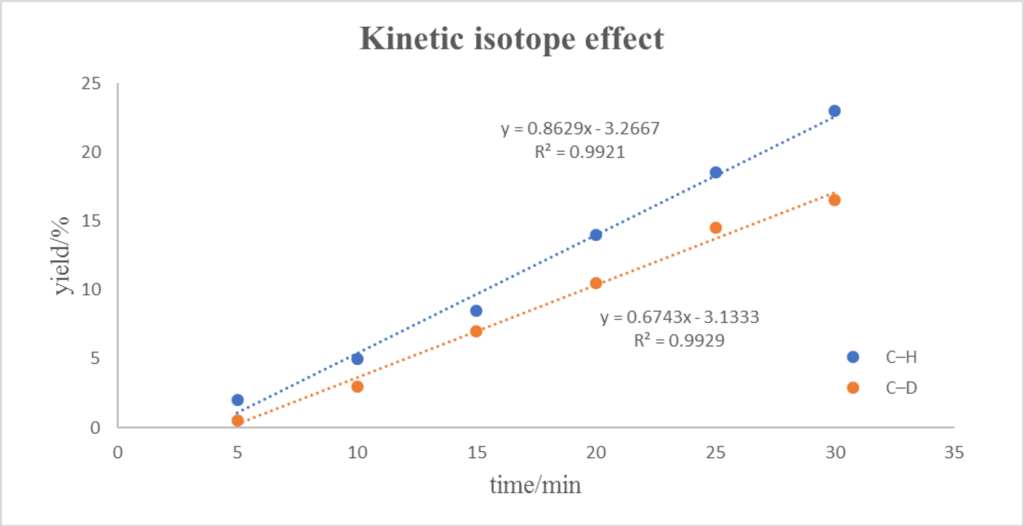

Figure S8. Kinetic isotope effect 2

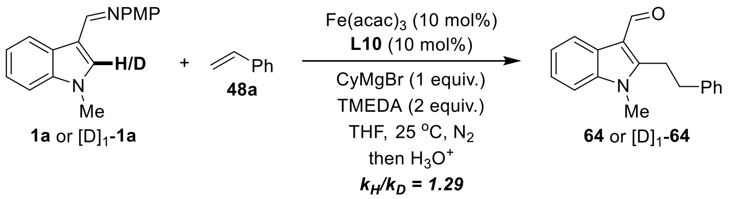

The kinetic isotope effect (KIE) was examined by applying the initial rate method. To a flame-dried and N_2-purged Schlenk tube were added indole substrate **1a** (0.2 mmol, 52.8 mg), $Fe(acac)_3$ (10 mol%, 0.02 mmol, 7.1 mg), **L10** (10 mol%, 0.02 mmol, 10.9 mg) and 1,3,5-trimethoxybenzene (0.067 mmol, 11.2 mg). The Schlenk tube was then sealed, purged and backfilled with N_2 three times. styrene (**48a**) (0.3 mmol, 34 μL), TMEDA (0.4 mmol, 60 μL) and tetrahydrofuran (0.5 mL) were added *via* syringe. CyMgBr (1 M in THF, 0.2 mmol, 0.2 mL) was then added dropwise and the resulting mixture was stirred at 25 °C (t = 0 min). Aliquots (50 μL) were removed periodically

every 20 min. The conversion was determined by ^{1}H NMR using 1,3,5-trimethoxybenzene as the internal standard. Then the same procedure was applied with indole substrate [D]$_1$-**1a**.

The following results were obtained.

Table S7. Kinetic isotope effect 3

Entry	Time (min)	Yield (%) (with **1a**)	Yield (%) (with [D]$_1$-**1a**)
1	20	1.5	0.5
2	40	4	2.5
3	60	6.5	3
4	80	9	6
5	100	13	9
6	120	15	11

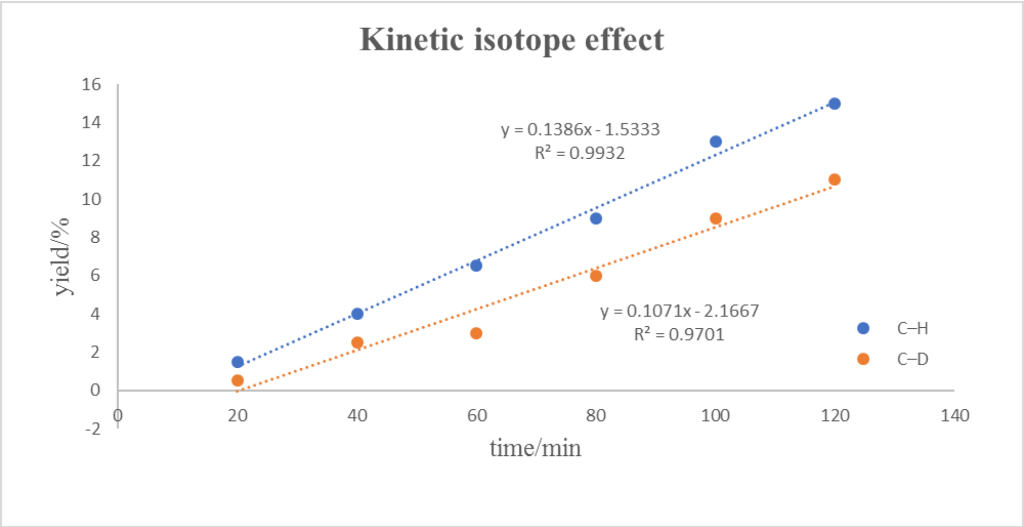

Figure S9. Kinetic isotope effect 3

9. Computational studies

9.1 Comnputational details

All DFT calculations were carried out with the Gaussian 16 program.[13] The geometry optimizations were conducted using the B3LYP functional[14] including Grimme's dispersion corrections[15,16] with a Becke-Johnson damping function. Def2-SVP basis set[17] was used for all the atoms. To confirm whether each optimized stationary point is an energy minimum or a transition state as well as to evaluate the zero-point vibrational energy and thermal corrections at 298 K, vibrational frequencies were computed at the same level of theory as for the geometry optimizations. On the basis of the gas-phase optimized structures, the single-point energies and solvent effects were evaluated at the TPSSh level of theory[18] including Grimme's dispersion corrections with a Becke-Johnson damping using def2-TZVP basis set[19] for all the atoms. The solvation energies were calculated using the self-consistent reaction field with the SMD implicit solvent model[20].

The 3D diagrams of computed species were generated by CYLView[21].

All the DFT optimized structures are provided in the format of XYZ files along with the *Supplementary Information*.

9.2 Multivariate linear regression

Multivariate modeling[22] was performed based on an assumption of linear relationships. The generalized model structure is as follows, consisting of a constant term, first-ordered parameters (*P*) and their coefficients (*β*).

$$\Delta\Delta G = const. + \sum_i \beta_i P_i$$

The free energy differences $\Delta\Delta G$ between branched and linear products are converted from the experimental regioselectivity ratio using the equation as follows, where *T* is the temperature at which the reaction was performed and *R* is the gas constant.

$$\Delta\Delta G = -RT\ln\frac{n_{branched}}{n_{linear}}$$

The multi-linear regression was then performed by data analysis functions in Microsoft Excel software using the hydroarylation data in Fig. 4 except **L7** with no

desired product. Cone angle[23] (θ), buried volumes[24] (%V_{Bur}, sphere radius at 3.5 and 5.0Å) as well as the spin density on the iron center of the styrene-coordinated iron(0)-NHC complex were selected as parameters (P_1, P_2, P_3 and P_4), which are exemplified in Figure S10. The output of the regression results gave the coefficients, constant and *R-square*.

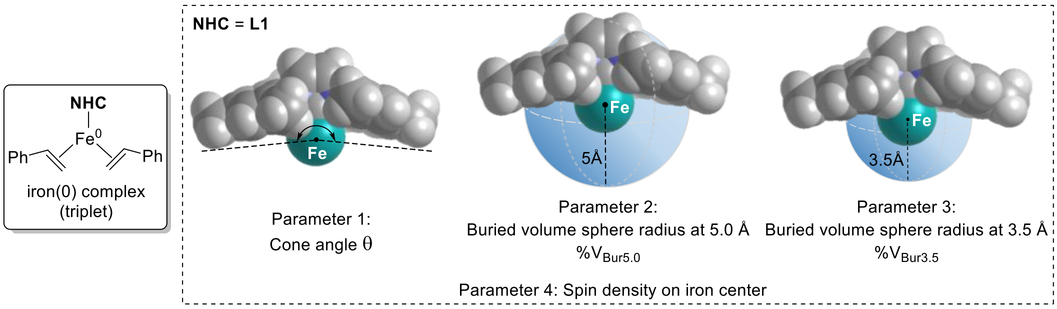

Figure S10. Parameters used in the multivariate linear regression of triplet iron(0) complex taking **L1** as example.

All the parameters were calculated based on DFT-optimized iron(0) complex structures with **L1** to **L15** except **L7**, at the B3LYP-D3(BJ) level of theory. The spin density on the iron center of the triplet complex was extracted from the natural bond orbital (NBO) analysis at the TPSSh-D3(BJ) level of theory.

Table S8. Calculated parameters for triplet iron(0) complex using **L1** to **L15** except **L7**.

Ligand	Cone angle θ	Buried volume %$V_{Bur5.0}$ (5.0 Å)	Buried volume %$V_{Bur3.5}$ (3.5 Å)	Spin density on iron center
**L1**	194.4	37.6	37.6	2.38
**L2**	212.2	49.4	49.4	2.34
**L3**	185.8	38.7	37.6	2.38
**L4**	195.8	39.5	39.5	2.42
**L5**	188.1	39.5	38.9	2.42
**L6**	171.1	28.2	30.7	2.37
**L8**	216.0	42.7	42.7	2.38
**L9**	215.2	36.4	44.1	2.37
**L10**	236.5	46.7	51.1	2.53
**L11**	259.3	41.1	52.0	3.06
**L12**	272.1	54.9	55.4	2.54
**L13**	251.7	50.1	60.5	2.96
**L14**	281.4	52.2	58.2	2.89
**L15**	288.0	52.9	59.2	3.24

The calculated parameters were then normalized using the min-max normalization method with the equation as follows.

$$x_{norm} = \frac{x - \min(x)}{\max(x) - min(x)}$$

The construction of multivariate linear regression model was accomplished by the following Python script. The dependence of the required Python package are:

pandas: 2.1.4;

numpy: 1.24.3;

scikit-learn: 1.3.0;

scipy: 1.11.4;

matplotlib: 3.8.0.

To test the applicability of the multivariate model, we performed leave-one-out (LOO) cross-validation given the small scale of datasize, where each sample was used once as the singleton validation set while the remaining sample formed the training set.

```
import pandas as pd
import numpy as np
from sklearn.model_selection import LeaveOneOut
from sklearn.linear_model import LinearRegression
from scipy.stats import pearsonr
import matplotlib.pyplot as plt

df = pd.read_csv('mvlr-data.csv')

target = df['delta_delta_free_energy'].to_numpy()
cone_angle = df['cone_angle_std'].to_numpy()
bv_5 = df['bv_5_std'].to_numpy()
bv_3 = df['bv_3.5_std'].to_numpy()
spin_iron = df['spin_iron_std'].to_numpy()
all_descriptors = np.column_stack((cone_angle, bv_5, bv_3, spin_iron))

loo = LeaveOneOut()
model = LinearRegression()
Y_true = []
Y_pred = []
for train_index, test_index in loo.split(all_descriptors):
    X_train, X_test = all_descriptors[train_index], all_descriptors[test_index]
    y_train, y_test = target[train_index], target[test_index]
    model.fit(X_train, y_train)
    y_pred = model.predict(X_test)
    Y_true.append(y_test[0])
    Y_pred.append(y_pred[0])

r, p_value = pearsonr(Y_true, Y_pred)
print(f"LOO CV Pearson r: {r:.4f}")

model = LinearRegression().fit(all_descriptors, target)
intercept = model.intercept_
coef = model.coef_
print("ŷ = {:.4f} + {:.4f}·x1 + {:.4f}·x2 + {:.4f}·x3 + {:.4f}·x4"
      .format(intercept, *coef))

Y_true = np.array(Y_true)
Y_pred = np.array(Y_pred)
plt.scatter(Y_true, Y_pred, color='royalblue', edgecolors='black', linewidths=1.0, alpha=0.5)
m, b = np.polyfit(Y_true, Y_pred, 1)
plt.plot(Y_true, m*Y_true + b, color='grey', linewidth=1)
plt.axvline(x=0, color='black', linewidth=1)
plt.axhline(y=0, color='black', linewidth=1)
plt.xlabel('Experimental ΔΔG')
plt.ylabel('Predicted ΔΔG')
plt.show()
```

9.3 The most favorable reaction pathways leading to competing linear product and branched products

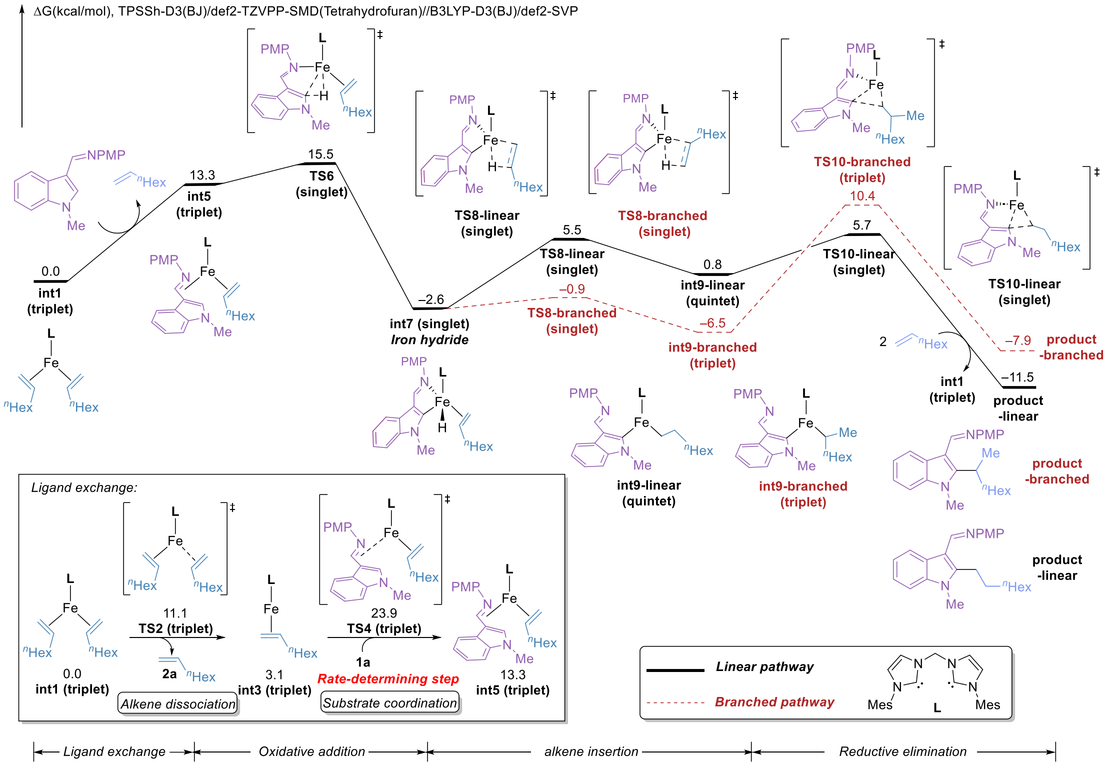

Figure S11. DFT-computed reaction pathways of iron-catalyzed hydroarylation with bis-NHC ligand starting from triplet iron(0) complex **int1**.

The olefin dissociation from **int1** is reversible *via* transition state **TS2**, generating a single olefin coordinated iron(0) species **int3**. However, the substrate coordination from **int3** requires a barrier of 23.9 kcal/mol *via* transition state **TS4**, which is most likely the rate-determining step of the catalytic cycle. The subsequent C–H activation occurs through oxidation addition, followed by a facile migratory insertion. Although, the secondary alkyl iron(II) intermediate **int9-branched** is more stable than primary alkyl iron(II) intermediate **int9-linear**, the reductive elimination from **int9-branched** is higher than **int9-linear** by 4.7 kcal/mol. Therefore, the reductive elimination is probably the regioselectivity-determining step.

9.4 Competing reaction pathways leading to linear product 3

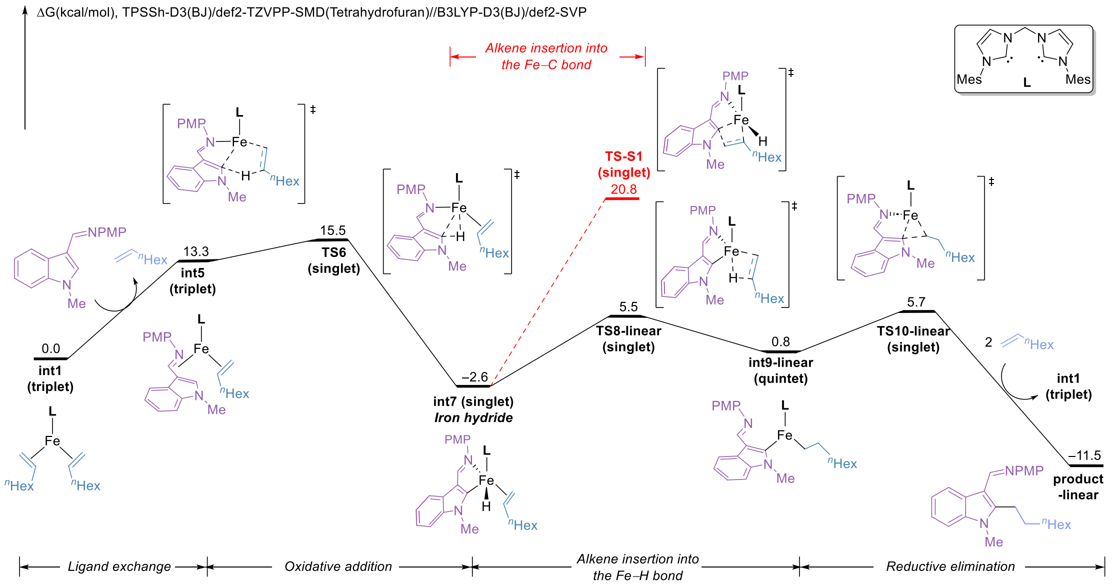

Figure S12. Competing reaction pathways of iron-catalyzed hydroarylation with bis-NHC ligand leading to linear product **3**.

The competing alkene insertion into the Fe–C bond is unfavorable by 15.3 kcal/mol *via* transition state **TS-S1**, compared with olefin insertion into the Fe–H bond through transition state **TS8-linear**.

9.5 Table of energy

Table S9. Zero-point correction (*ZPE*), thermal correction to enthalpy (*TCH*), thermal correction to Gibbs free energy (*TCG*), energies (*E*), enthalpies (*H*), and Gibbs free energies (*G*) (in Hartree) of the structures calculated at the TPSSh-D3(BJ)/def2-TZVP-SMD(Tetrahydrofuran)//B3LYP-D3(BJ)/def2-SVP level of theory.

Structures	*ZPE*	*TCH*	*TCG*	*E*	*H*	*G*	Imaginary Frequency
int1 (singlet)	0.92899	0.84017	0.84017	–3082.2191	–3081.3789	–3081.3789	
int1 (triplet)	0.92529	0.97894	0.83106	–3082.2478	–3081.2689	–3081.4168	
TS2 (singlet)	0.92430	0.83501	0.83501	–3082.2055	–3081.3705	–3081.3705	147.97*i*
TS2 (triplet)	0.92224	0.97608	0.82638	–3082.2255	–3081.2494	–3081.3991	66.05*i*
int3 (singlet)	0.70100	0.74203	0.62679	–2767.5495	–2766.8074	–2766.9227	
int3 (triplet)	0.69978	0.74182	0.62100	–2767.5765	–2766.8346	–2766.9555	

TS4 (triplet)	0.99507	1.05468	0.89770	–3610.1624	–3609.1077	–3609.2647	119.14*i*
int5 (singlet)	0.99847	1.05786	0.90154	–3610.1765	–3609.1187	–3609.2750	
int5 (triplet)	0.99634	1.05637	0.89936	–3610.1839	–3609.1275	–3609.2845	
TS6 (singlet)	0.99813	1.05677	0.90329	–3610.1843	–3609.1275	–3609.2810	658.04*i*
TS6 (triplet)	0.99383	1.05315	0.89836	–3610.1734	–3609.1202	–3609.2750	554.23*i*
TS6 (quintet)	0.98759	1.04869	0.88705	–3610.1614	–3609.1127	–3609.2743	362.99*i*
int7 (singlet)	0.99695	1.05570	0.90201	–3610.2119	–3609.1562	–3609.3099	
int7 (triplet)	0.99319	1.05310	0.89387	–3610.1745	–3609.1214	–3609.2806	
int7 (quintet)	0.99095	1.05224	0.88986	–3610.1817	–3609.1294	–3609.2918	
TS8-linear (singlet)	0.99652	0.90371	0.90371	–3610.2007	–3609.2970	–3609.2970	586.46*i*
TS8-linear (triplet)	0.99252	0.89343	0.89343	–3610.1699	–3609.2764	–3609.2764	468.79*i*
TS8-linear (quintet)	0.98942	1.04985	0.88930	–3610.1369	–3609.0870	–3609.2476	669.87*i*
TS8-branched (singlet)	0.99586	1.05426	0.90129	–3610.2085	–3609.1543	–3609.3072	552.49*i*
TS8-branched (triplet)	0.99227	1.05190	0.89322	–3610.1713	–3609.1194	–3609.2781	601.80*i*
TS8-branched (quintet)	0.98832	1.04837	0.88827	–3610.1405	–3609.0921	–3609.2522	765.95*i*
int9-linear (singlet)	1.00211	1.06090	0.90724	–3610.2010	–3609.1401	–3609.2937	
int9-linear (triplet)	0.99802	1.05769	0.90050	–3610.1917	–3609.1340	–3609.2912	
int9-linear (quintet)	0.99598	0.89404	0.89404	–3610.1985	–3609.3045	–3609.3045	
int9-branched (singlet)	1.00053	1.05944	0.90682	–3610.2094	–3609.1500	–3609.3026	
int9-branched (triplet)	0.99716	1.05727	0.89846	–3610.2146	–3609.1574	–3609.3162	
int9-branched (quintet)	0.99624	1.05669	0.89553	–3610.1945	–3609.1378	–3609.2989	
TS10-linear (singlet)	0.99871	1.05668	0.90580	–3610.2025	–3609.1458	–3609.2967	394.22*i*

TS10-linear (triplet)	0.99706	1.05597	0.90046	–3610.1937	–3609.1377	–3609.2932	424.45*i*
TS10-linear (quintet)	0.99481	0.89464	0.89464	–3610.1695	–3609.2748	–3609.2748	255.55*i*
TS10-branched (singlet)	0.99900	1.05663	0.90813	–3610.1962	–3609.1395	–3609.2880	310.28*i*
TS10-branched (striplet)	0.99711	1.05582	0.90257	–3610.1917	–3609.1359	–3609.2892	350.22*i*
TS10-branched (quintet)	0.99486	1.05432	0.89865	–3610.1693	–3609.1149	–3609.2706	146.64*i*
TS-S1 (singlet)	0.99670	1.05425	0.90368	–3610.1763	–3609.1221	–3609.2726	141.53*i*
TS-S1 (triplet)	0.99240	1.05136	0.89585	–3610.1581	–3609.1067	–3609.2622	371.43*i*
TS-S1 (quintet)	0.98872	1.04882	0.88875	–3610.1265	–3609.0776	–3609.2377	454.06
product-linear	0.51940	0.54871	0.45569	–1157.2818	–1156.7330	–1156.8261	
product-branched	0.51927	0.54867	0.45635	–1157.2767	–1156.7280	–1156.8203	
1a	0.29296	0.31105	0.24650	–842.5949	–842.2838	–842.3484	
2a	0.22150	0.23298	0.18496	–314.6443	–314.4113	–314.4594	

10. NMR spectra

^{1}H NMR spectrum of **L8** (300 MHz, DMSO-d_6)

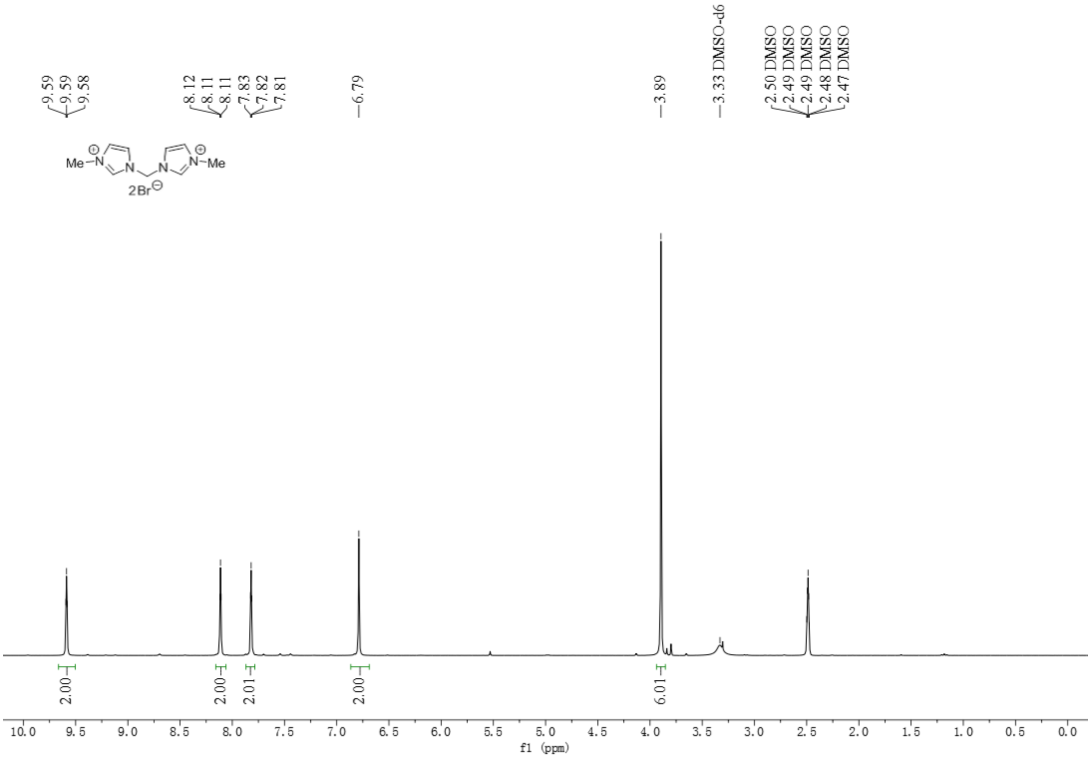

^{13}C NMR spectrum of **L8** (75 MHz, DMSO-d_6)

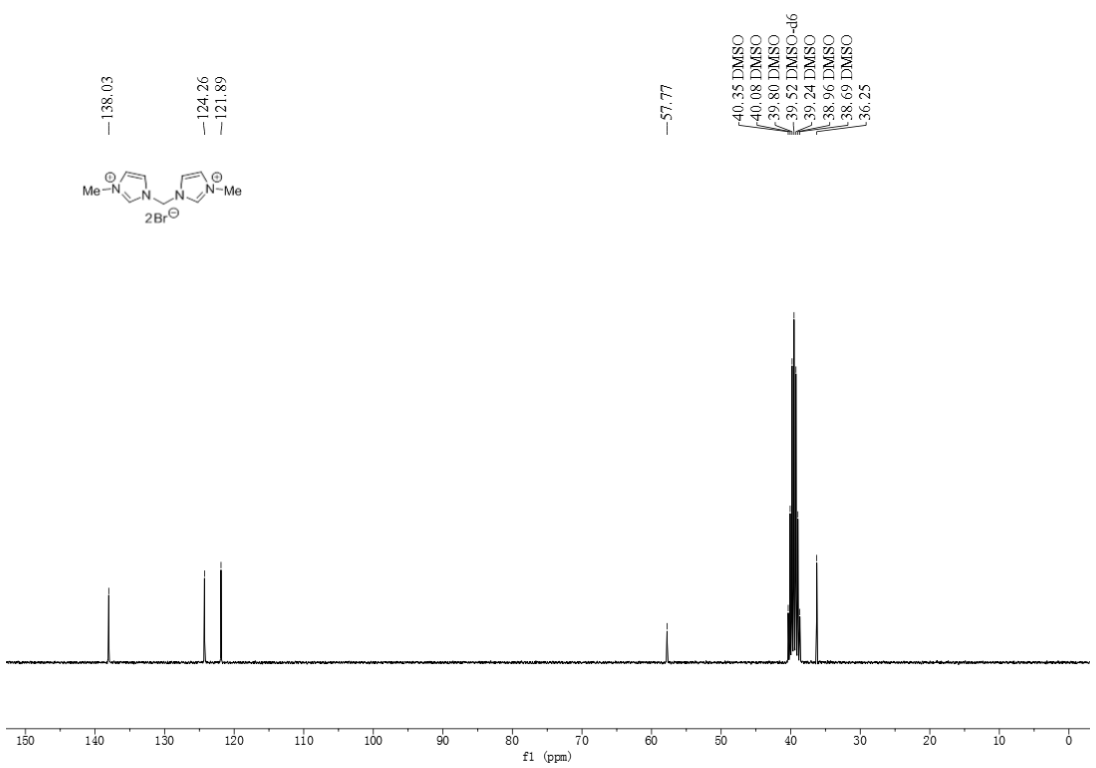

^{1}H NMR spectrum of **L9** (300 MHz, DMSO-d_6)

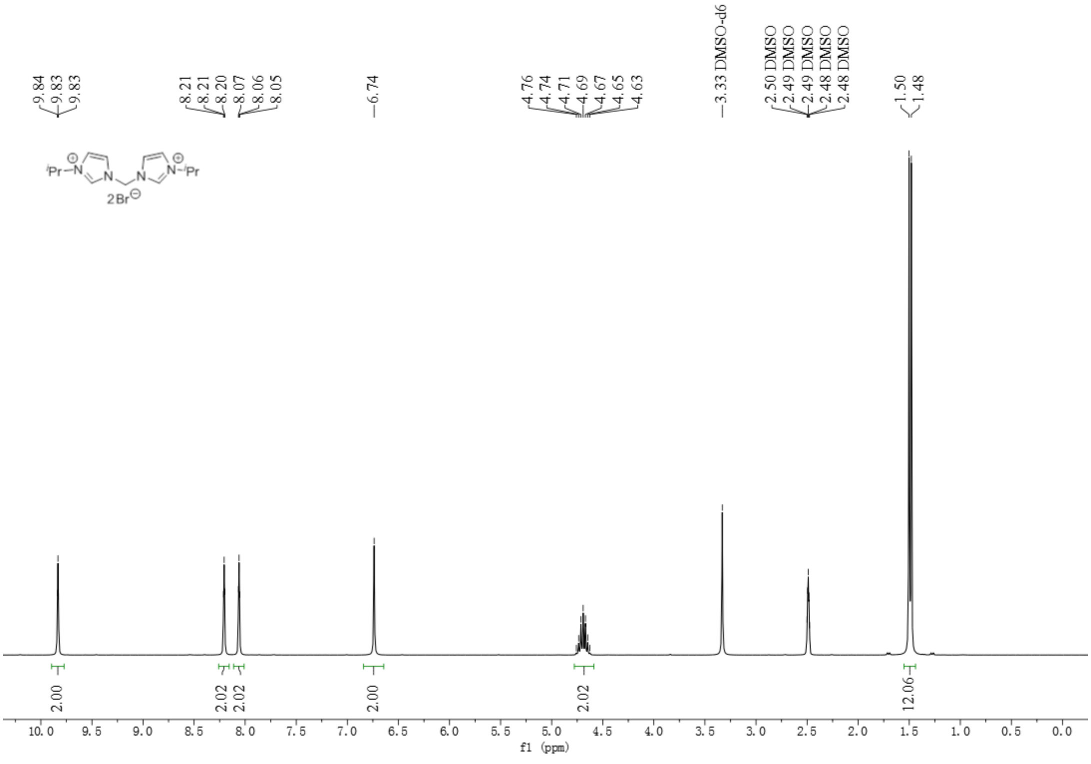

^{13}C NMR spectrum of **L9** (75 MHz, DMSO-d_6)

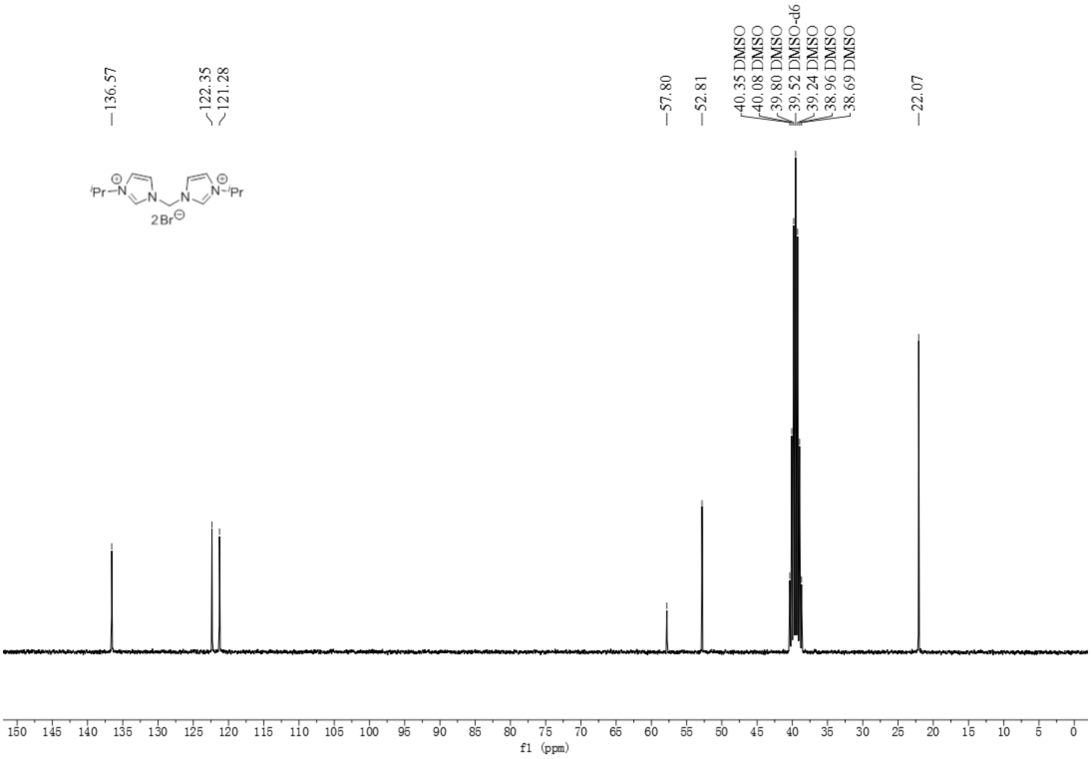

^{1}H NMR spectrum of **L10** (300 MHz, $CDCl_3$)

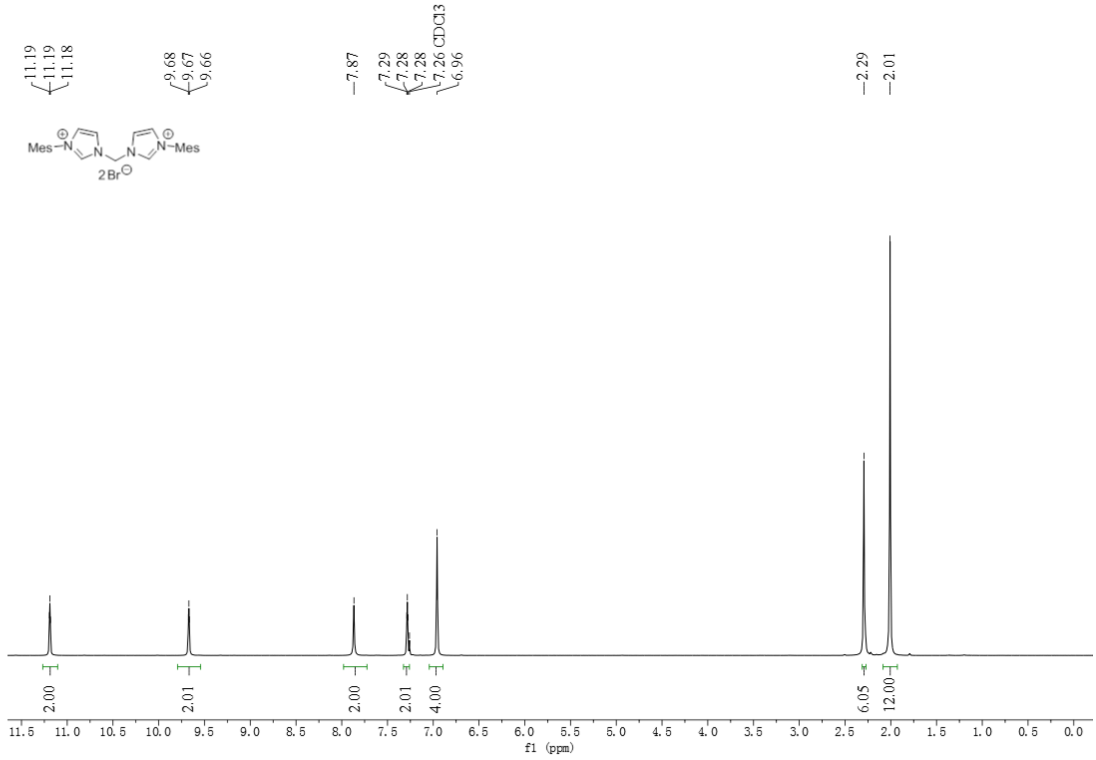

^{13}C NMR spectrum of **L10** (75 MHz, $CDCl_3$)

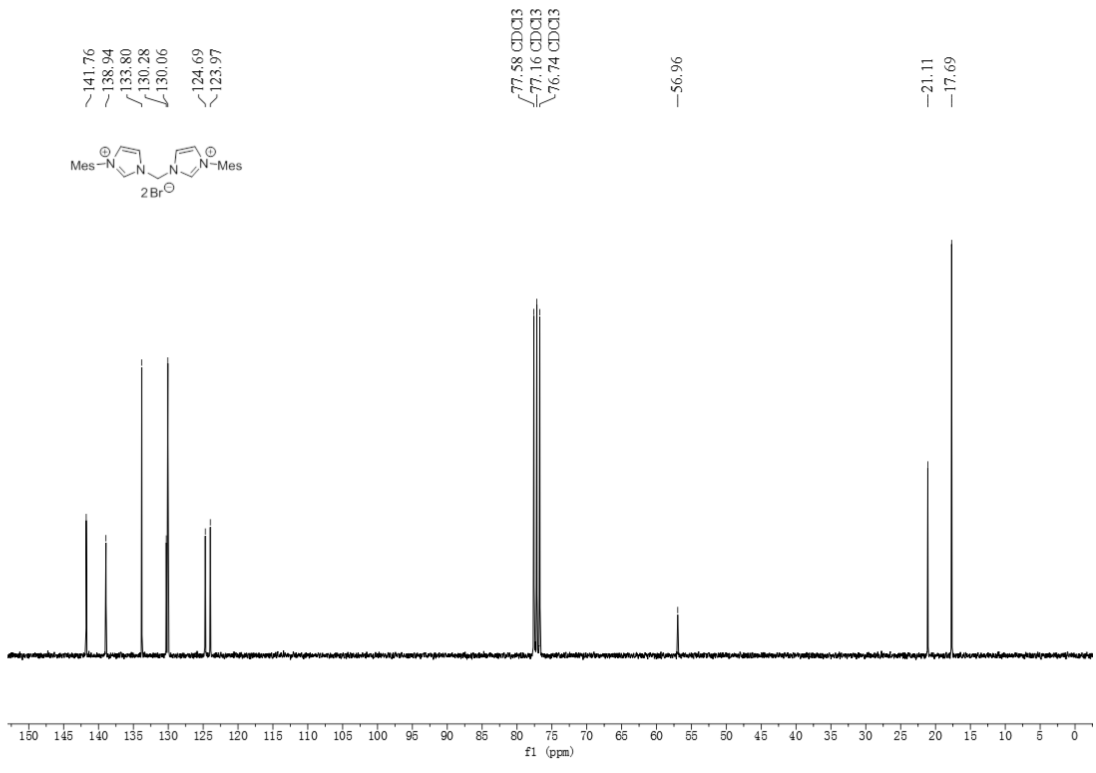

^{1}H NMR spectrum of **L11** (300 MHz, DMSO-d_6)

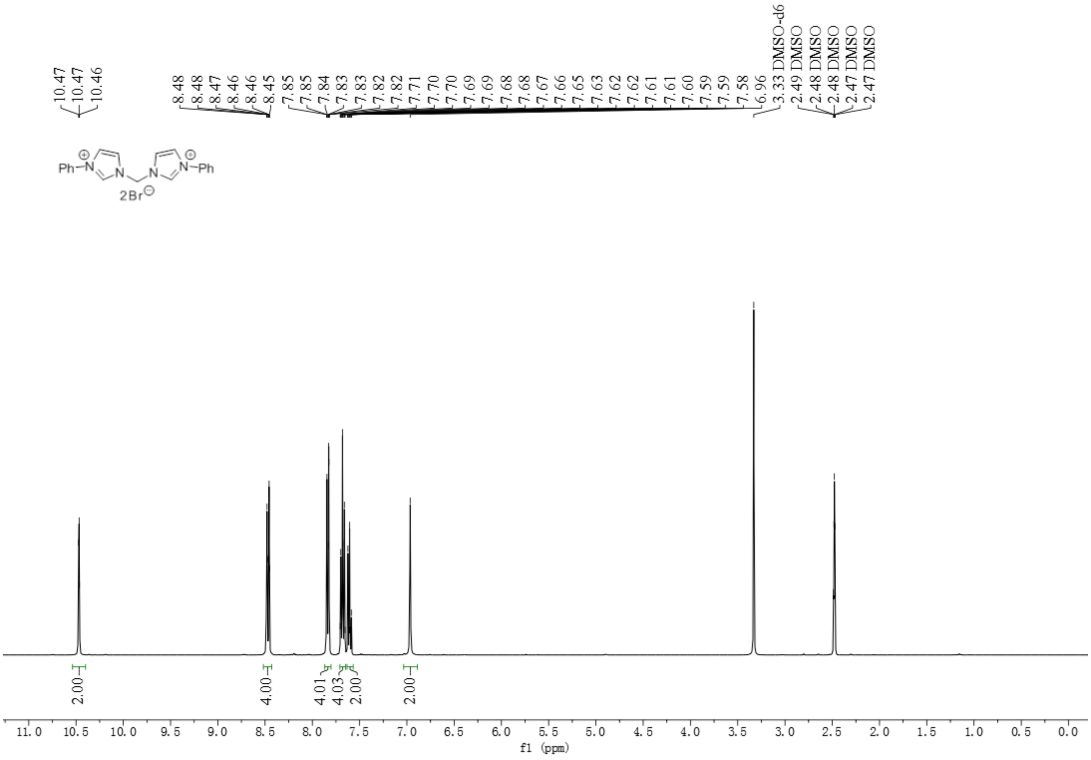

^{13}C NMR spectrum of **L11** (75 MHz, DMSO-d_6)

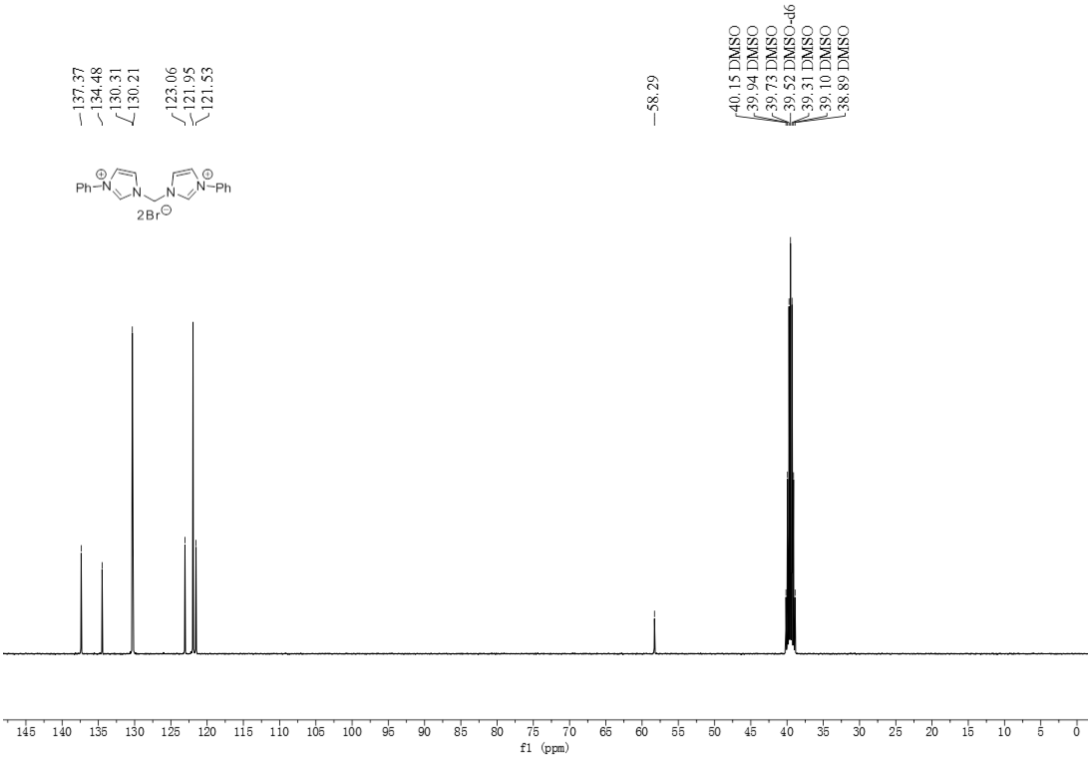

^{1}H NMR spectrum of **L12** (300 MHz, DMSO-d_6)

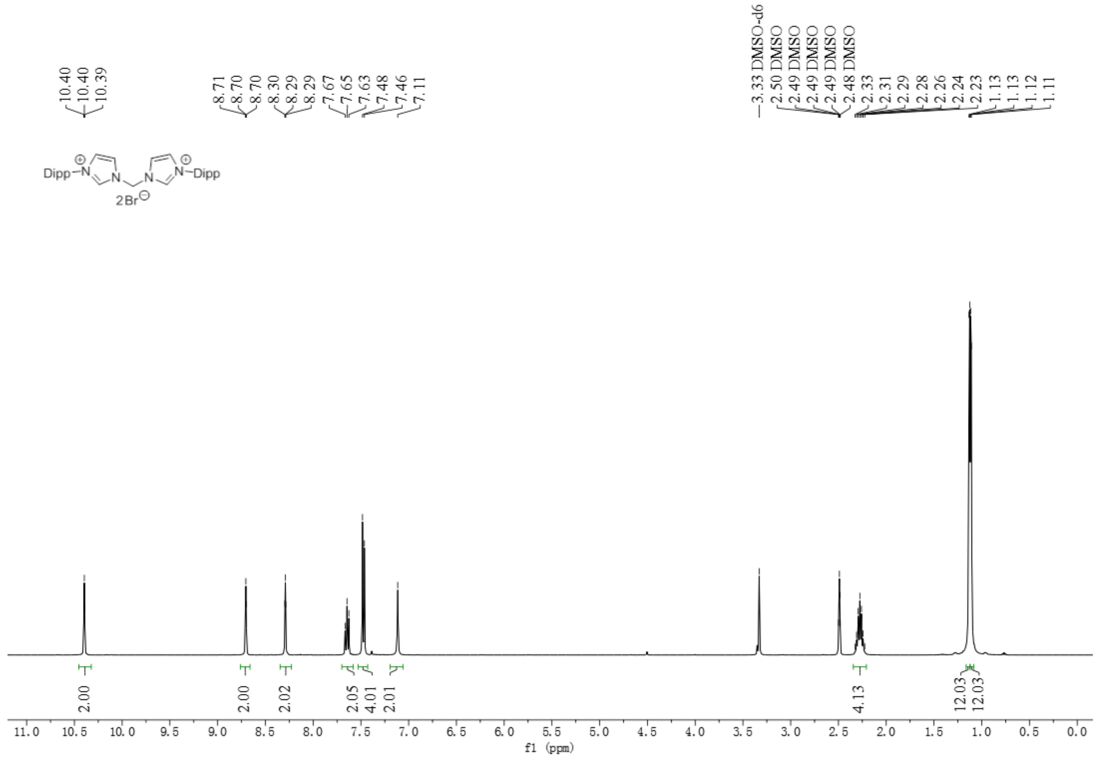

^{13}C NMR spectrum of **L12** (75 MHz, DMSO-d_6)

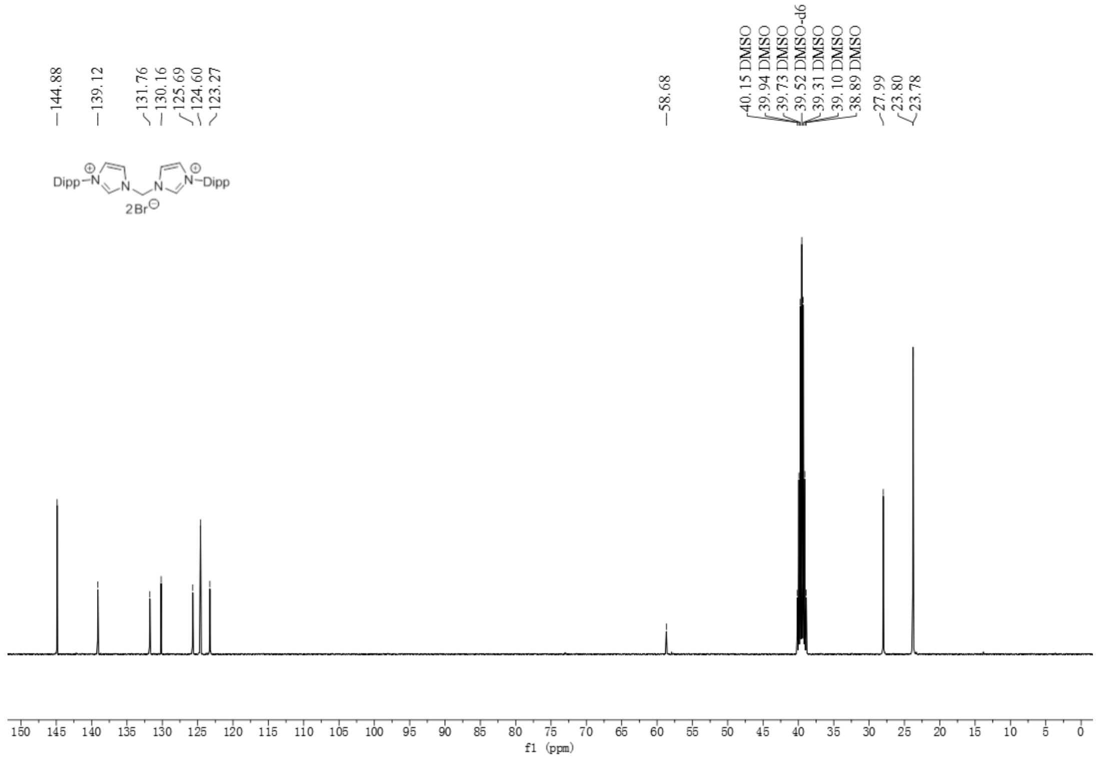

^{1}H NMR spectrum of **L13** (300 MHz, DMSO-d_6)

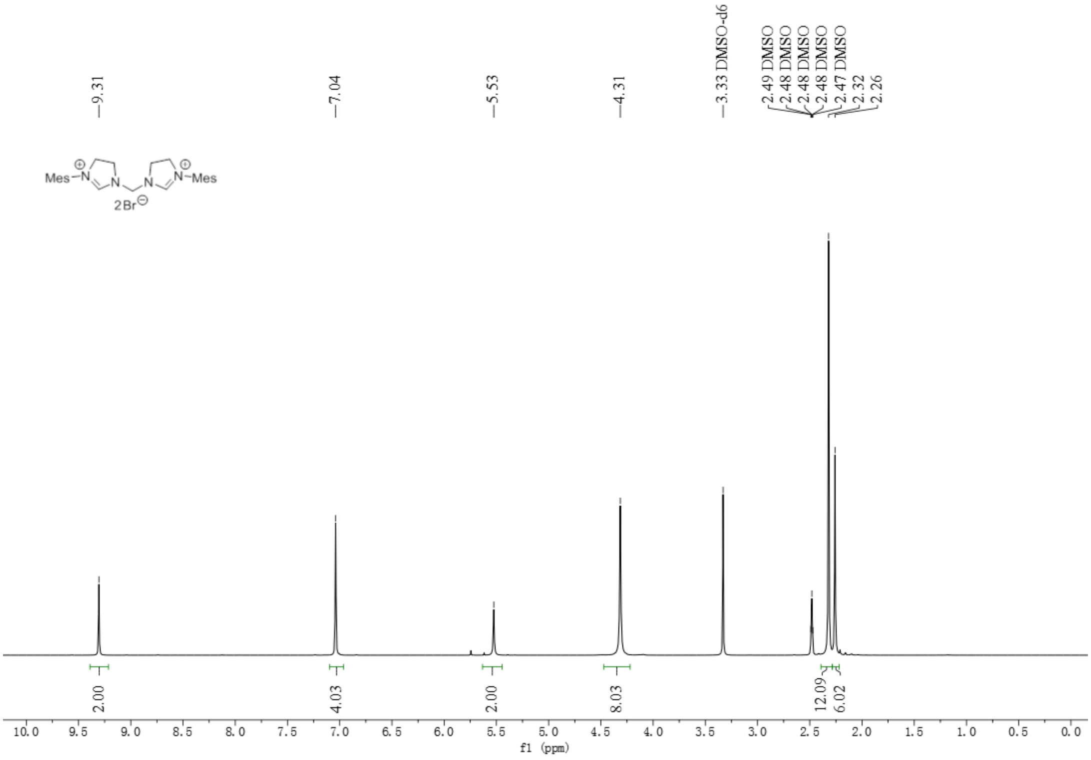

^{13}C NMR spectrum of **L13** (75 MHz, DMSO-d_6)

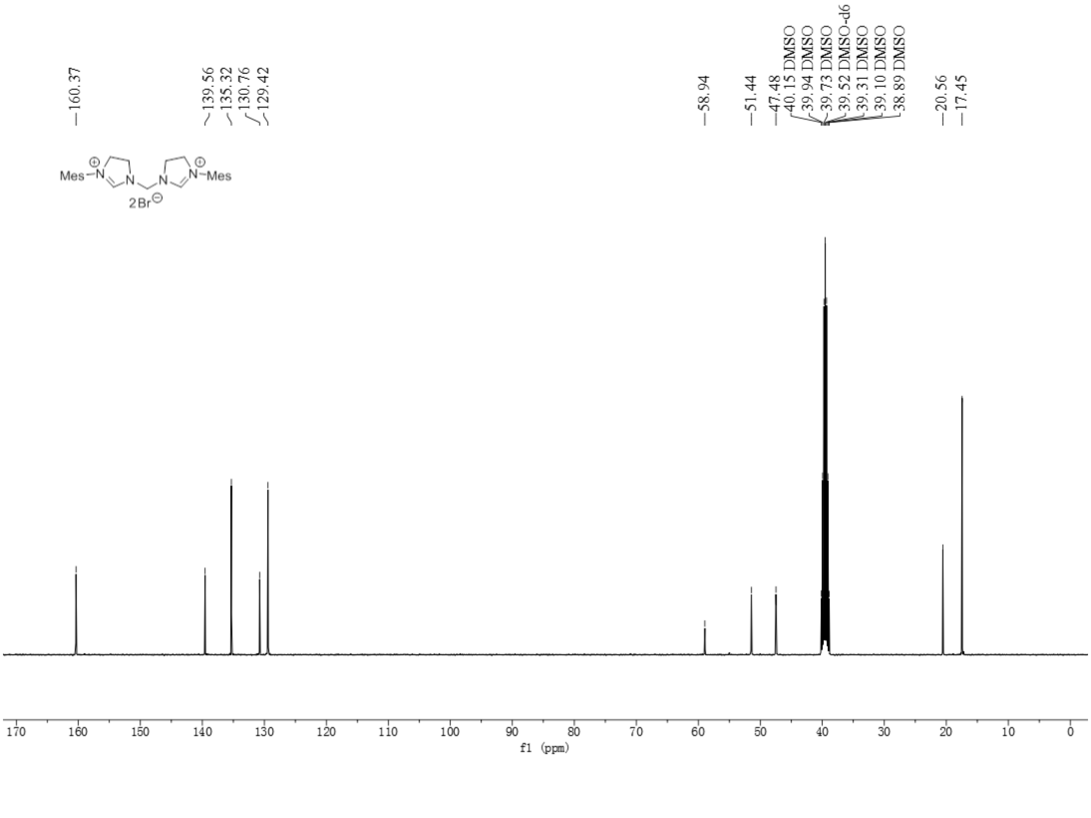

^{1}H NMR spectrum of **L14** (300 MHz, $CDCl_3$)

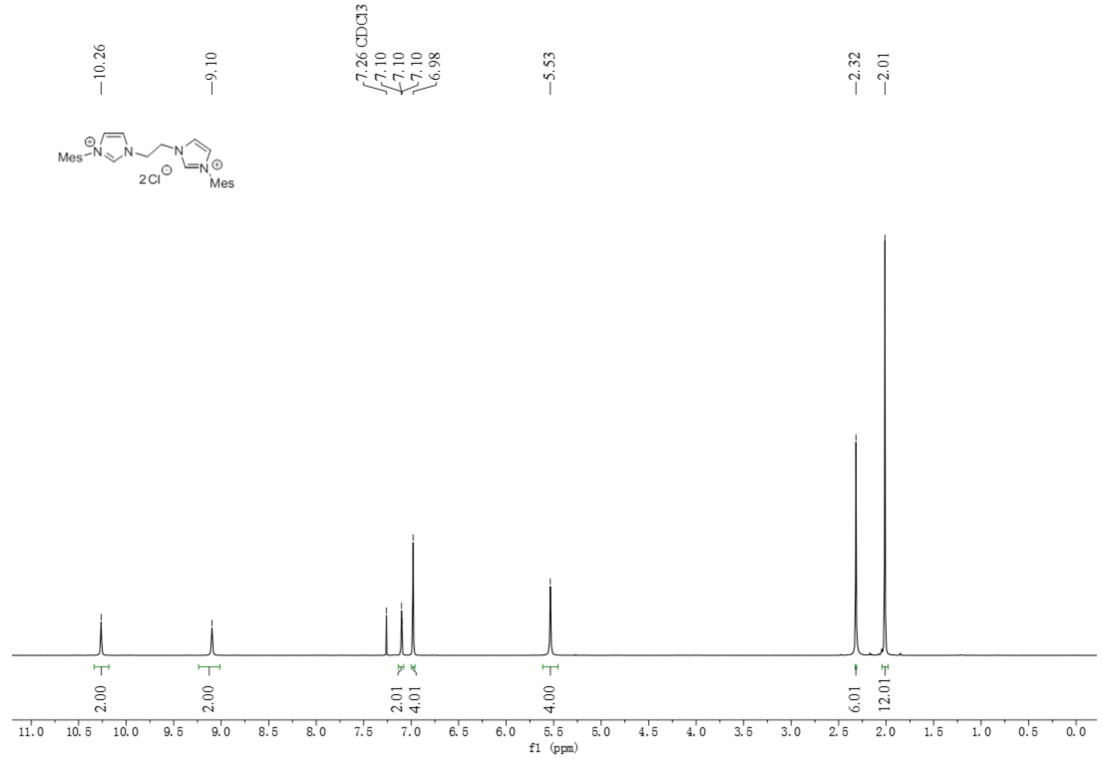

^{13}C NMR spectrum of **L14** (75 MHz, $CDCl_3$)

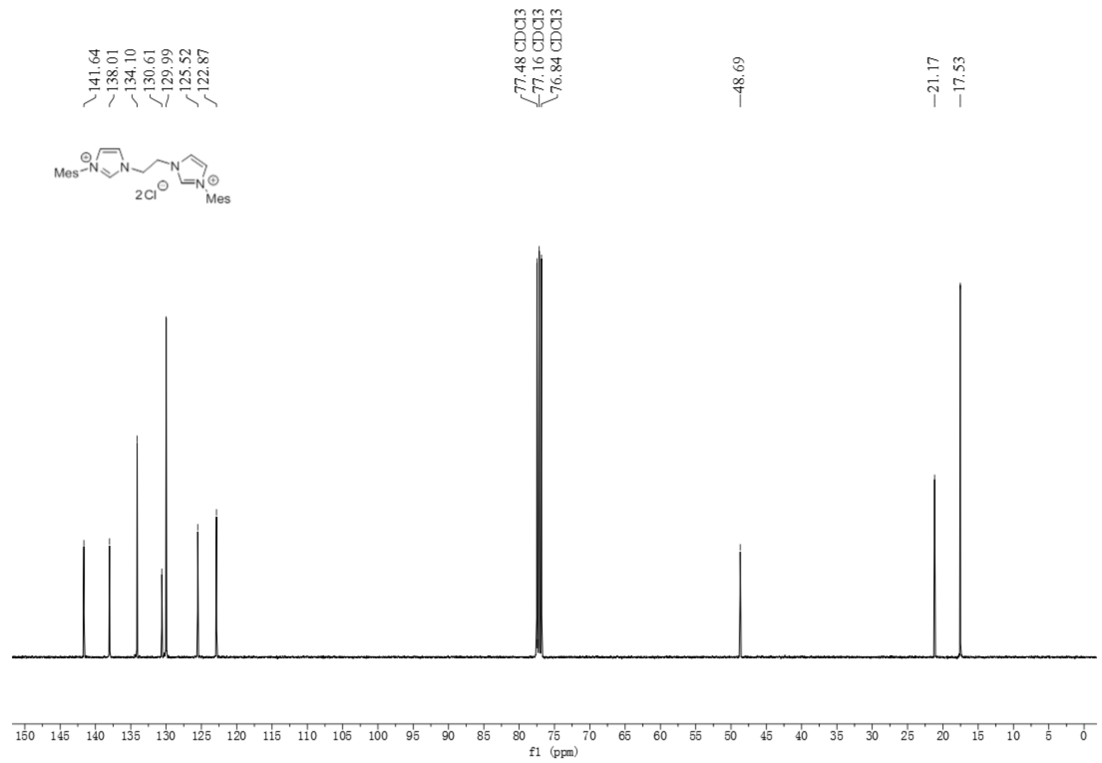

^{1}H NMR spectrum of **L15** (300 MHz, $CDCl_3$)

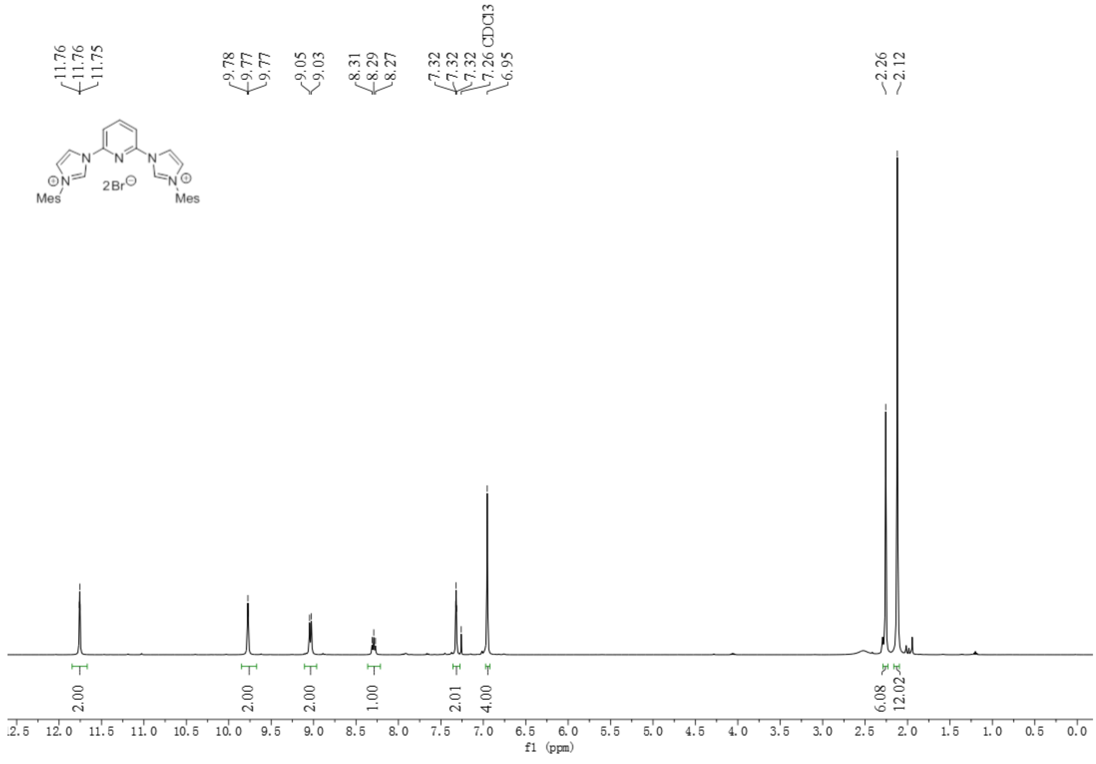

^{13}C NMR spectrum of **L15** (75 MHz, $CDCl_3$)

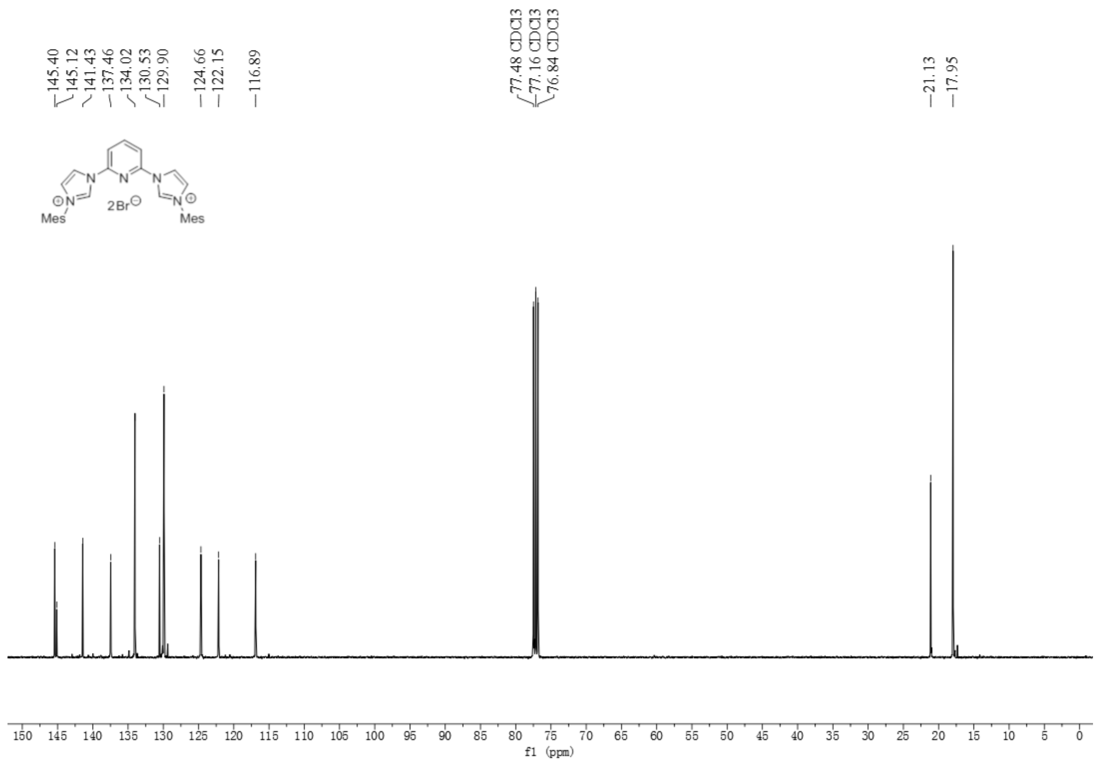

^{1}H NMR spectrum of **3** (300 MHz, $CDCl_3$)

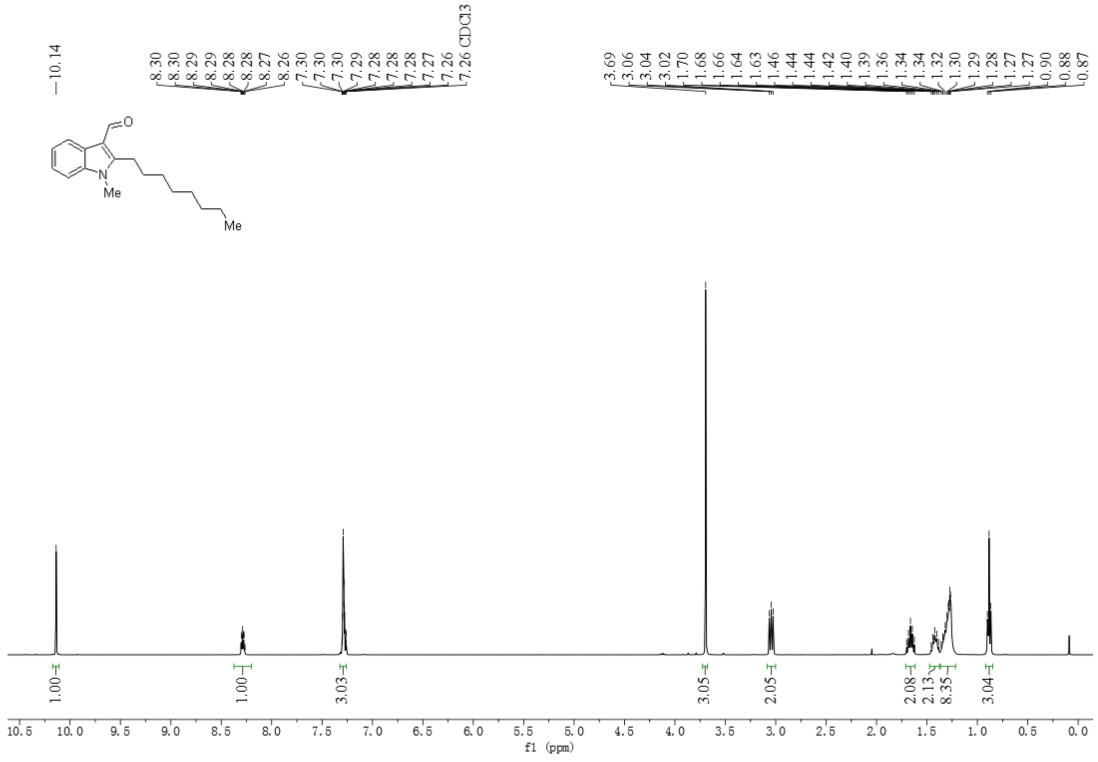

^{13}C NMR spectrum of **3** (75 MHz, $CDCl_3$)

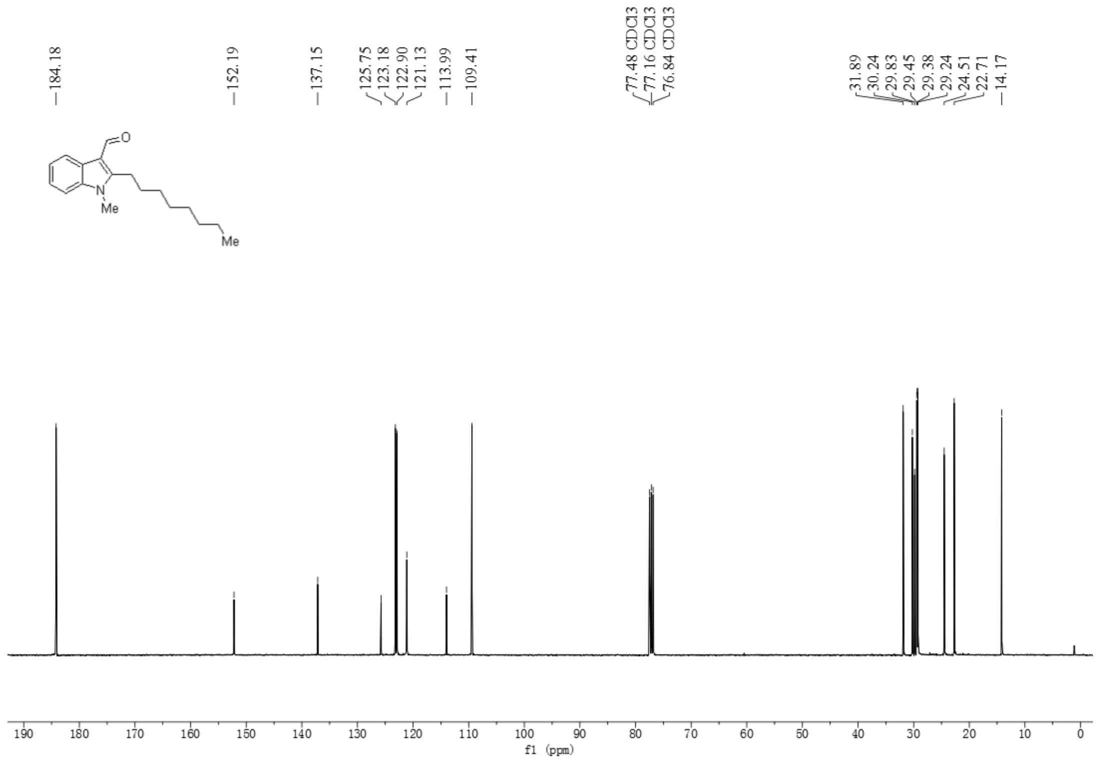

^{1}H NMR spectrum of **4** (300 MHz, $CDCl_3$)

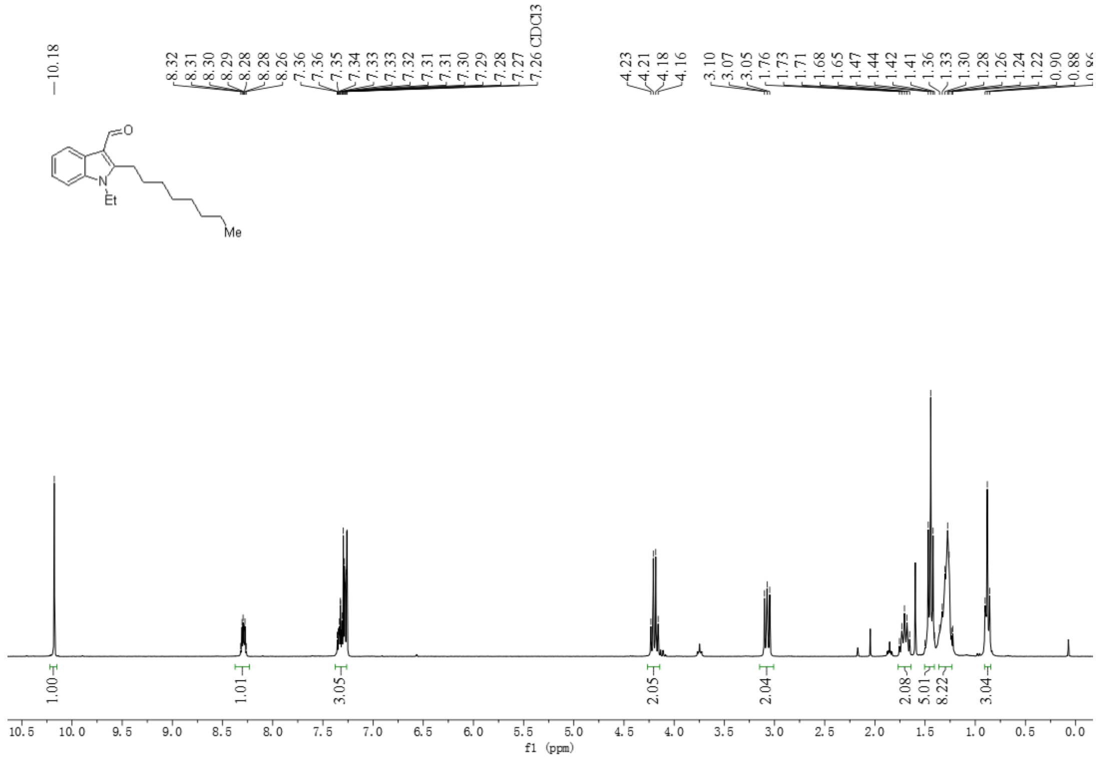

^{13}C NMR spectrum of **4** (75 MHz, $CDCl_3$)

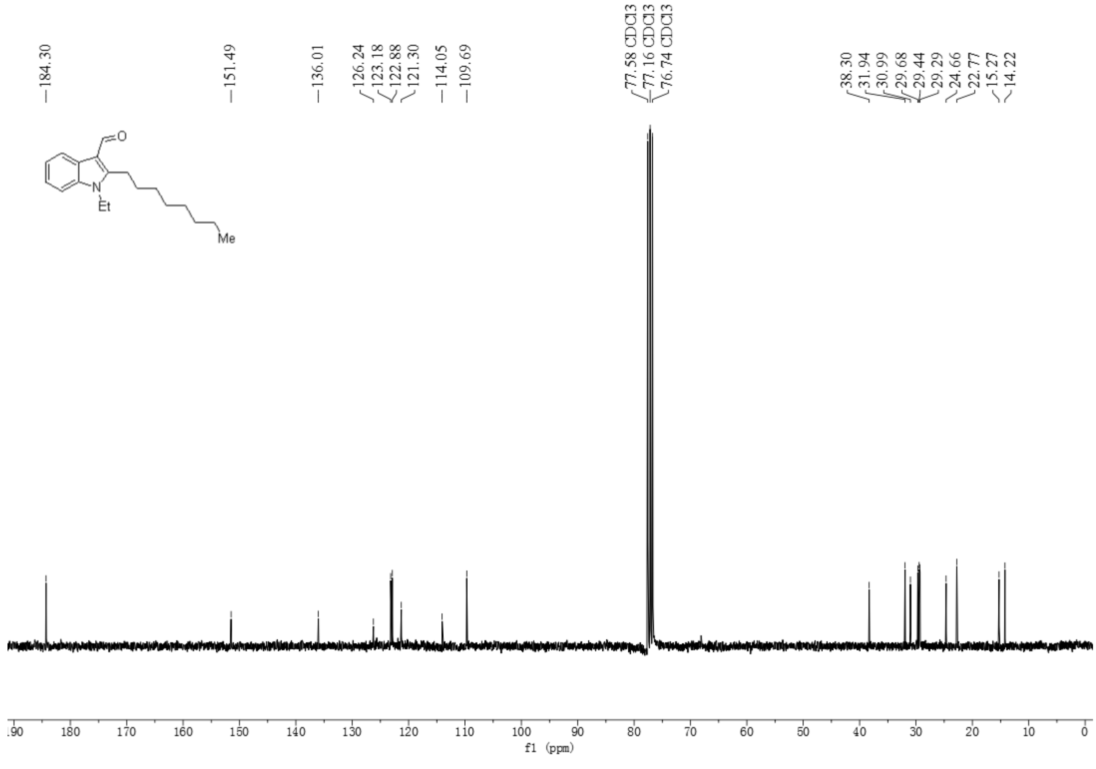

^{1}H NMR spectrum of **5** (300 MHz, $CDCl_3$)

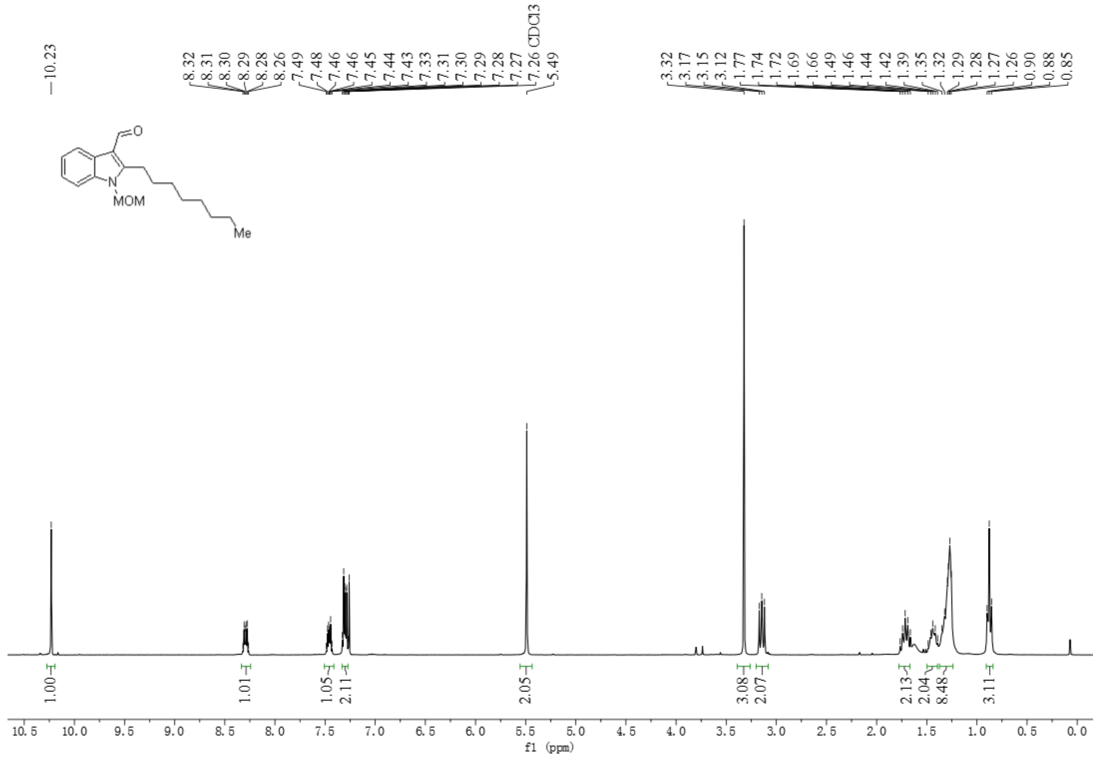

^{13}C NMR spectrum of **5** (75 MHz, $CDCl_3$)

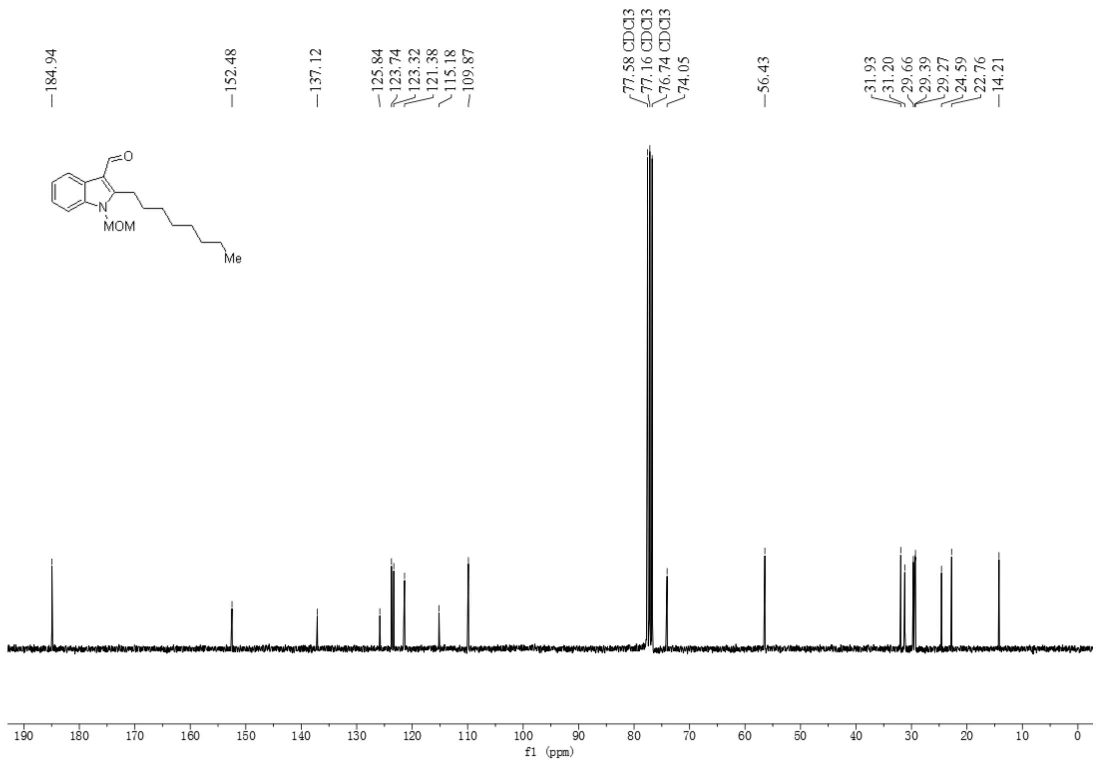

^{1}H NMR spectrum of **6** (300 MHz, $CDCl_3$)

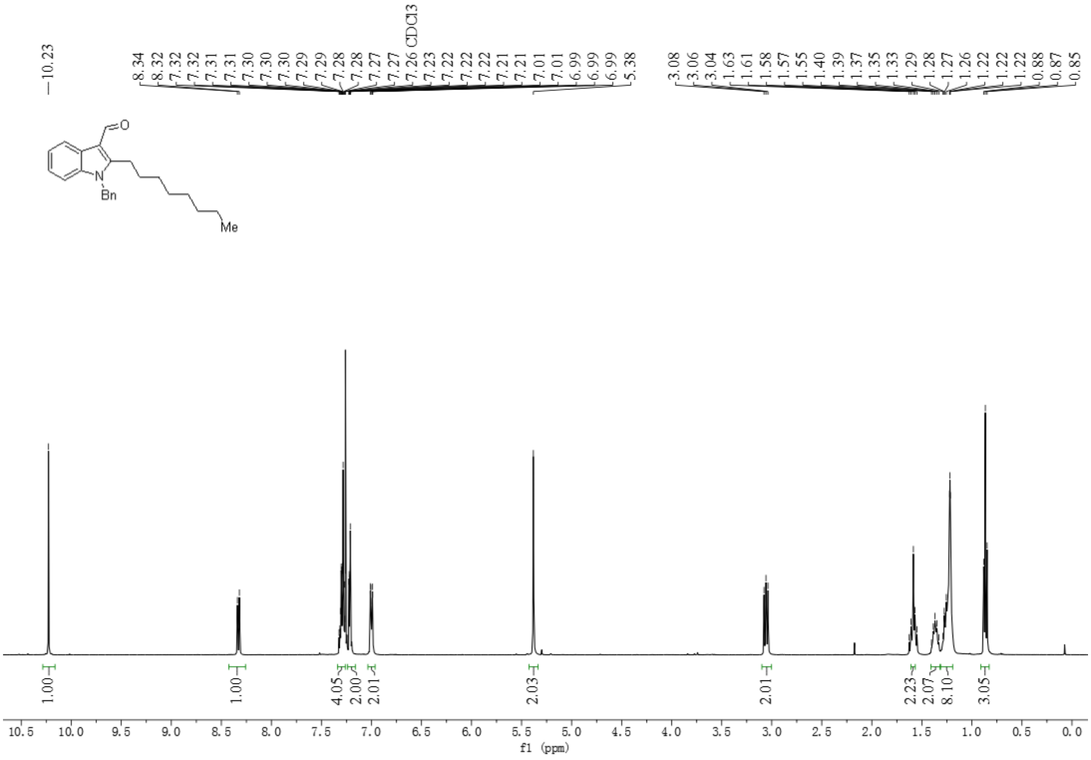

^{13}C NMR spectrum of **6** (75 MHz, $CDCl_3$)

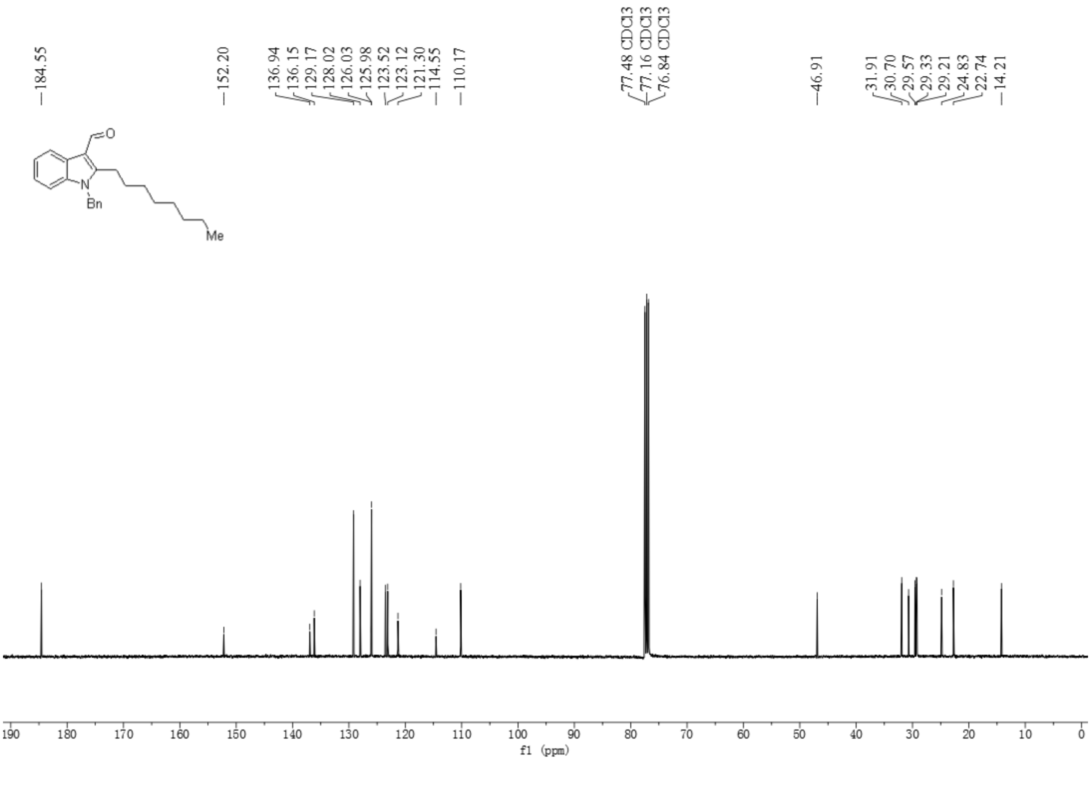

^{1}H NMR spectrum of **7** (300 MHz, $CDCl_3$)

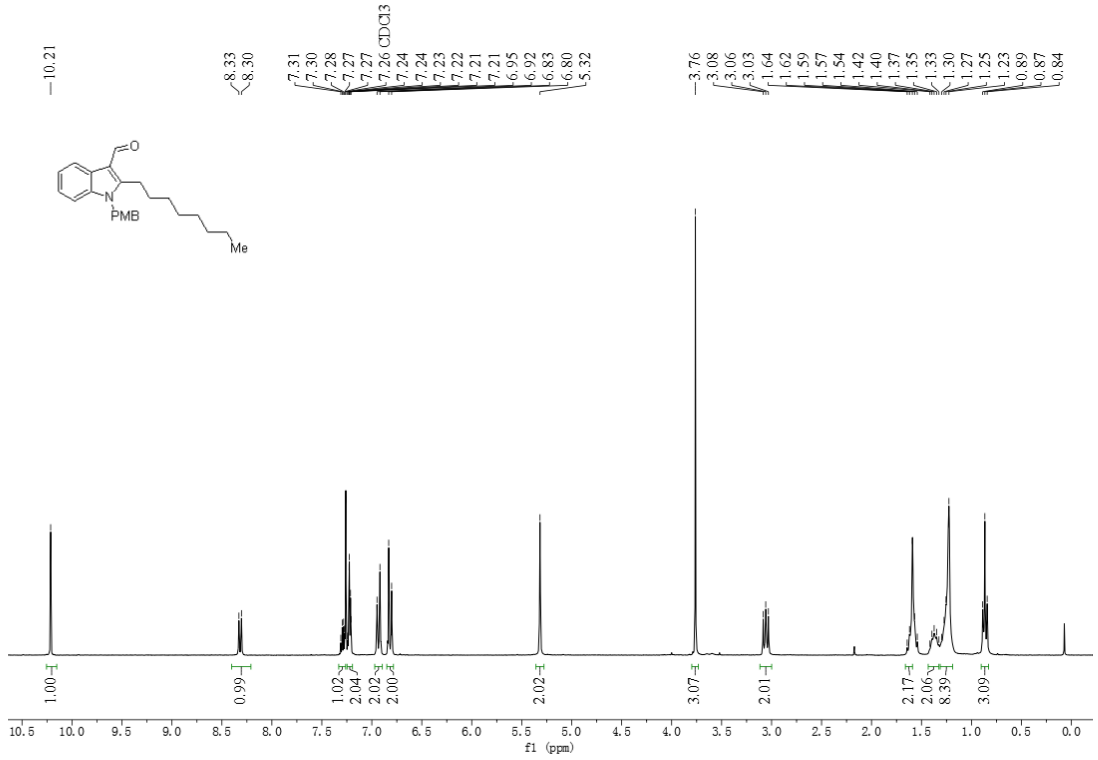

^{13}C NMR spectrum of **7** (75 MHz, $CDCl_3$)

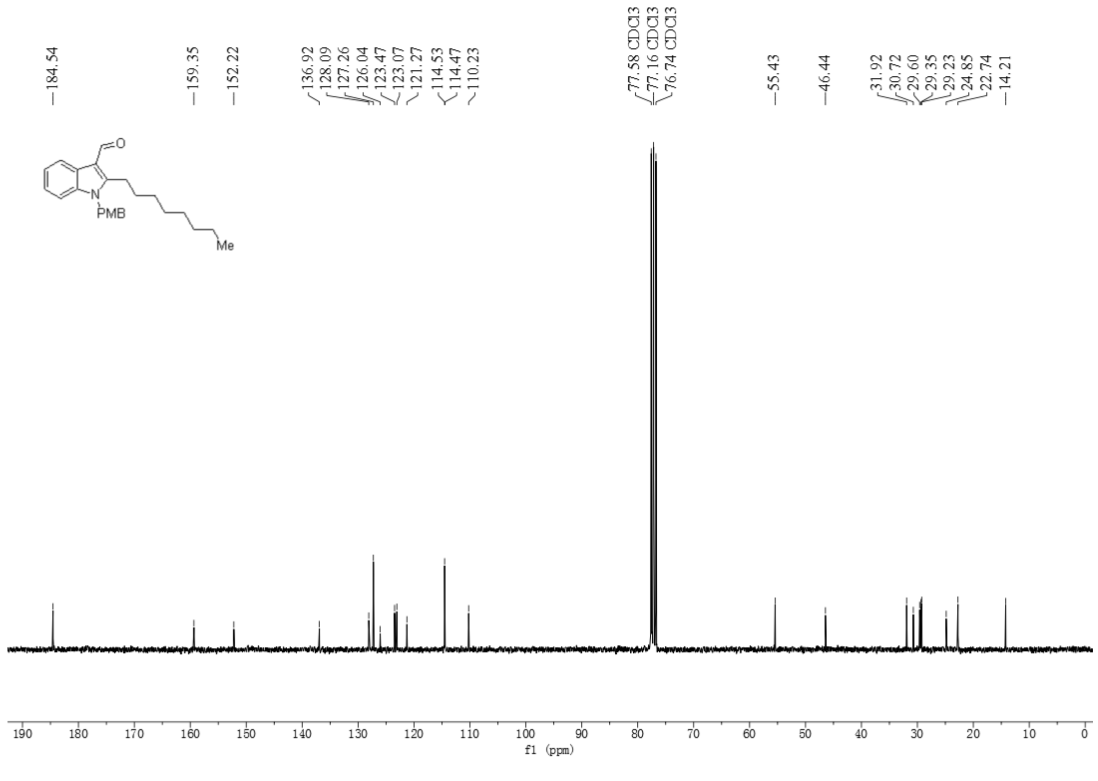

^{1}H NMR spectrum of **8** (300 MHz, $CDCl_3$)

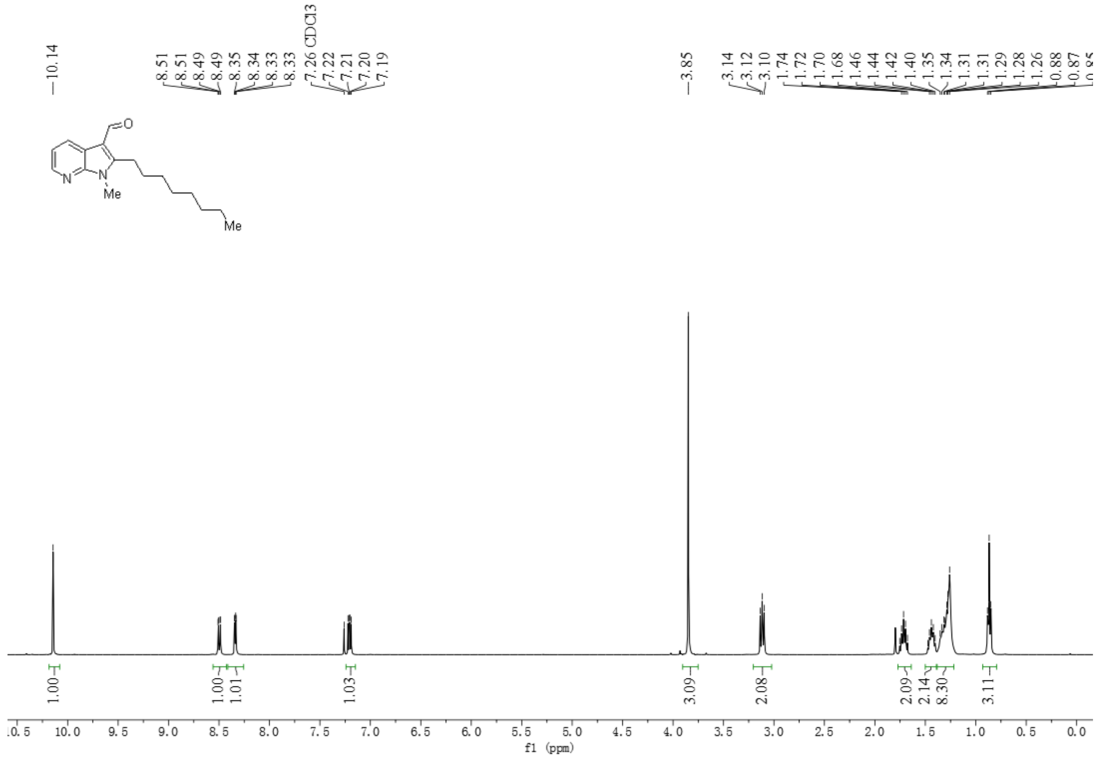

^{13}C NMR spectrum of **8** (75 MHz, $CDCl_3$)

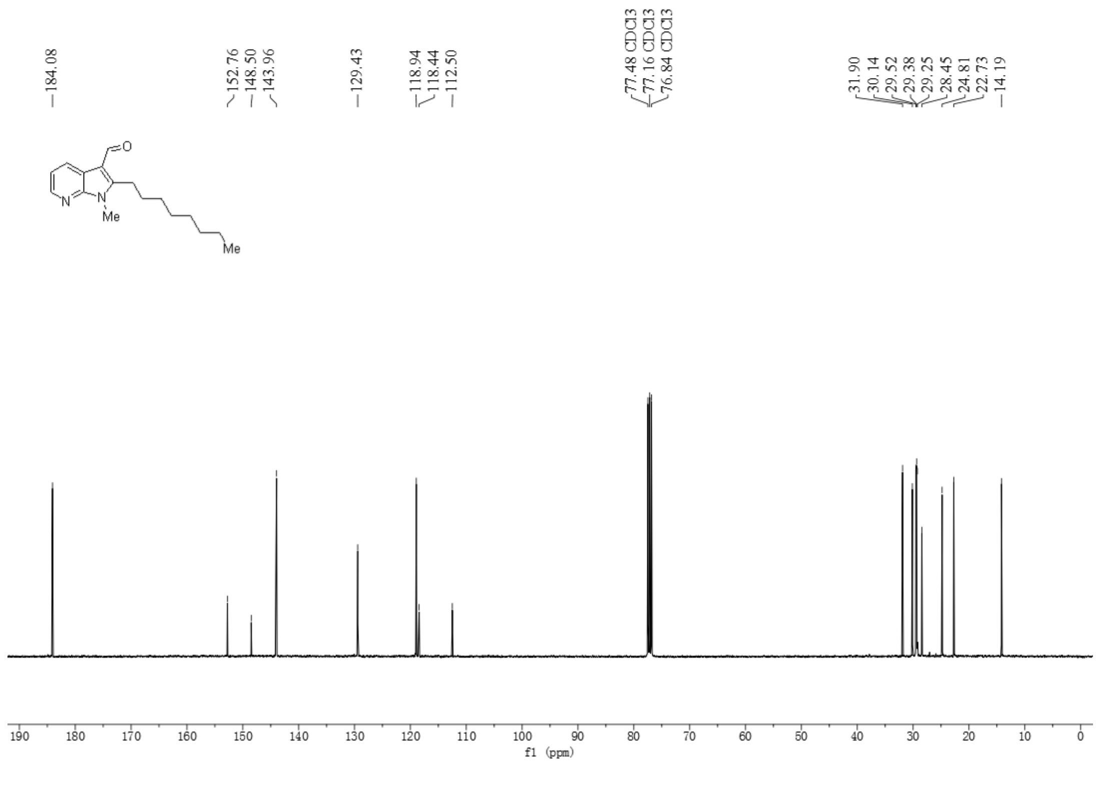

^{1}H NMR spectrum of **9** (300 MHz, $CDCl_3$)

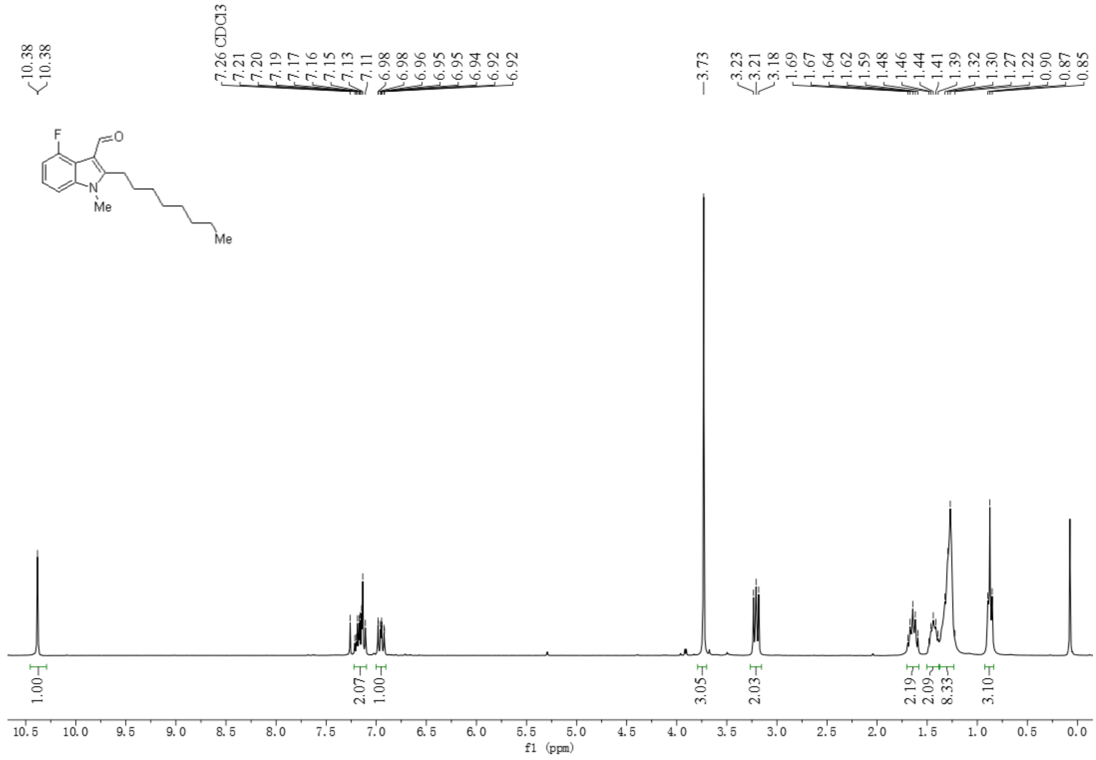

^{13}C NMR spectrum of **9** (75 MHz, $CDCl_3$)

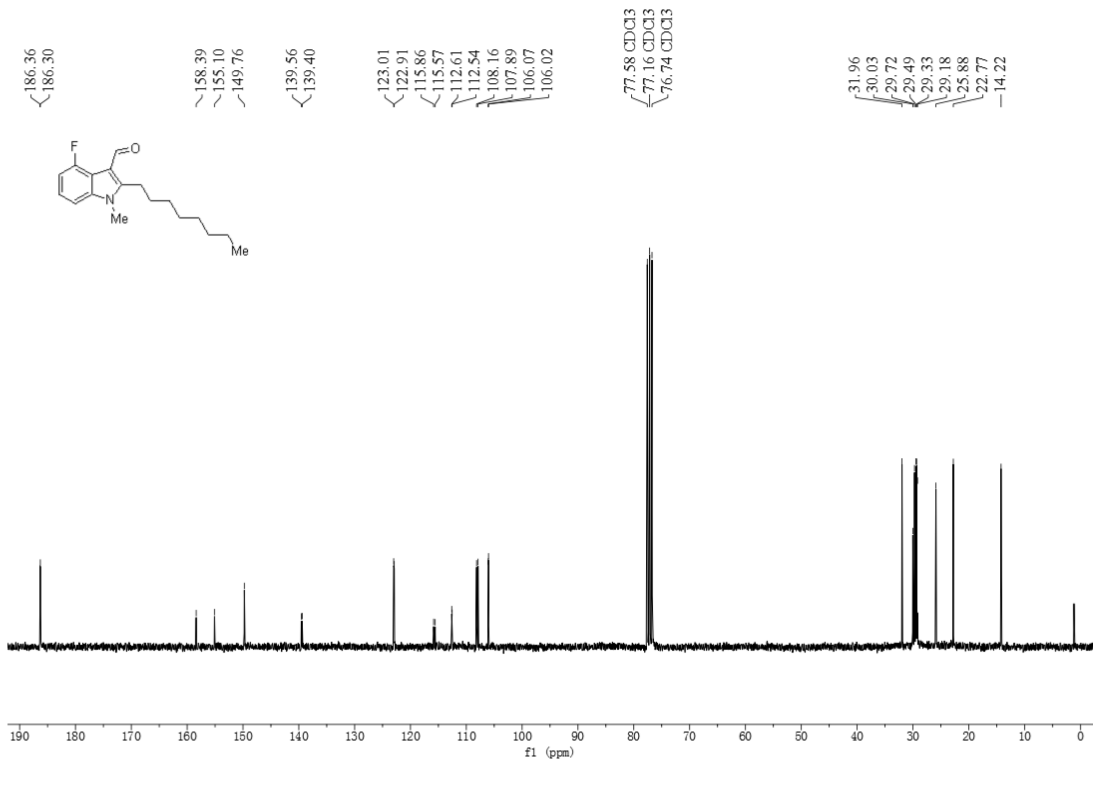

^{19}F NMR spectrum of **9** (282 MHz, $CDCl_3$)

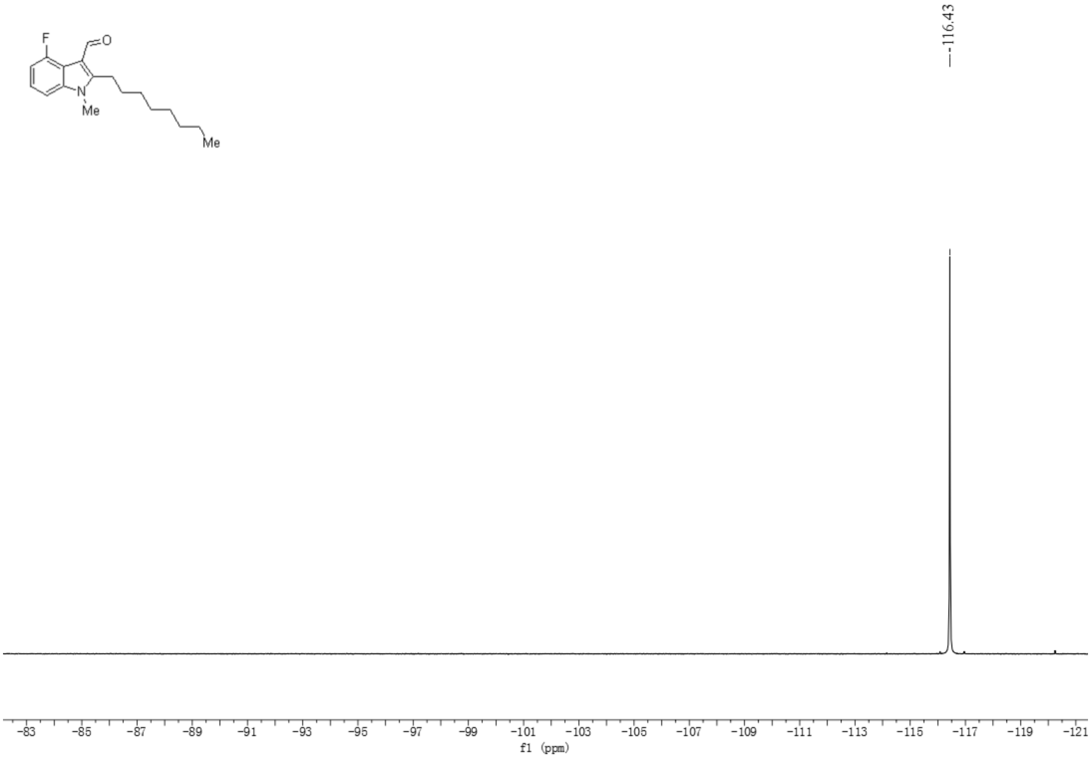

^{1}H NMR spectrum of **10** (300 MHz, $CDCl_3$)

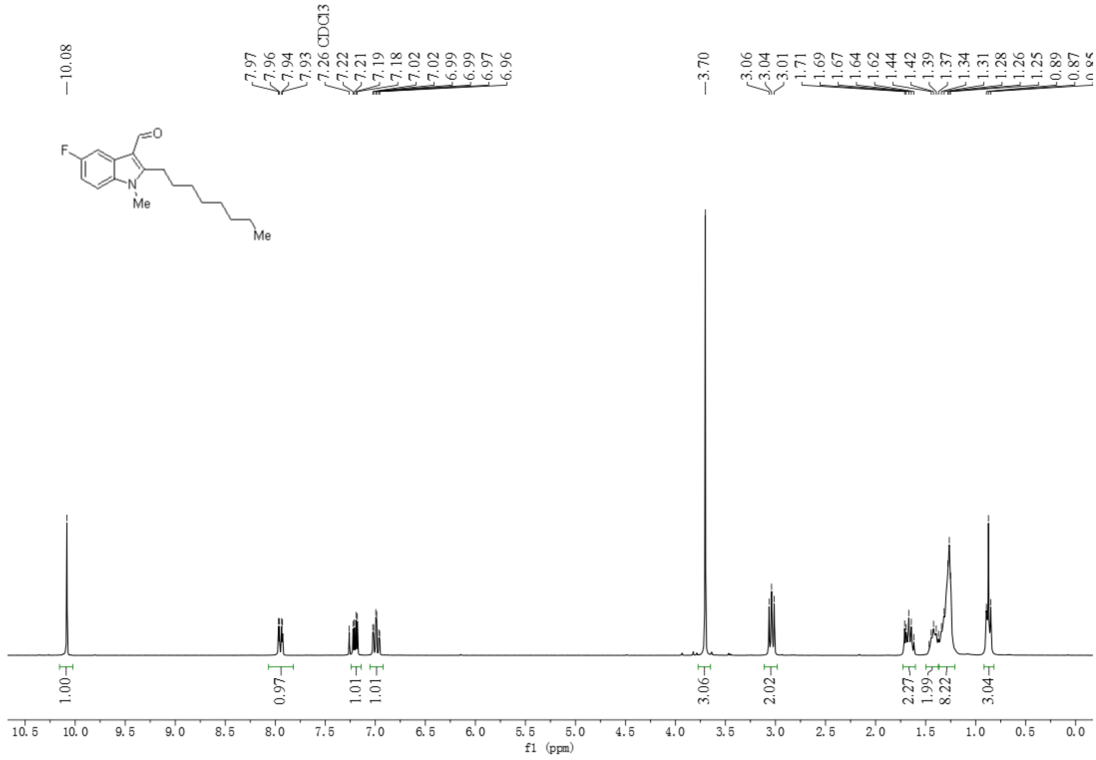

^{13}C NMR spectrum of **10** (75 MHz, $CDCl_3$)

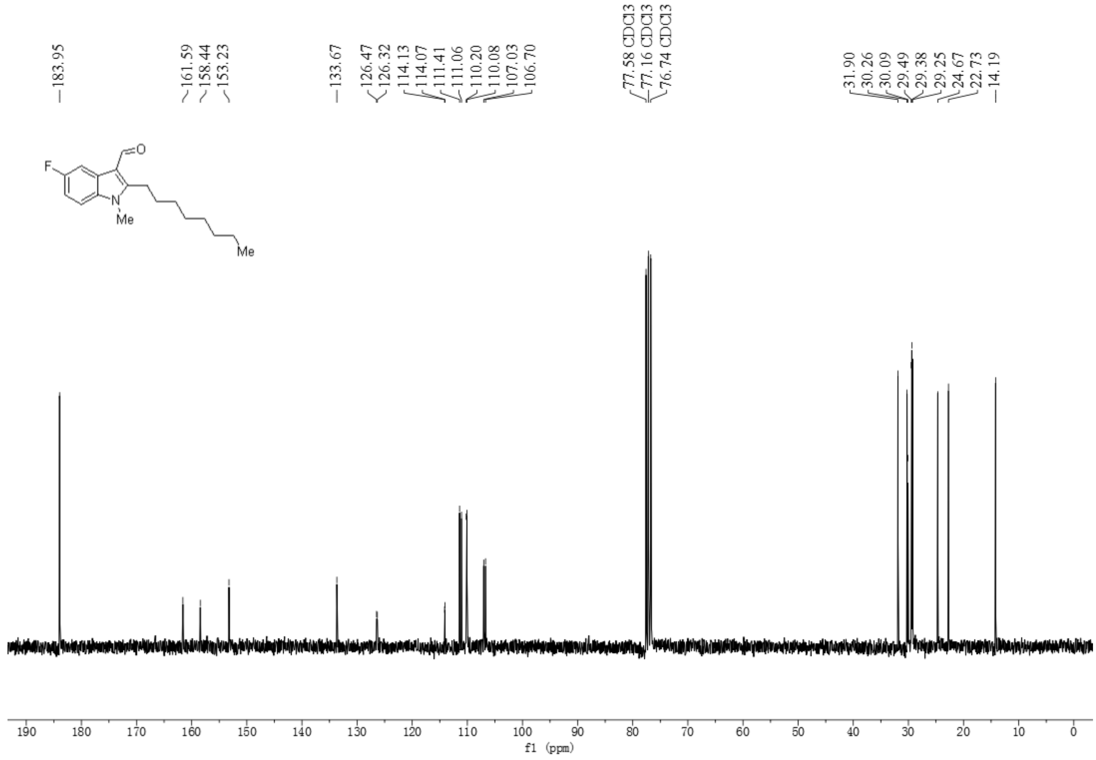

^{19}F NMR spectrum of **10** (282 MHz, $CDCl_3$)

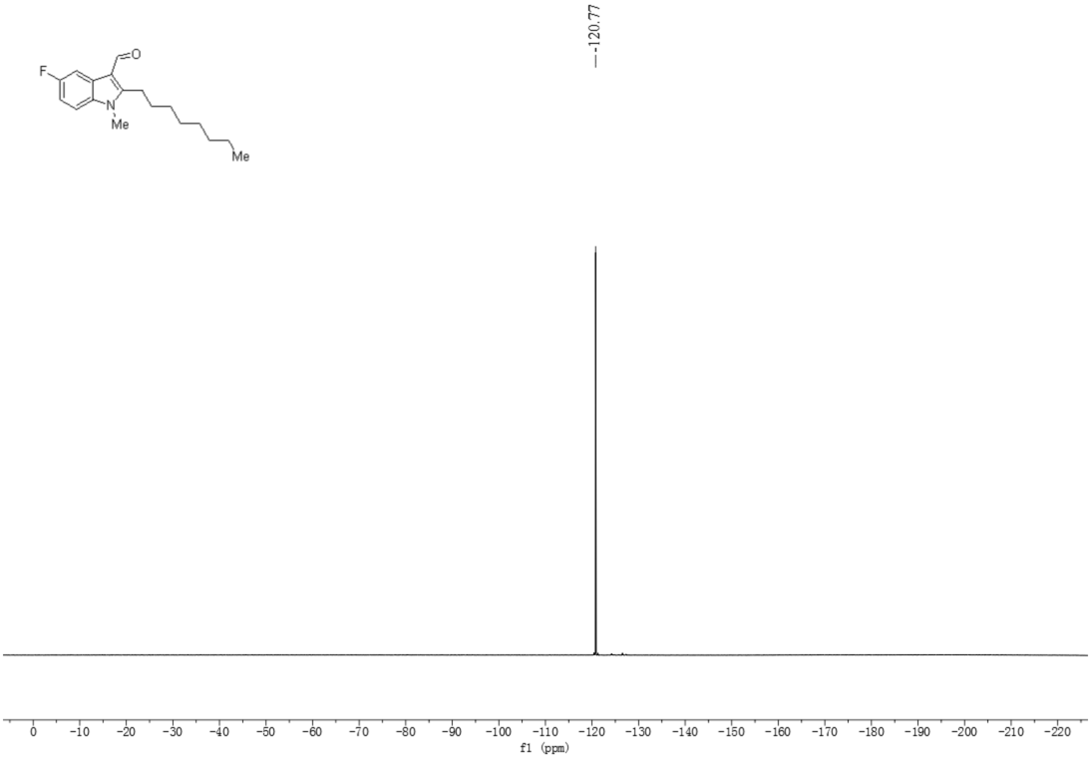

^{1}H NMR spectrum of **11** (300 MHz, $CDCl_3$)

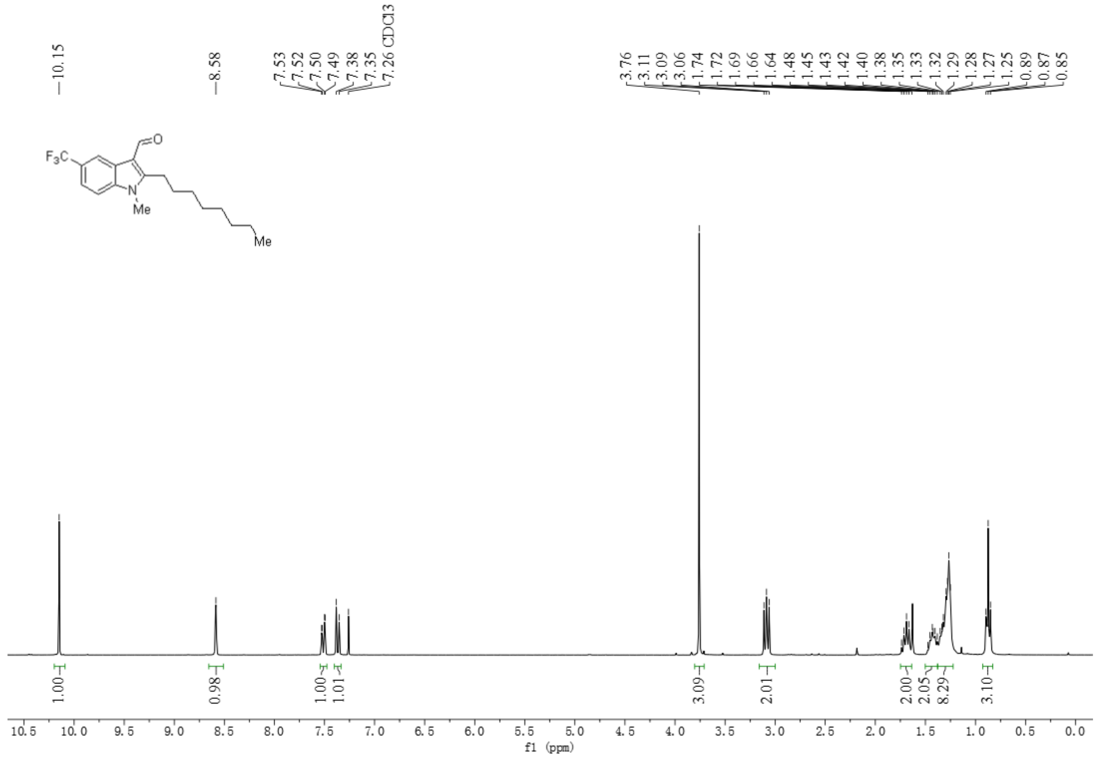

^{13}C NMR spectrum of **11** (75 MHz, $CDCl_3$)

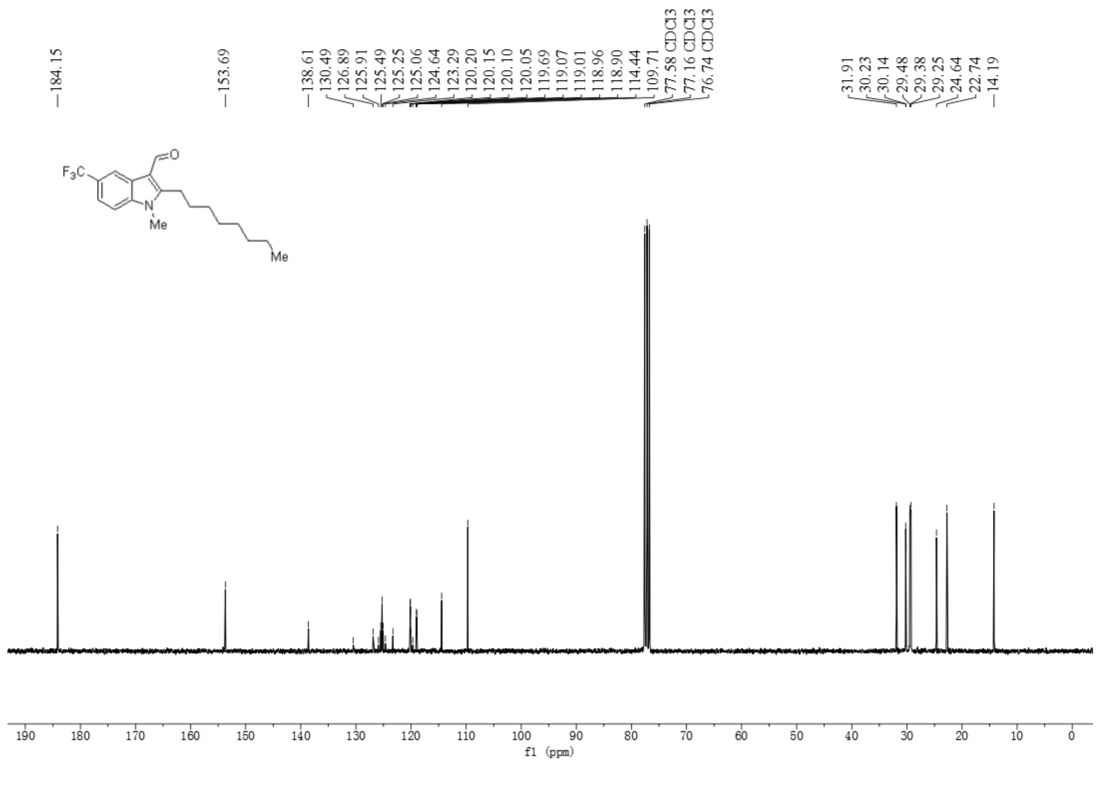

^{19}F NMR spectrum of **11** (282 MHz, $CDCl_3$)

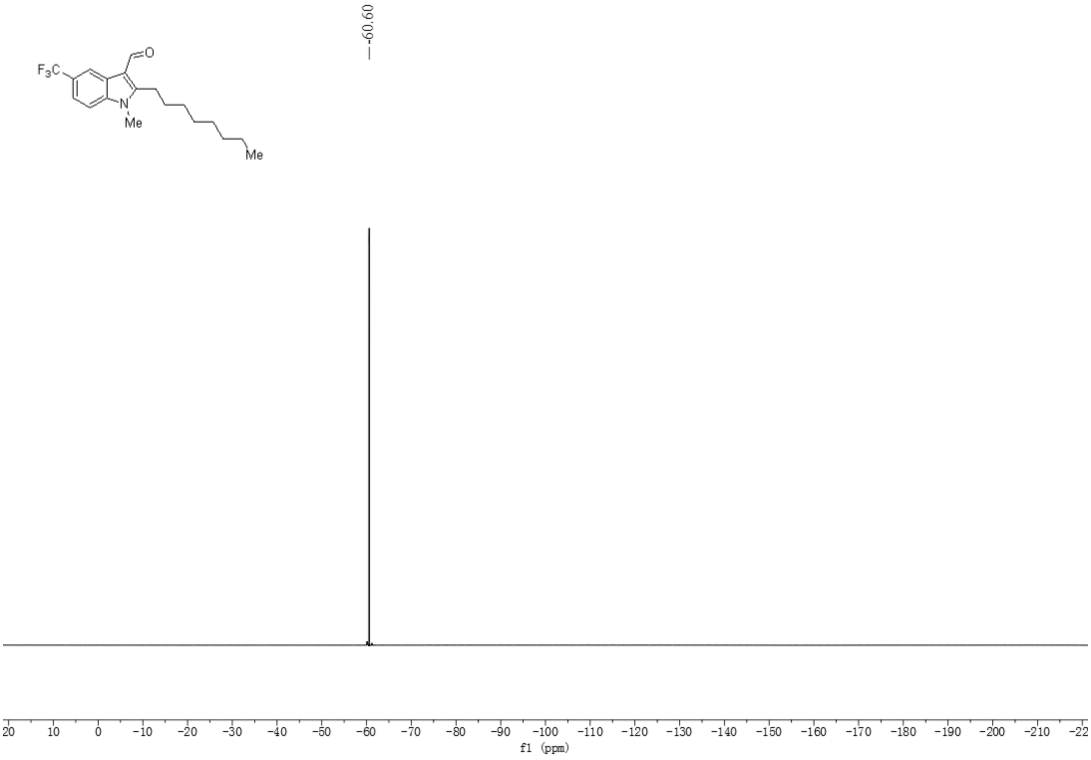

^{1}H NMR spectrum of **12** (300 MHz, $CDCl_3$)

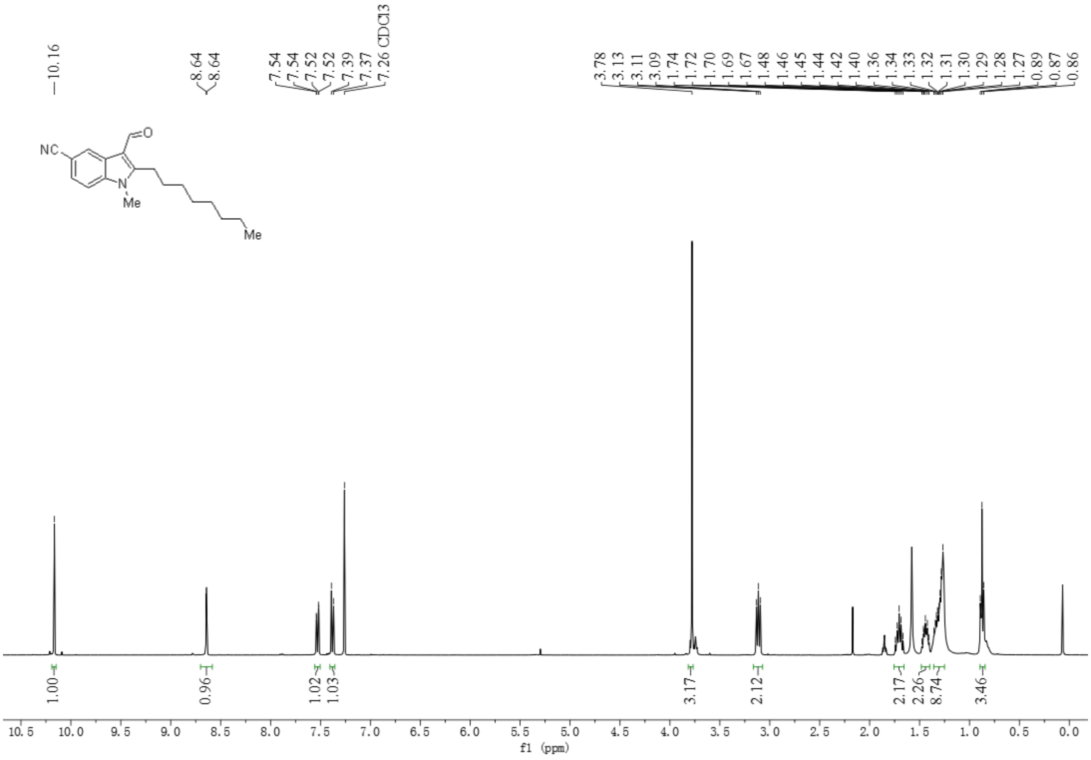

^{13}C NMR spectrum of **12** (75 MHz, $CDCl_3$)

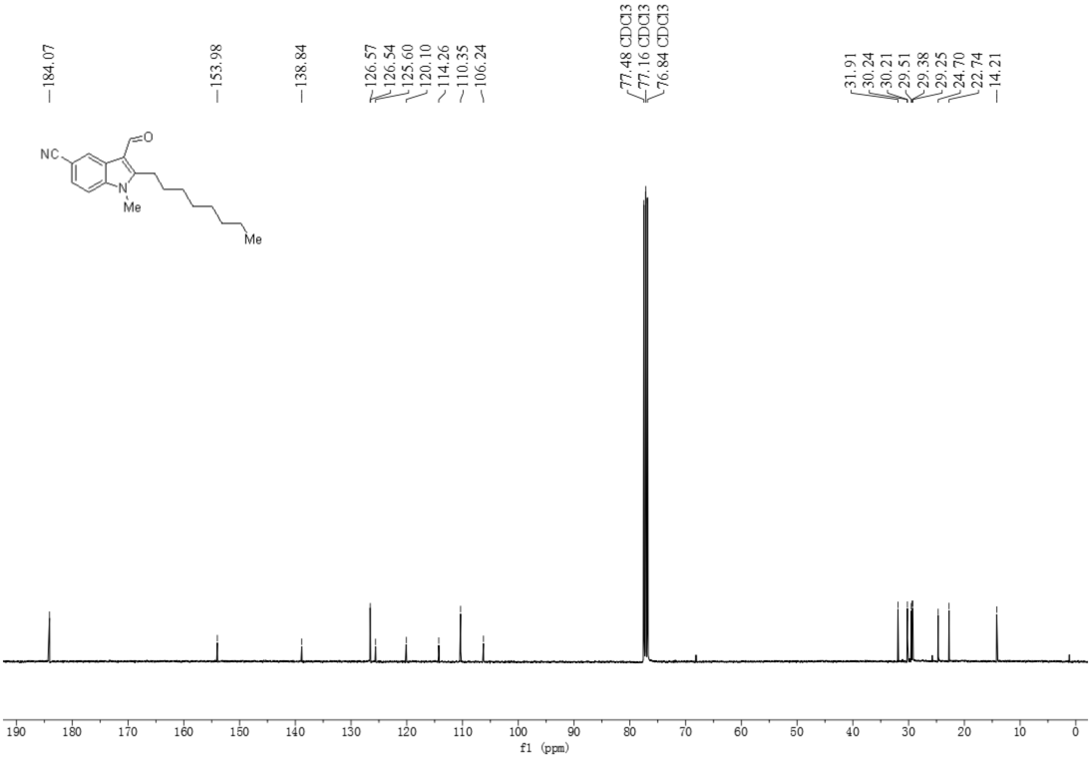

^{1}H NMR spectrum of **13** (300 MHz, $CDCl_3$)

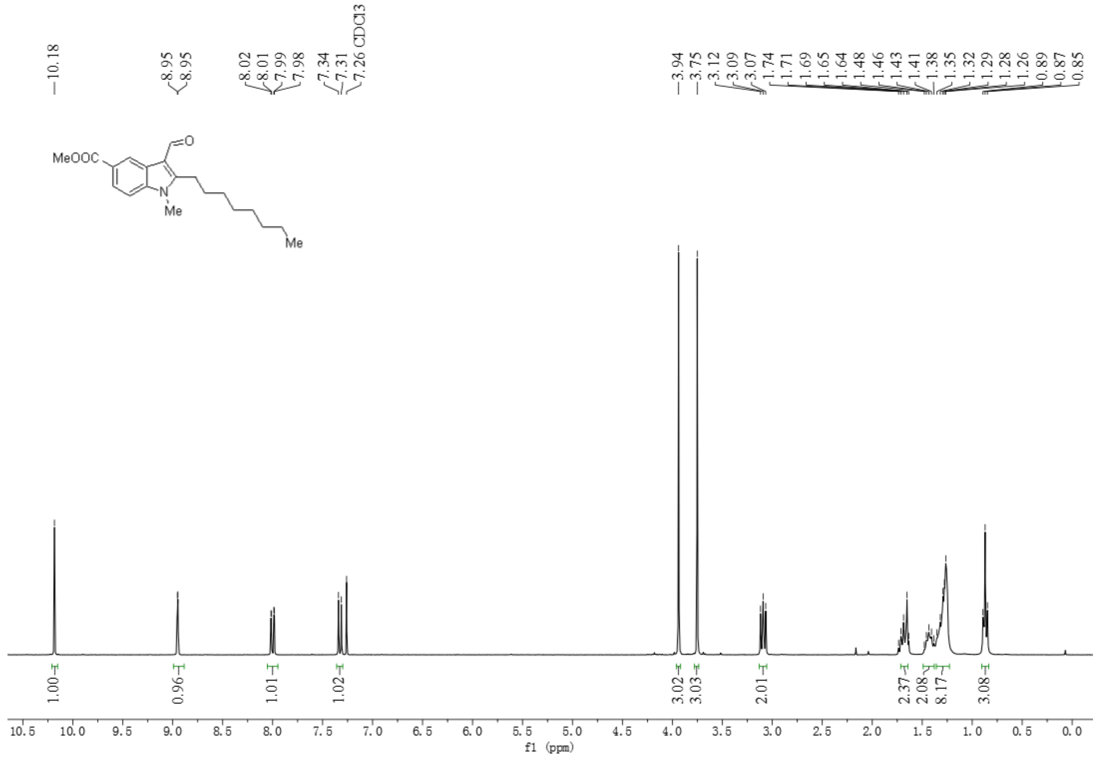

^{13}C NMR spectrum of **13** (75 MHz, $CDCl_3$)

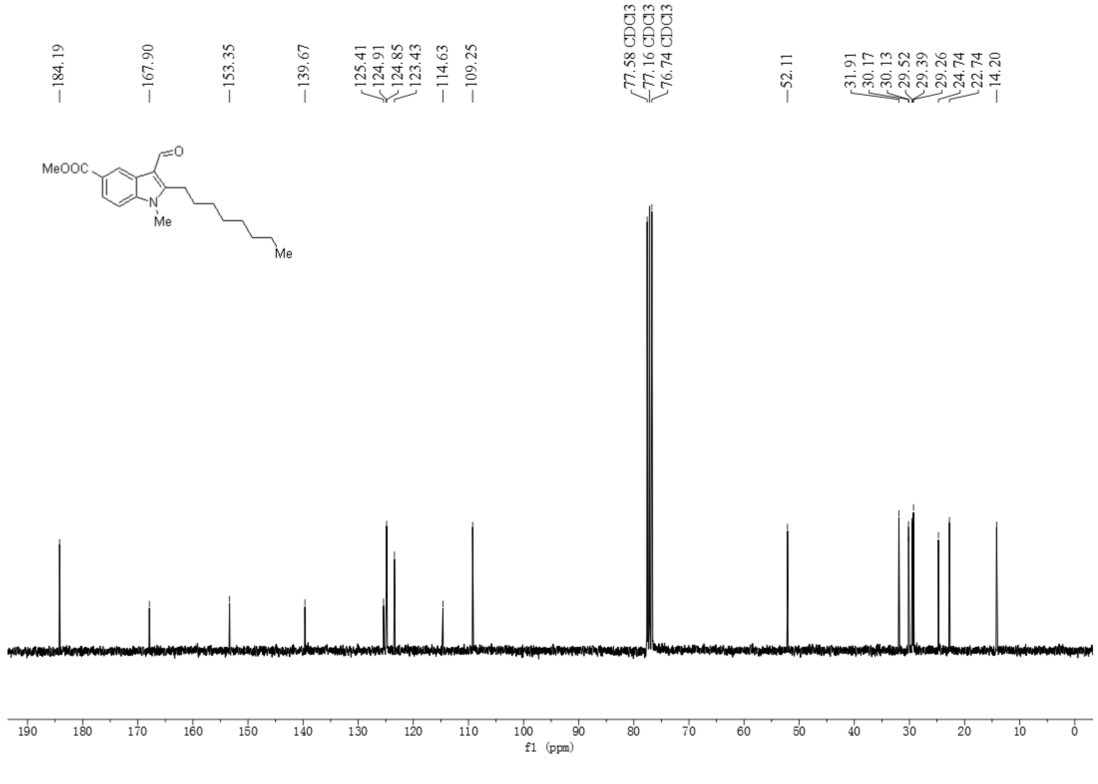

^{1}H NMR spectrum of **14** (300 MHz, $CDCl_3$)

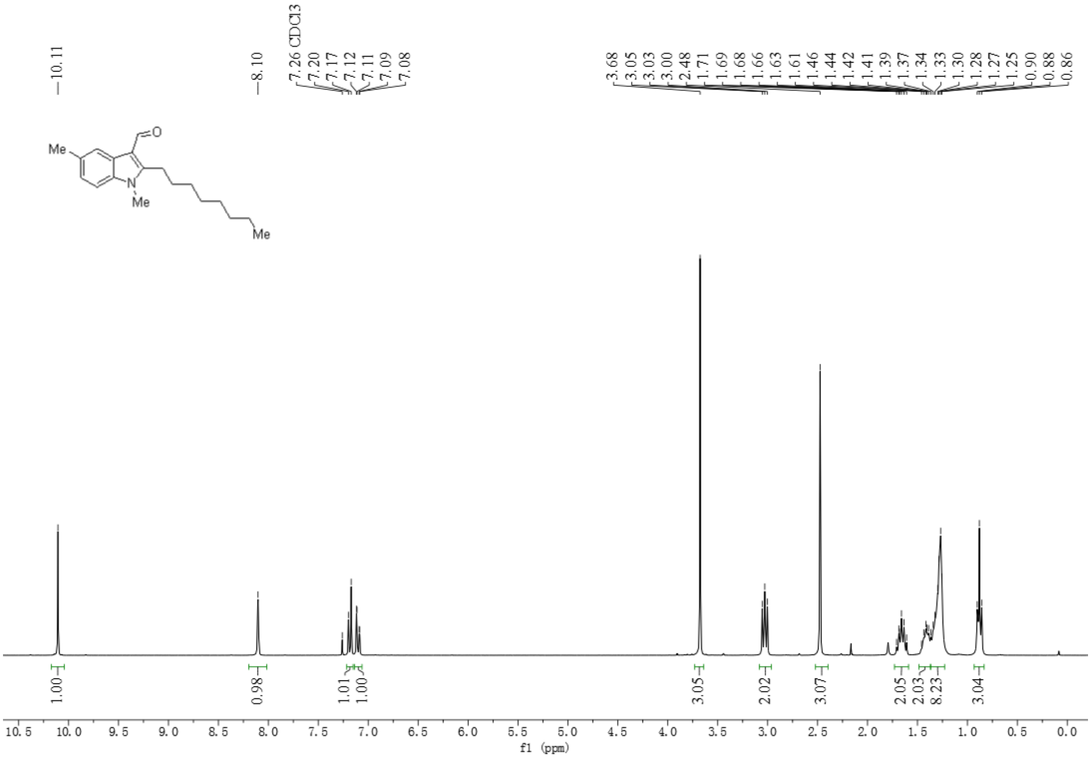

^{13}C NMR spectrum of **14** (75 MHz, $CDCl_3$)

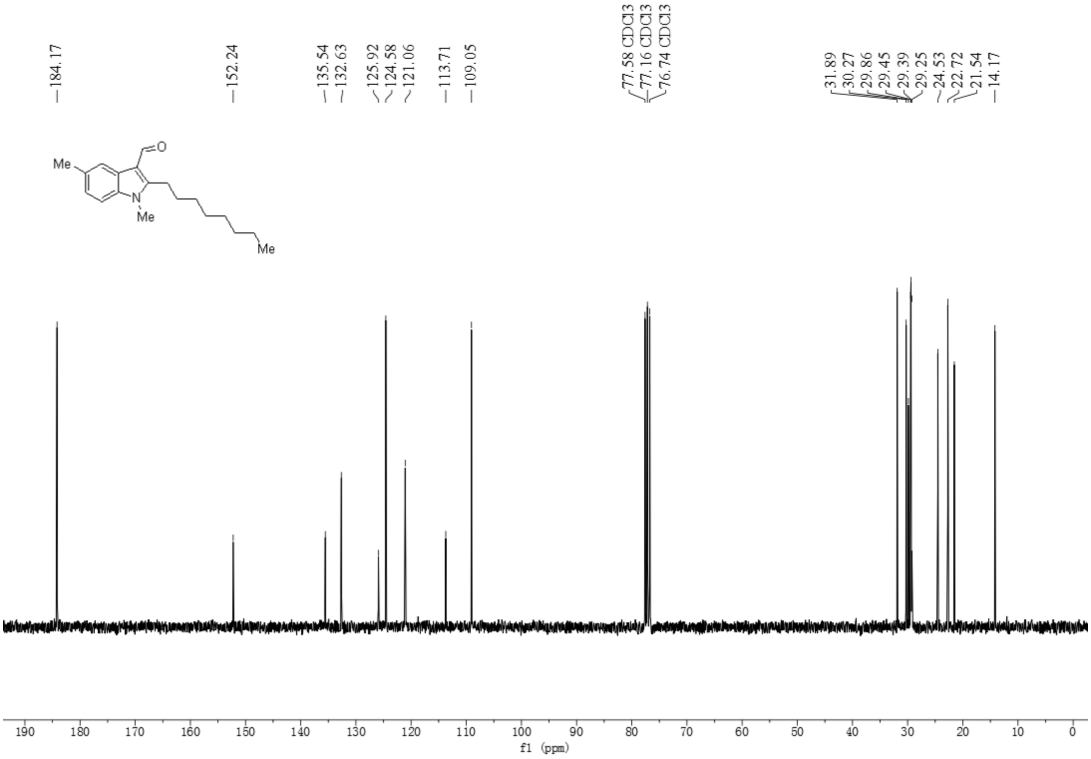

^{1}H NMR spectrum of **15** (300 MHz, $CDCl_3$)

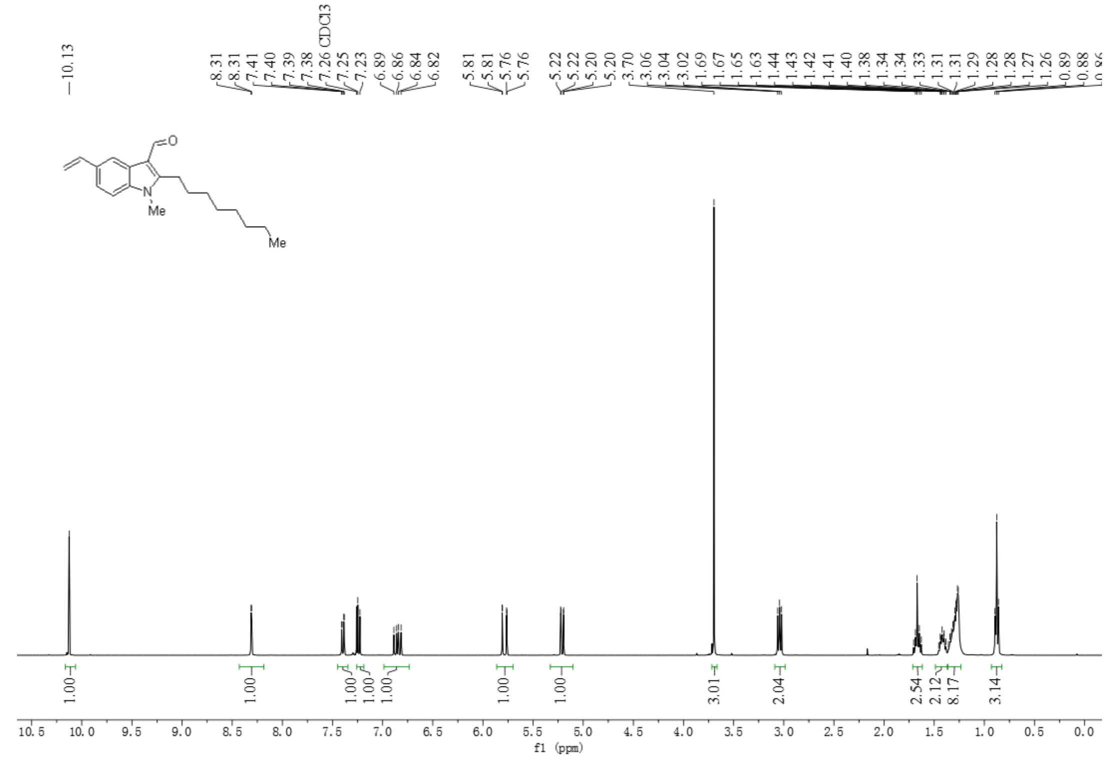

^{13}C NMR spectrum of **15** (75 MHz, $CDCl_3$)

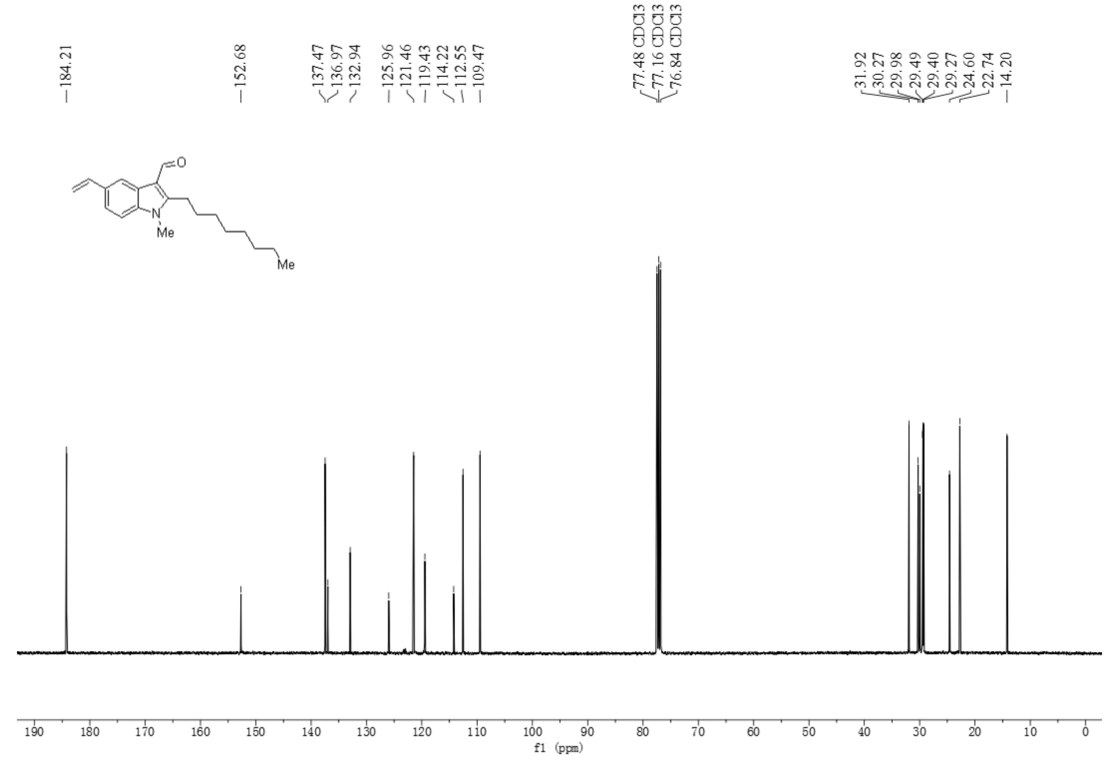

^{1}H NMR spectrum of **16** (300 MHz, $CDCl_3$)

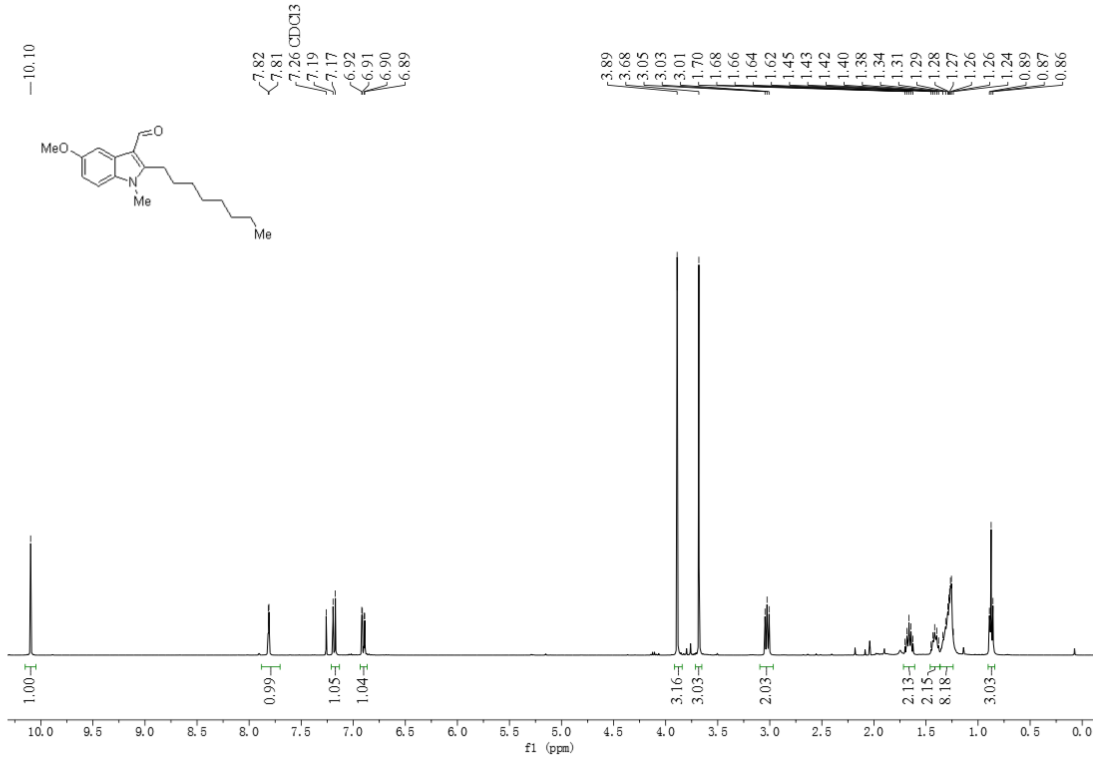

^{13}C NMR spectrum of **16** (75 MHz, $CDCl_3$)

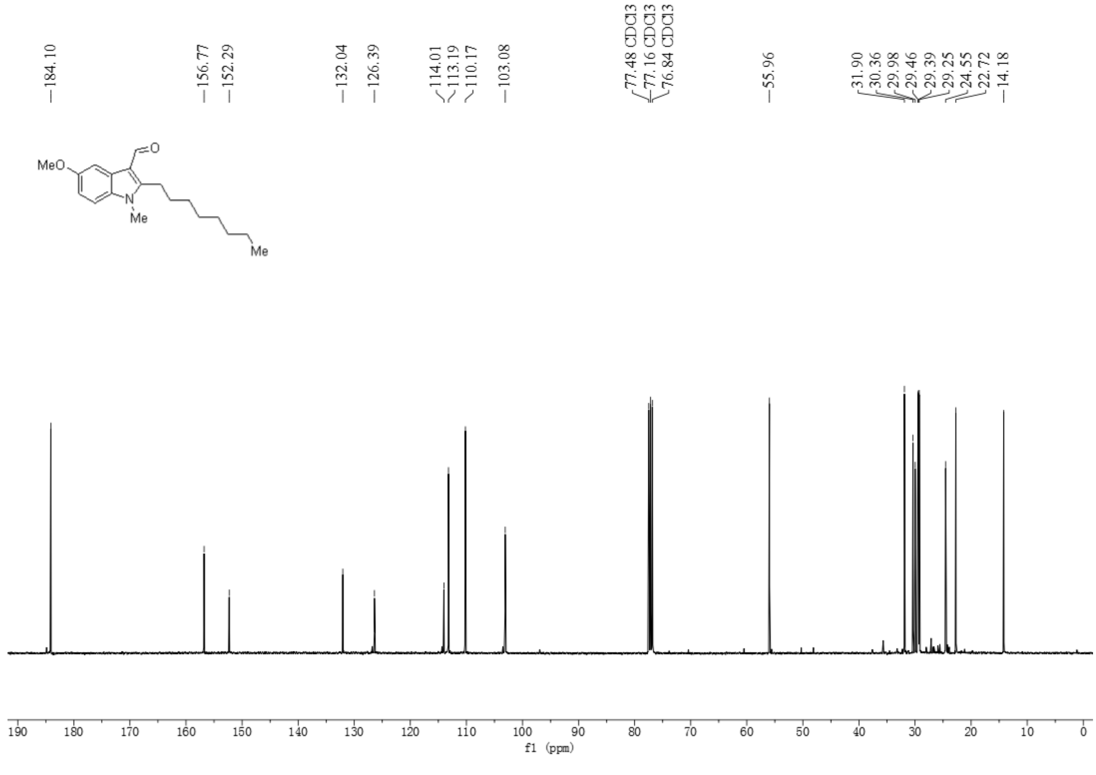

^{1}H NMR spectrum of **17** (300 MHz, $CDCl_3$)

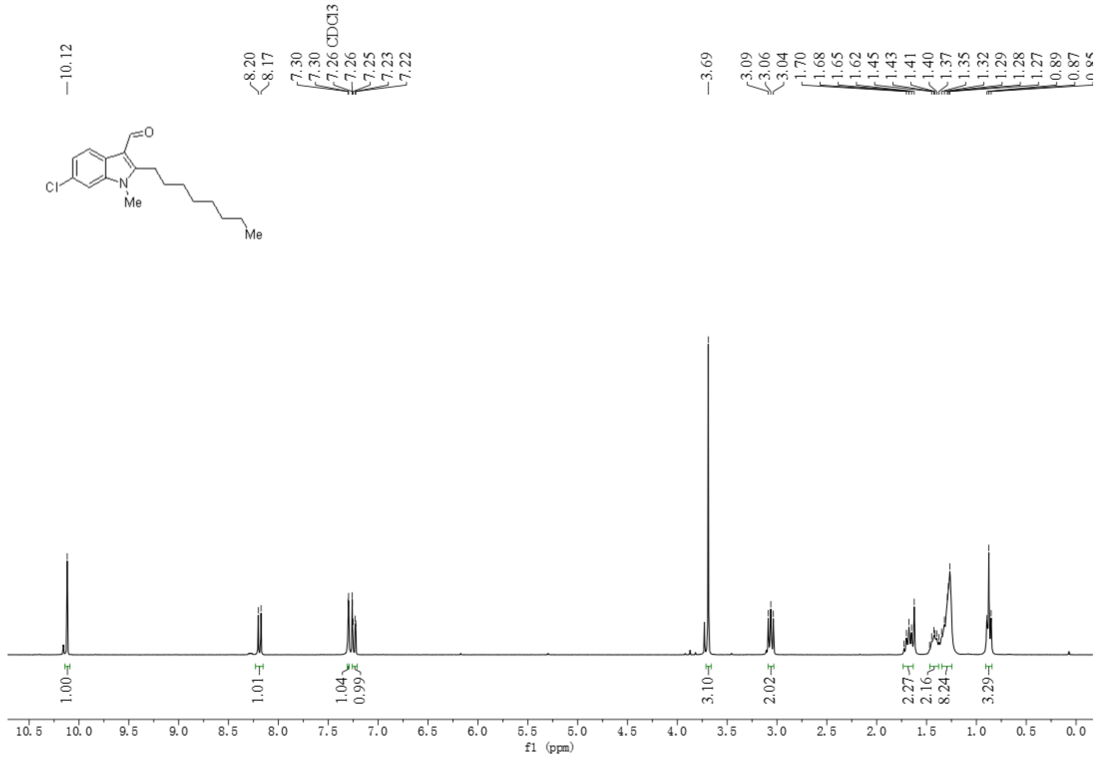

^{13}C NMR spectrum of **17** (75 MHz, $CDCl_3$)

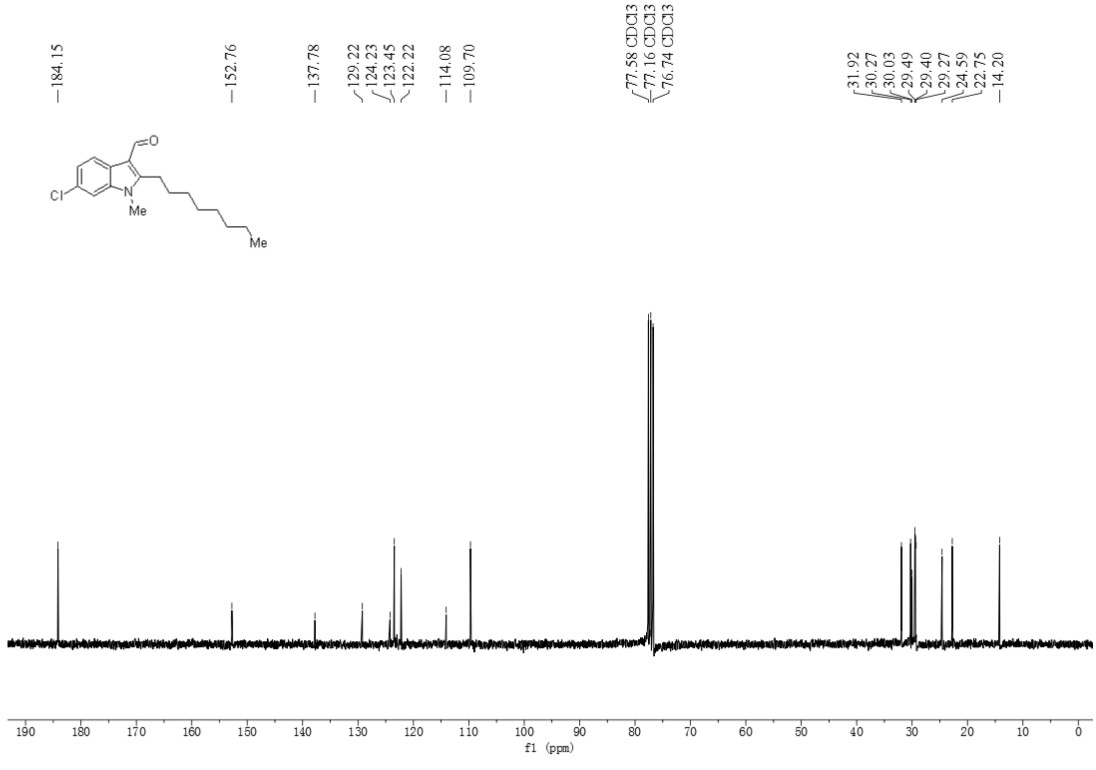

^{1}H NMR spectrum of **18** (300 MHz, $CDCl_3$)

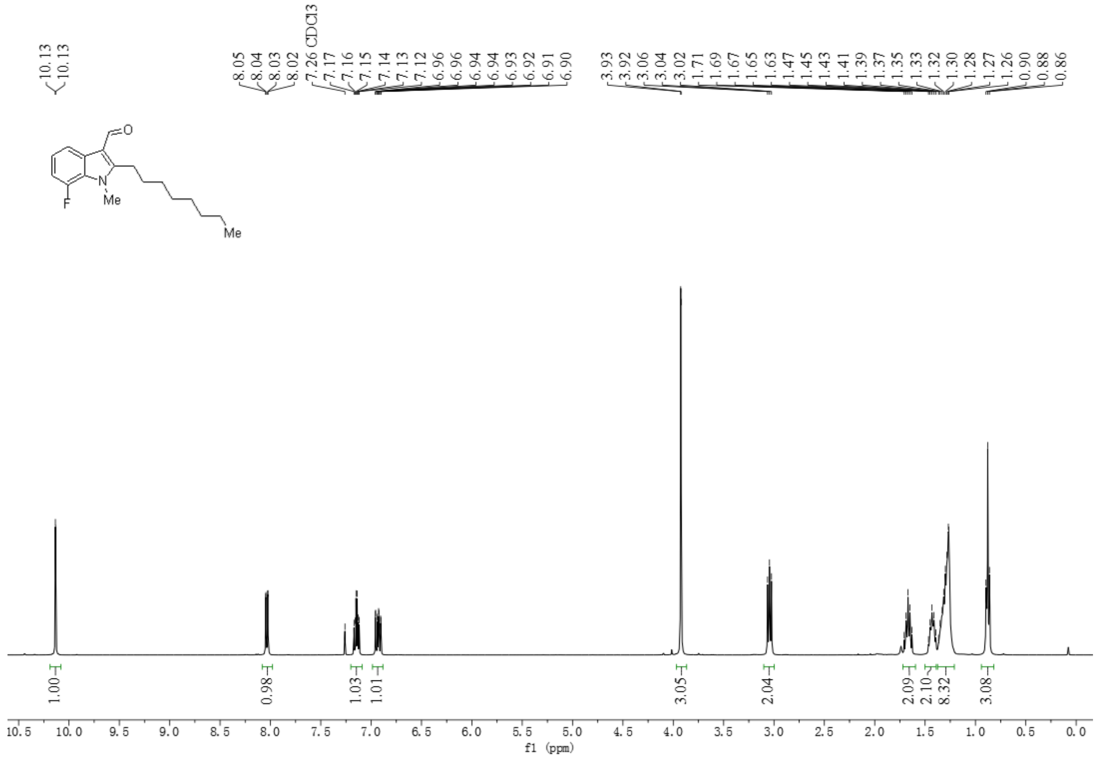

^{13}C NMR spectrum of **18** (75 MHz, $CDCl_3$)

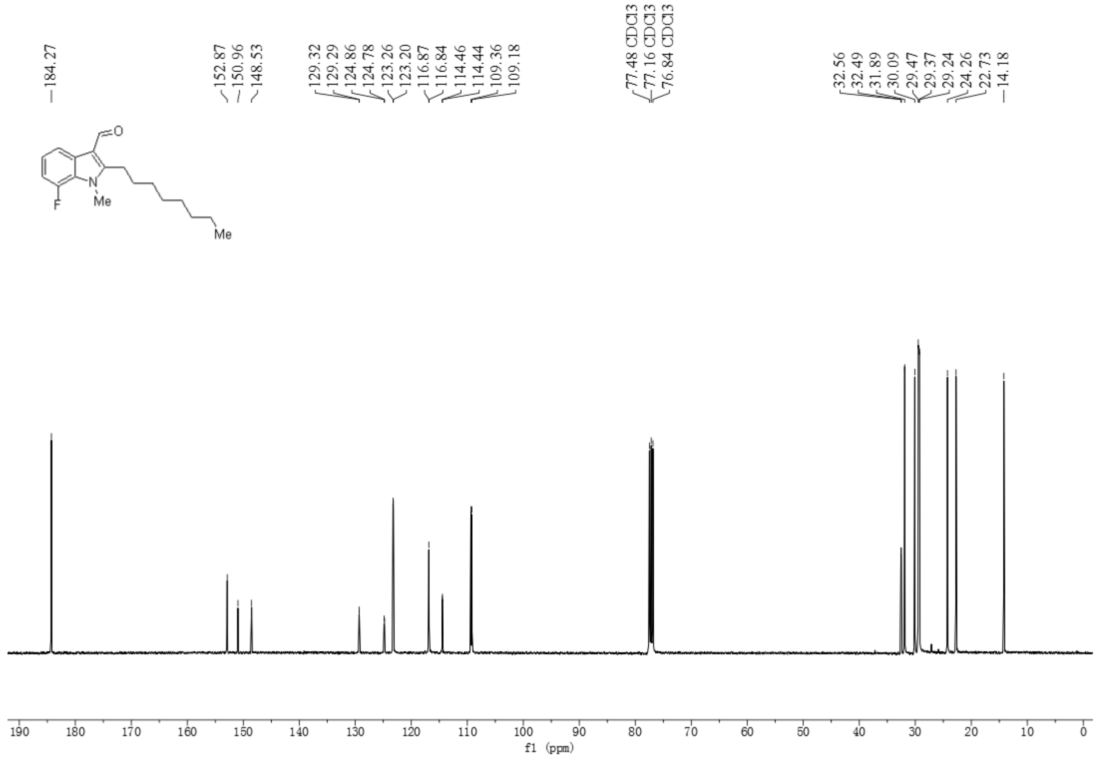

^{19}F NMR spectrum of **18** (282 MHz, $CDCl_3$)

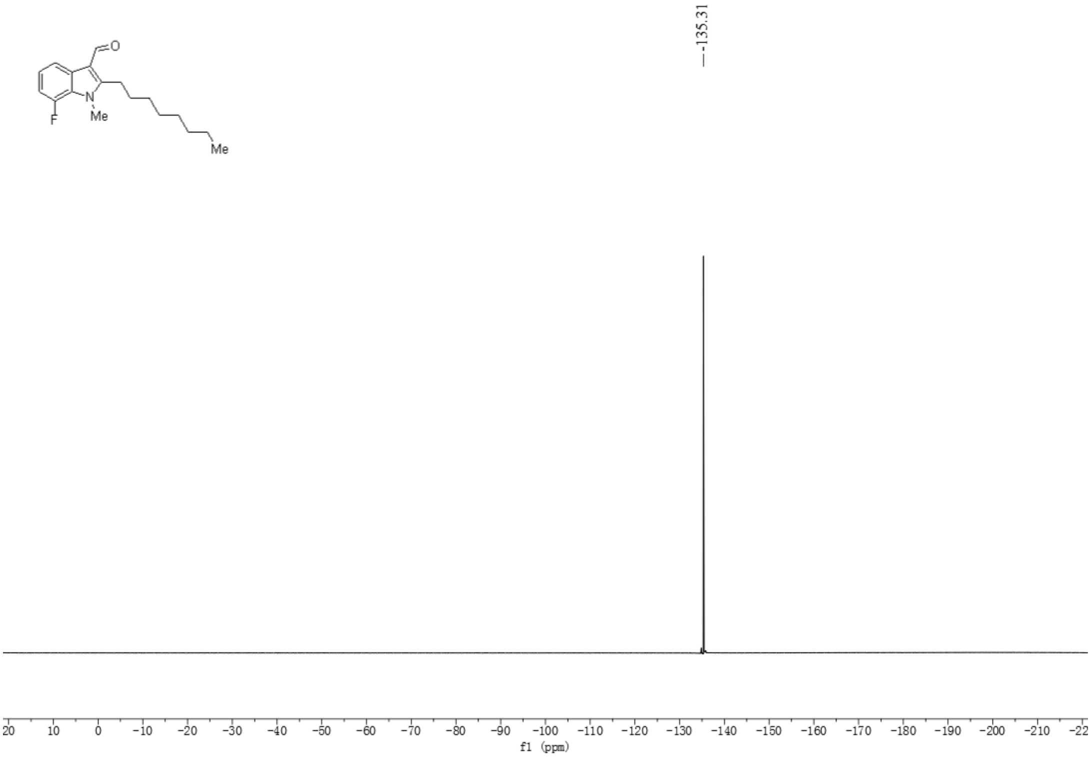

^{1}H NMR spectrum of **19** (300 MHz, $CDCl_3$)

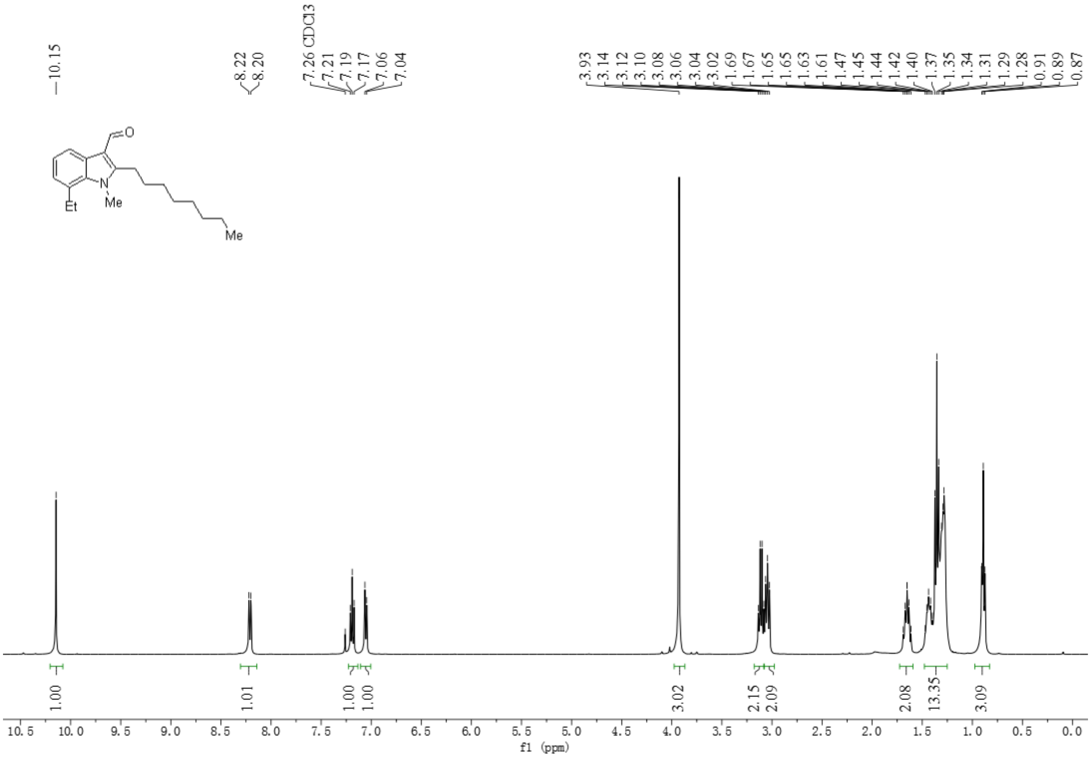

^{13}C NMR spectrum of **19** (75 MHz, $CDCl_3$)

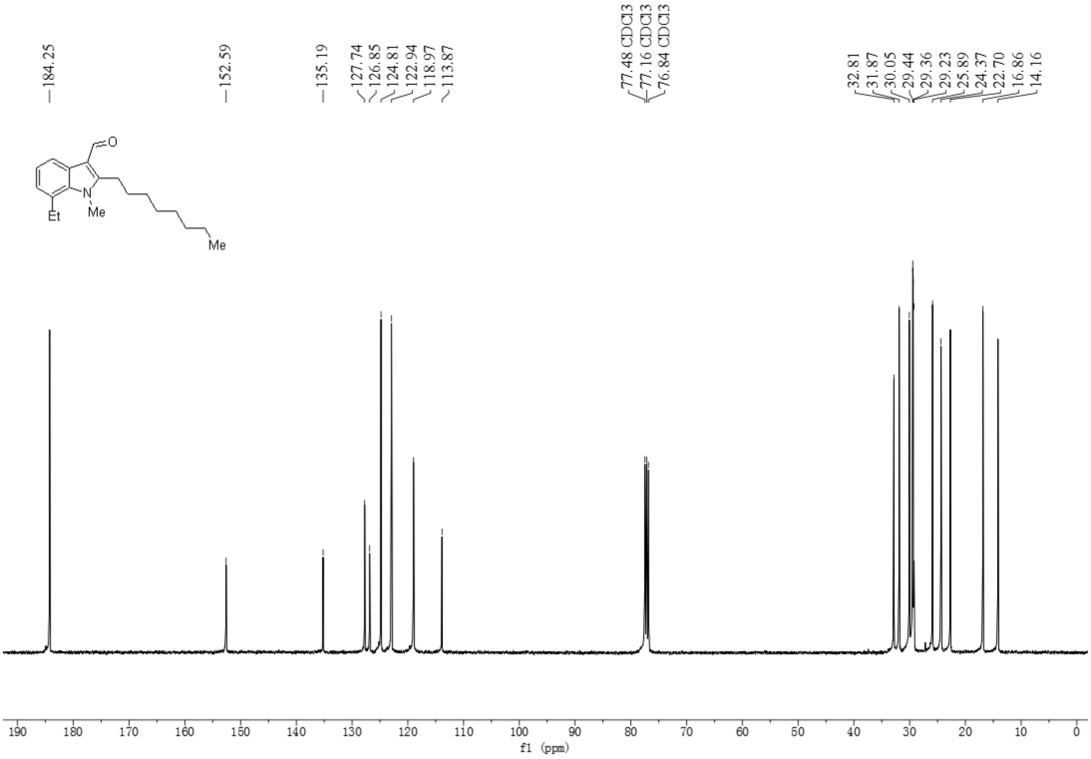

^{1}H NMR spectrum of **20** (300 MHz, $CDCl_3$)

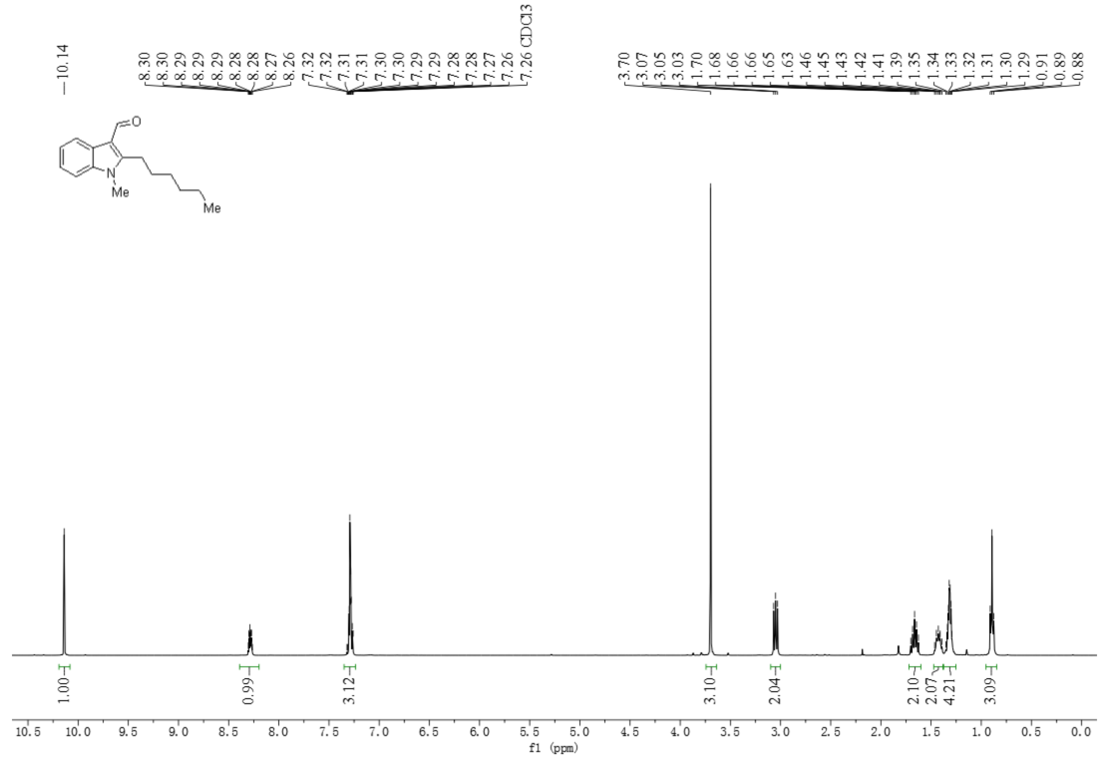

^{13}C NMR spectrum of **20** (75 MHz, $CDCl_3$)

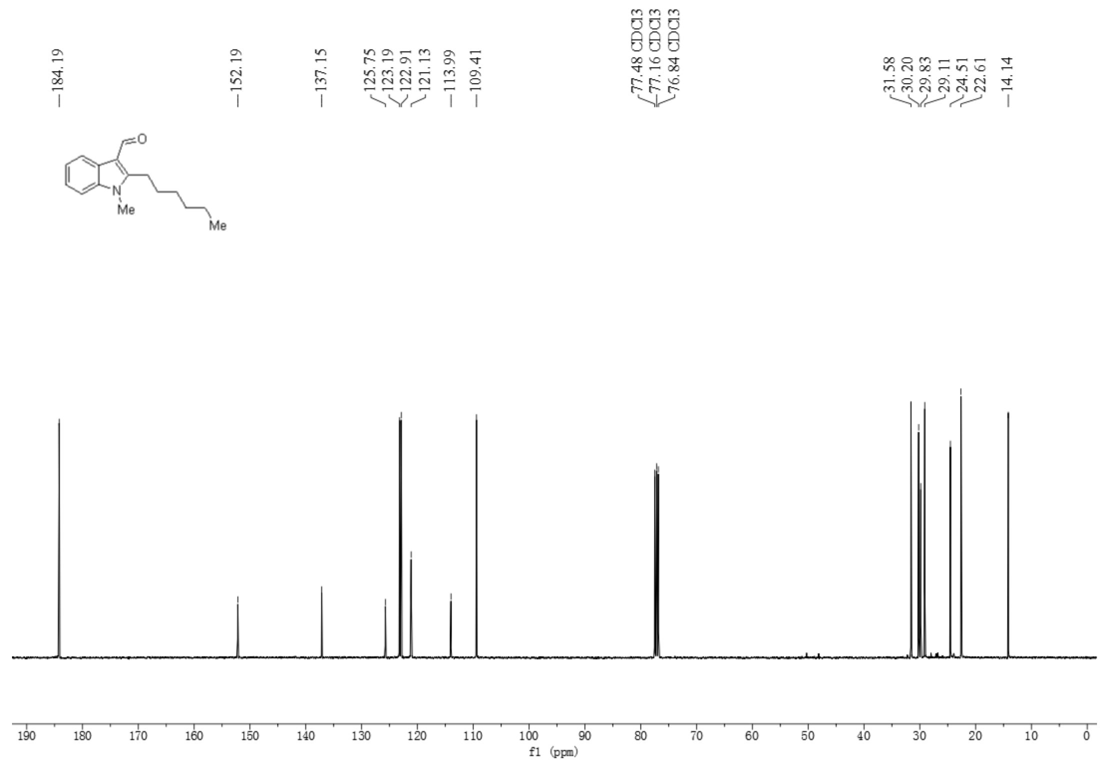

^{1}H NMR spectrum of **21** (300 MHz, $CDCl_3$)

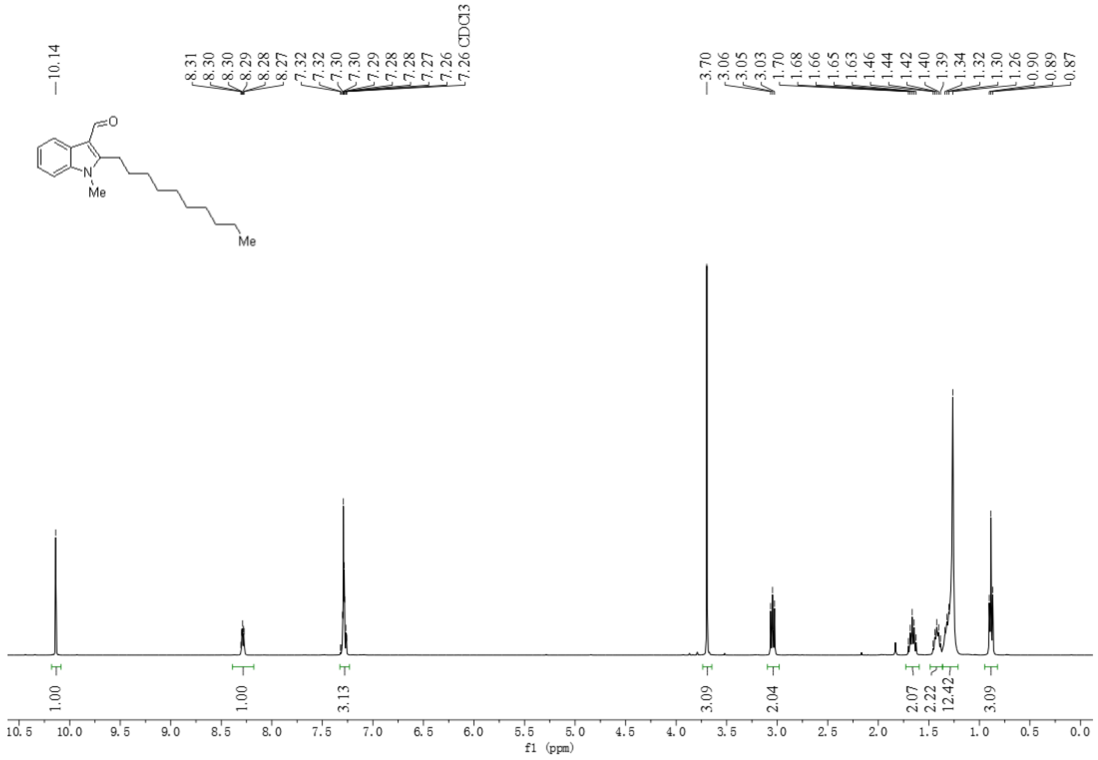

^{13}C NMR spectrum of **21** (75 MHz, $CDCl_3$)

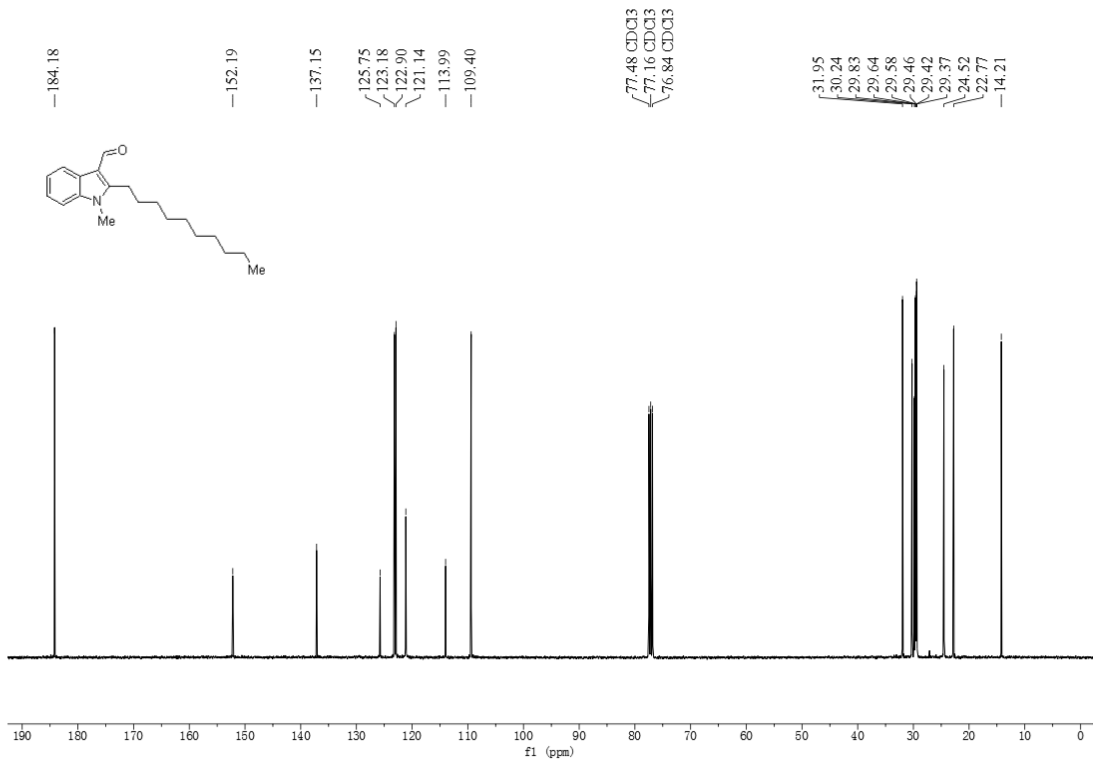

^{1}H NMR spectrum of **22** (300 MHz, $CDCl_3$)

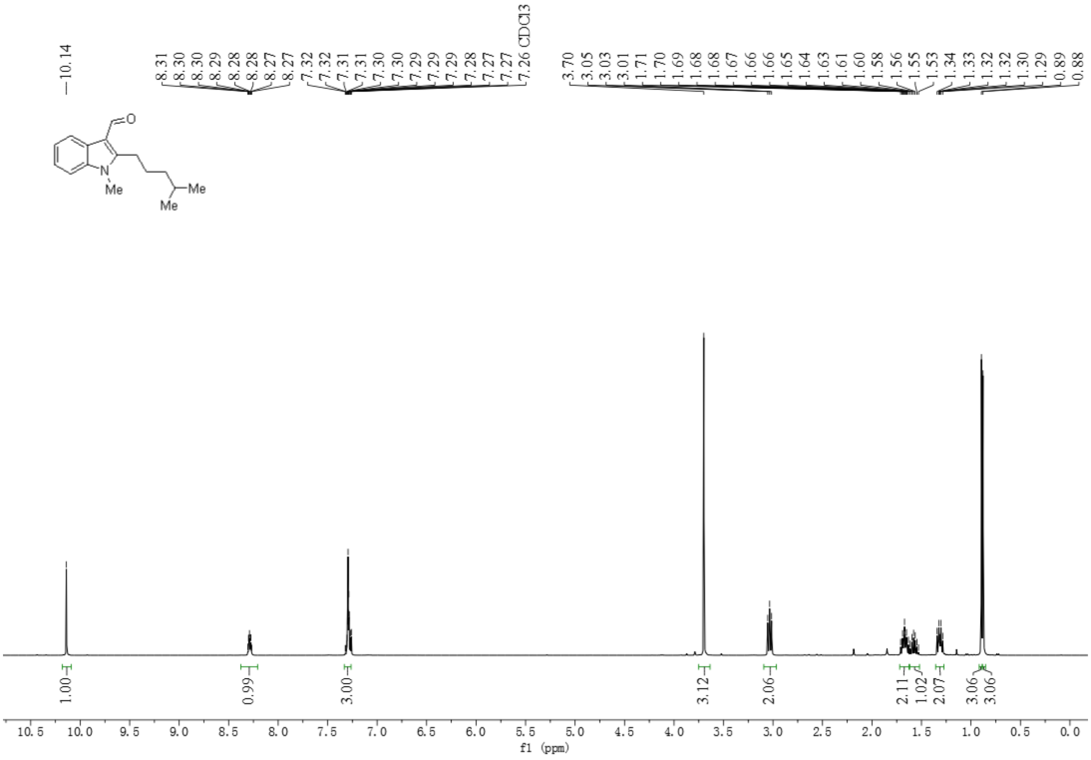

^{13}C NMR spectrum of **22** (75 MHz, $CDCl_3$)

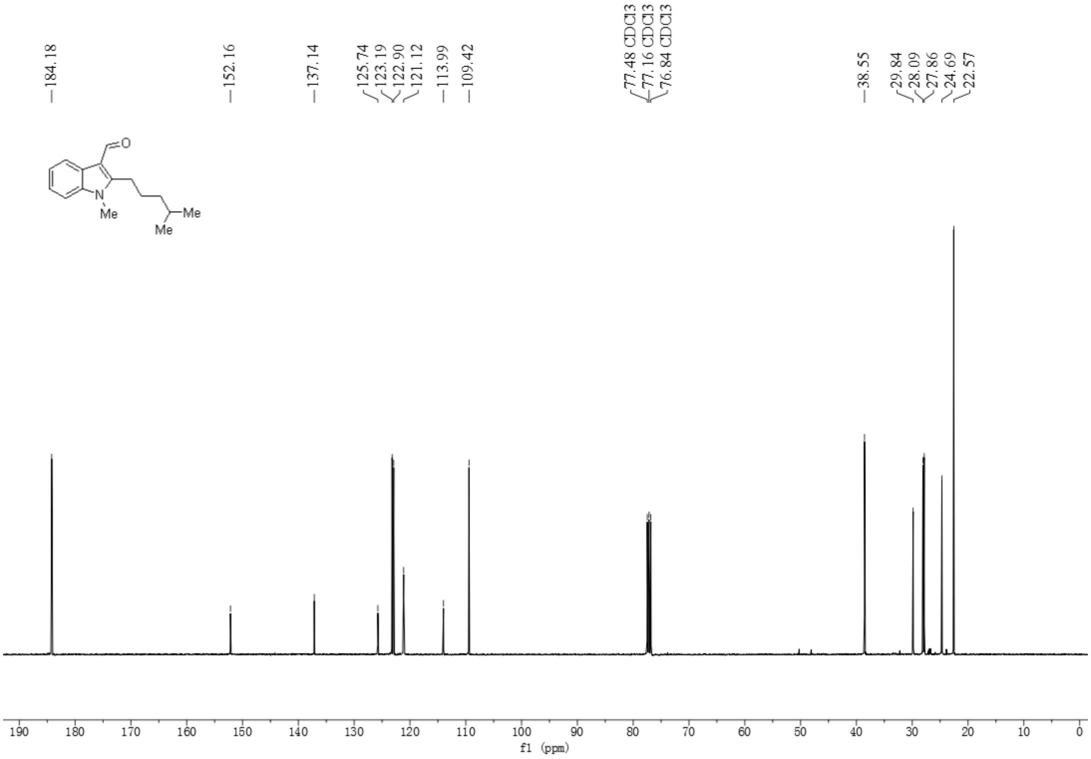

^{1}H NMR spectrum of **23** (300 MHz, $CDCl_3$)

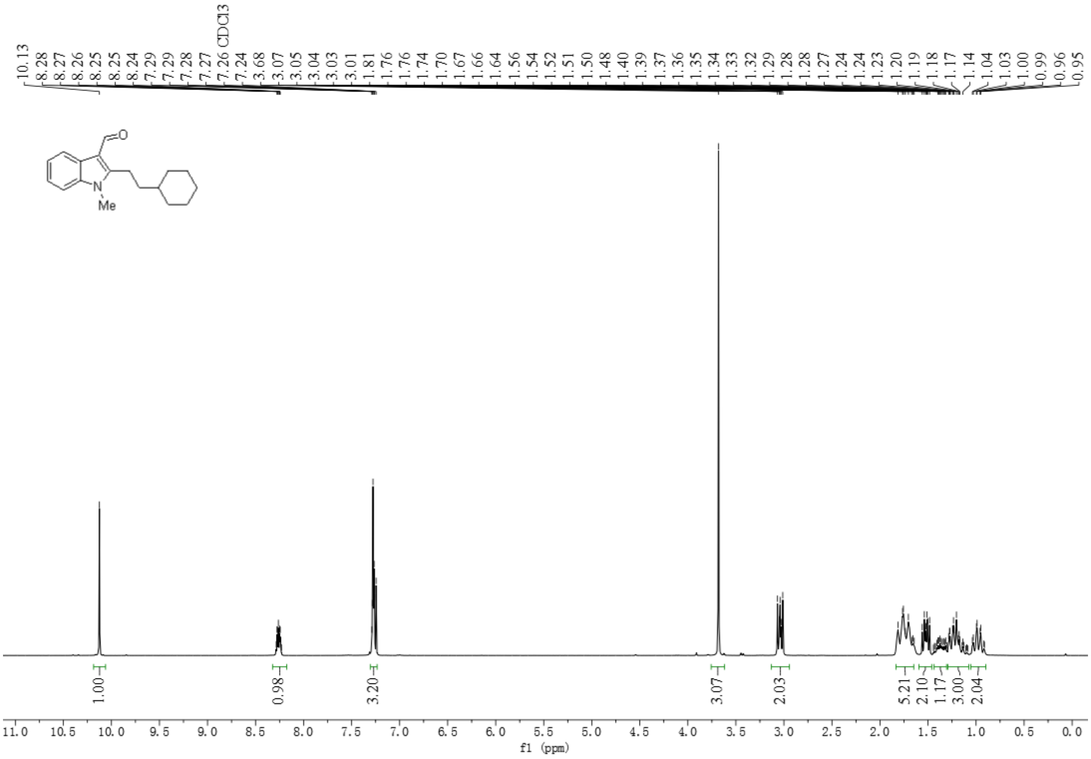

^{13}C NMR spectrum of **23** (75 MHz, $CDCl_3$)

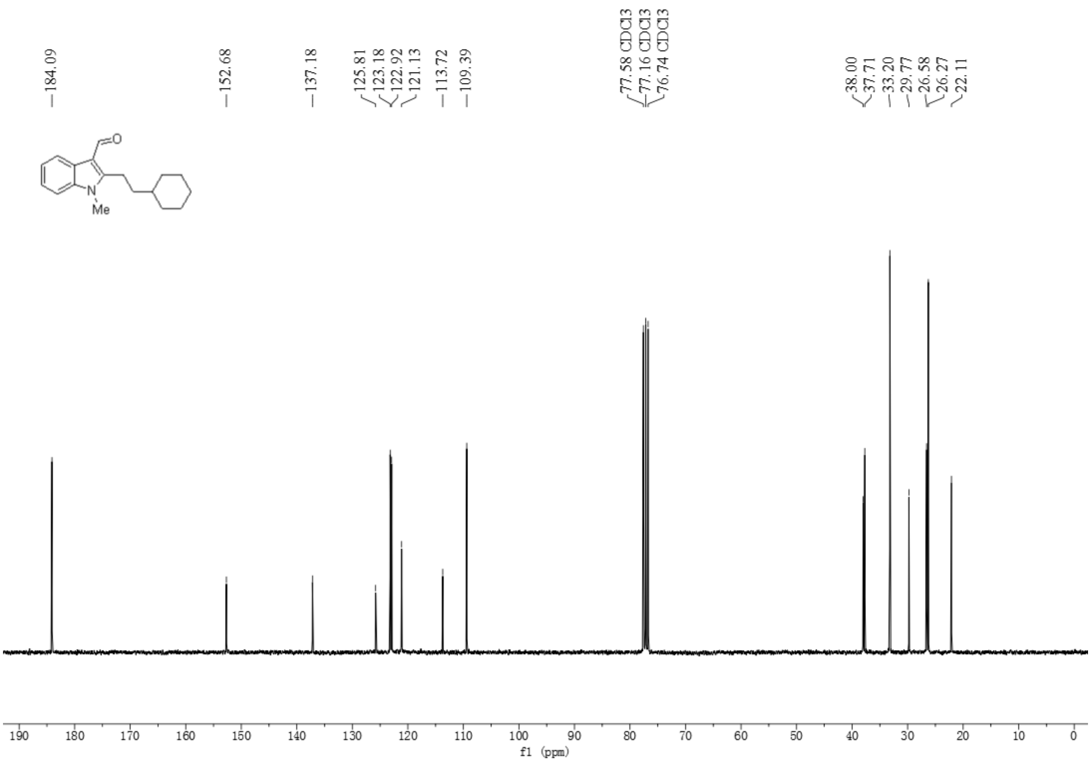

^{1}H NMR spectrum of **24** (300 MHz, $CDCl_3$)

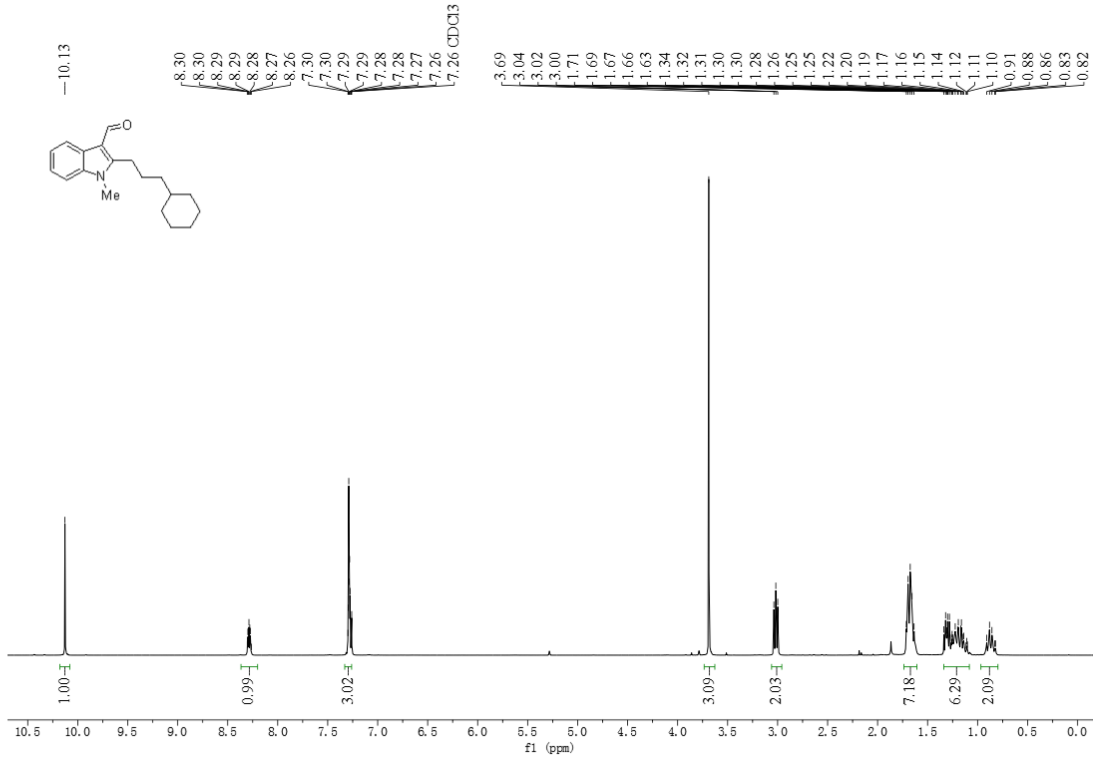

^{13}C NMR spectrum of **24** (75 MHz, $CDCl_3$)

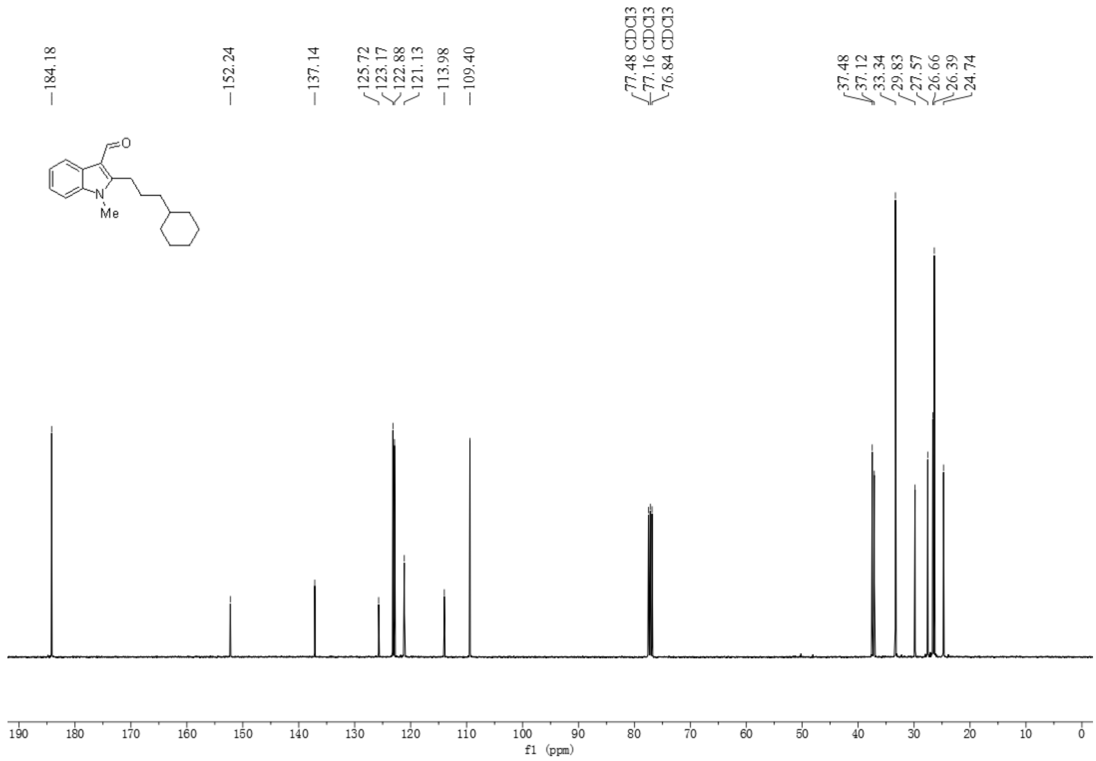

^{1}H NMR spectrum of **25** (300 MHz, $CDCl_3$)

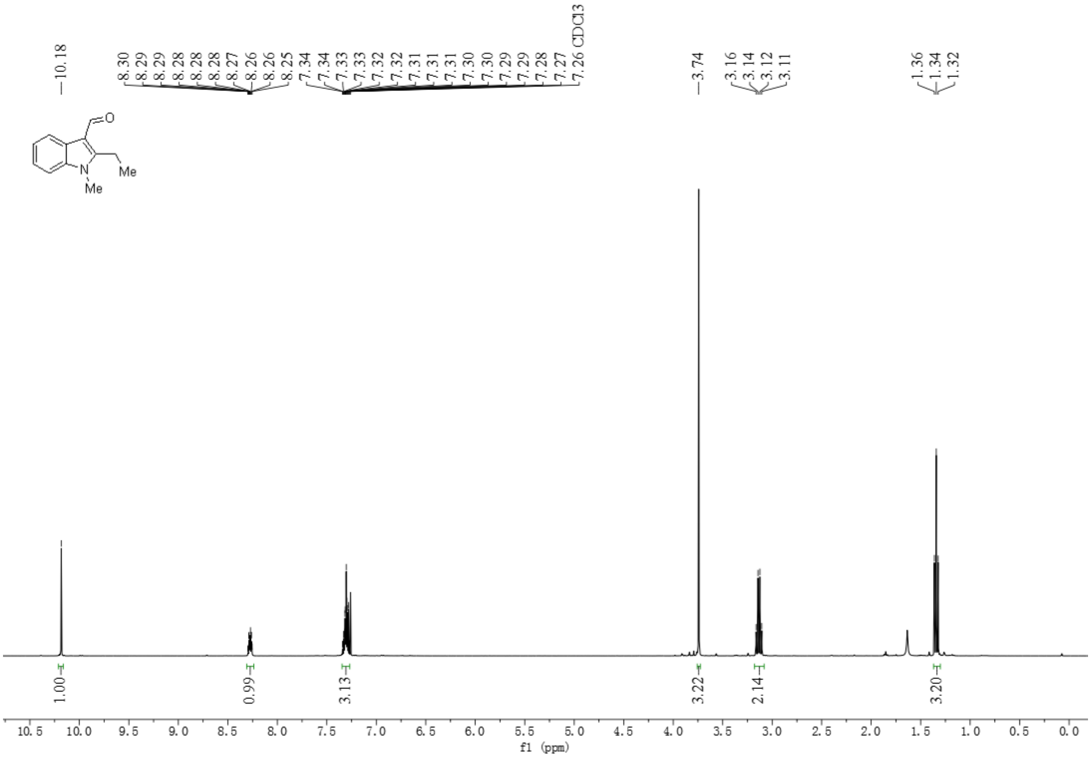

^{13}C NMR spectrum of **25** (75 MHz, $CDCl_3$)

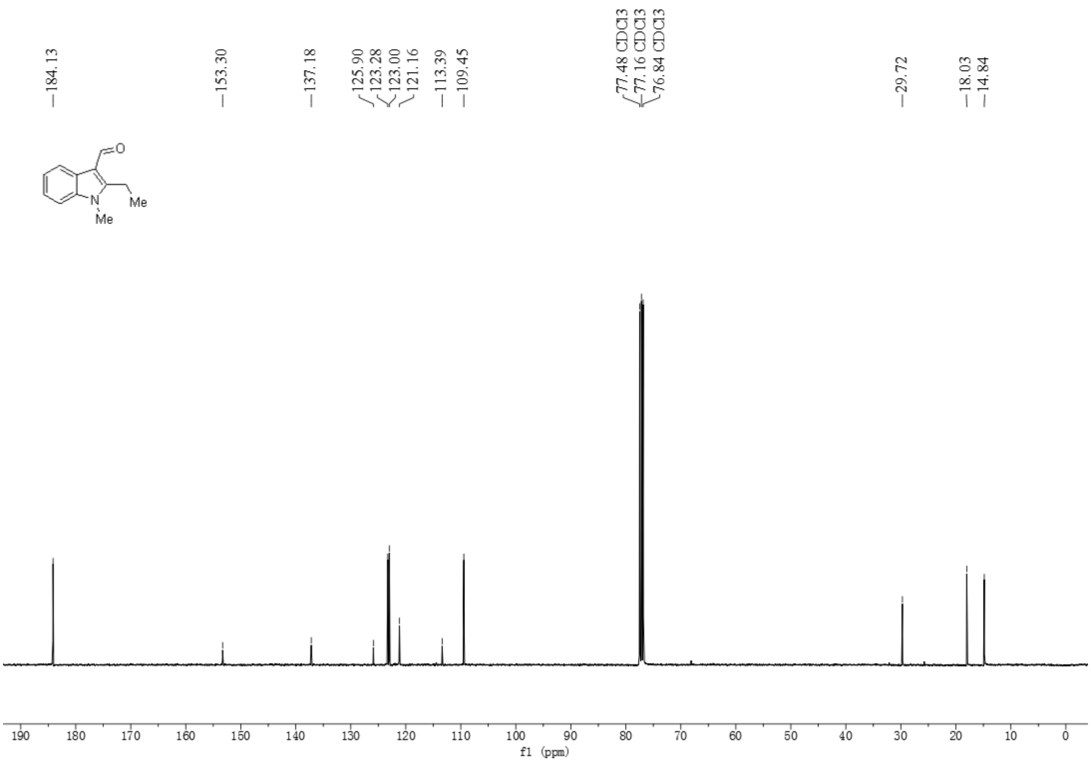

^{1}H NMR spectrum of **26** (300 MHz, $CDCl_3$)

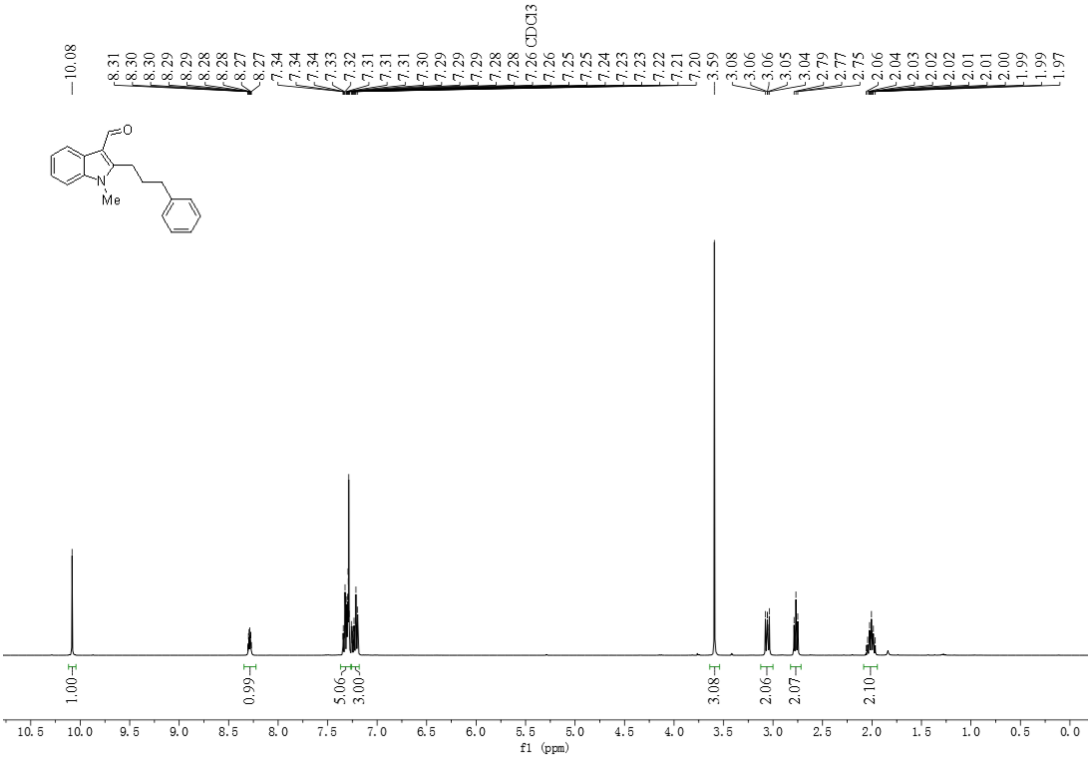

^{13}C NMR spectrum of **26** (75 MHz, $CDCl_3$)

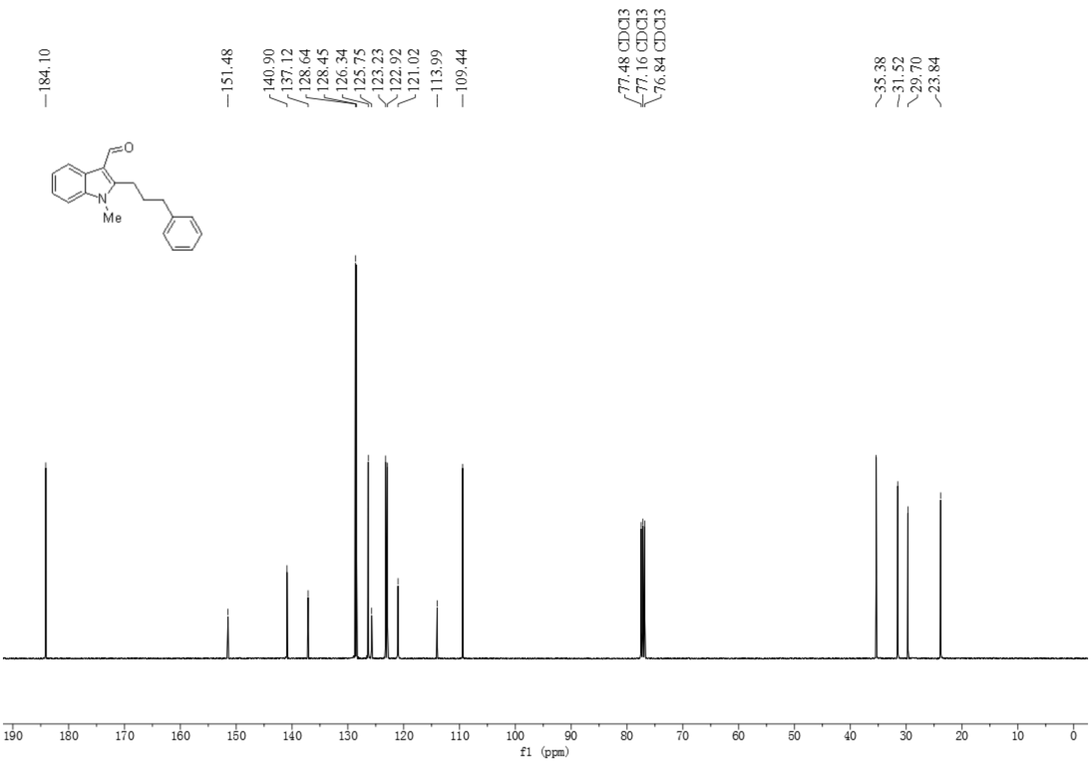

^{1}H NMR spectrum of **27** (300 MHz, $CDCl_3$)

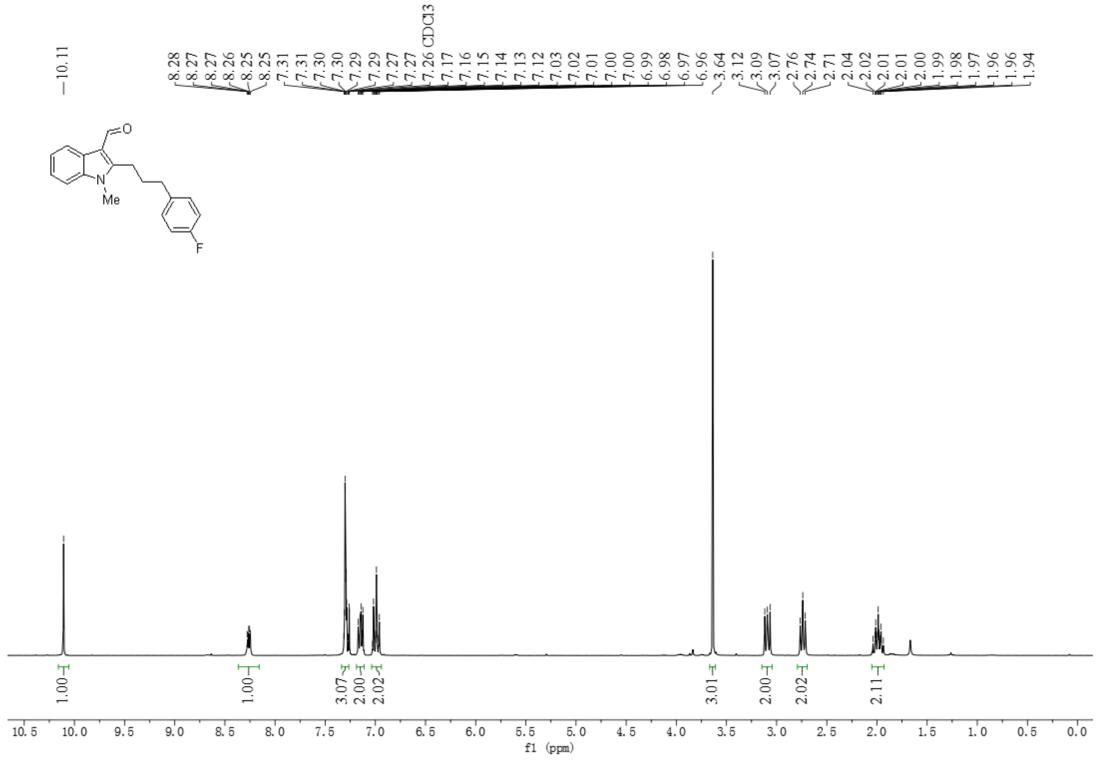

^{13}C NMR spectrum of **27** (75 MHz, $CDCl_3$)

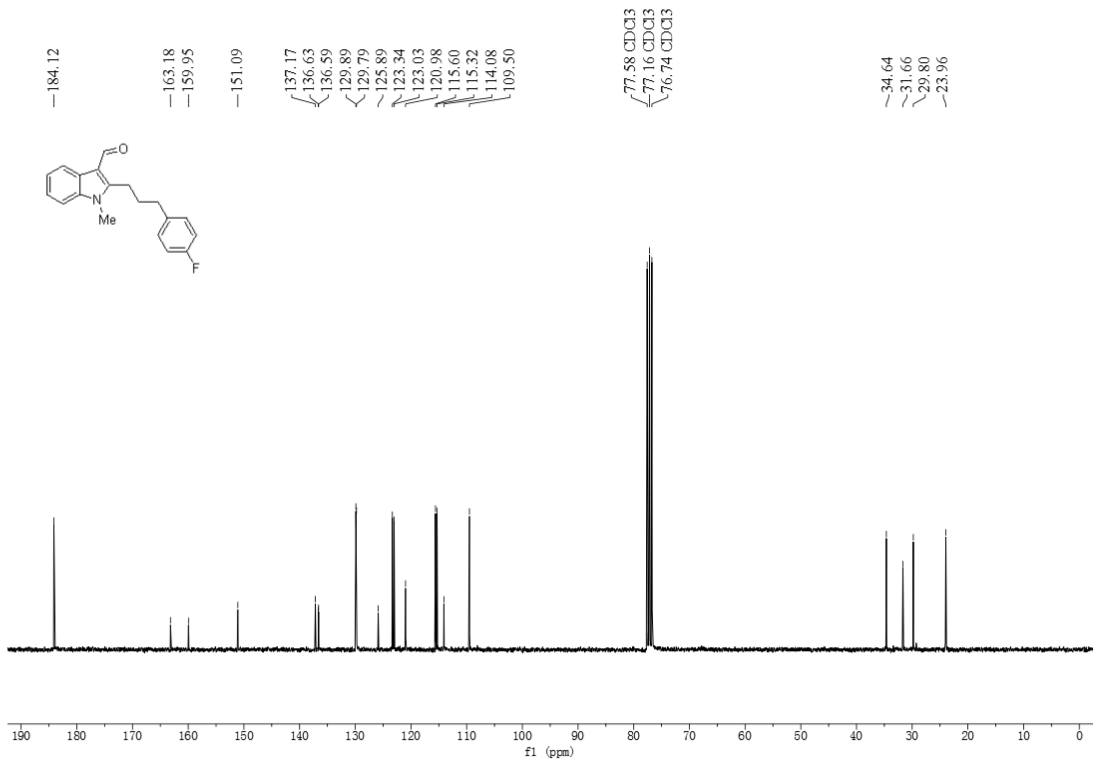

^{19}F NMR spectrum of **27** (282 MHz, $CDCl_3$)

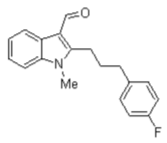

-116.93

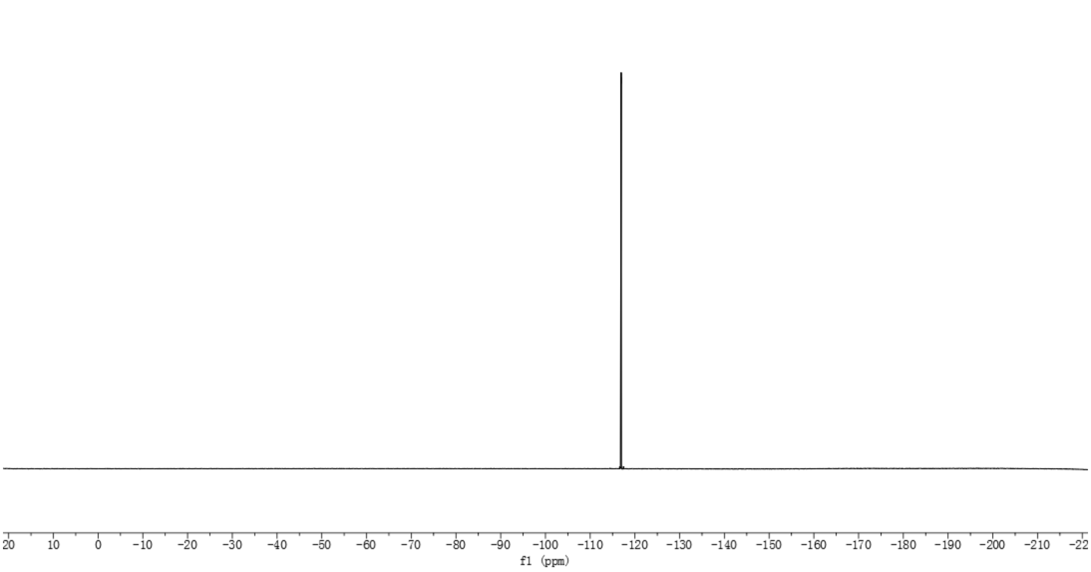

^{1}H NMR spectrum of **28** (300 MHz, $CDCl_3$)

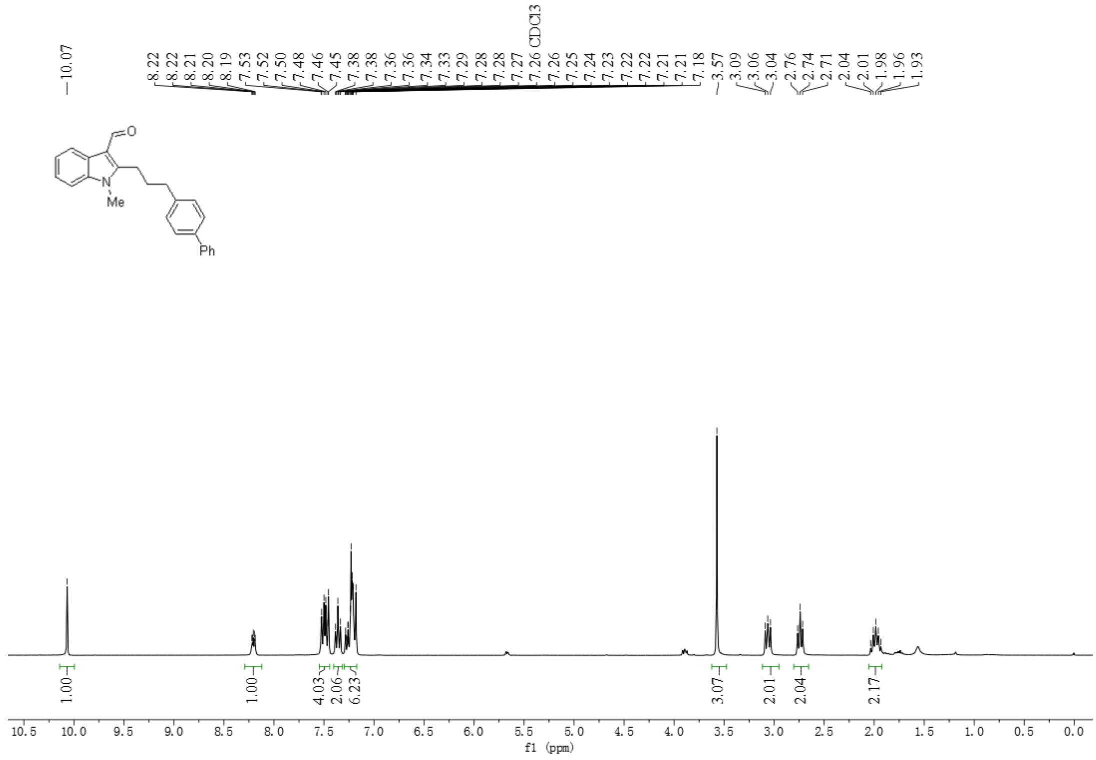

^{13}C NMR spectrum of **28** (75 MHz, $CDCl_3$)

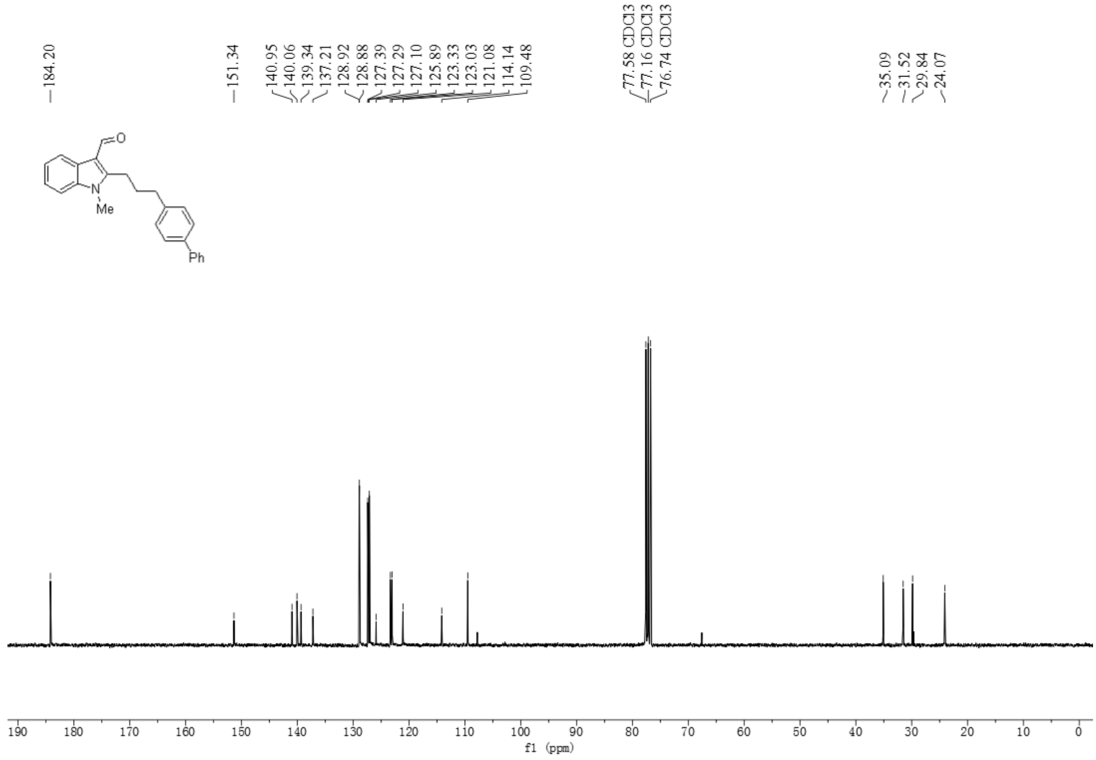

^{1}H NMR spectrum of **29** (300 MHz, $CDCl_3$)

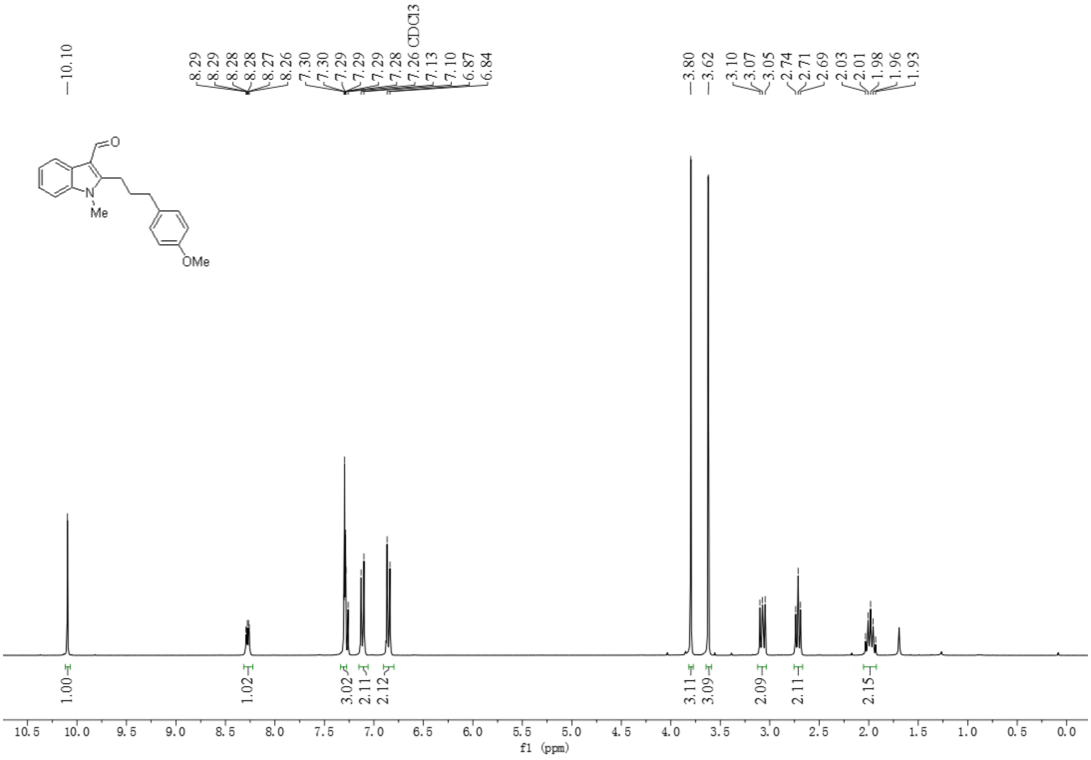

^{13}C NMR spectrum of **29** (75 MHz, $CDCl_3$)

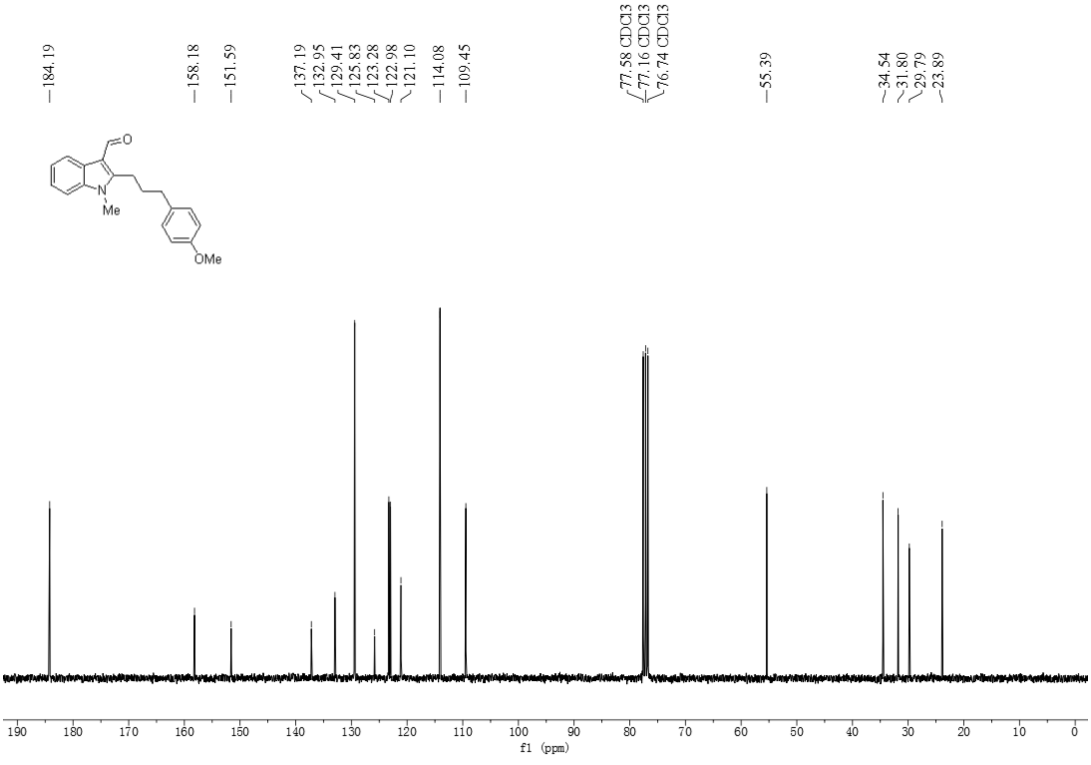

^{1}H NMR spectrum of **30** (300 MHz, $CDCl_3$)

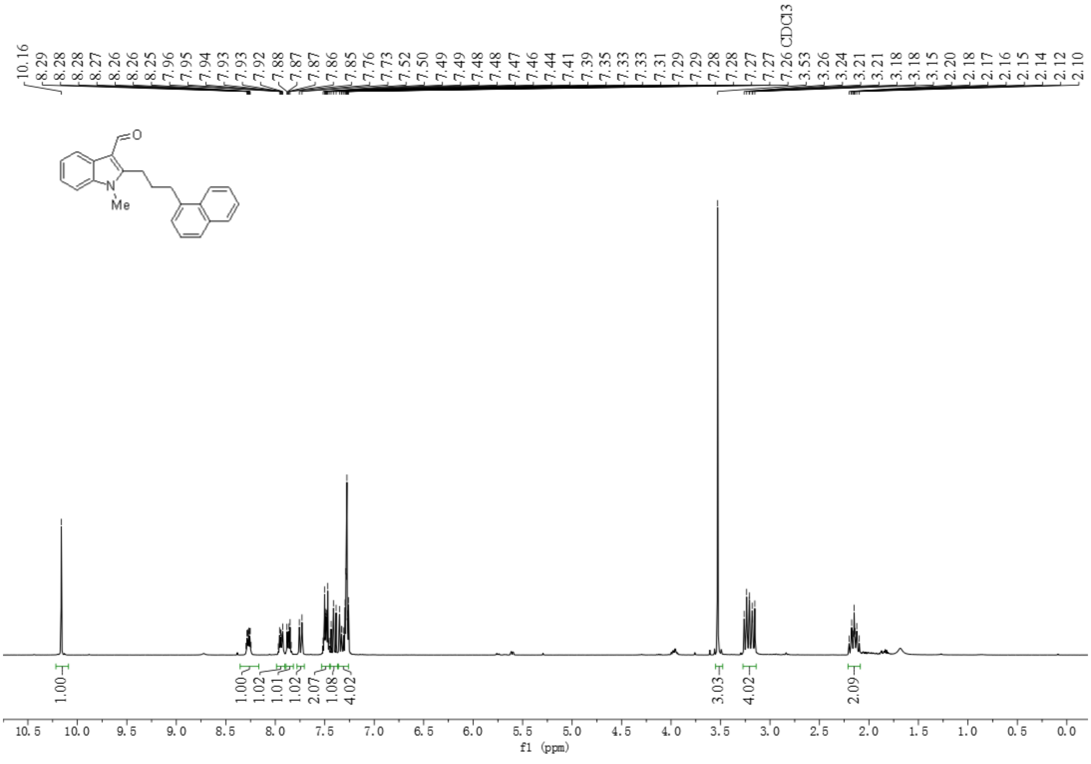

^{13}C NMR spectrum of **30** (75 MHz, $CDCl_3$)

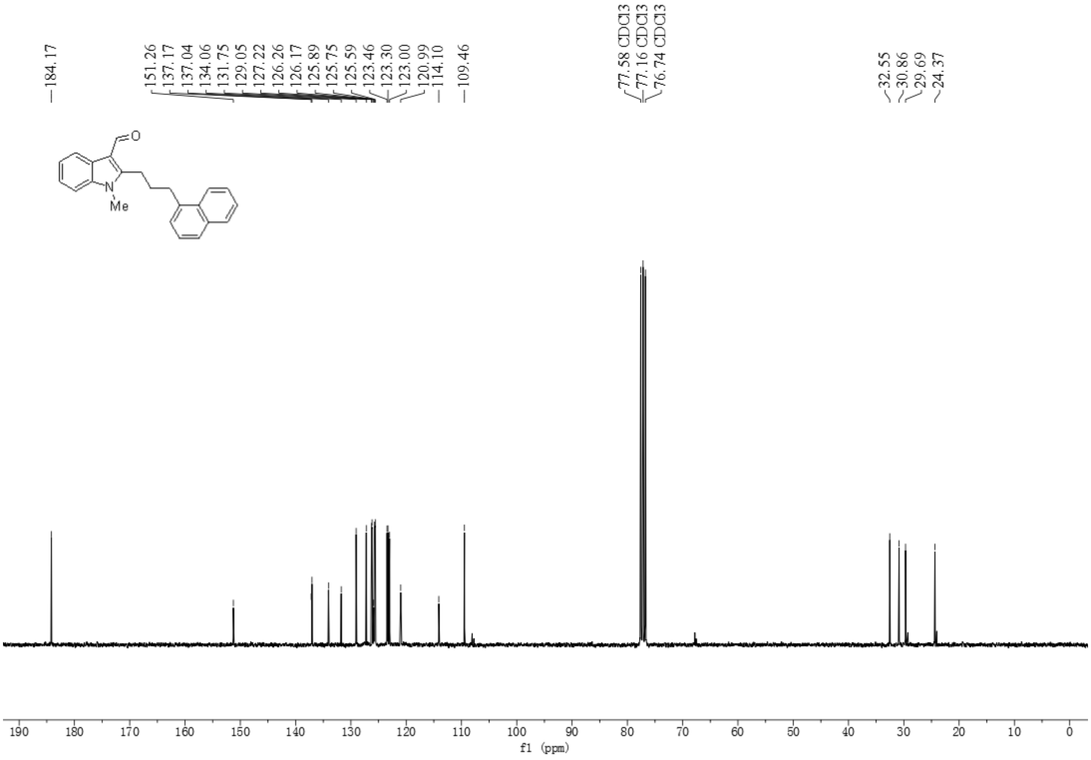

^{1}H NMR spectrum of **31** (300 MHz, $CDCl_3$)

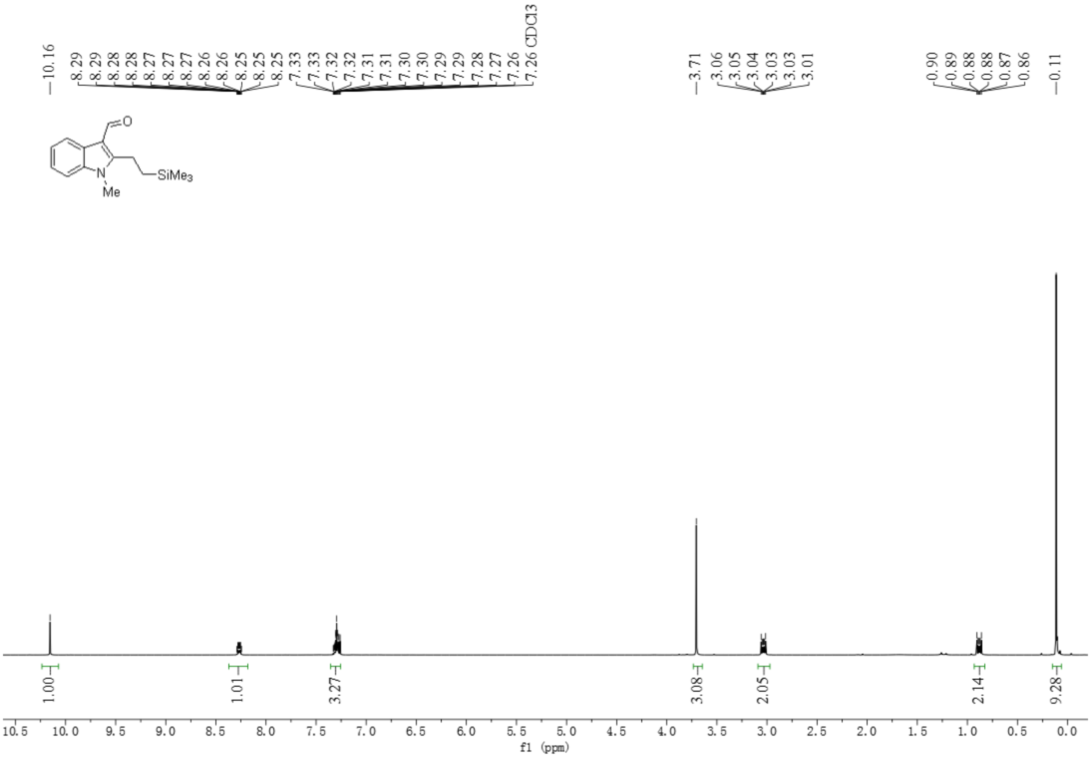

^{13}C NMR spectrum of **31** (75 MHz, $CDCl_3$)

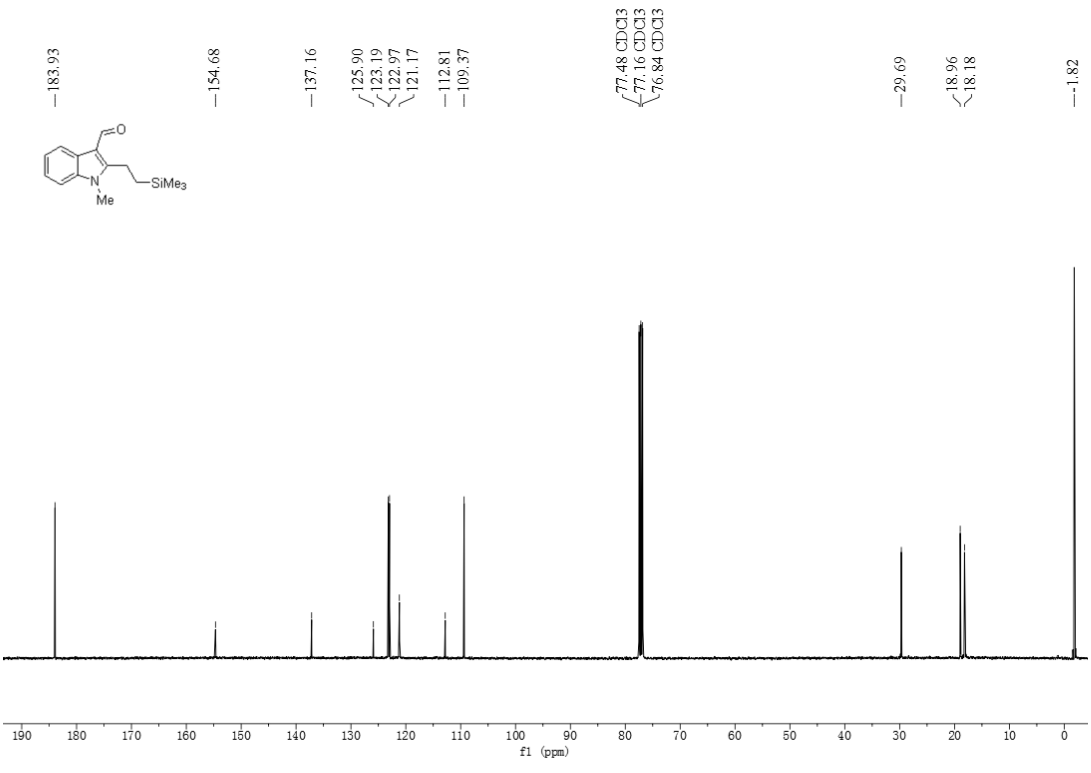

^{1}H NMR spectrum of **32** (300 MHz, $CDCl_3$)

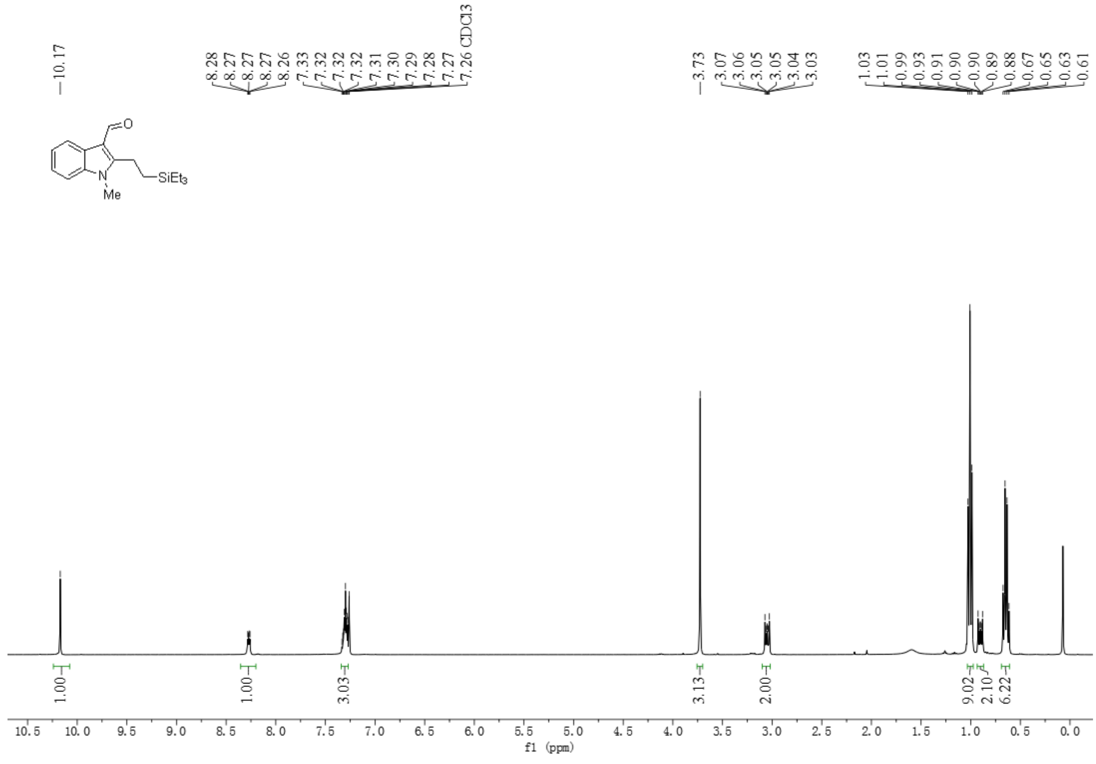

^{13}C NMR spectrum of **32** (75 MHz, $CDCl_3$)

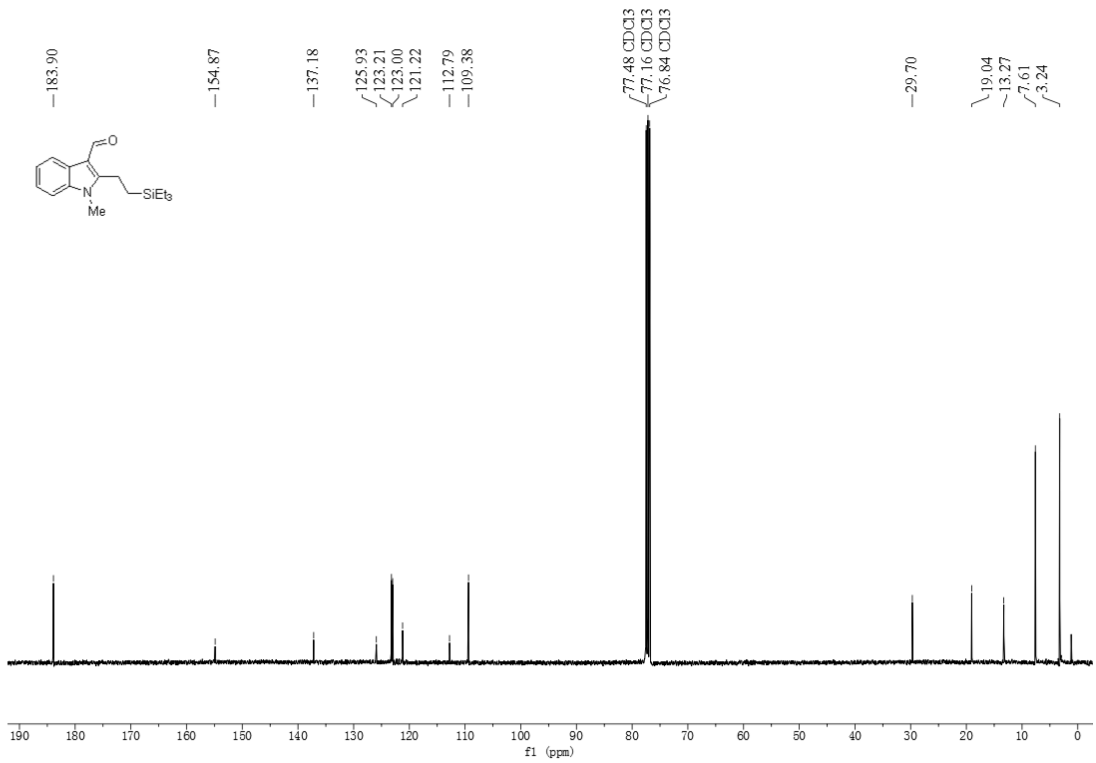

^1H NMR spectrum of **33** (300 MHz, $CDCl_3$)

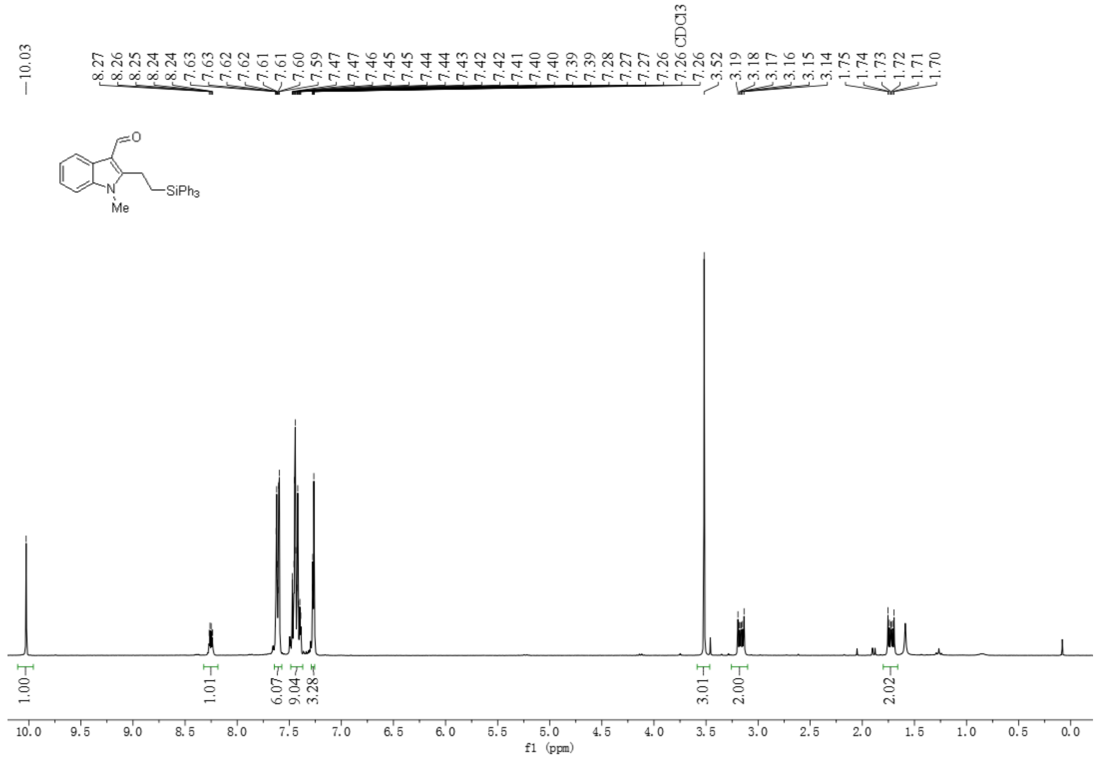

^{13}C NMR spectrum of **33** (75 MHz, $CDCl_3$)

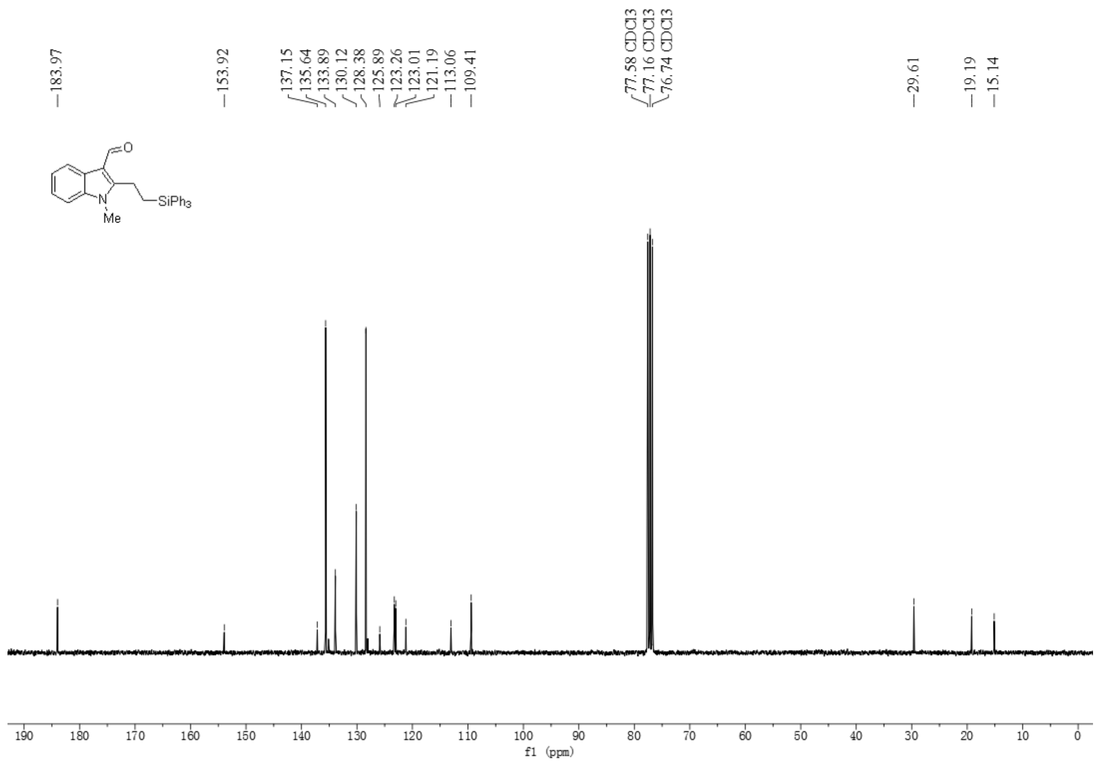

^{1}H NMR spectrum of **34** (300 MHz, $CDCl_3$)

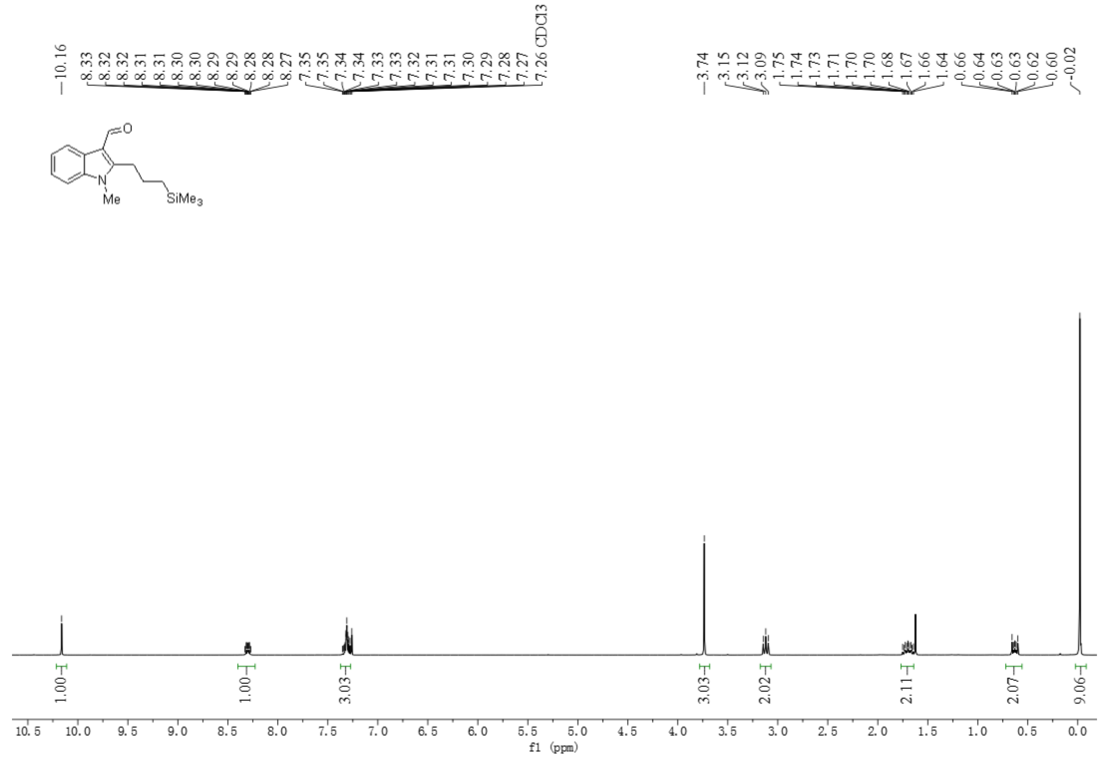

^{13}C NMR spectrum of **34** (75 MHz, $CDCl_3$)

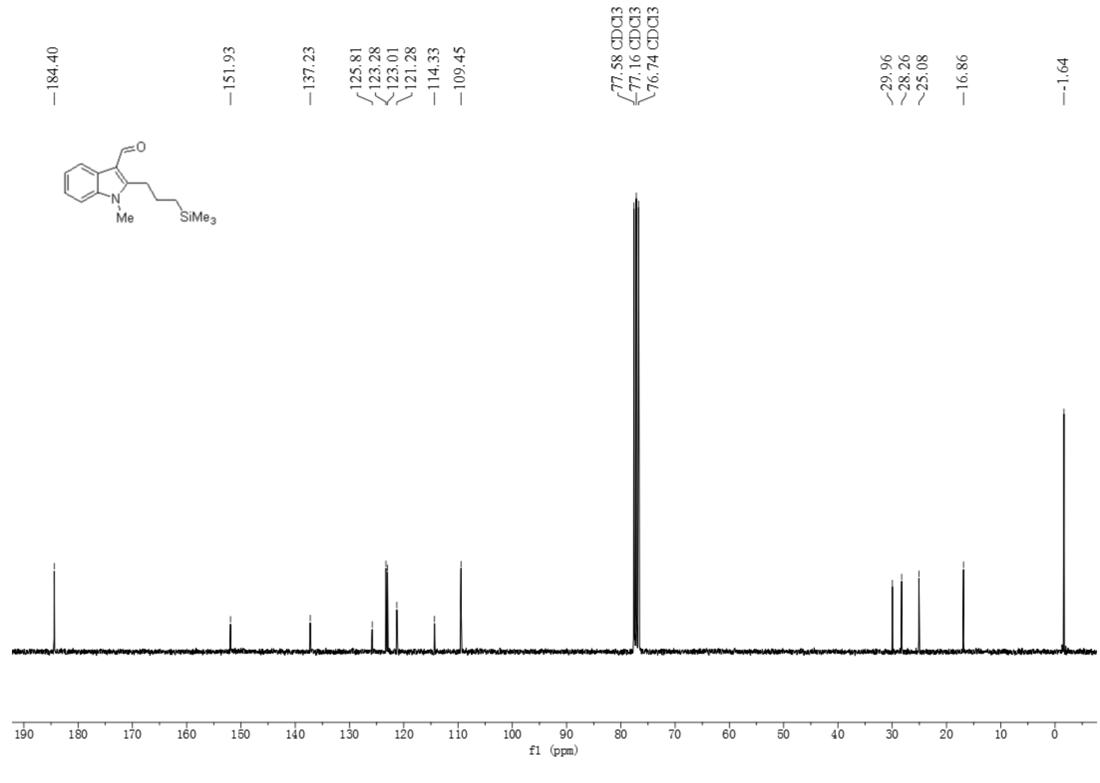

^{1}H NMR spectrum of **35** (300 MHz, $CDCl_3$)

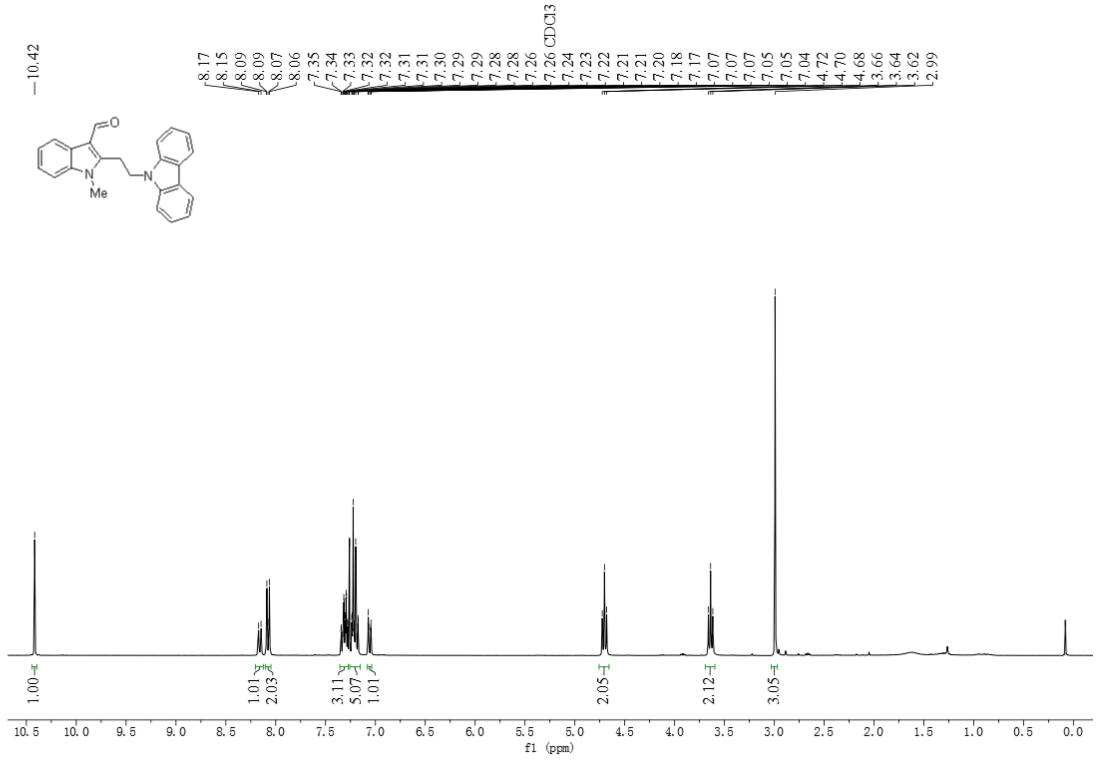

^{13}C NMR spectrum of **35** (75 MHz, $CDCl_3$)

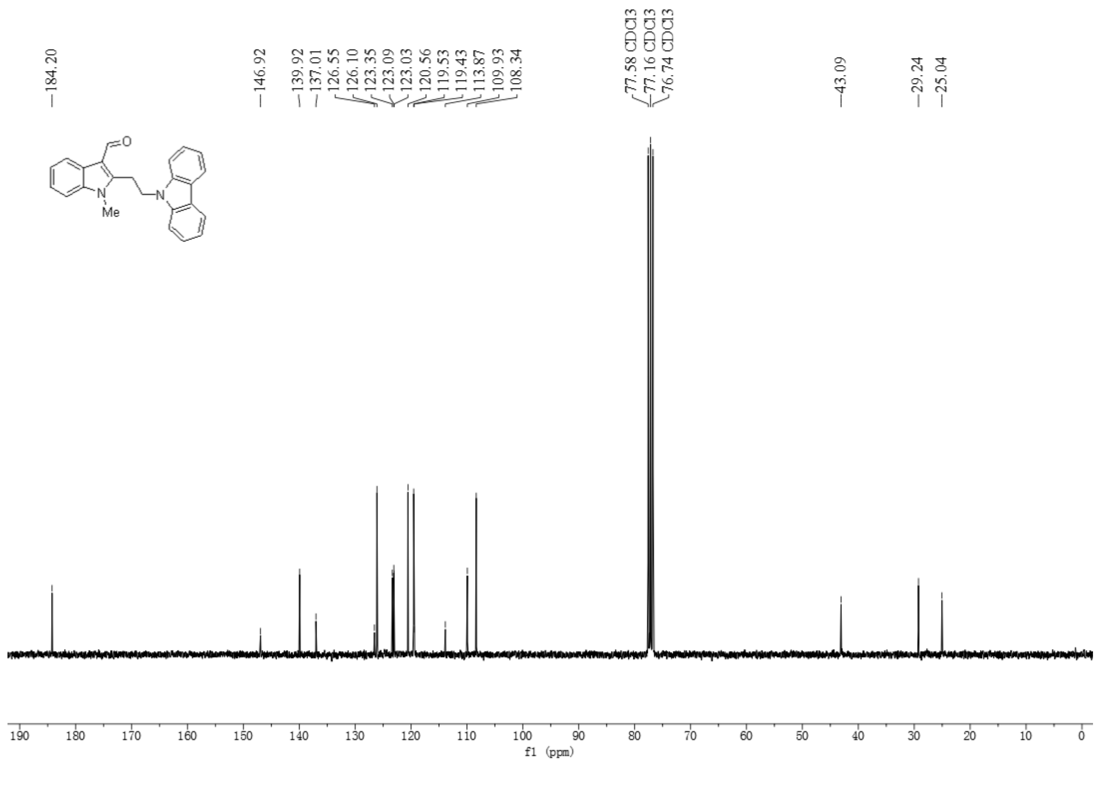

^{1}H NMR spectrum of **36** (300 MHz, $CDCl_3$)

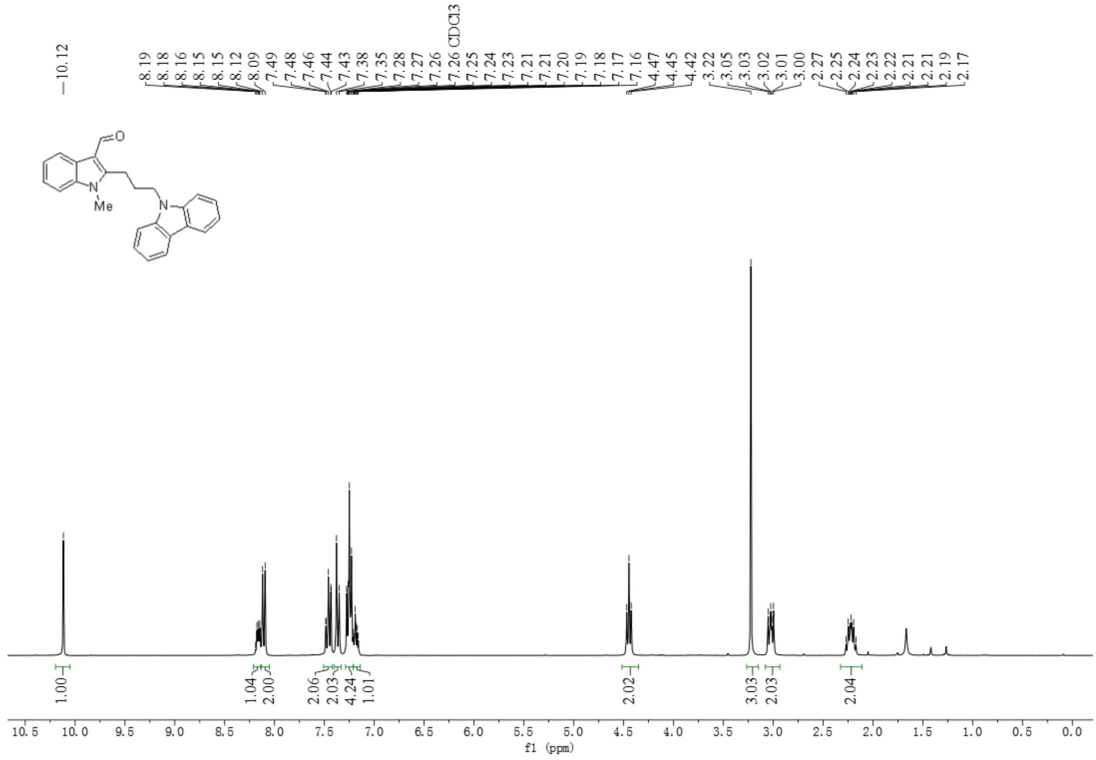

^{13}C NMR spectrum of **36** (75 MHz, $CDCl_3$)

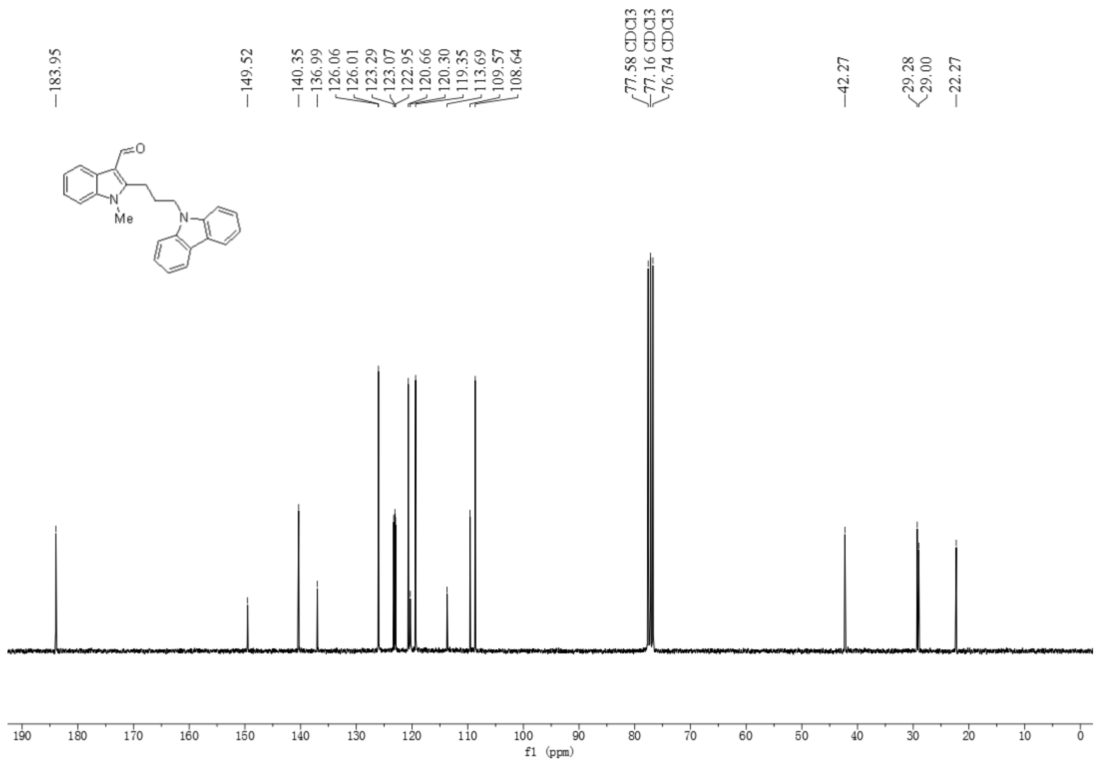

^{1}H NMR spectrum of **37** (300 MHz, $CDCl_3$)

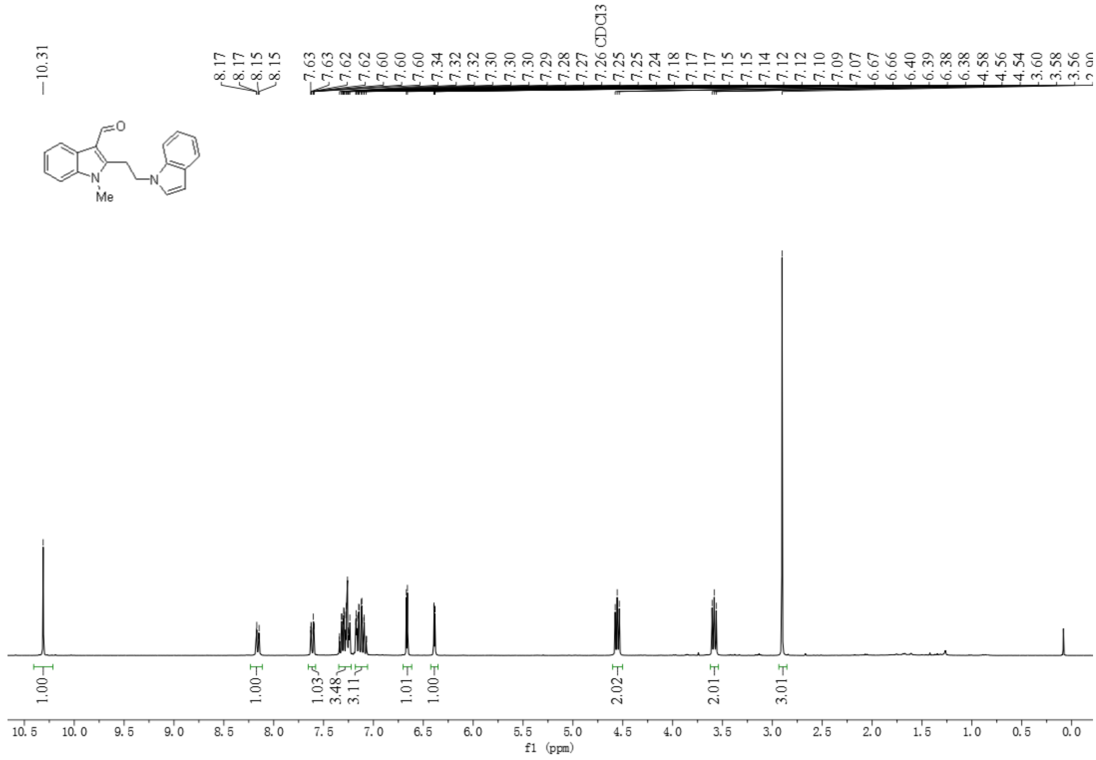

^{13}C NMR spectrum of **37** (75 MHz, $CDCl_3$)

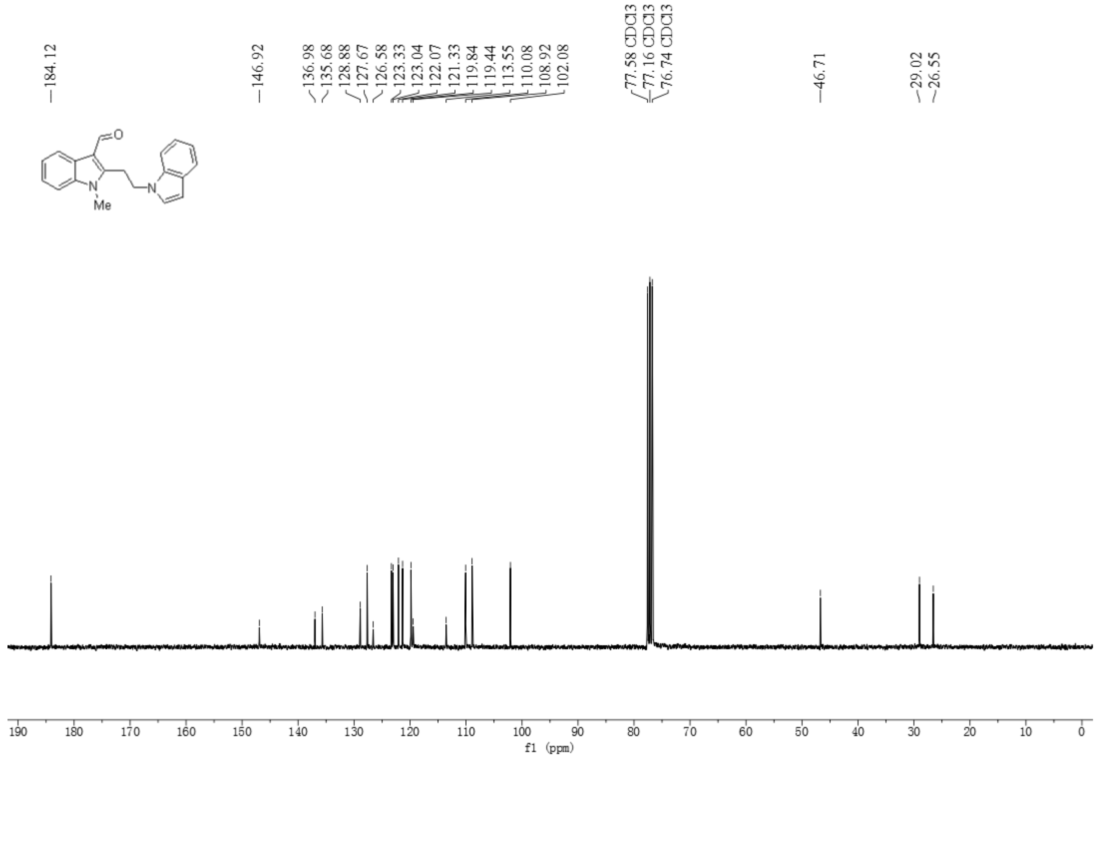

^{1}H NMR spectrum of **38** (300 MHz, $CDCl_3$)

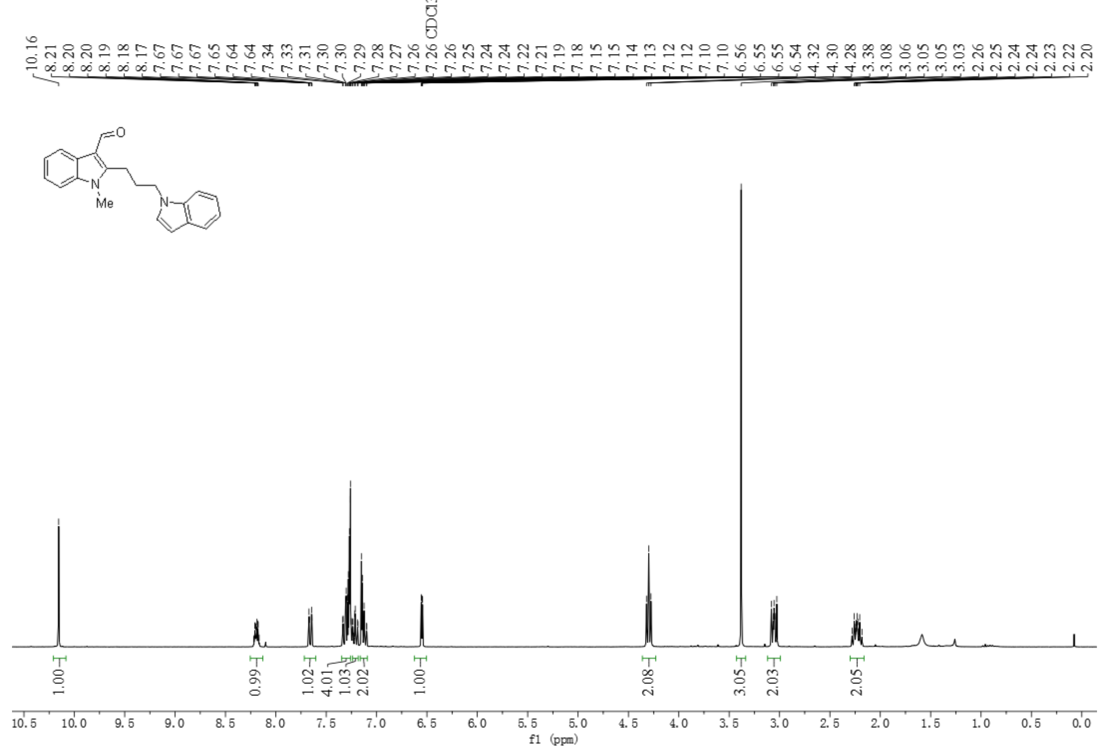

^{13}C NMR spectrum of **38** (75 MHz, $CDCl_3$)

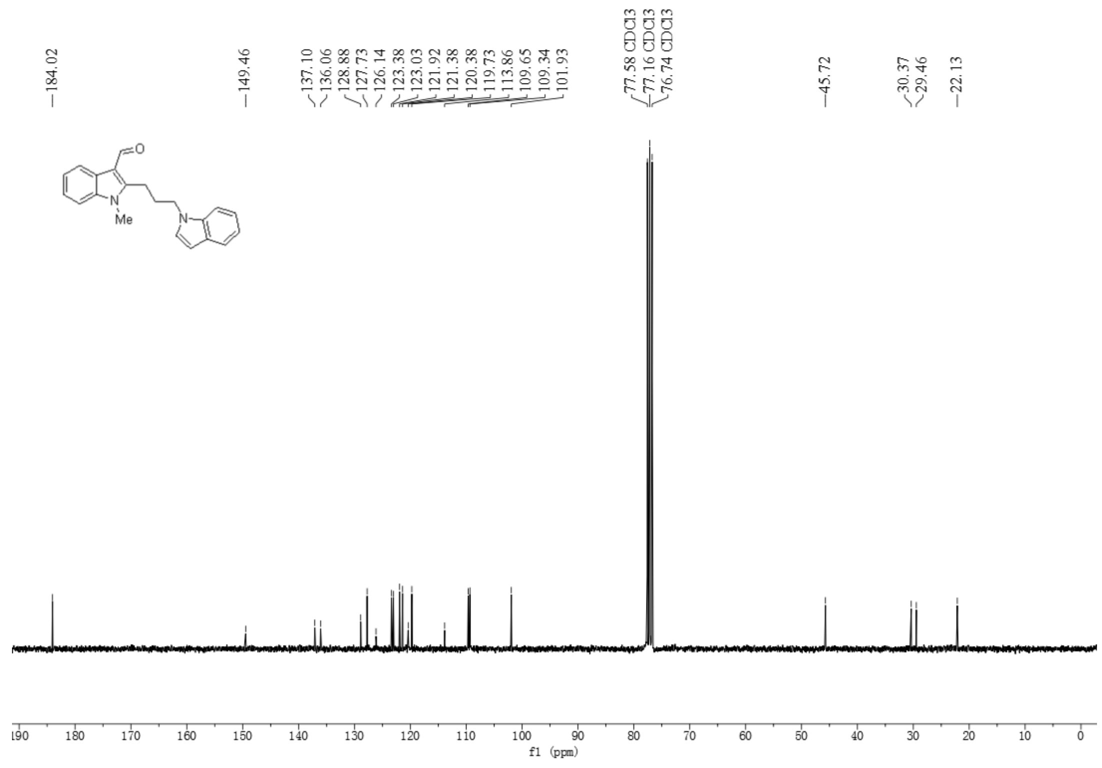

^{1}H NMR spectrum of **39** (300 MHz, $CDCl_3$)

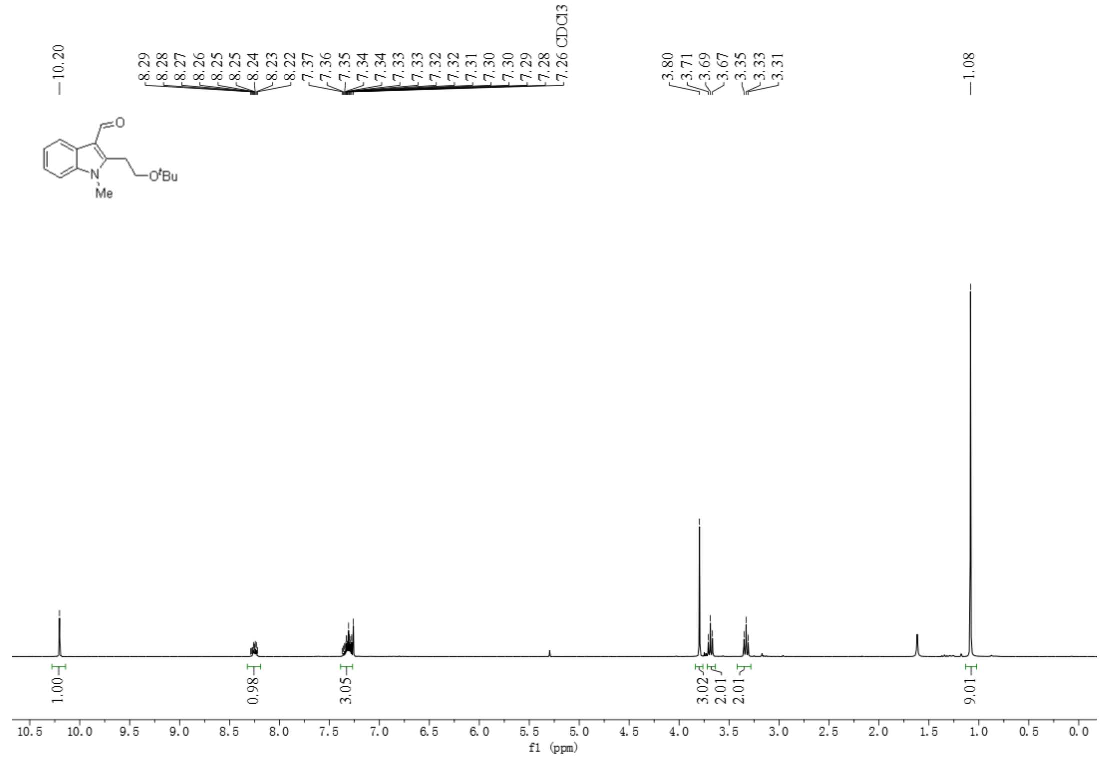

^{13}C NMR spectrum of **39** (75 MHz, $CDCl_3$)

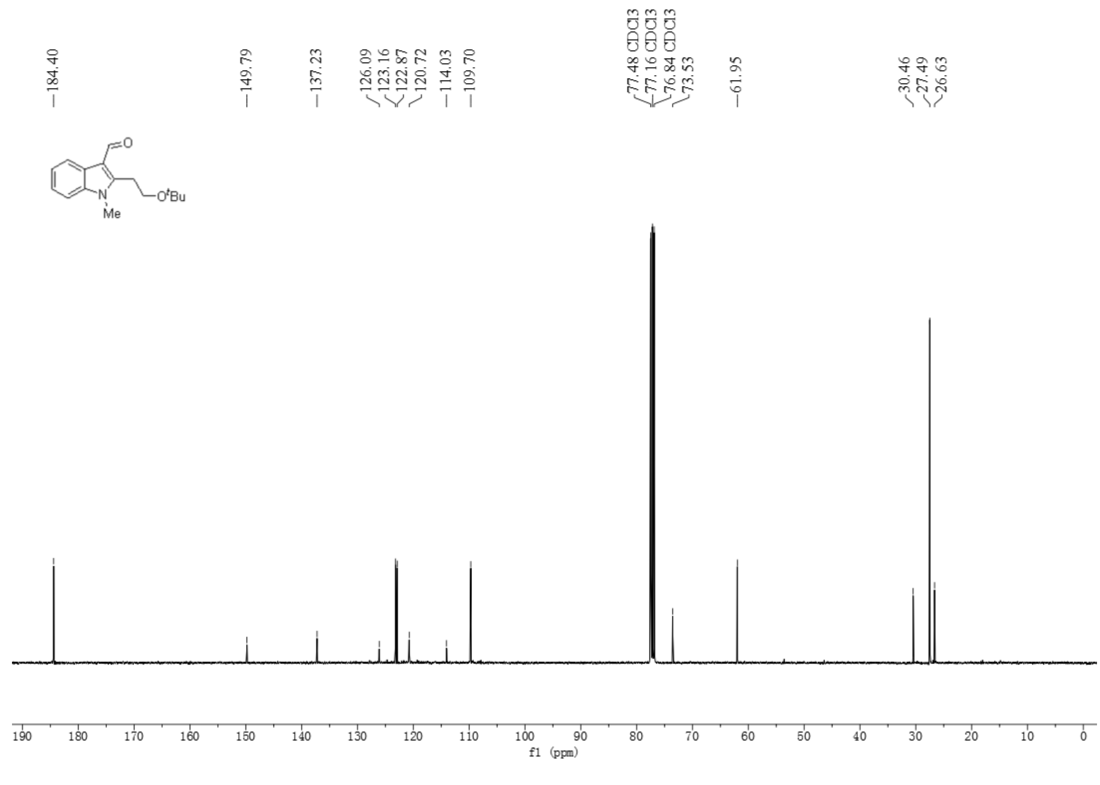

^{1}H NMR spectrum of **40** (300 MHz, $CDCl_3$)

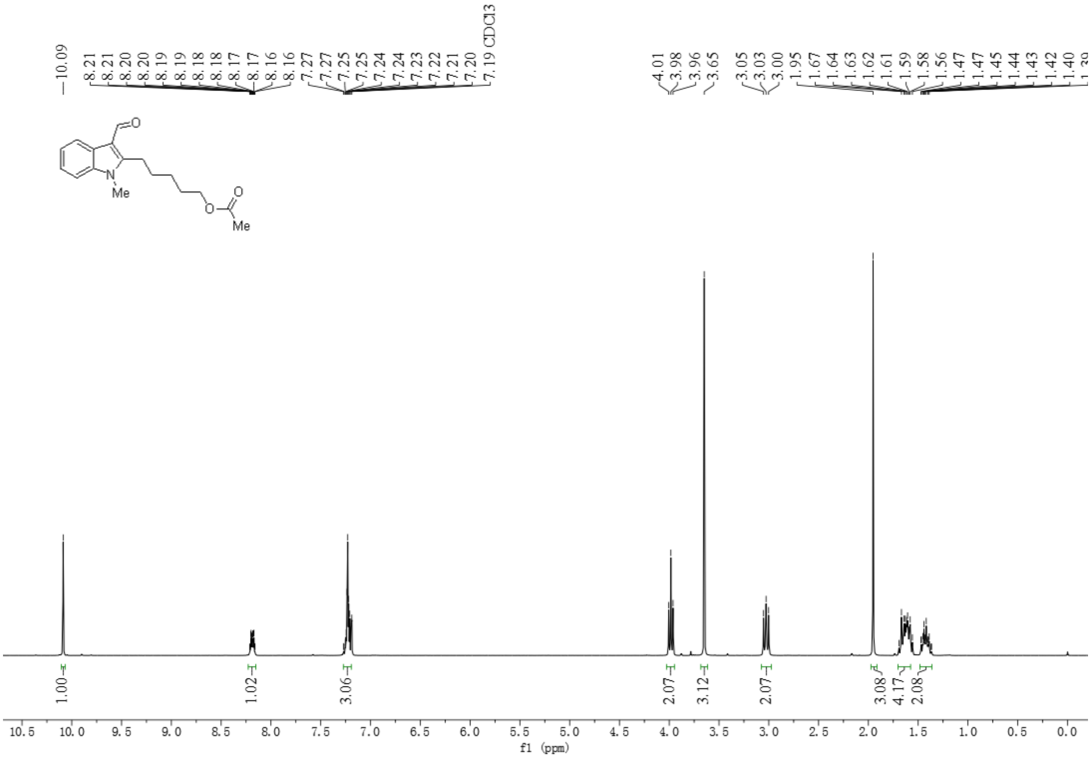

^{13}C NMR spectrum of **40** (75 MHz, $CDCl_3$)

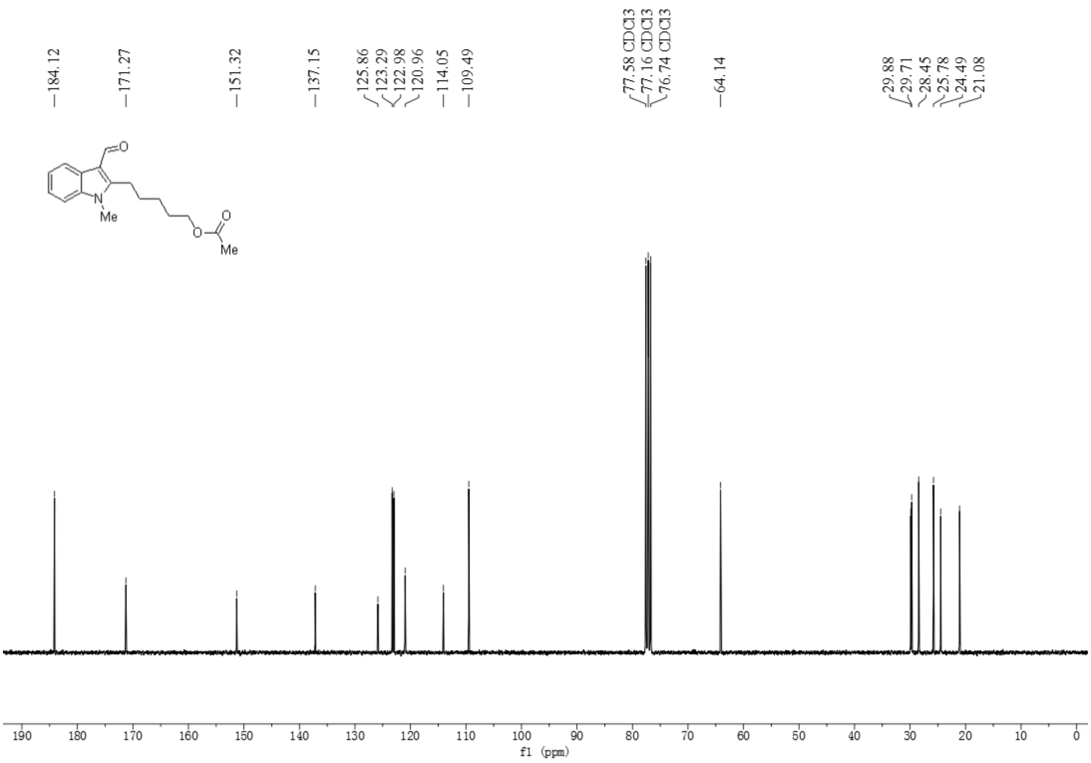

^{1}H NMR spectrum of **41** (300 MHz, $CDCl_3$)

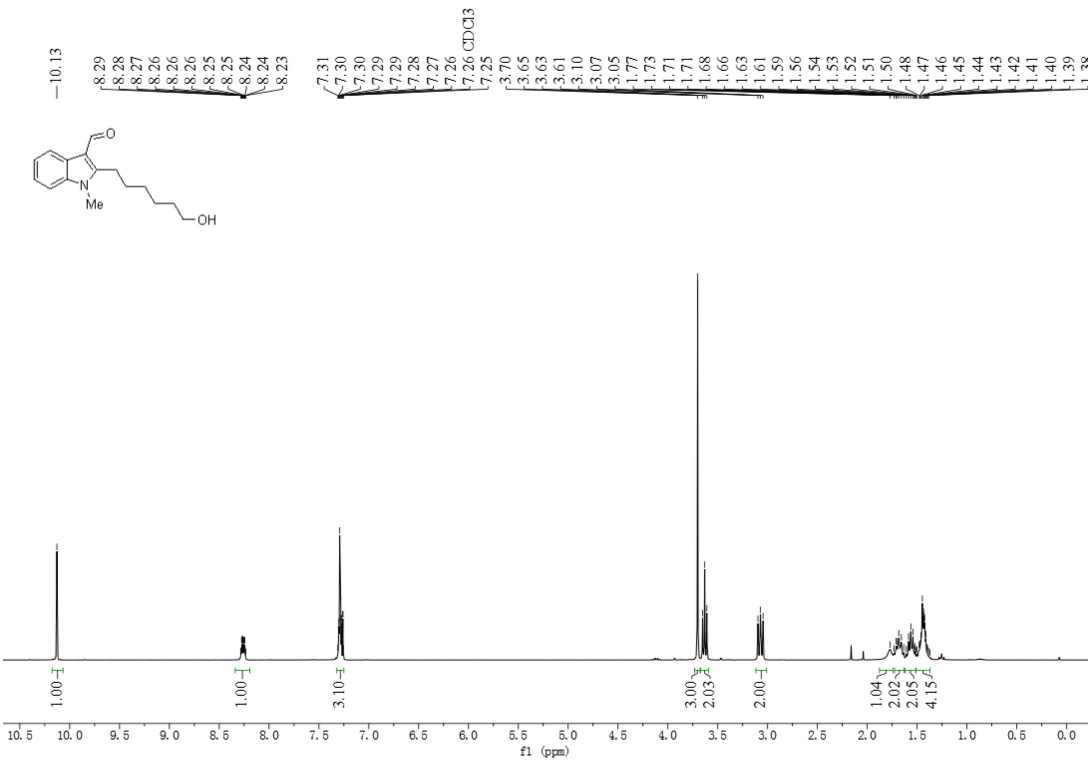

^{13}C NMR spectrum of **41** (75 MHz, $CDCl_3$)

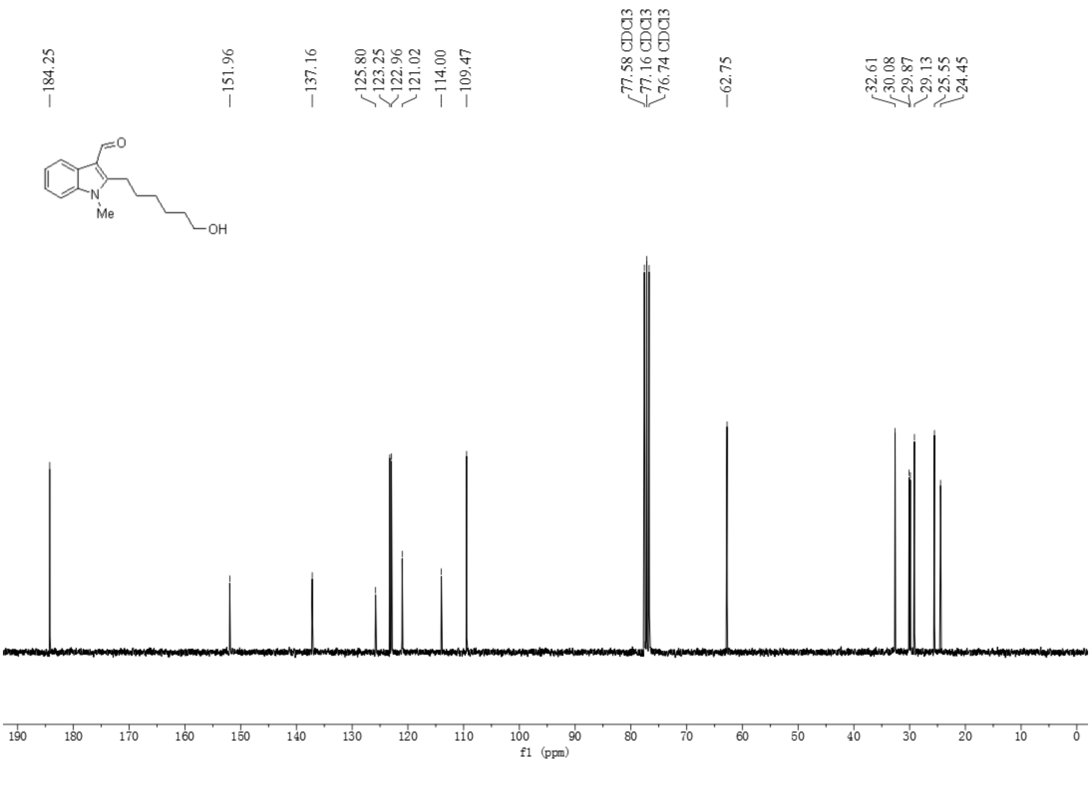

^{1}H NMR spectrum of **42** (300 MHz, $CDCl_3$)

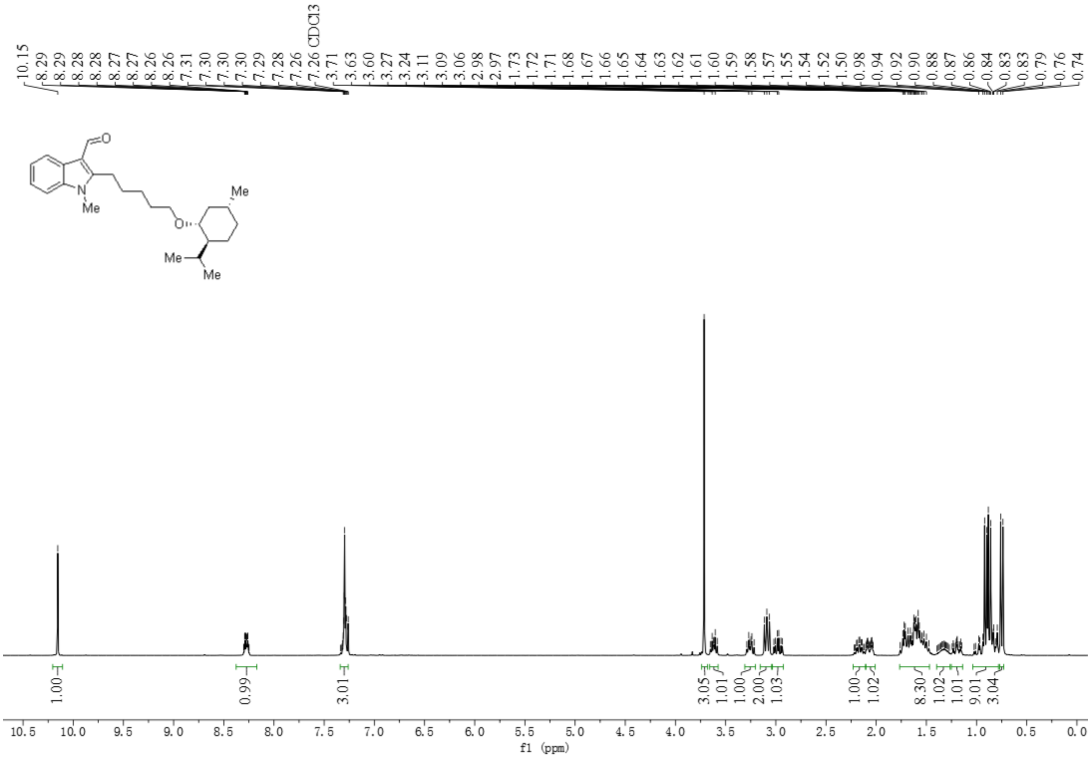

^{13}C NMR spectrum of **42** (75 MHz, $CDCl_3$)

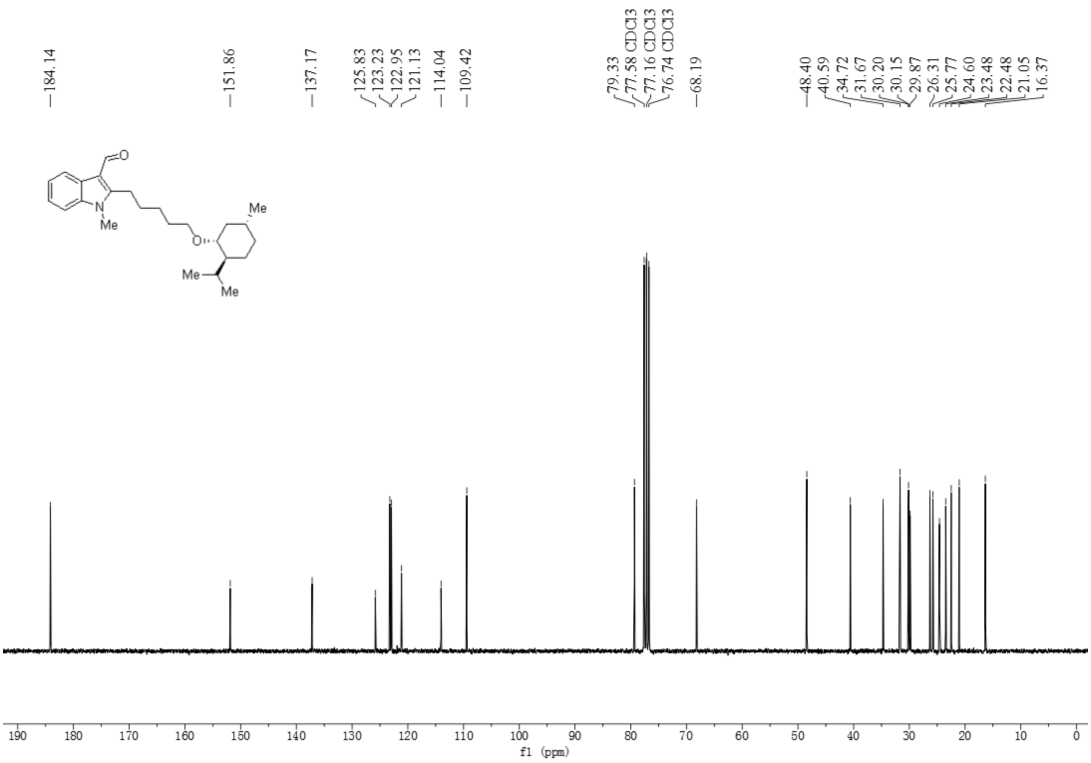

^{1}H NMR spectrum of **43** (300 MHz, $CDCl_3$)

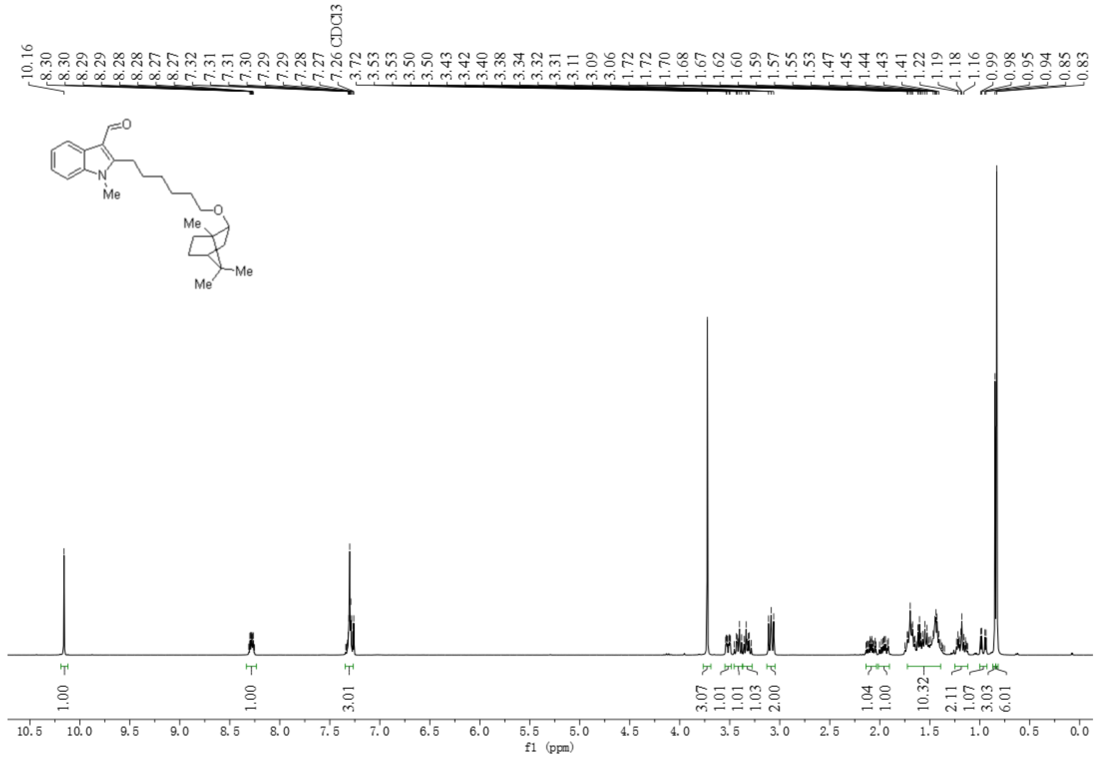

^{13}C NMR spectrum of **43** (75 MHz, $CDCl_3$)

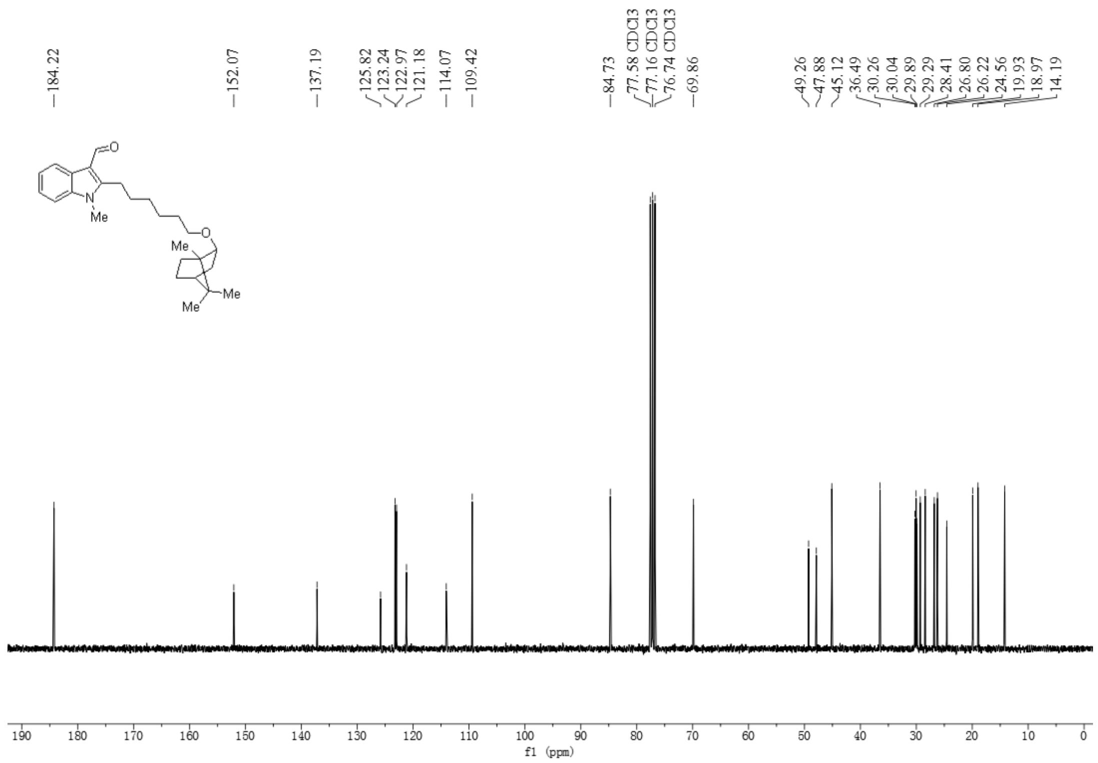

^{1}H NMR spectrum of **44** (300 MHz, $CDCl_3$)

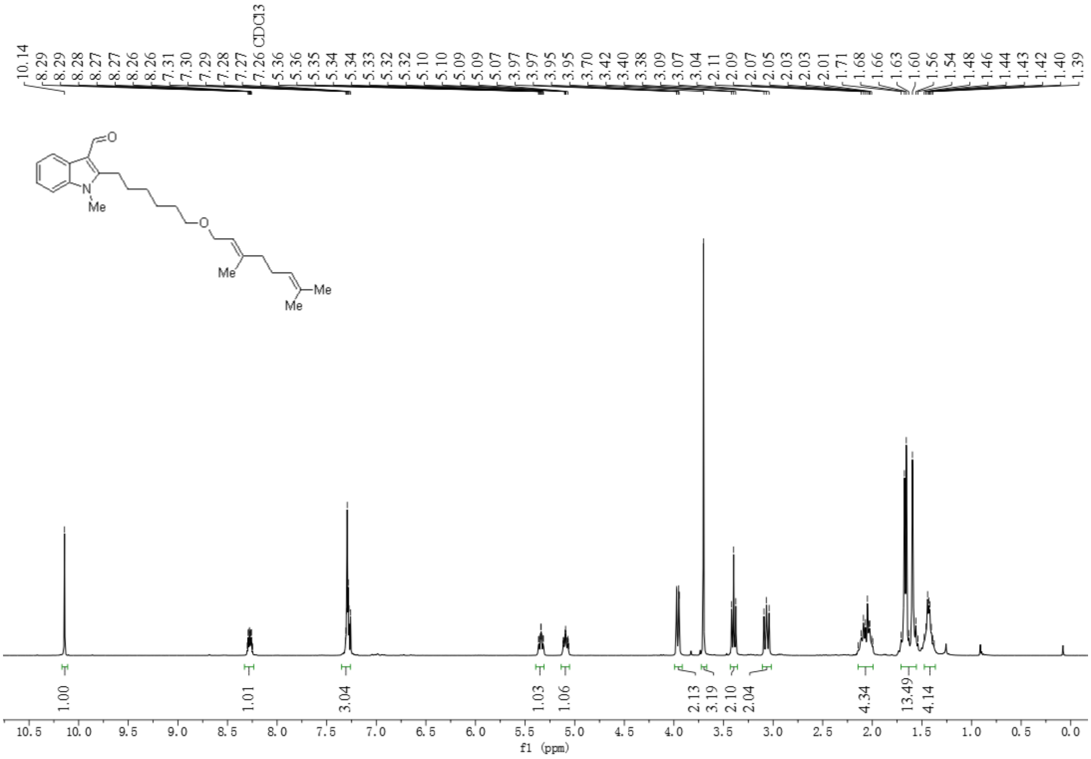

^{13}C NMR spectrum of **44** (75 MHz, $CDCl_3$)

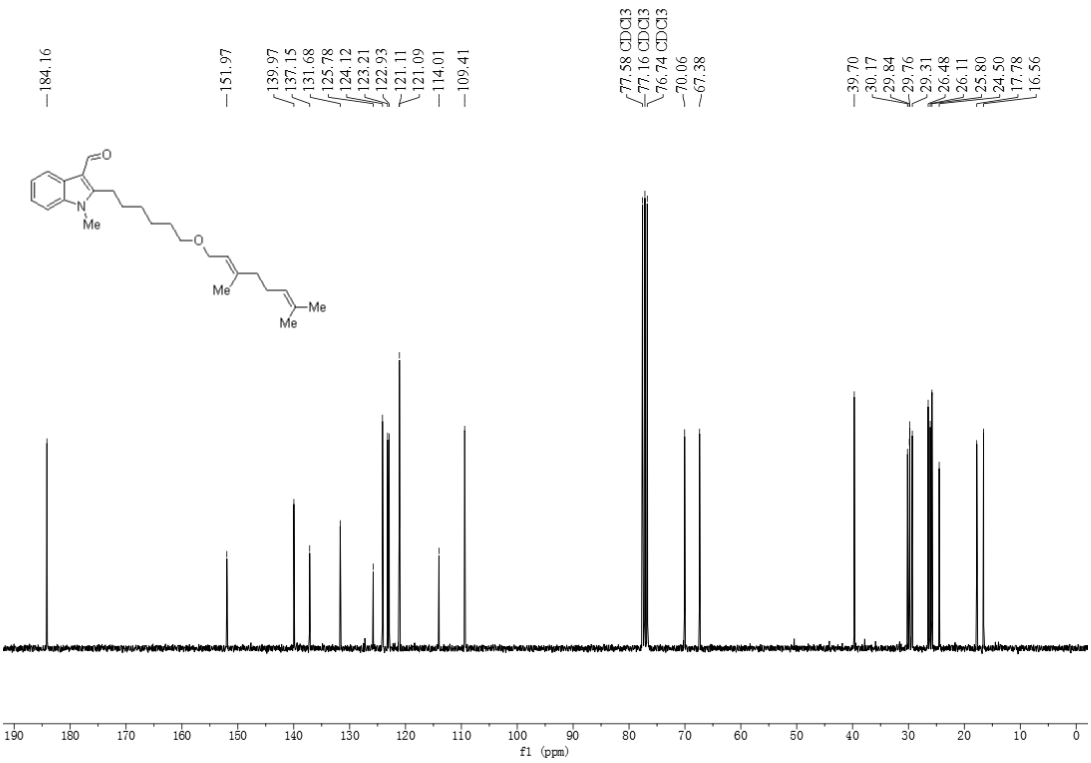

^{1}H NMR spectrum of **45** (300 MHz, $CDCl_3$)

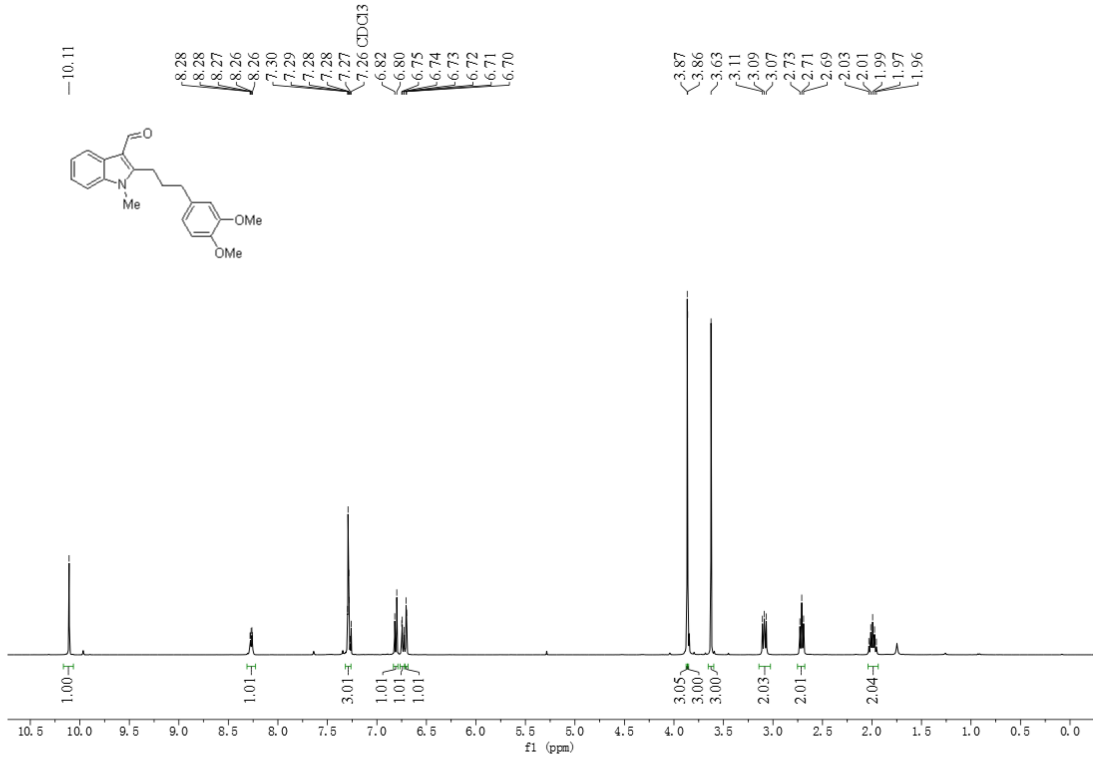

^{13}C NMR spectrum of **45** (75 MHz, $CDCl_3$)

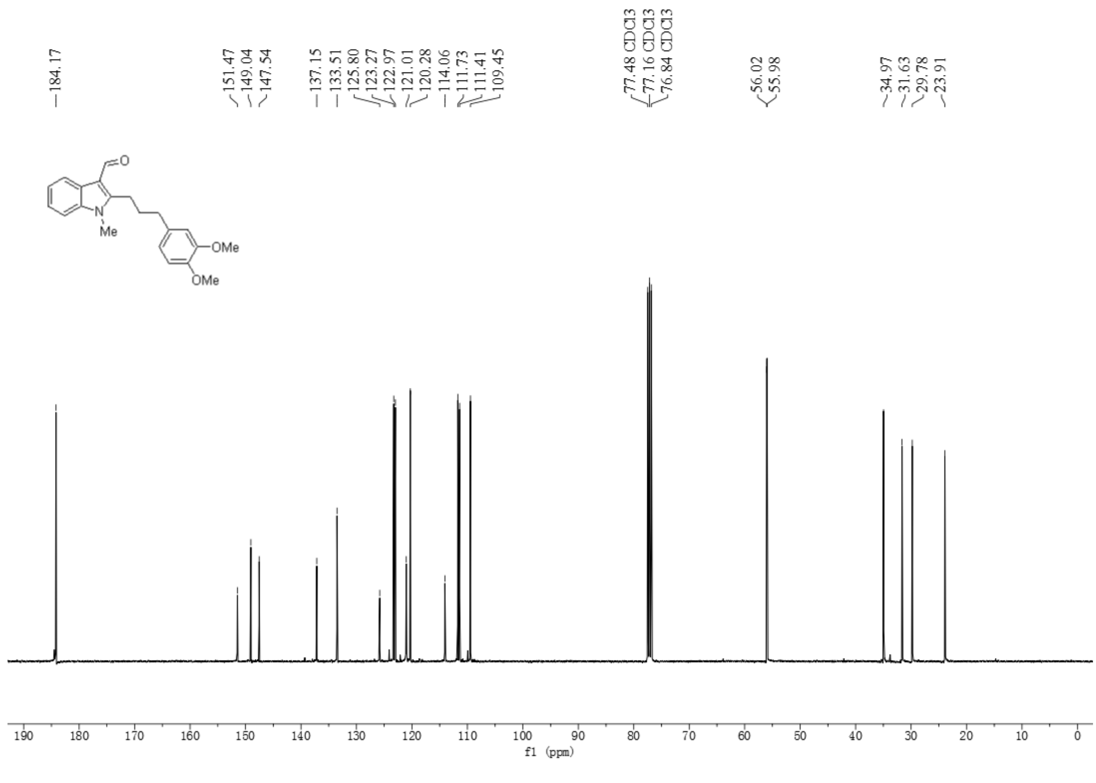

^{1}H NMR spectrum of **46** (300 MHz, $CDCl_3$)

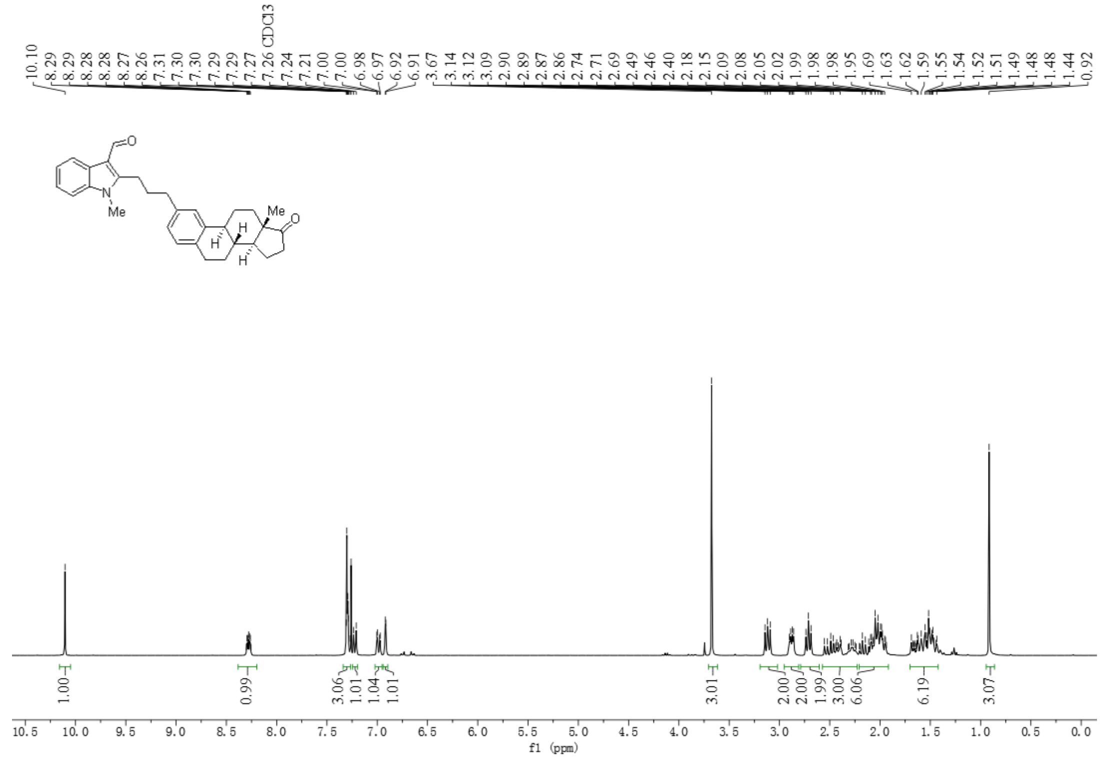

^{13}C NMR spectrum of **46** (75 MHz, $CDCl_3$)

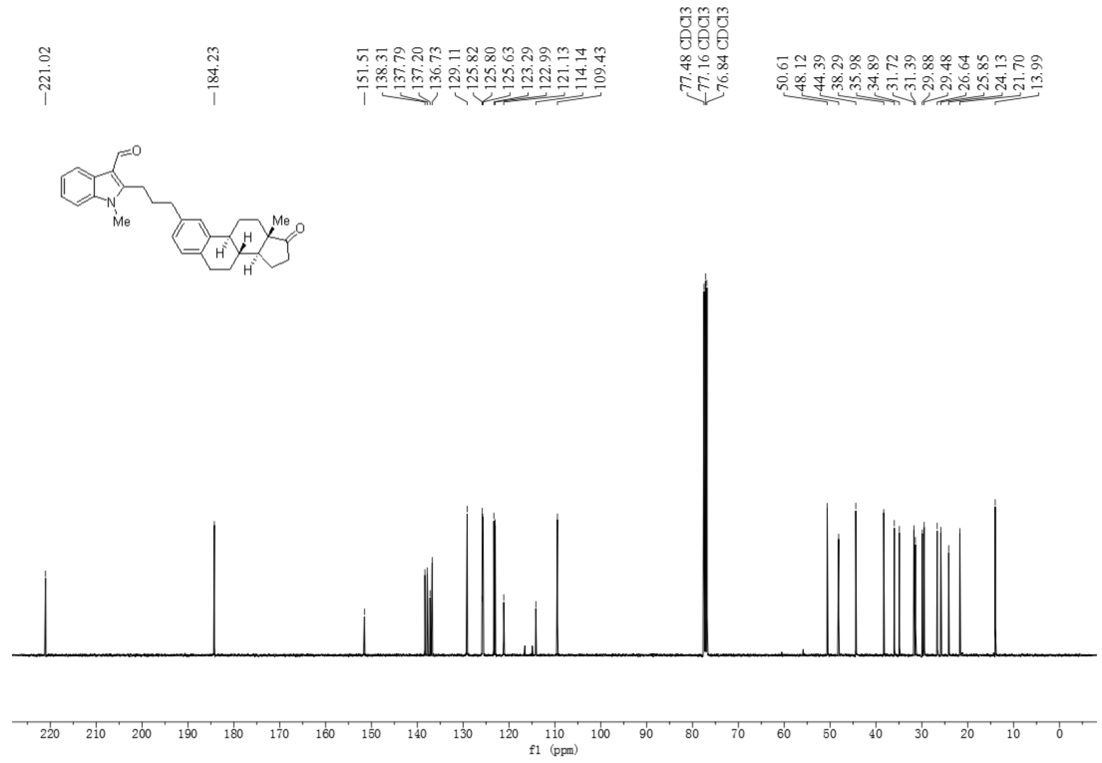

^{1}H NMR spectrum of **47** (300 MHz, $CDCl_3$)

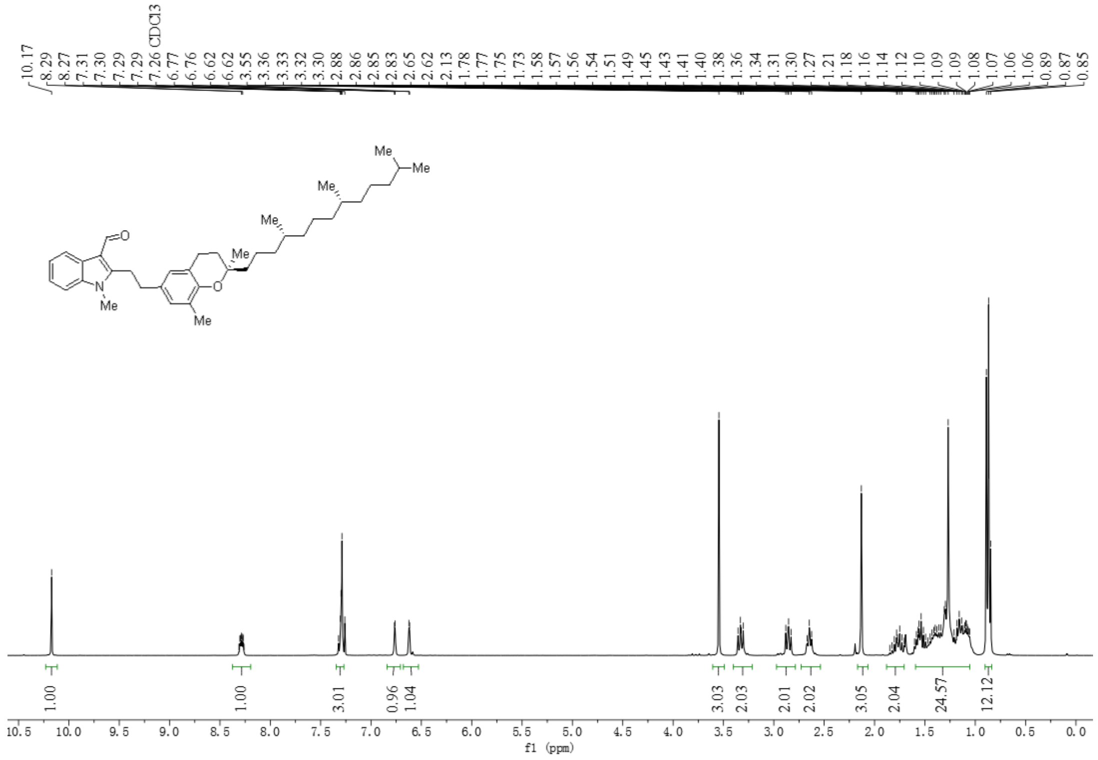

^{13}C NMR spectrum of **47** (75 MHz, $CDCl_3$)

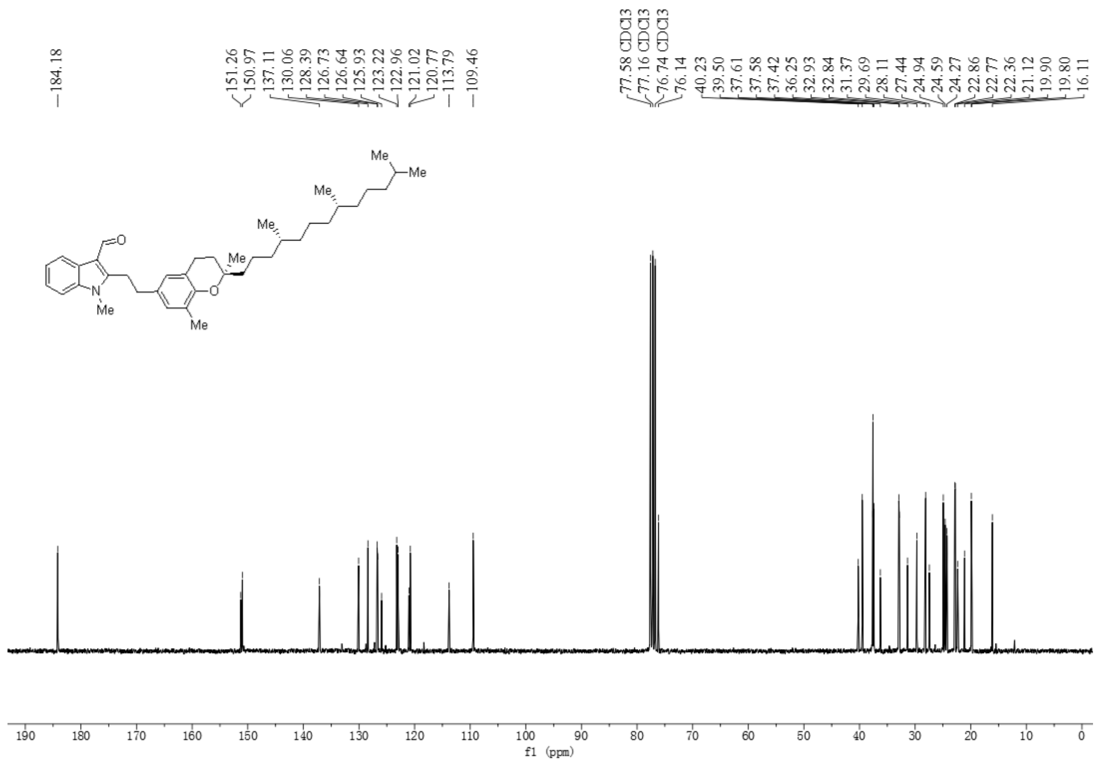

^{1}H NMR spectrum of **49** (300 MHz, $CDCl_3$)

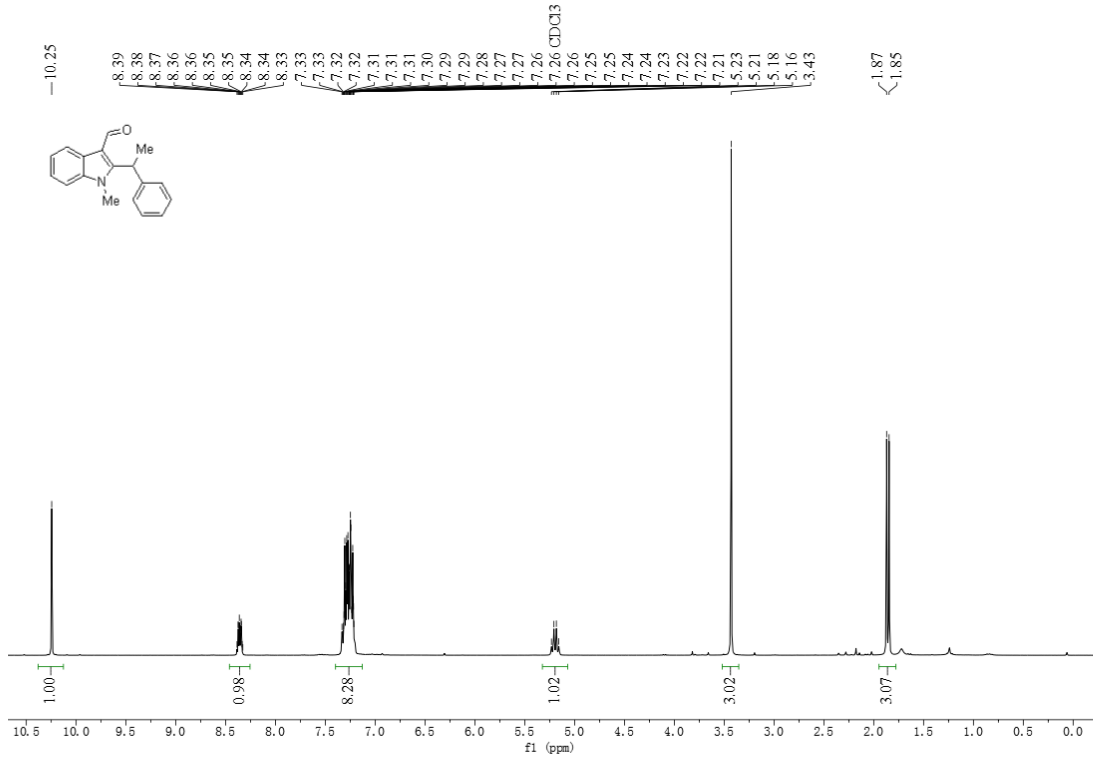

^{13}C NMR spectrum of **49** (75 MHz, $CDCl_3$)

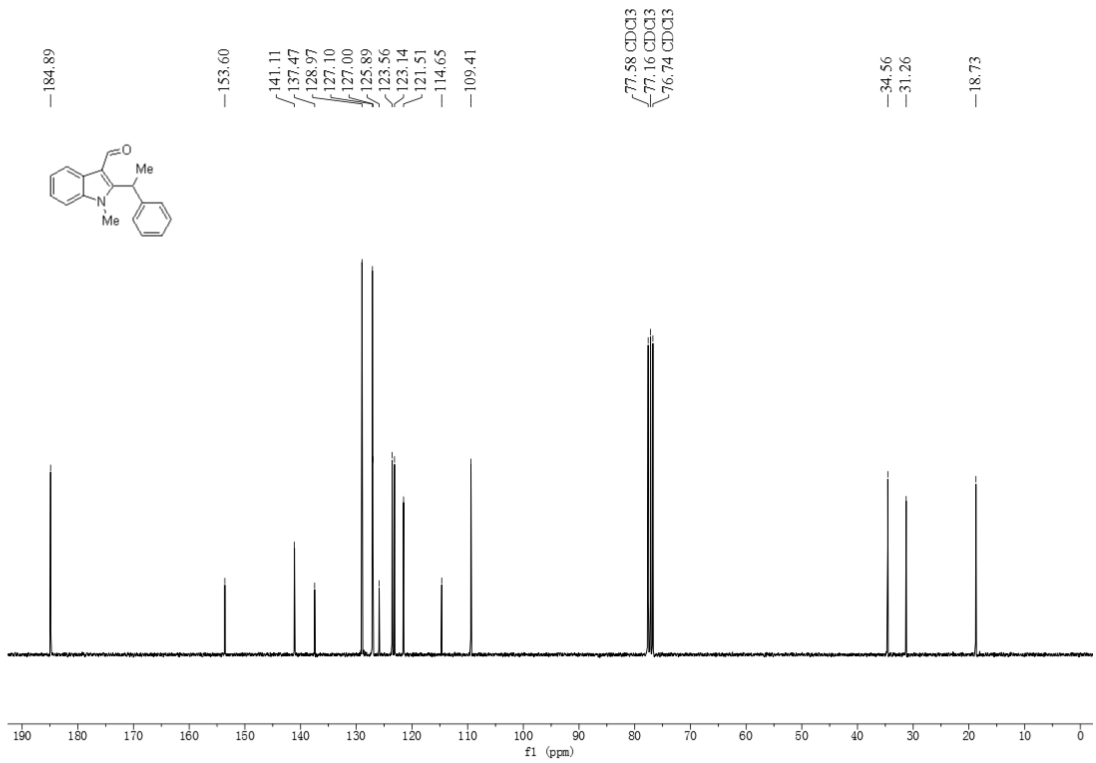

^{1}H NMR spectrum of **50** (300 MHz, $CDCl_3$)

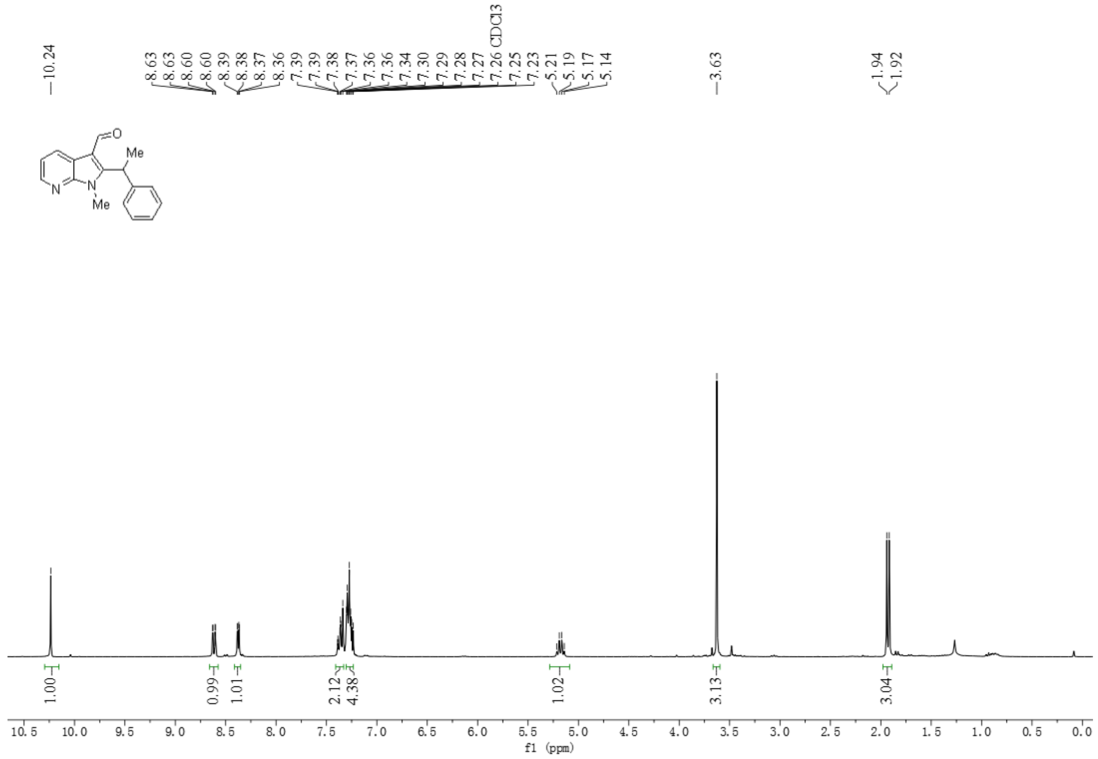

^{13}C NMR spectrum of **50** (75 MHz, $CDCl_3$)

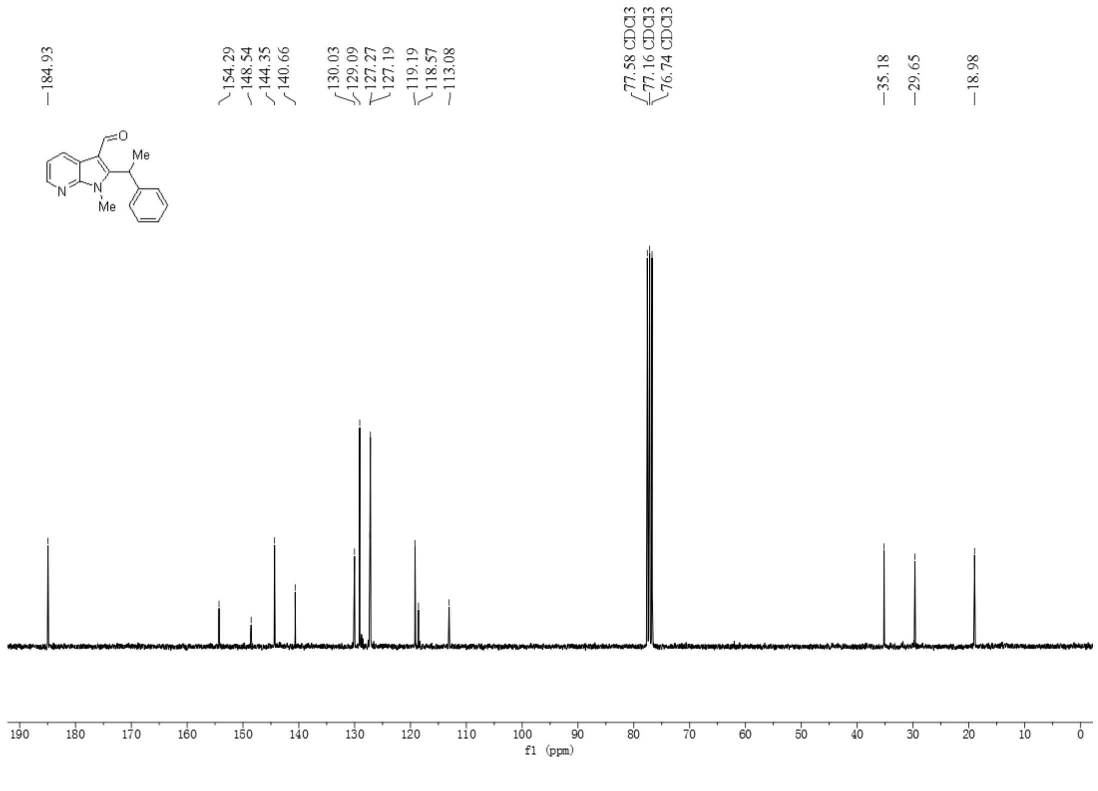

^{1}H NMR spectrum of **51** (300 MHz, $CDCl_3$)

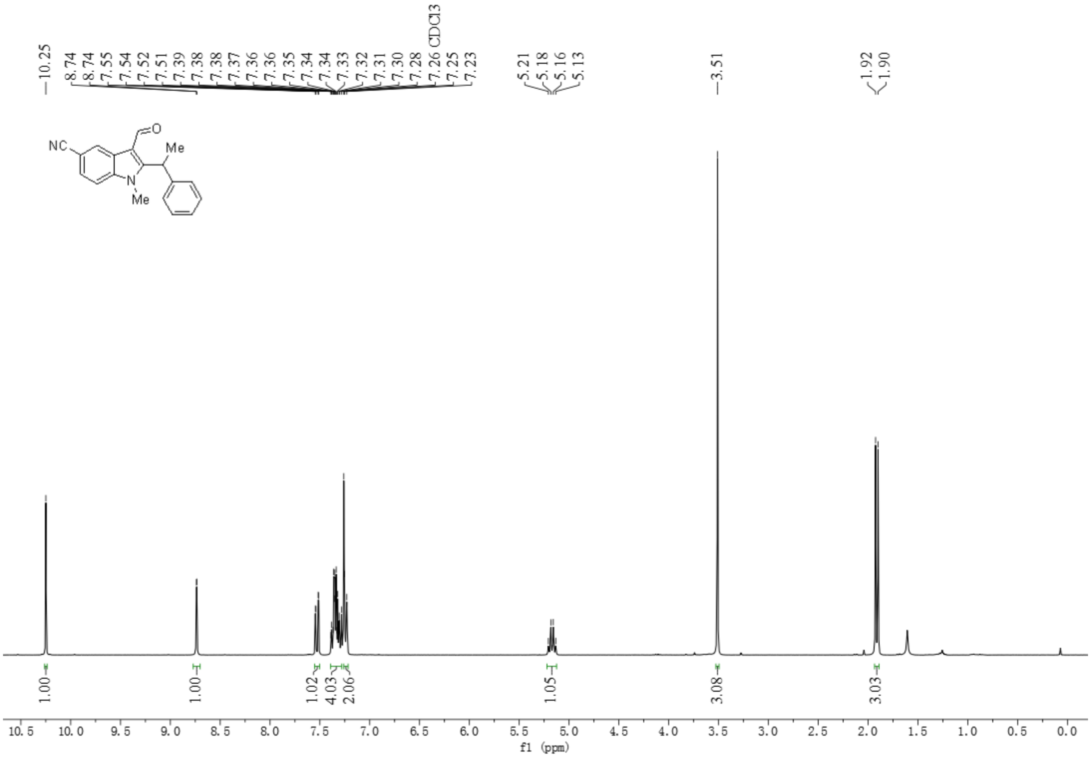

^{13}C NMR spectrum of **51** (75 MHz, $CDCl_3$)

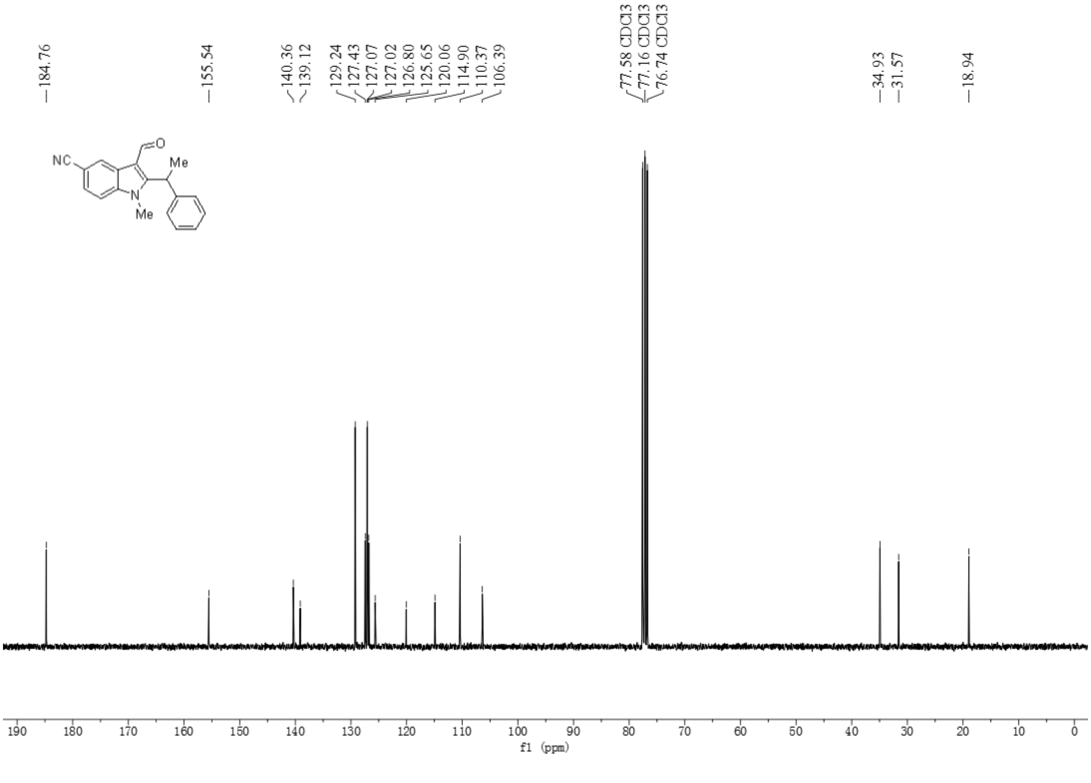

^{1}H NMR spectrum of **52** (300 MHz, $CDCl_3$)

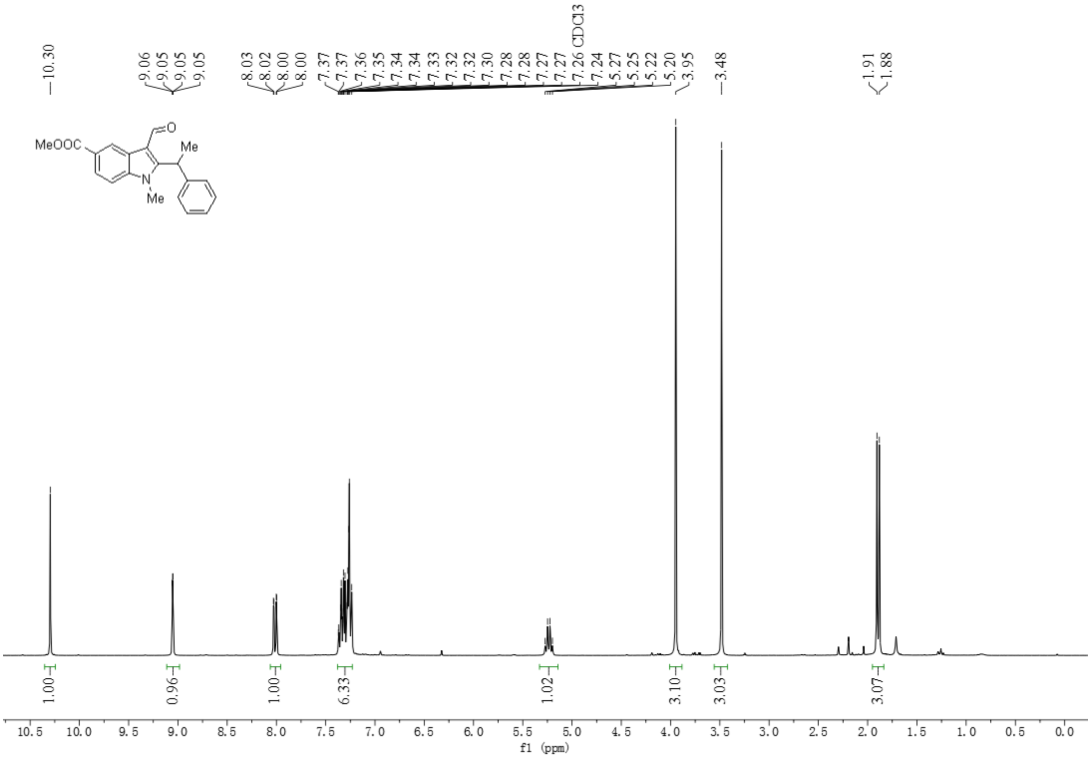

^{13}C NMR spectrum of **52** (75 MHz, $CDCl_3$)

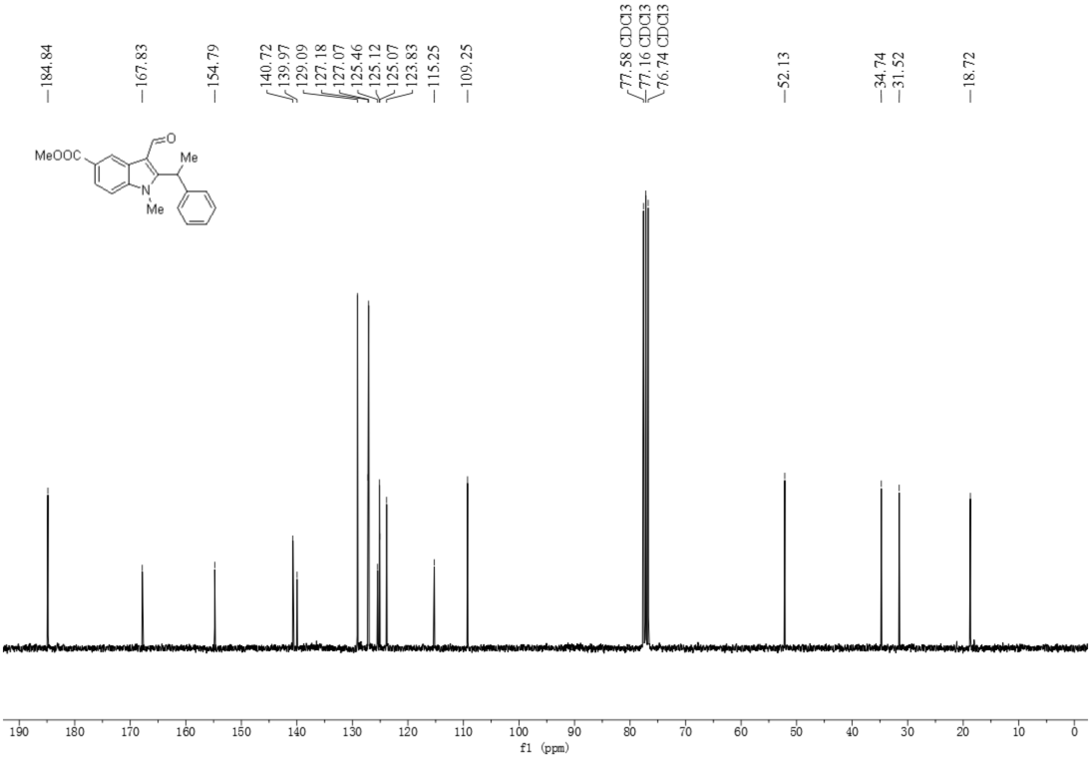

^{1}H NMR spectrum of **53** (300 MHz, $CDCl_3$)

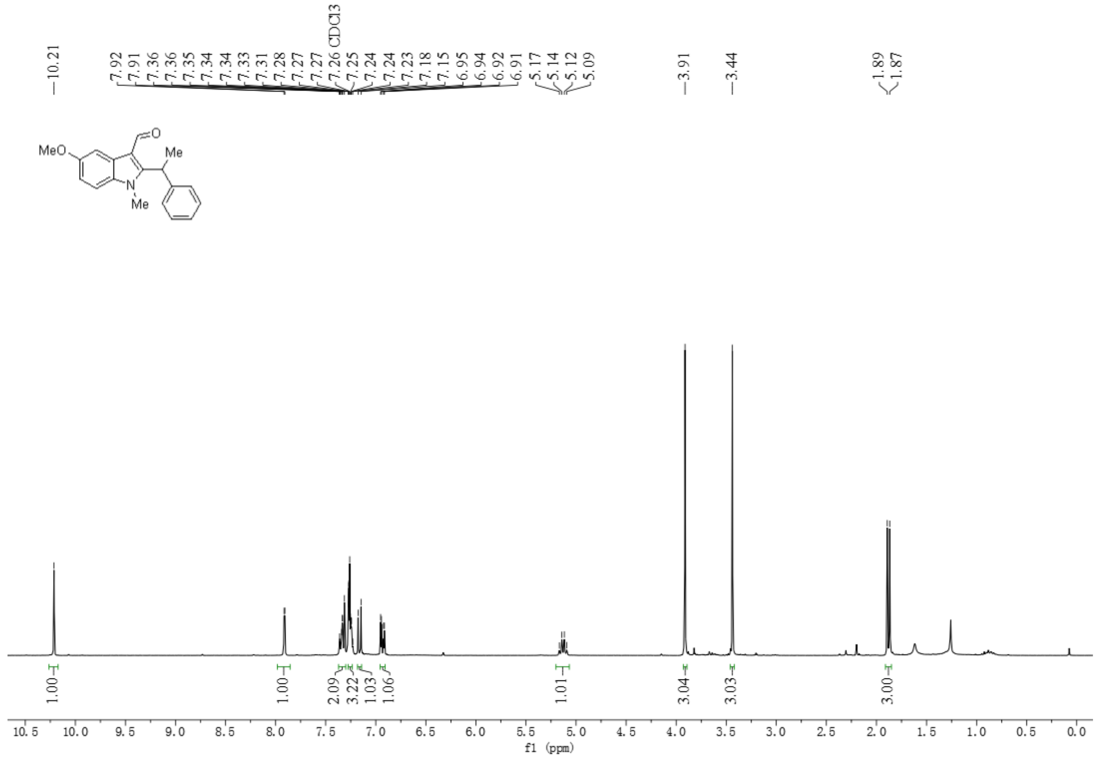

^{13}C NMR spectrum of **53** (75 MHz, $CDCl_3$)

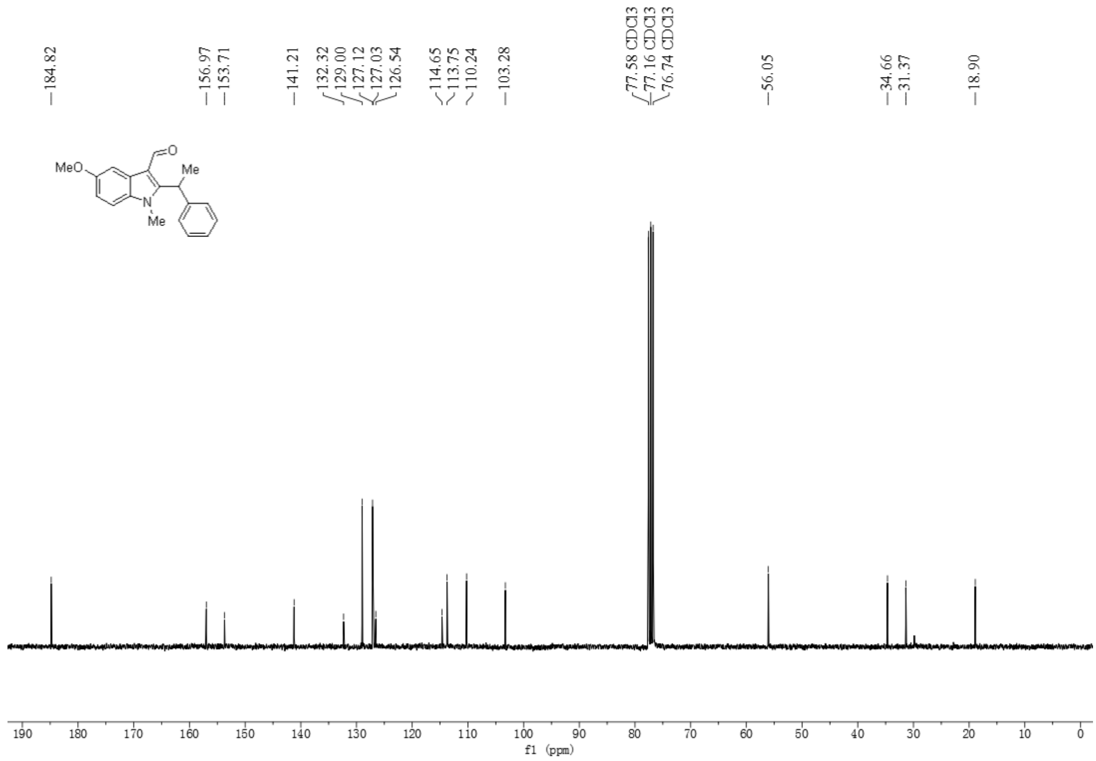

^{1}H NMR spectrum of **54** (300 MHz, $CDCl_3$)

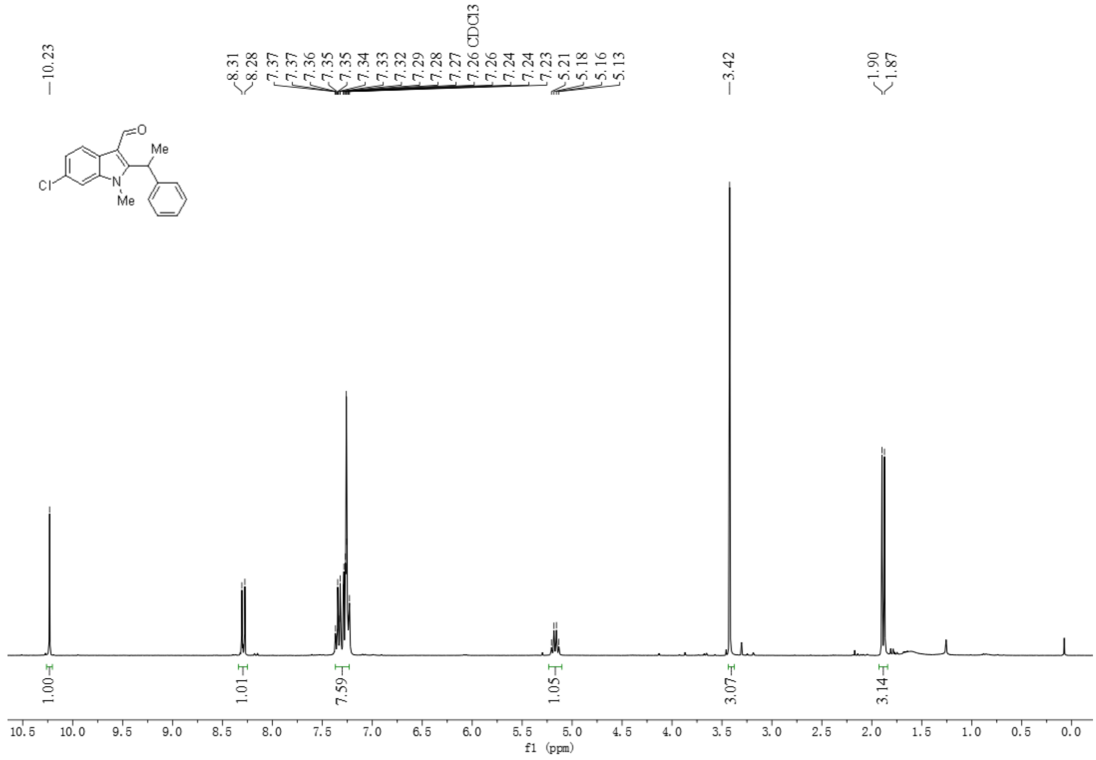

^{13}C NMR spectrum of **54** (75 MHz, $CDCl_3$)

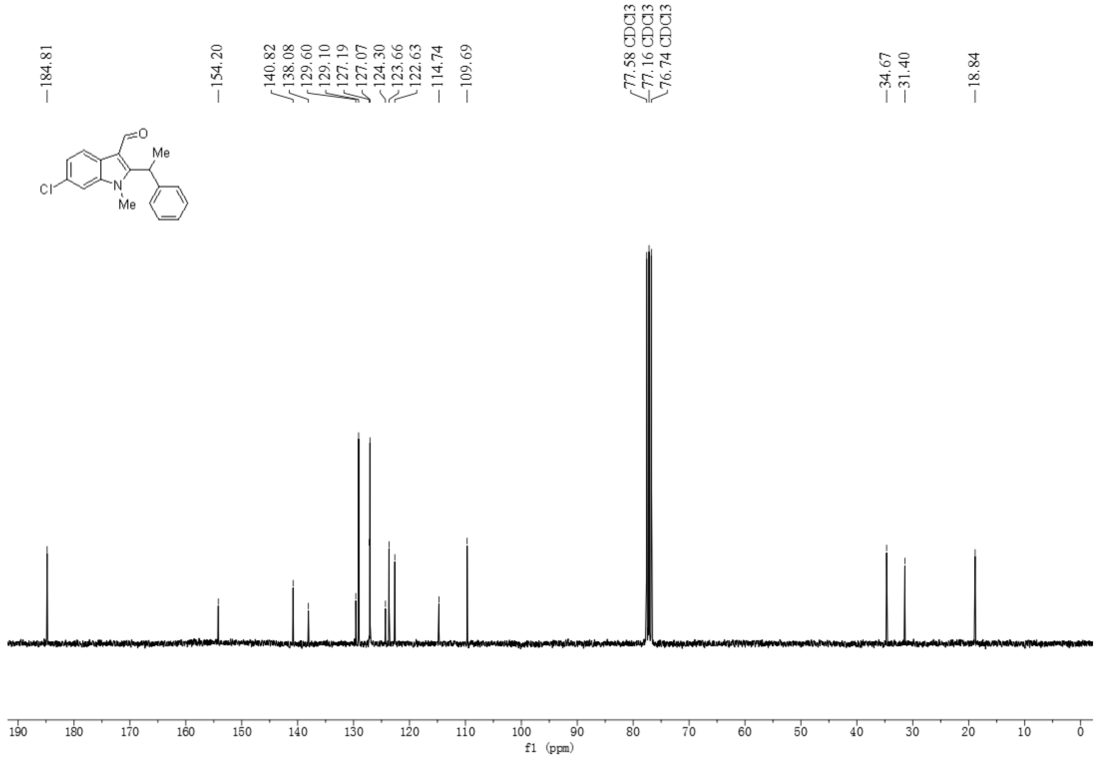

^{1}H NMR spectrum of **55** (300 MHz, $CDCl_3$)

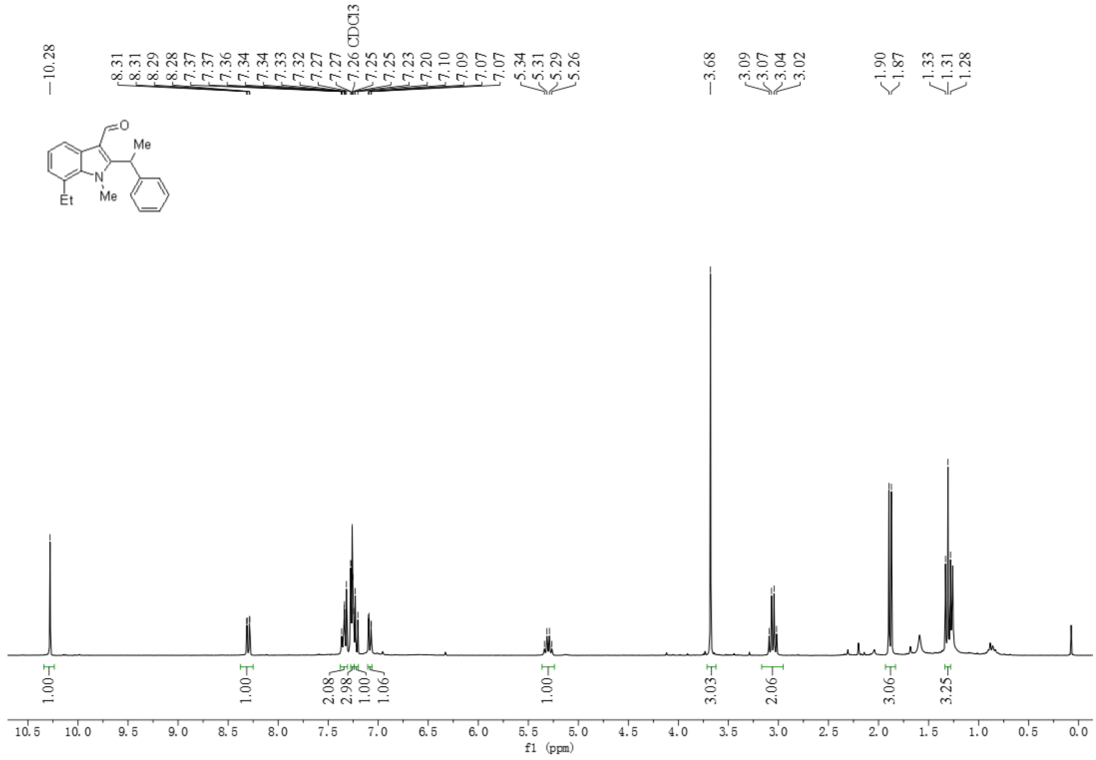

^{13}C NMR spectrum of **55** (75 MHz, $CDCl_3$)

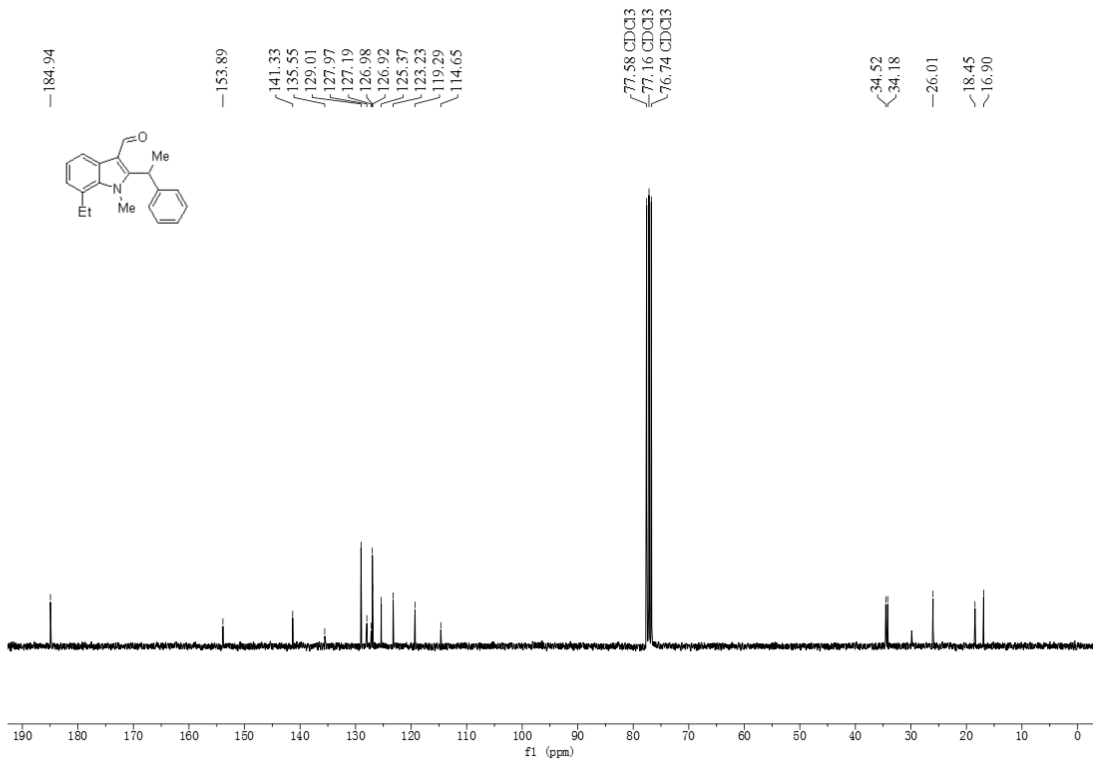

^{1}H NMR spectrum of **56** (300 MHz, $CDCl_3$)

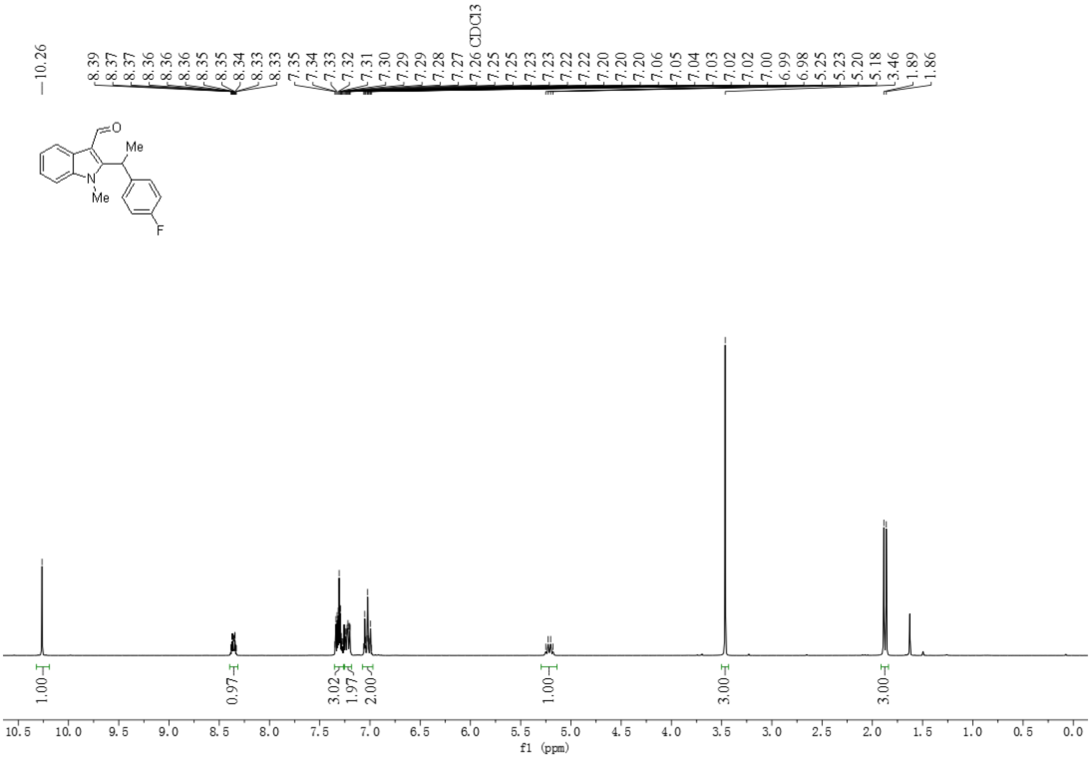

^{13}C NMR spectrum of **56** (75 MHz, $CDCl_3$)

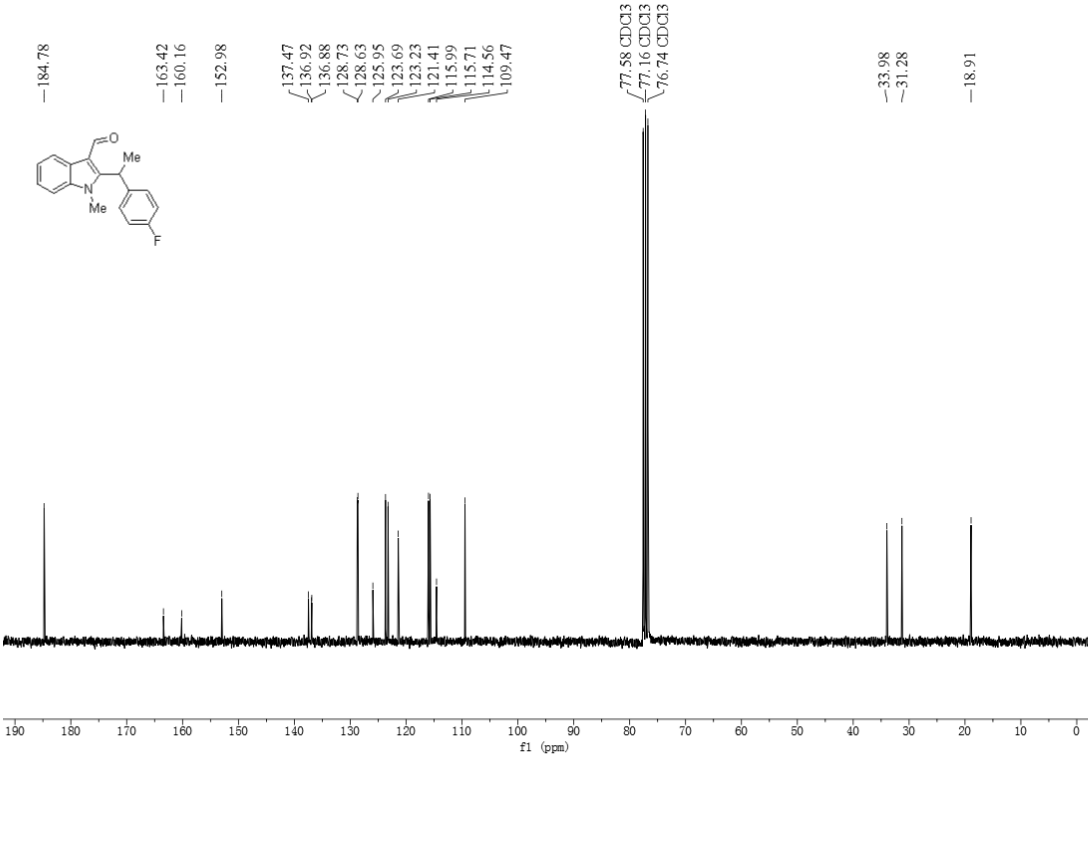

^{19}F NMR spectrum of **56** (282 MHz, $CDCl_3$)

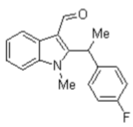

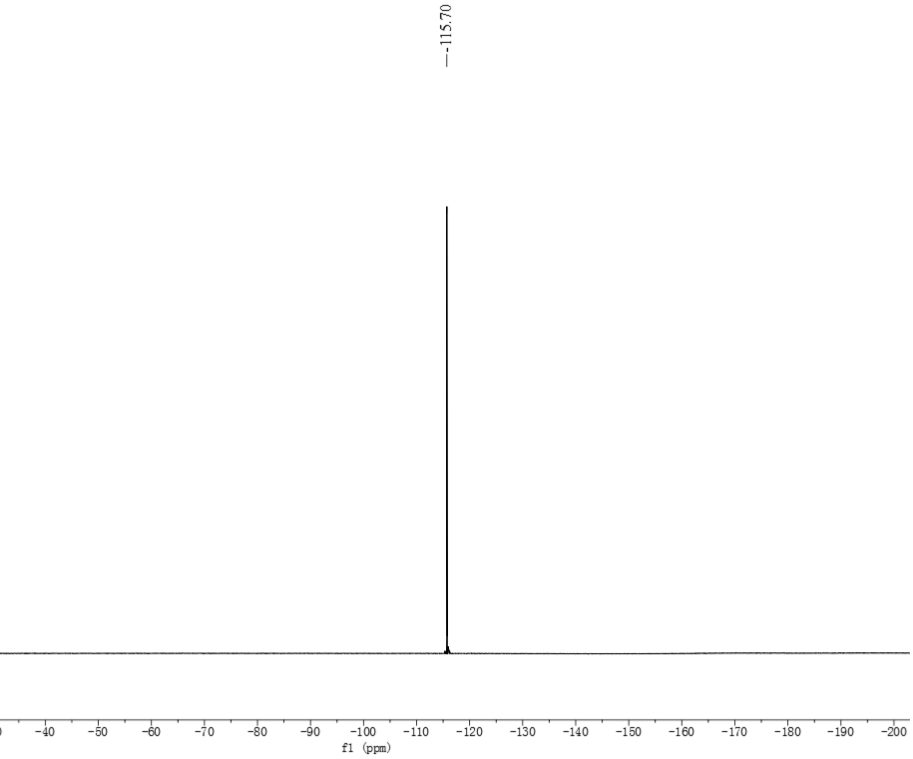

^{1}H NMR spectrum of **57** (300 MHz, $CDCl_3$)

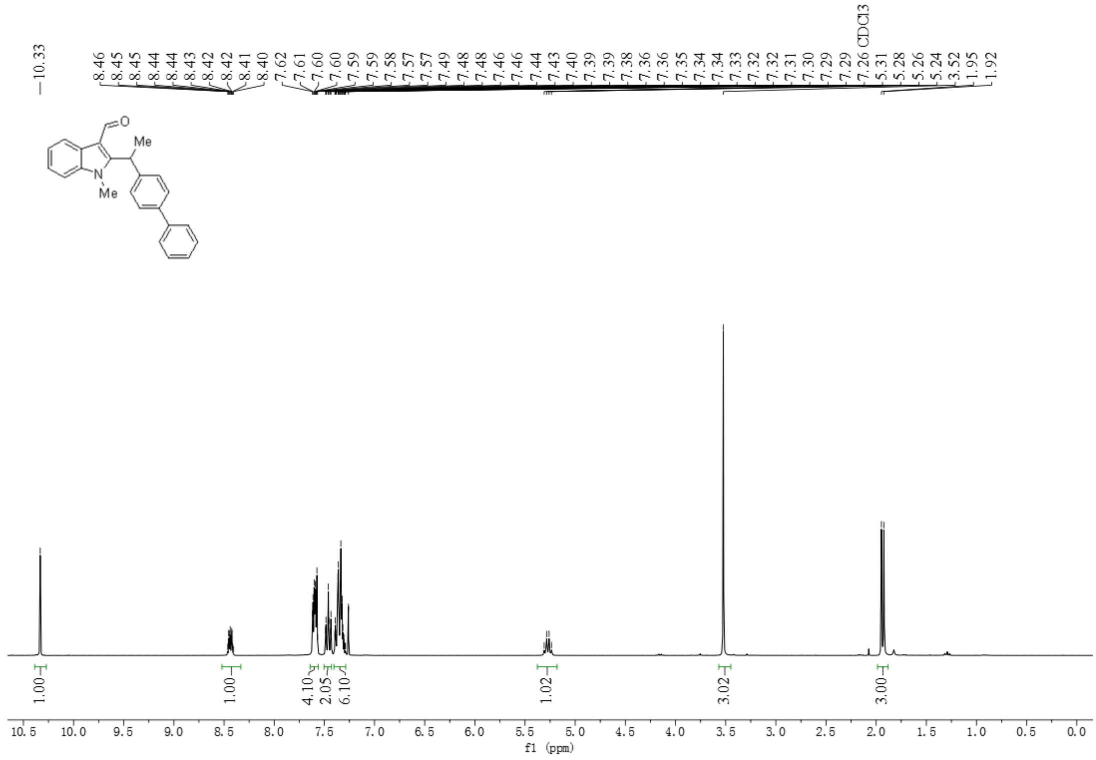

^{13}C NMR spectrum of **57** (75 MHz, $CDCl_3$)

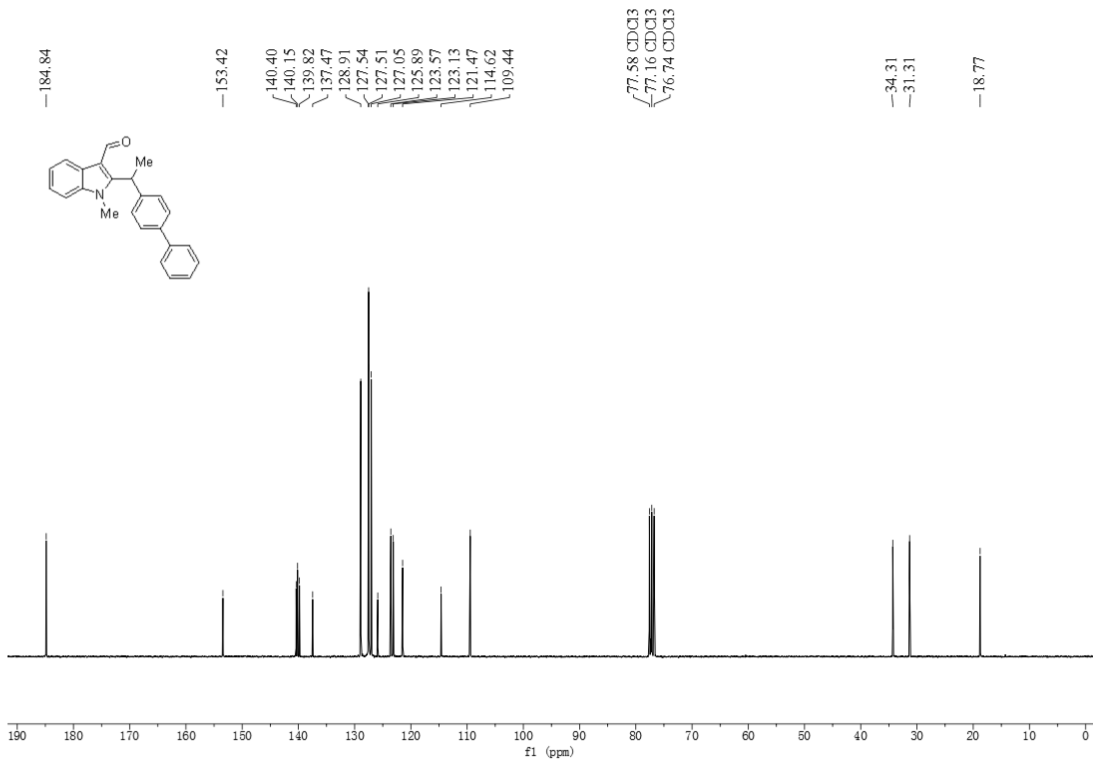

^{1}H NMR spectrum of **58** (300 MHz, $CDCl_3$)

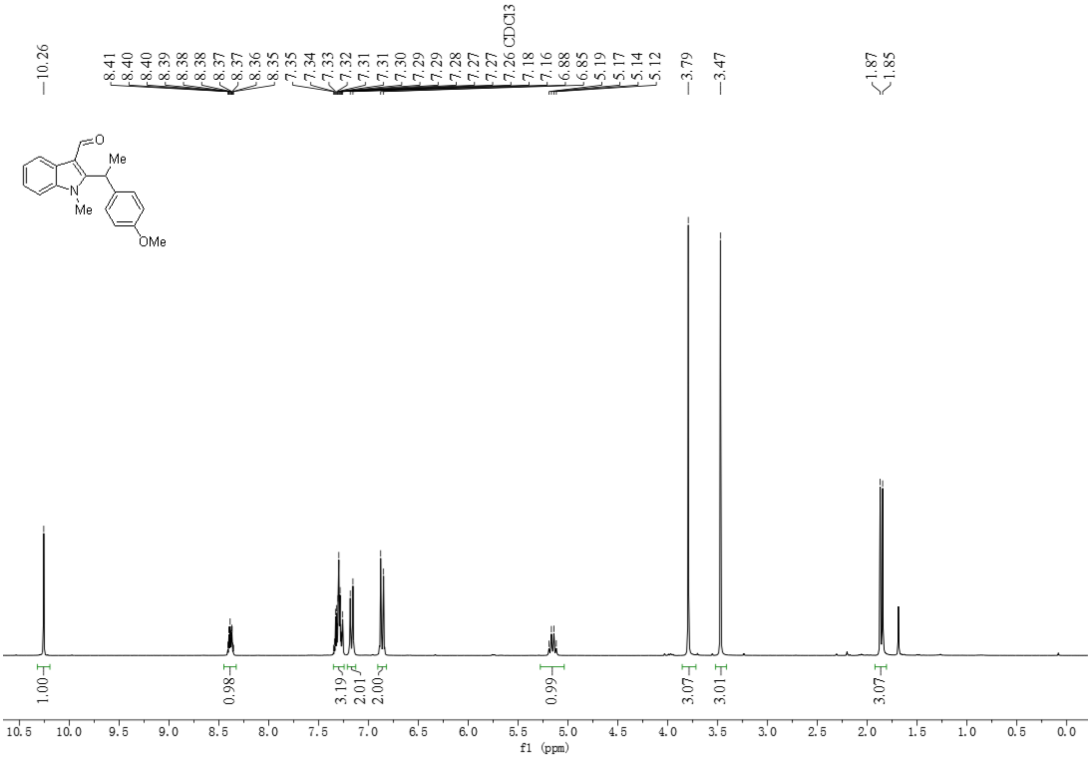

^{13}C NMR spectrum of **58** (75 MHz, $CDCl_3$)

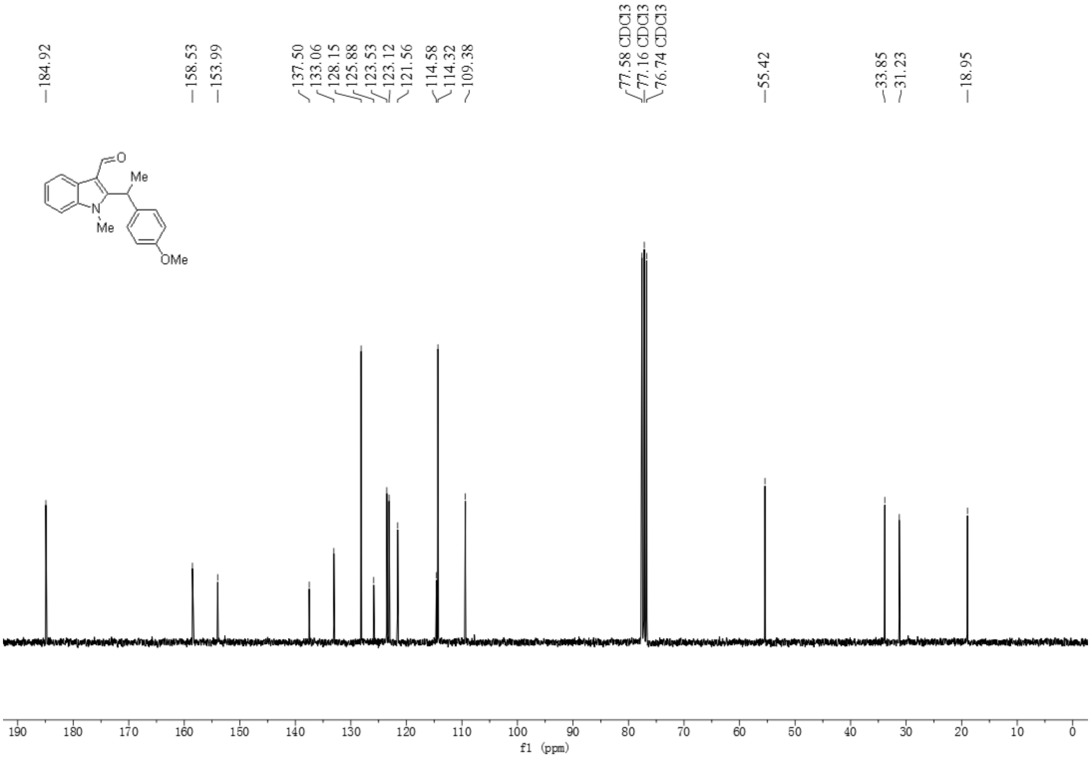

^{1}H NMR spectrum of **59** (300 MHz, $CDCl_3$)

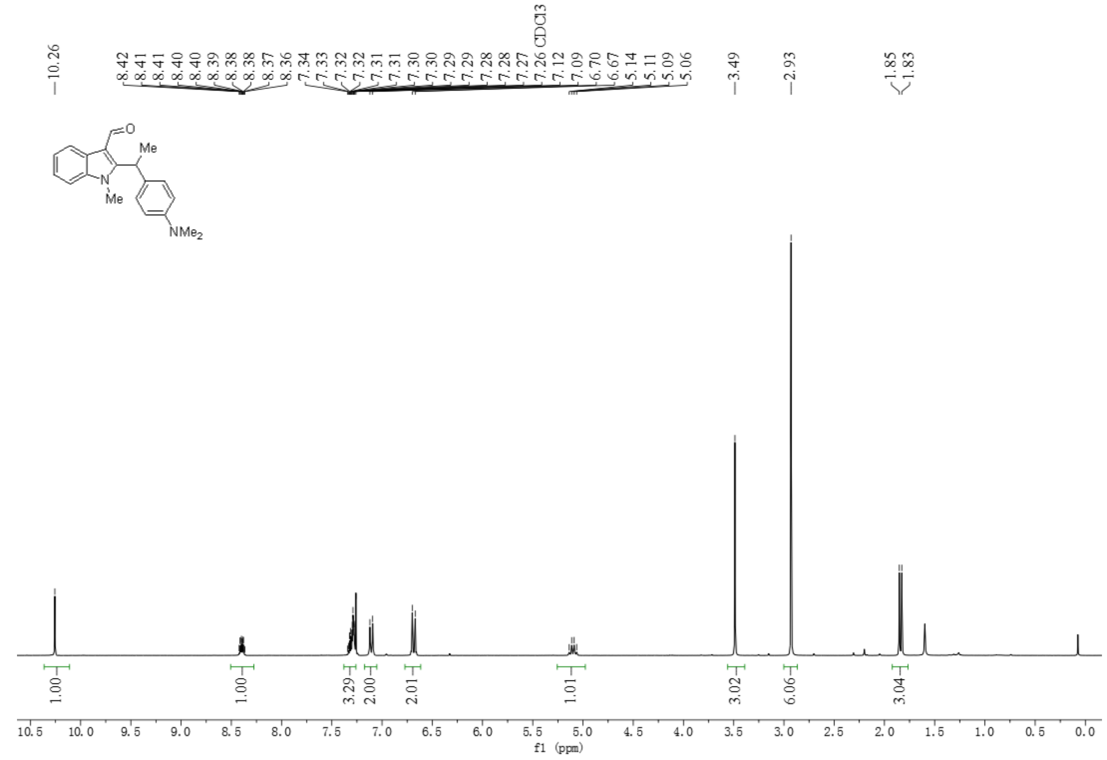

^{13}C NMR spectrum of **59** (75 MHz, $CDCl_3$)

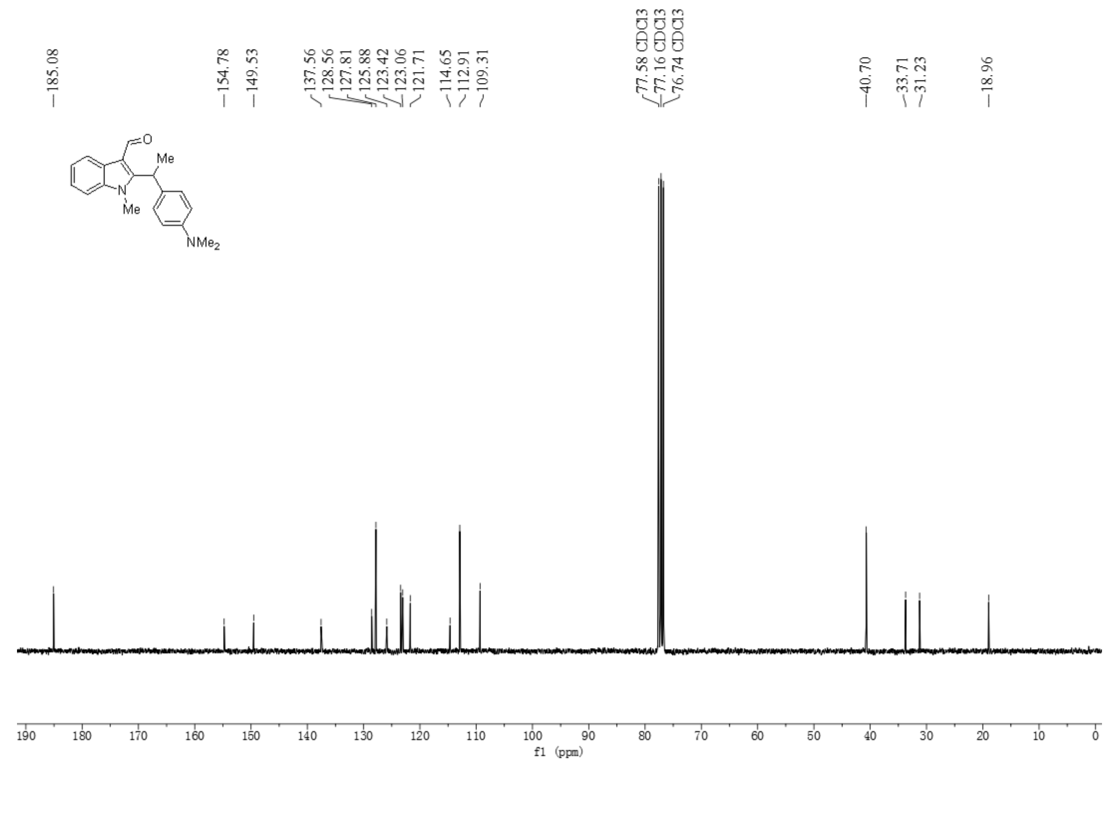

^{1}H NMR spectrum of **60** (300 MHz, $CDCl_3$)

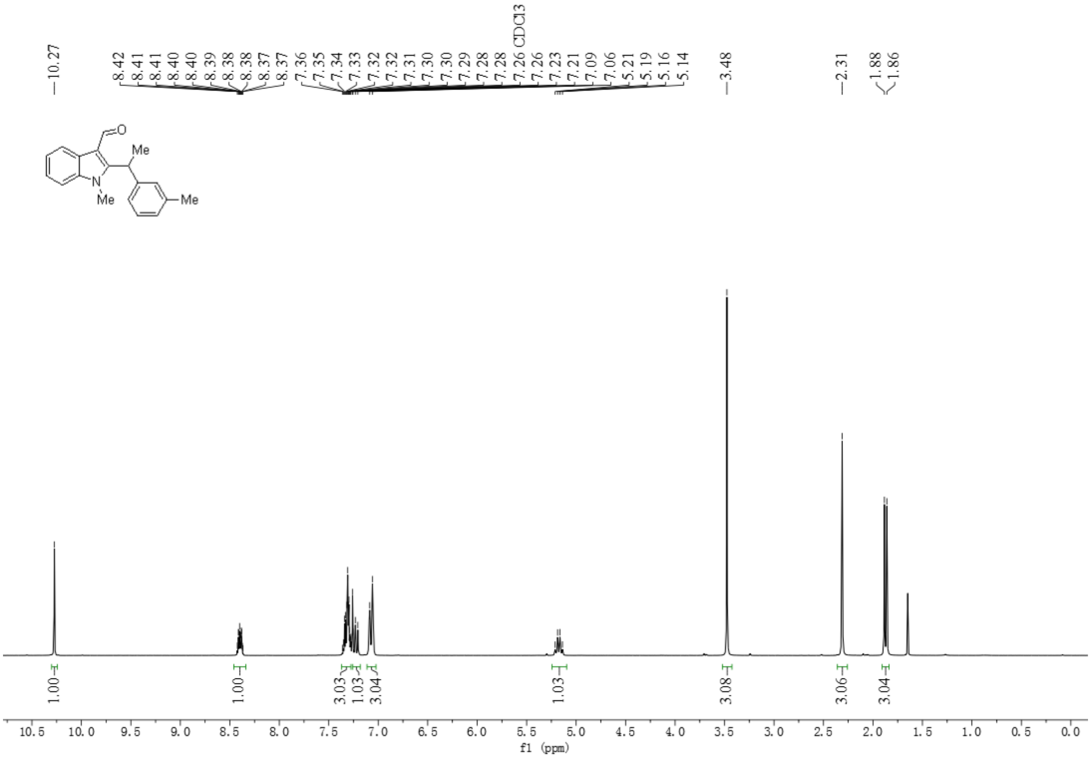

^{13}C NMR spectrum of **60** (75 MHz, $CDCl_3$)

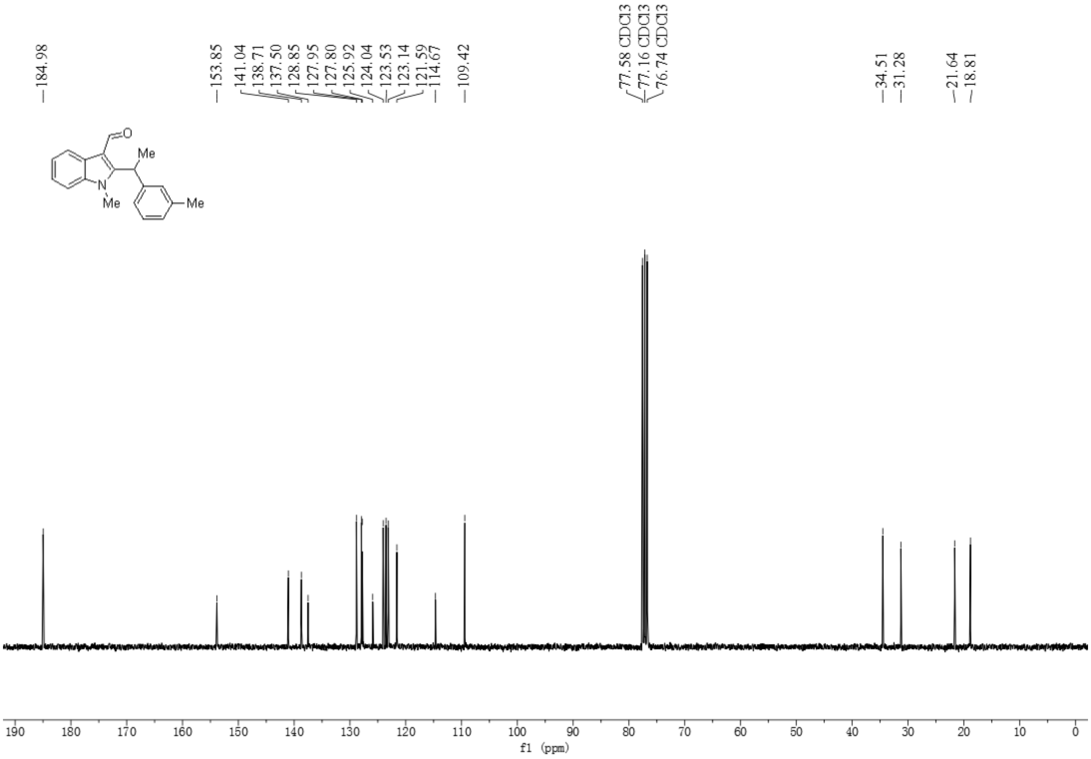

^{1}H NMR spectrum of **61** (300 MHz, $CDCl_3$)

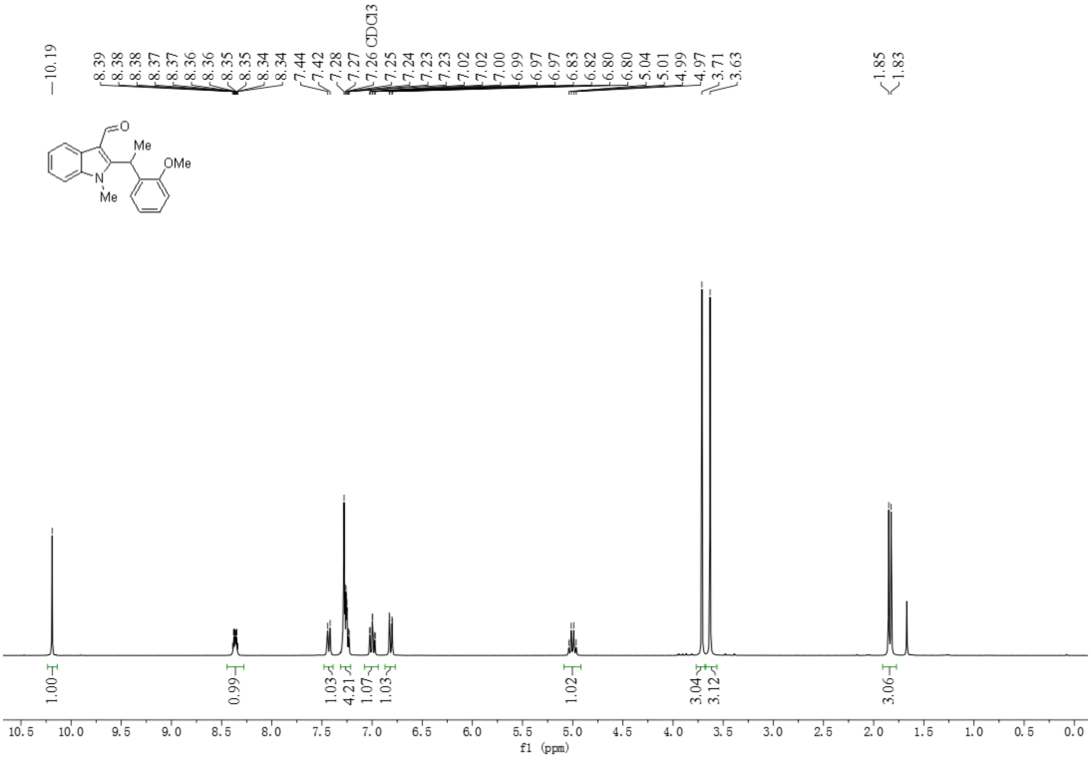

^{13}C NMR spectrum of **61** (75 MHz, $CDCl_3$)

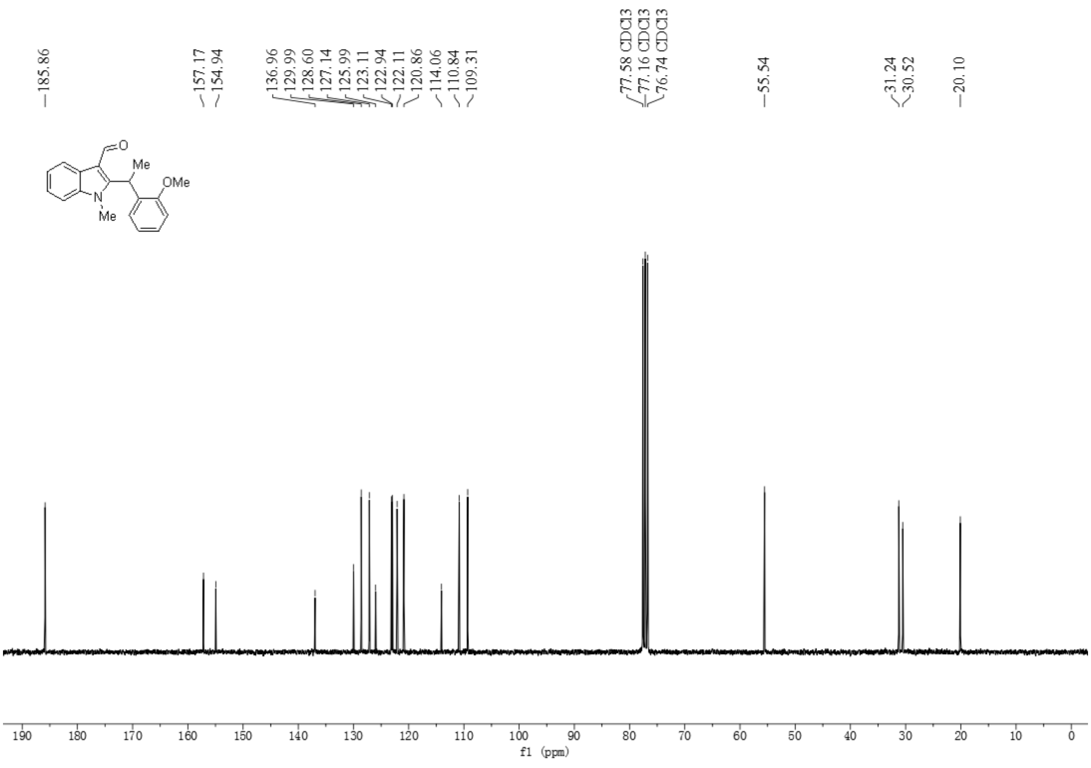

^{1}H NMR spectrum of **62** (300 MHz, $CDCl_3$)

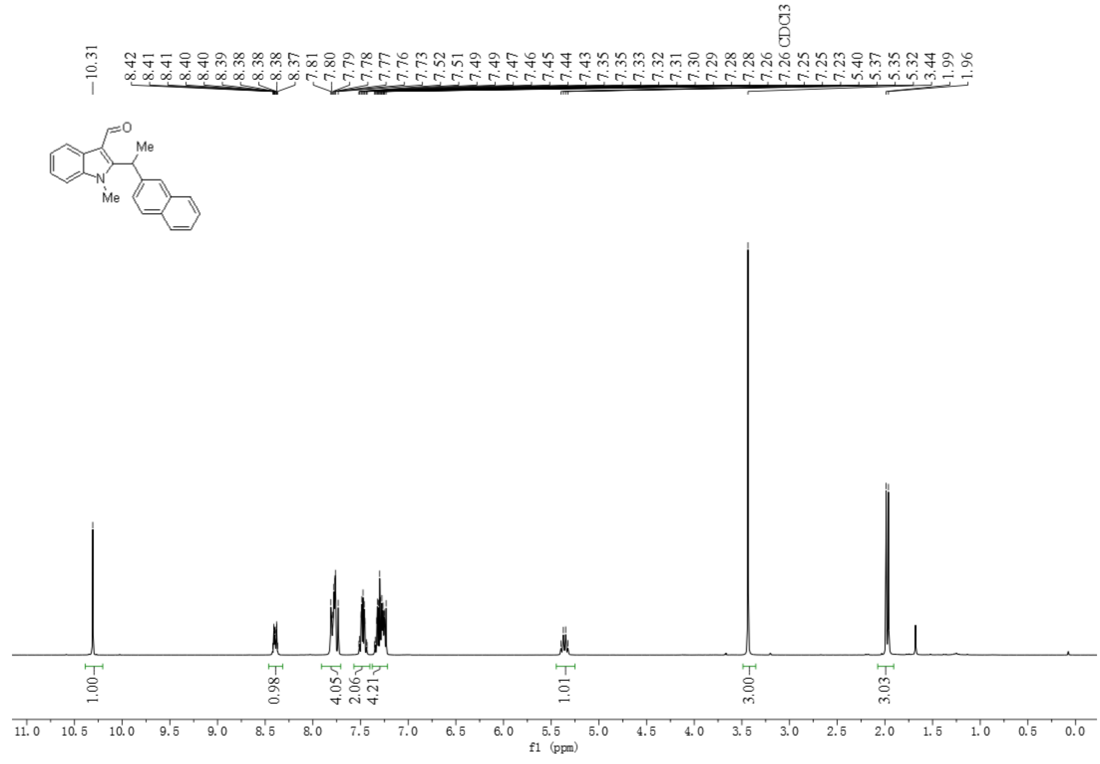

^{13}C NMR spectrum of **62** (75 MHz, $CDCl_3$)

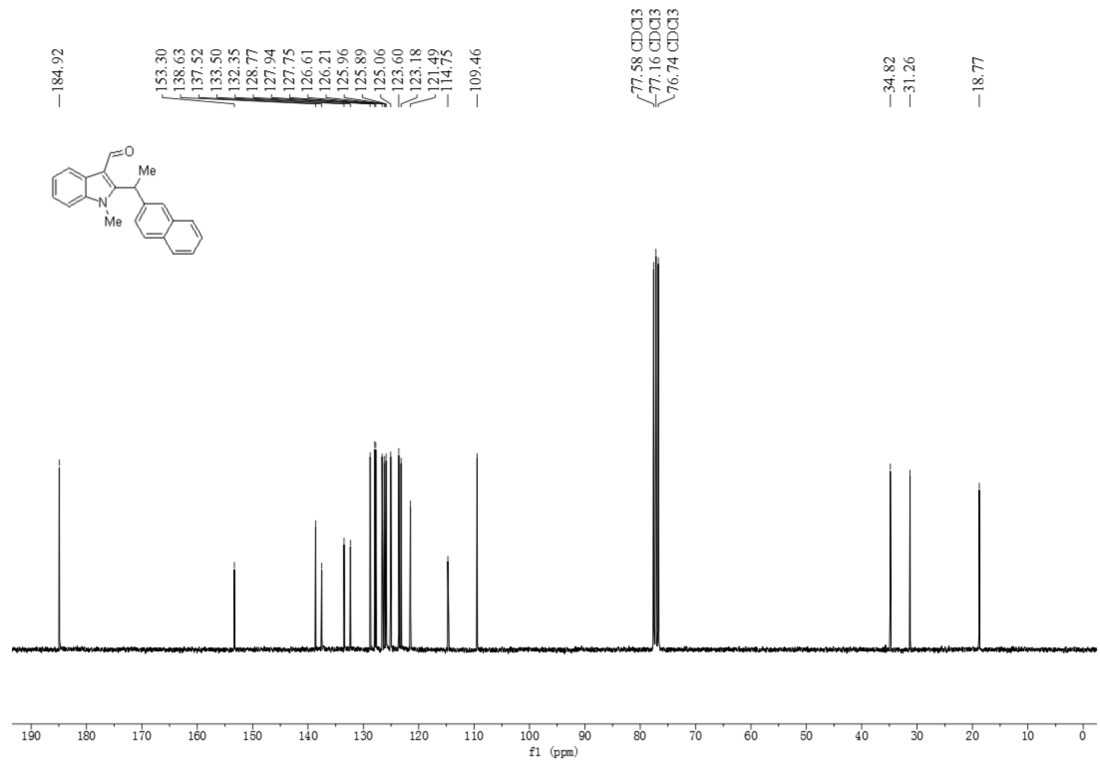

^{1}H NMR spectrum of **63** (300 MHz, $CDCl_3$)

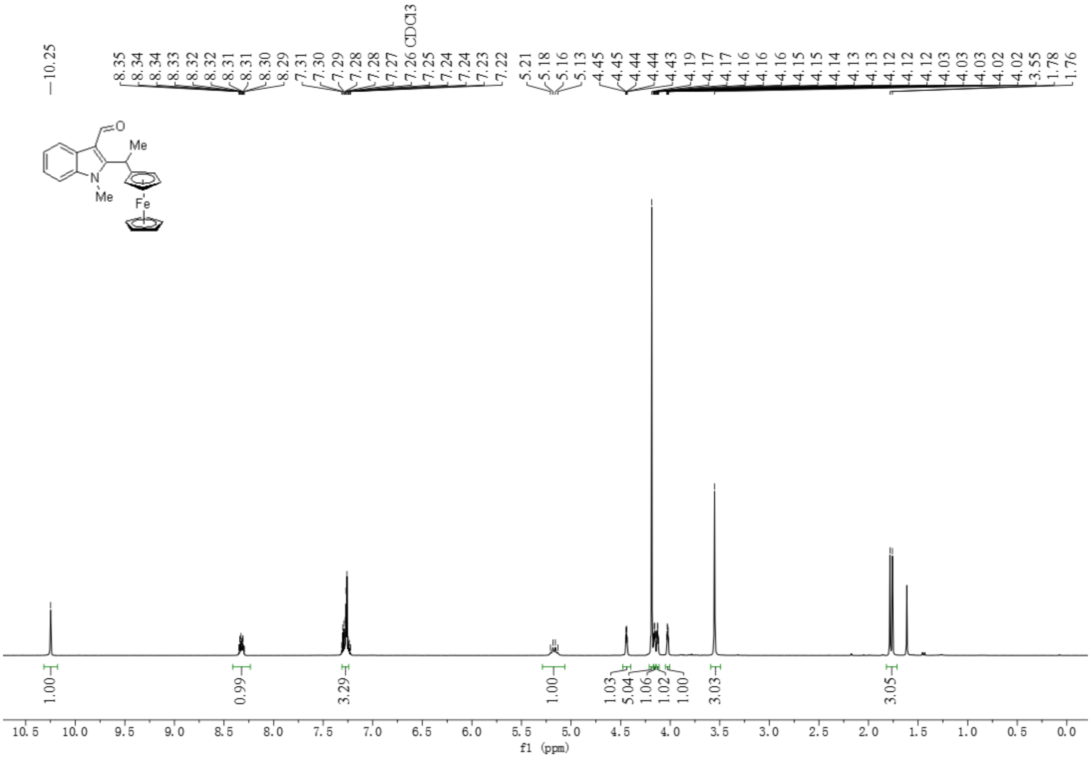

^{13}C NMR spectrum of **63** (75 MHz, $CDCl_3$)

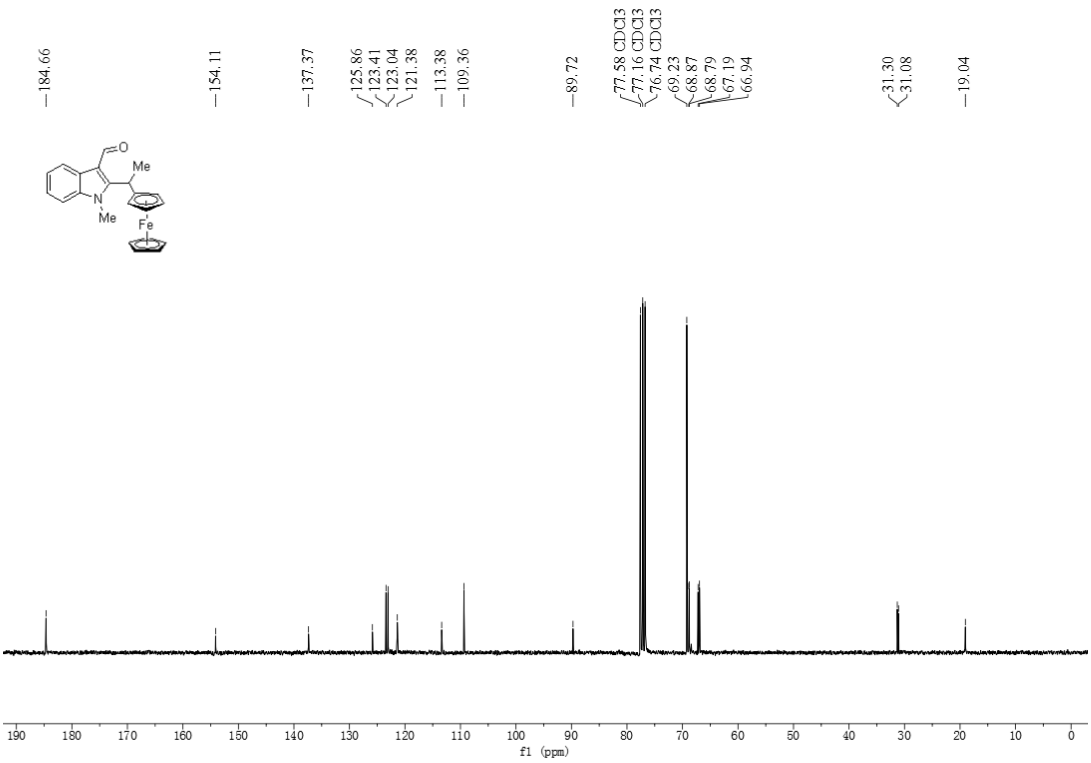

^{1}H NMR spectrum of **64** (300 MHz, $CDCl_3$)

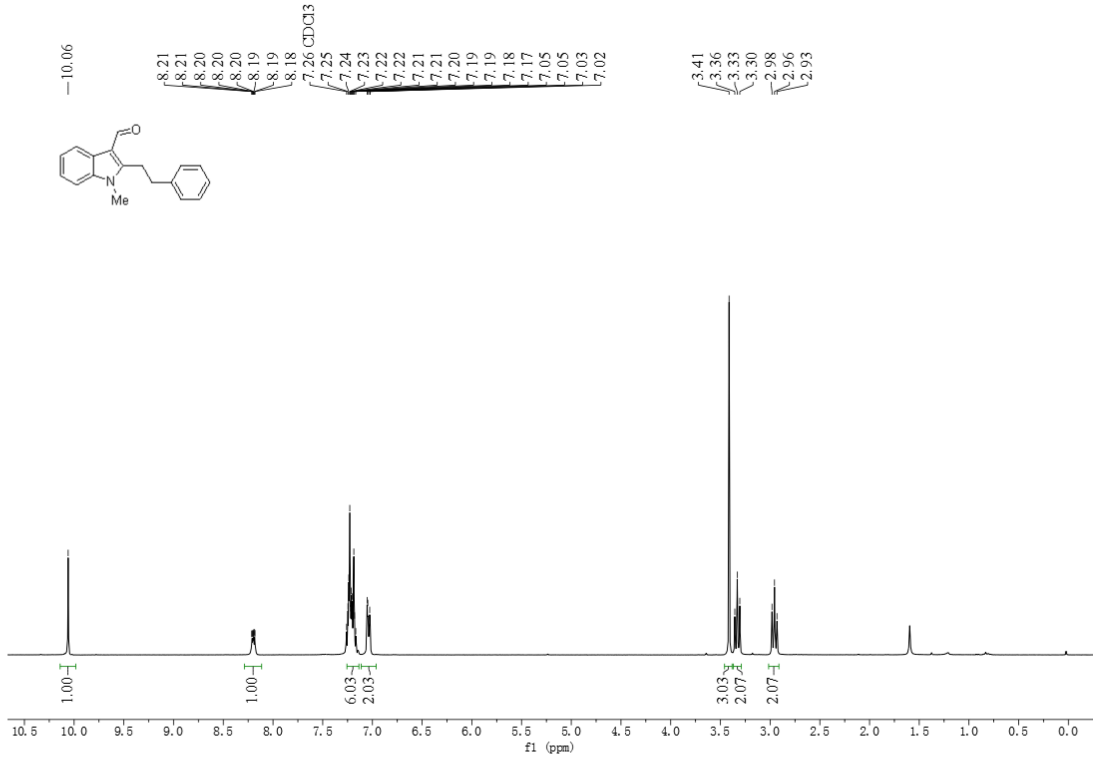

^{13}C NMR spectrum of **64** (75 MHz, $CDCl_3$)

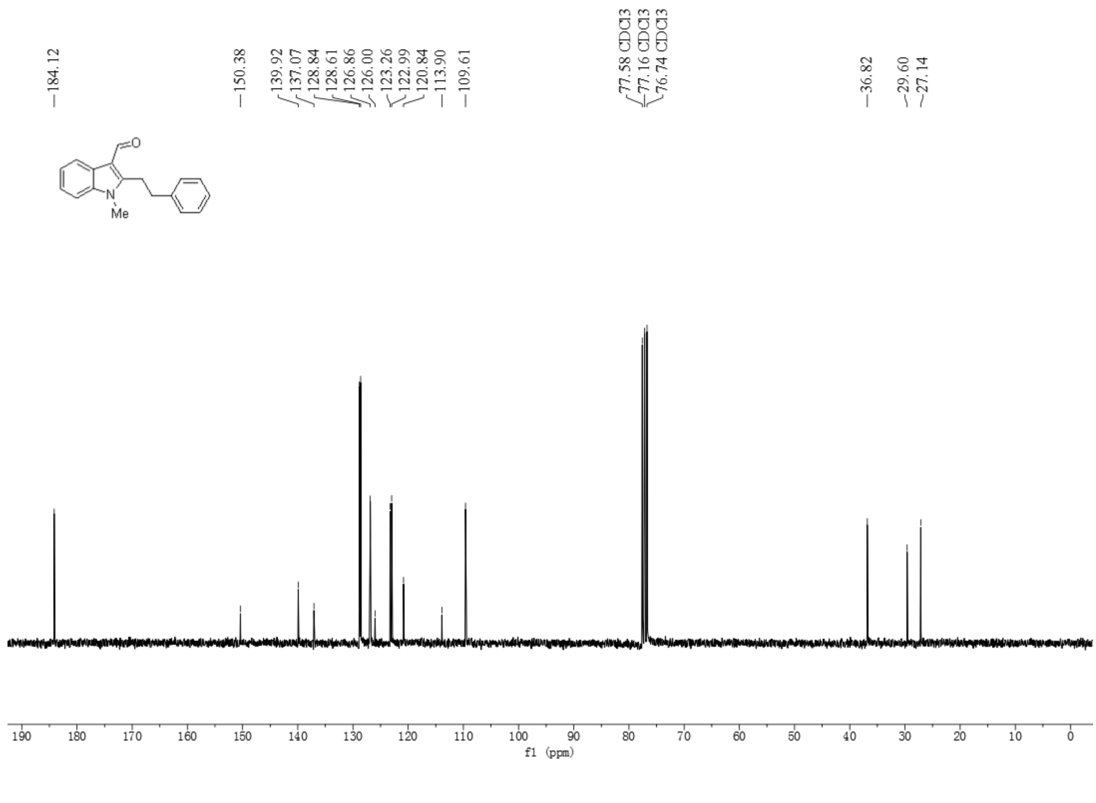

^{1}H NMR spectrum of **65** (300 MHz, $CDCl_3$)

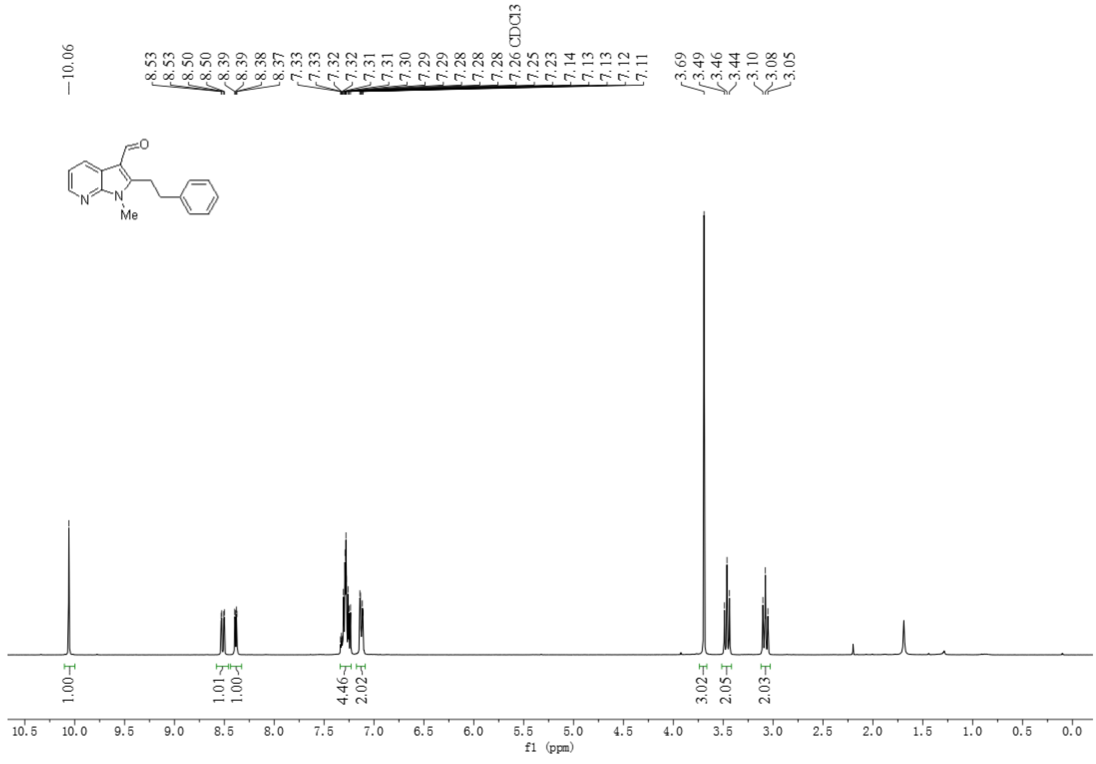

^{13}C NMR spectrum of **65** (75 MHz, $CDCl_3$)

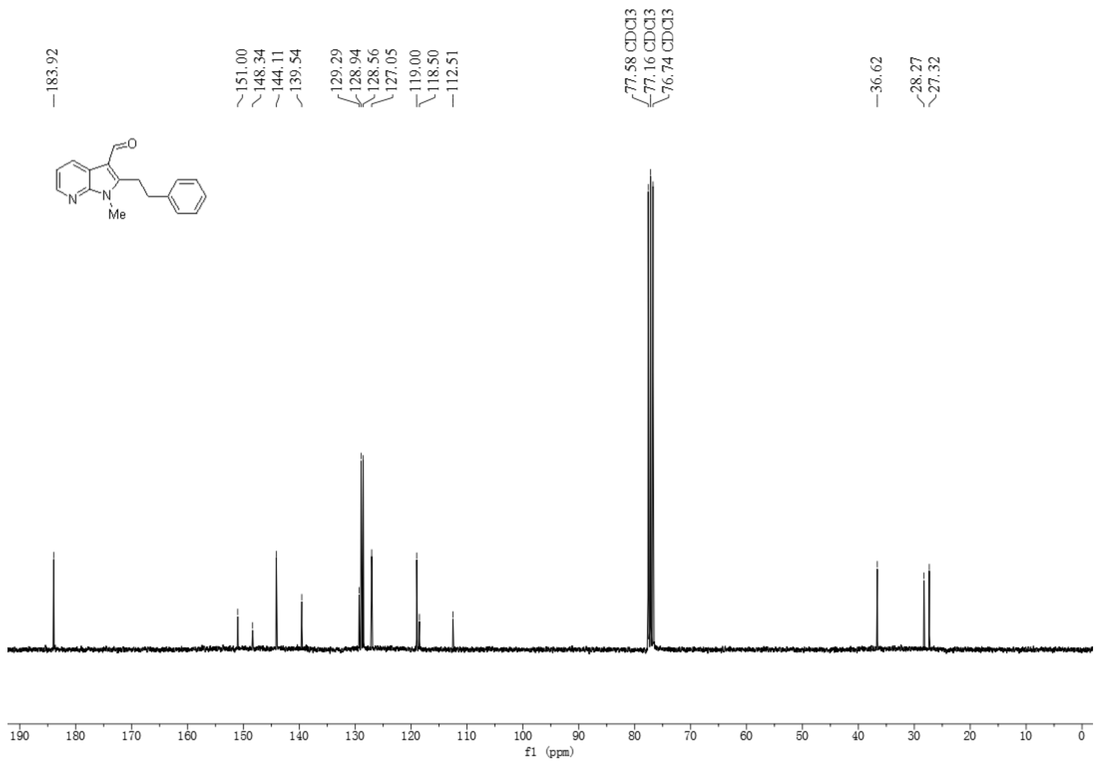

^{1}H NMR spectrum of **66** (300 MHz, $CDCl_3$)

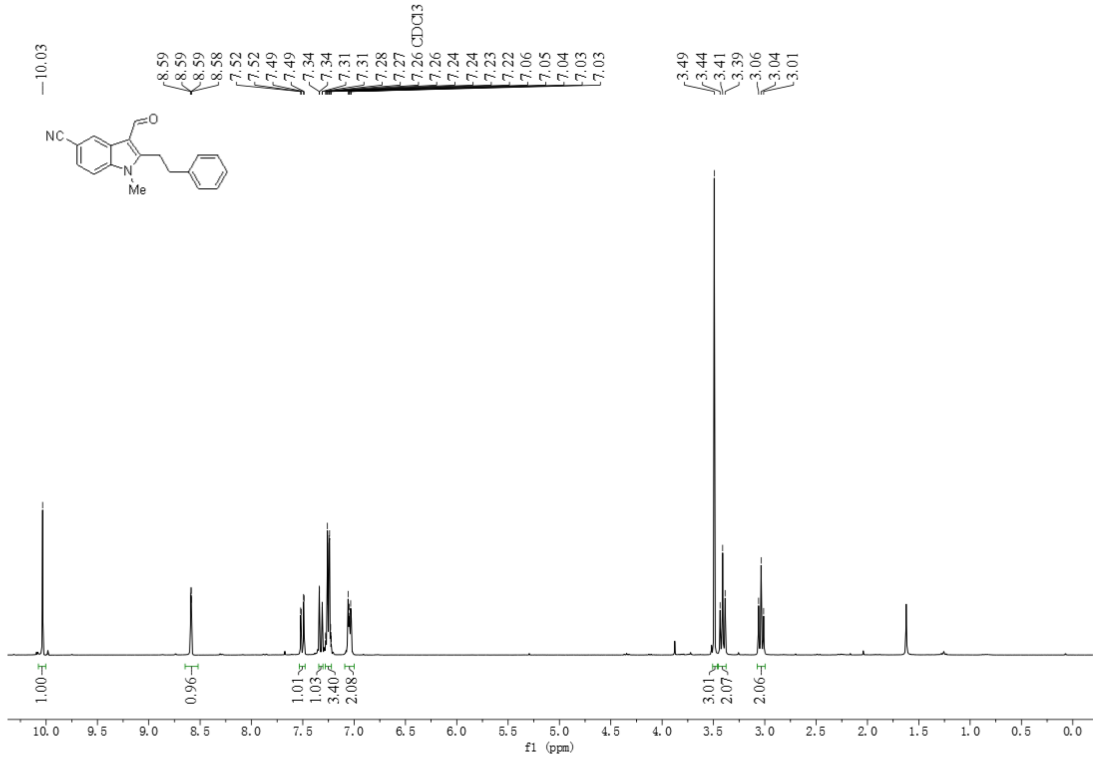

^{13}C NMR spectrum of **66** (75 MHz, $CDCl_3$)

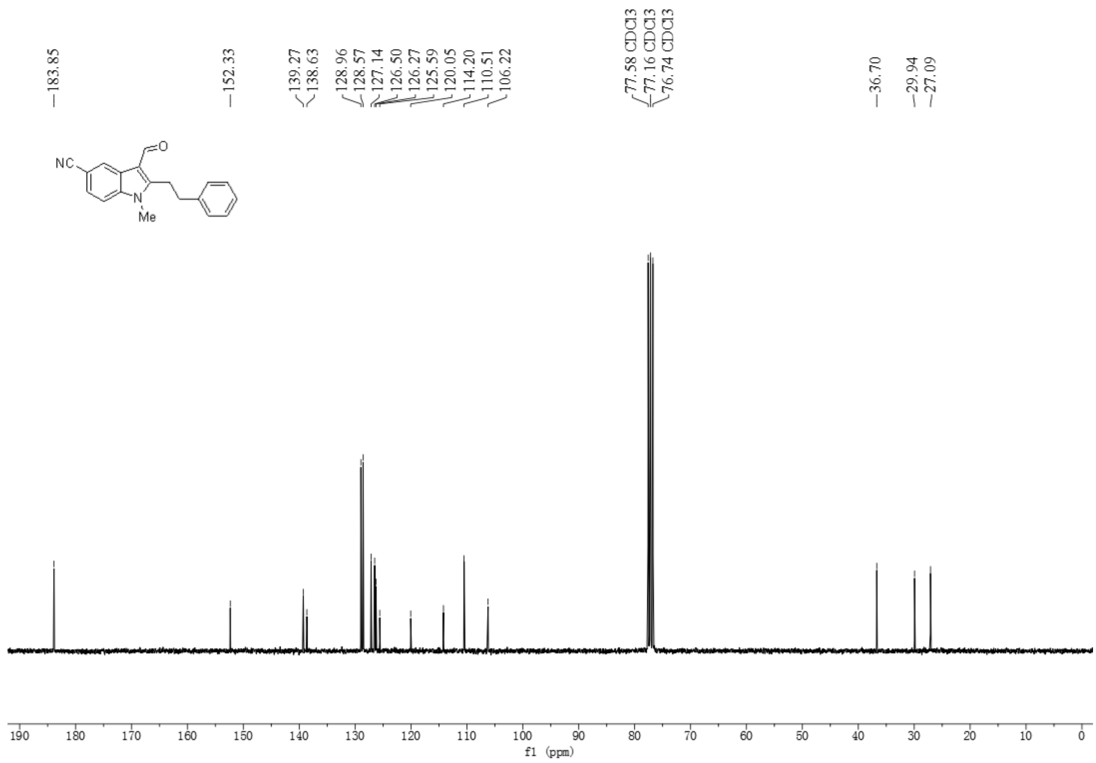

^{1}H NMR spectrum of **67** (300 MHz, $CDCl_3$)

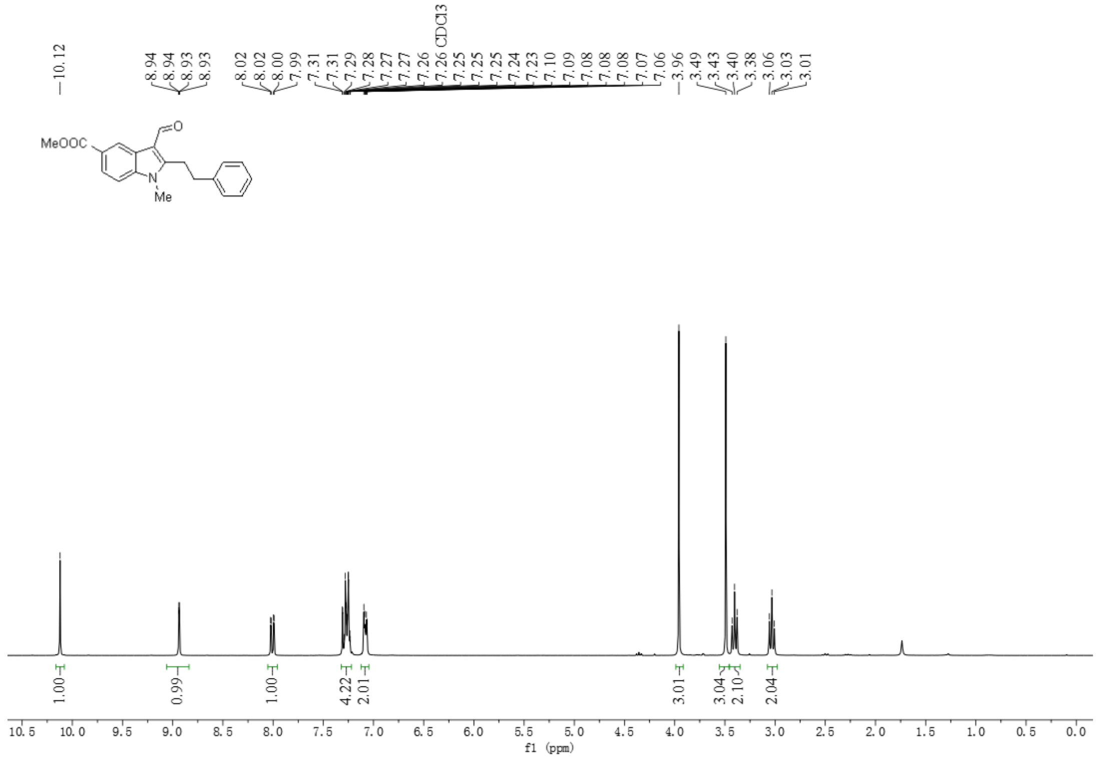

^{13}C NMR spectrum of **67** (75 MHz, $CDCl_3$)

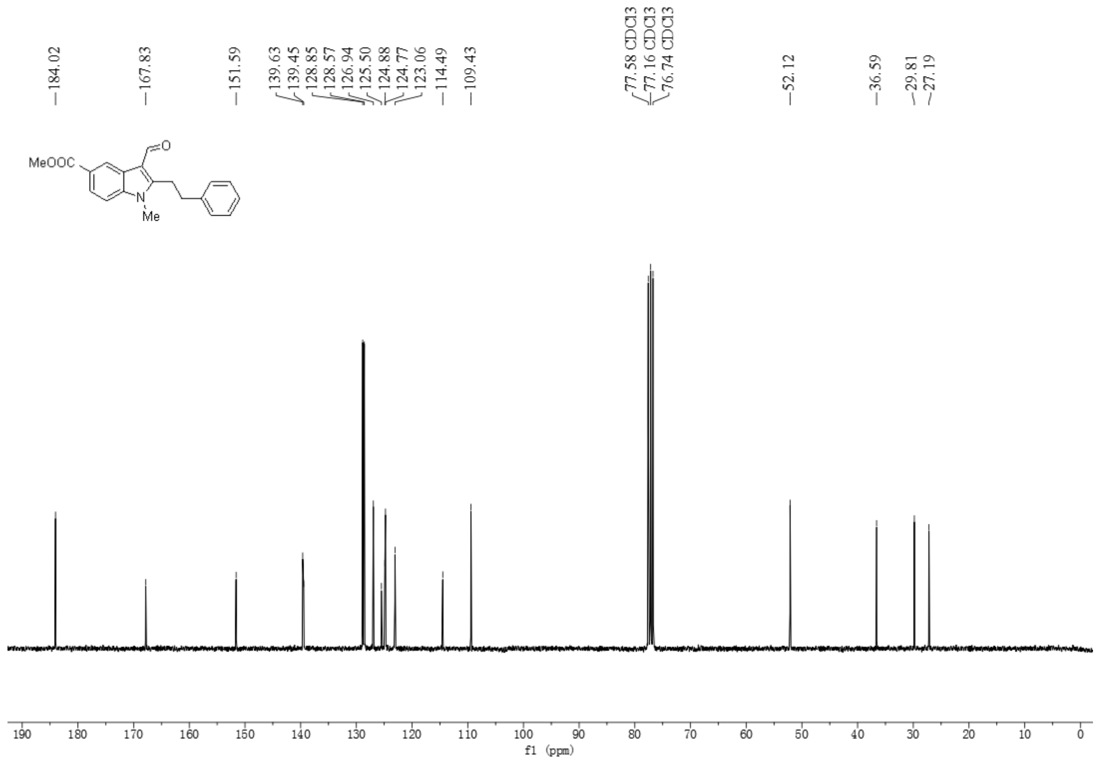

^{1}H NMR spectrum of **68** (300 MHz, $CDCl_3$)

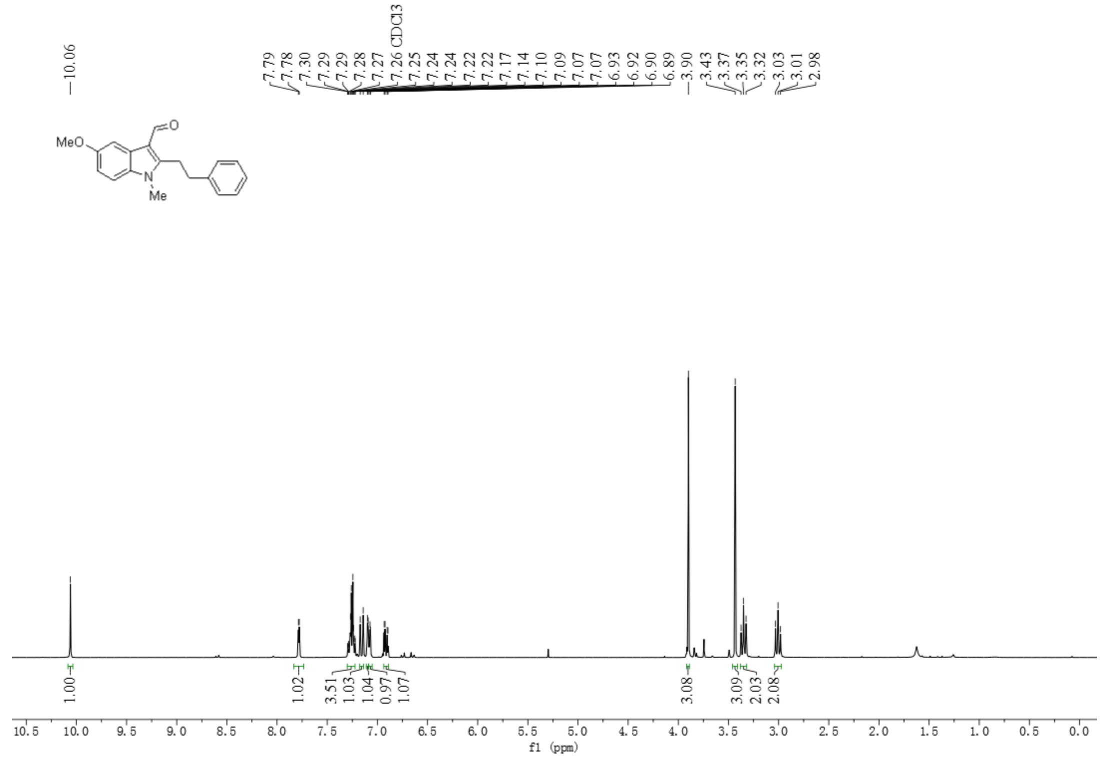

^{13}C NMR spectrum of **68** (75 MHz, $CDCl_3$)

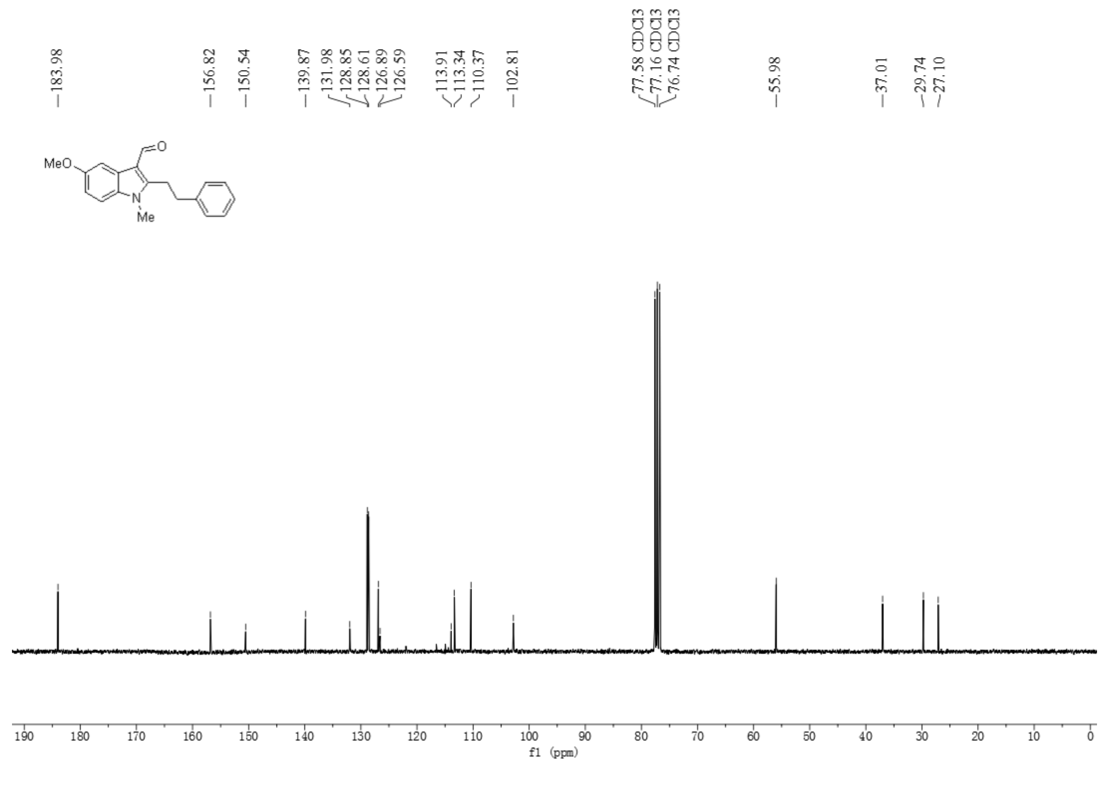

^{1}H NMR spectrum of **69** (300 MHz, $CDCl_3$)

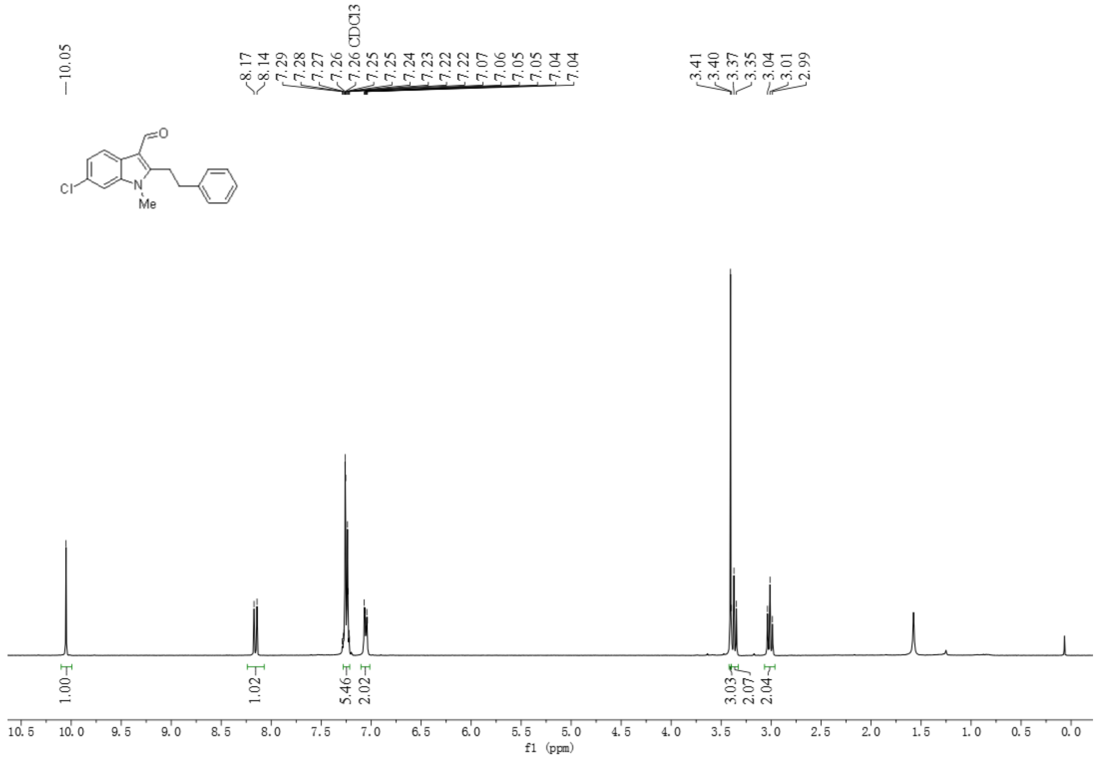

^{13}C NMR spectrum of **69** (75 MHz, $CDCl_3$)

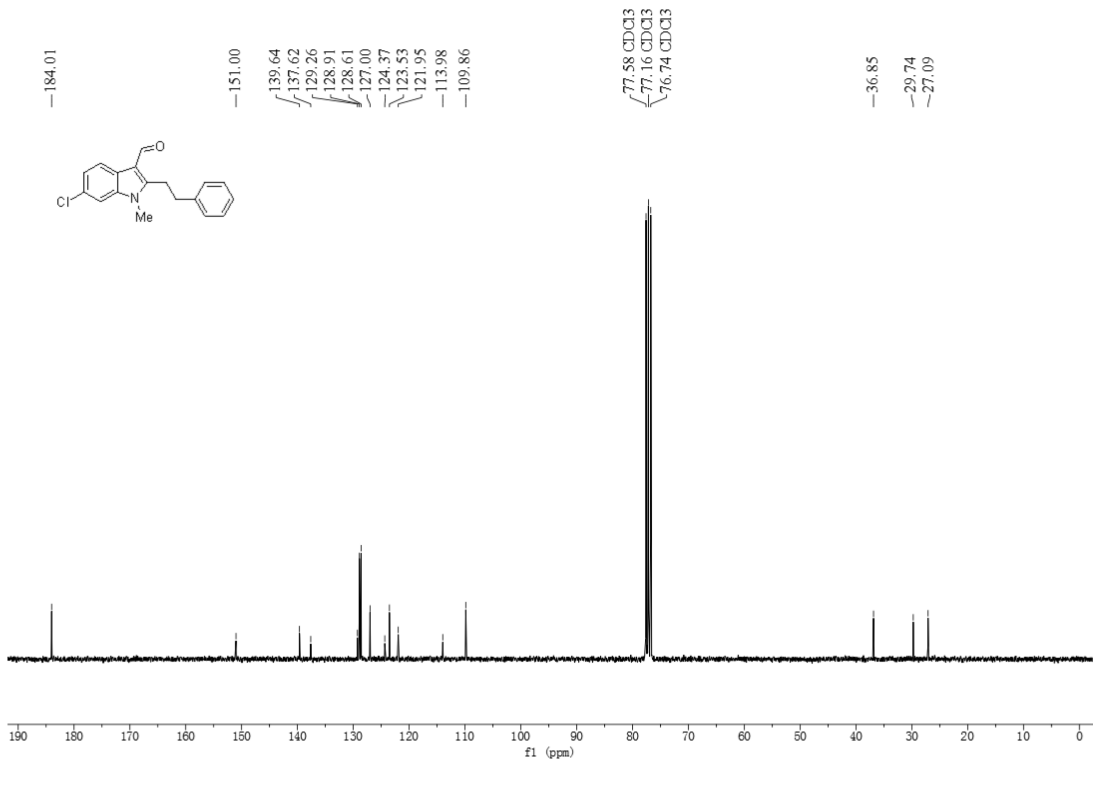

^{1}H NMR spectrum of **70** (300 MHz, $CDCl_3$)

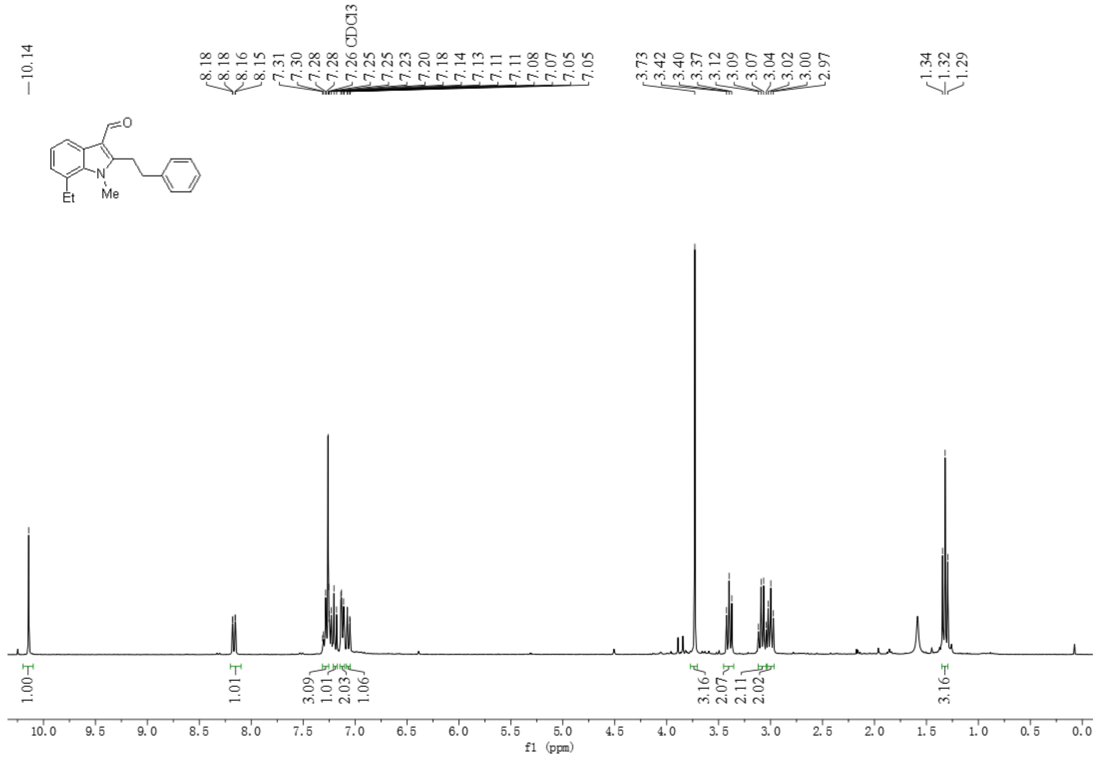

^{13}C NMR spectrum of **70** (75 MHz, $CDCl_3$)

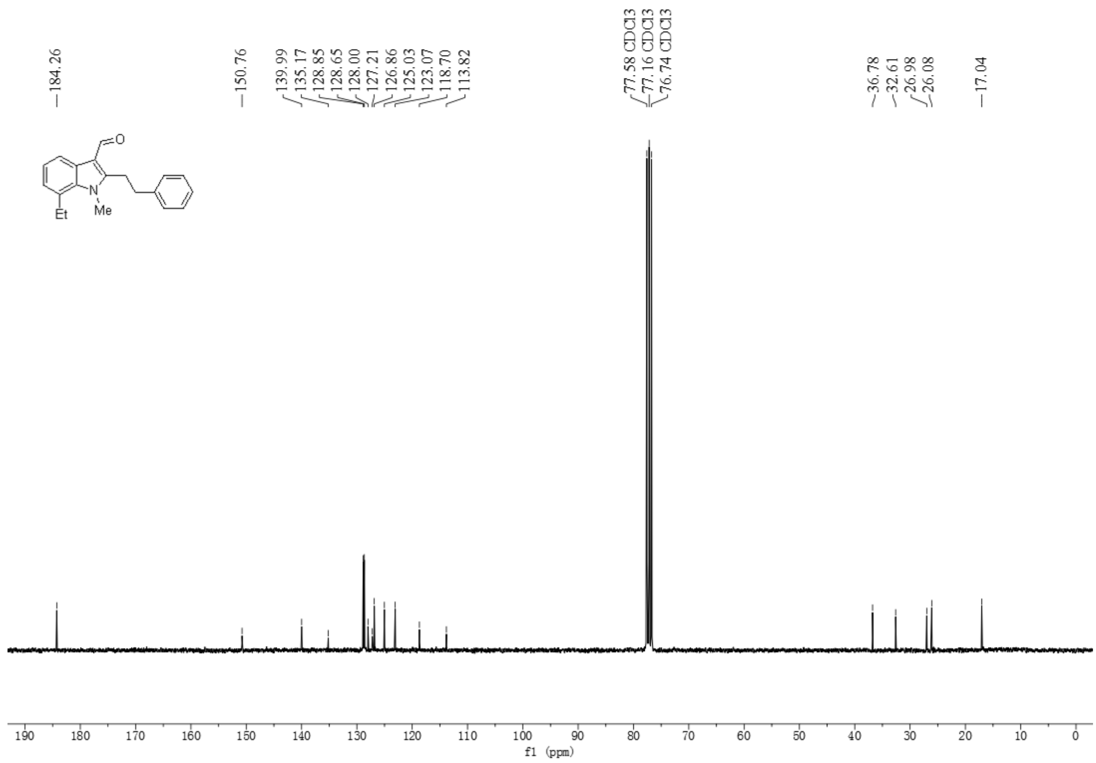

^{1}H NMR spectrum of **71** (300 MHz, $CDCl_3$)

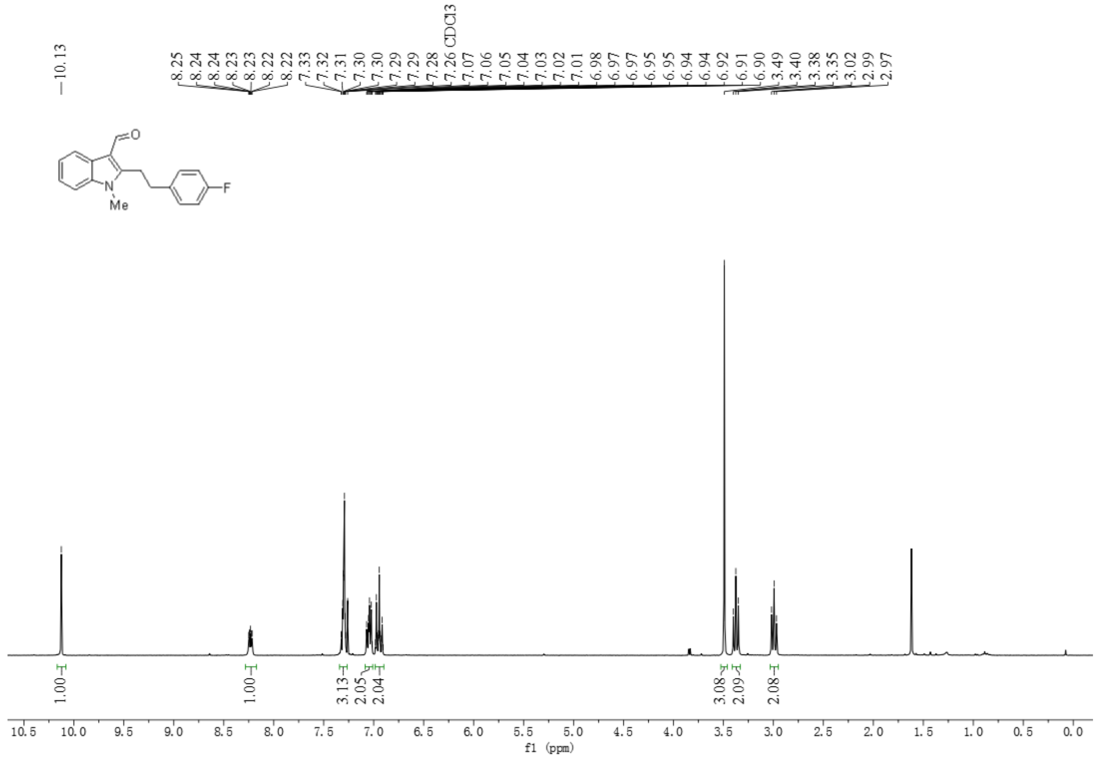

^{13}C NMR spectrum of **71** (75 MHz, $CDCl_3$)

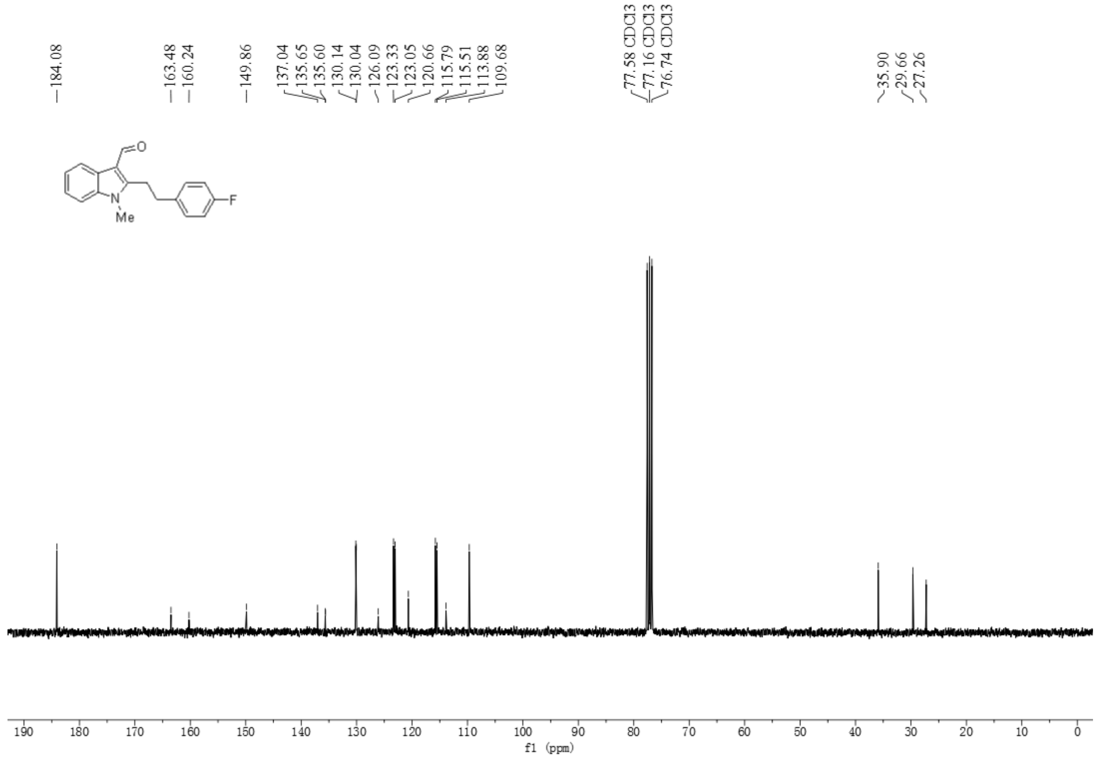

^{19}F NMR spectrum of **71** (282 MHz, $CDCl_3$)

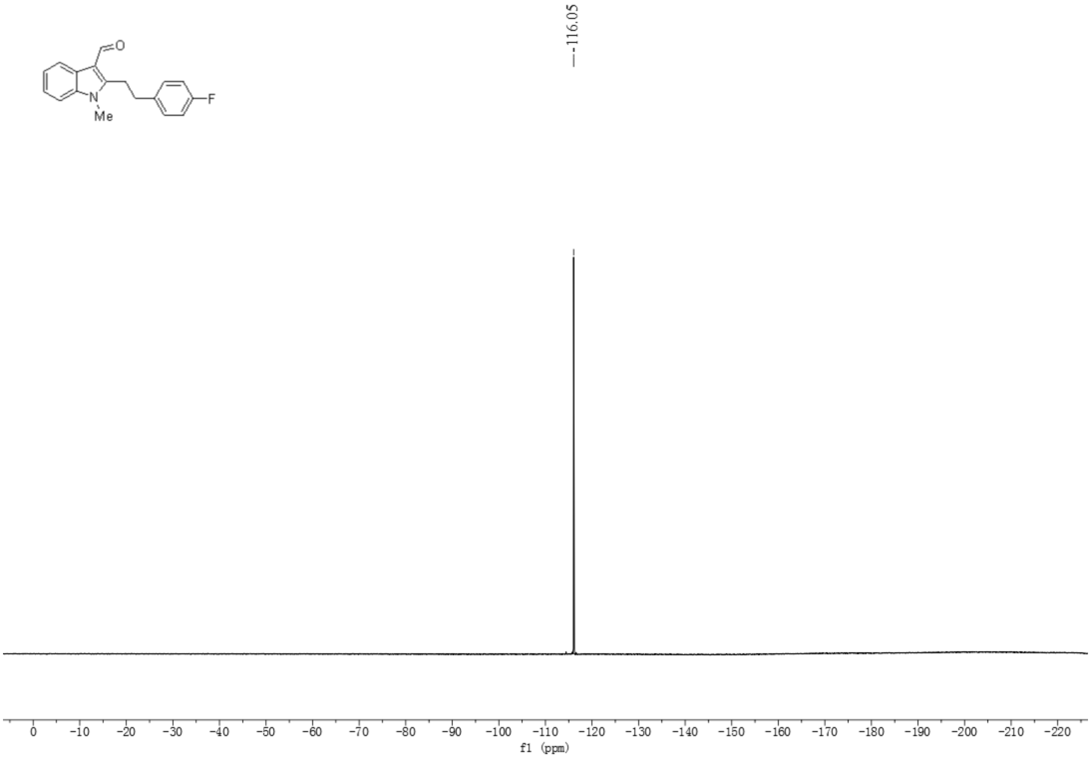

^1H NMR spectrum of **72** (300 MHz, $CDCl_3$)

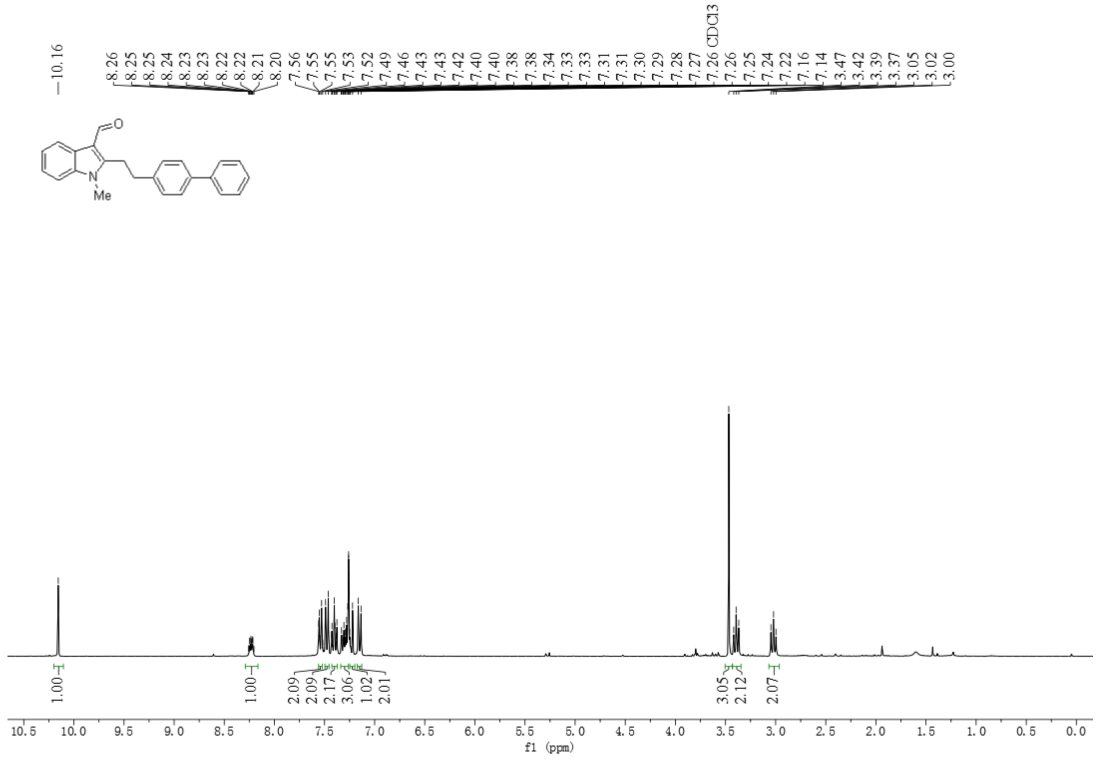

^{13}C NMR spectrum of **72** (75 MHz, $CDCl_3$)

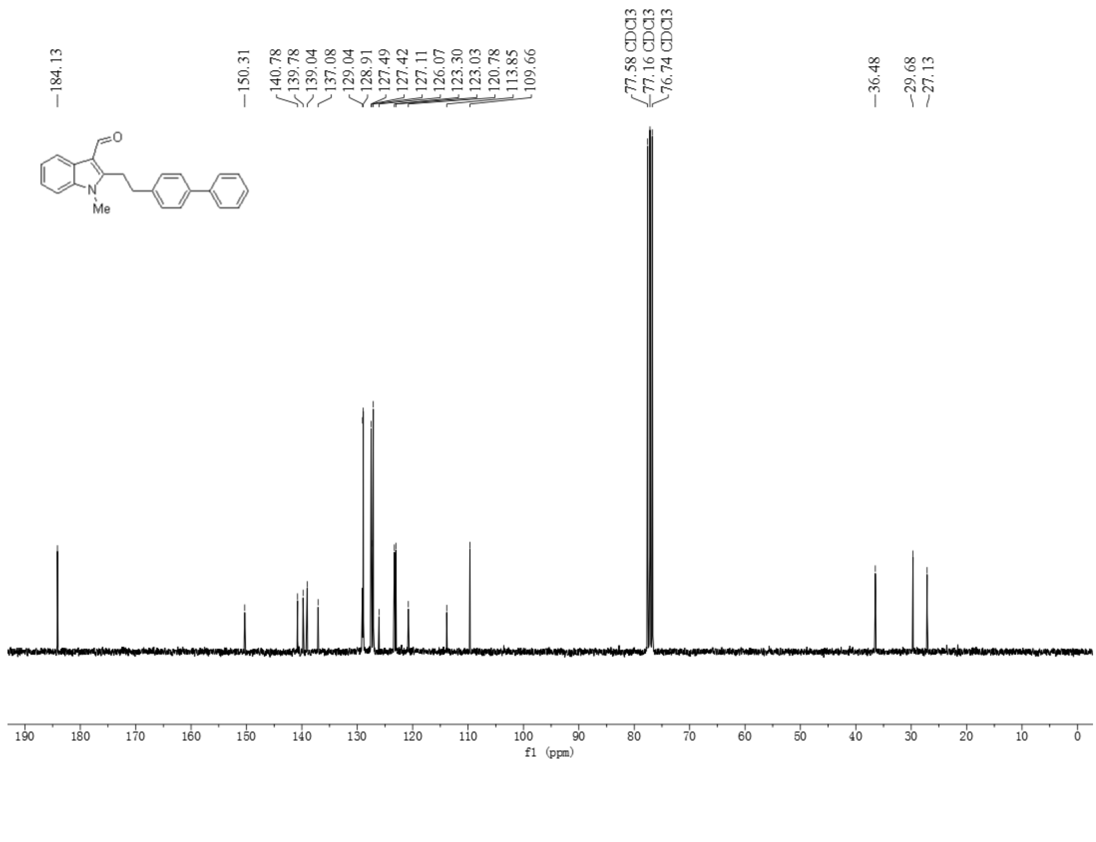

^{1}H NMR spectrum of **73** (300 MHz, $CDCl_3$)

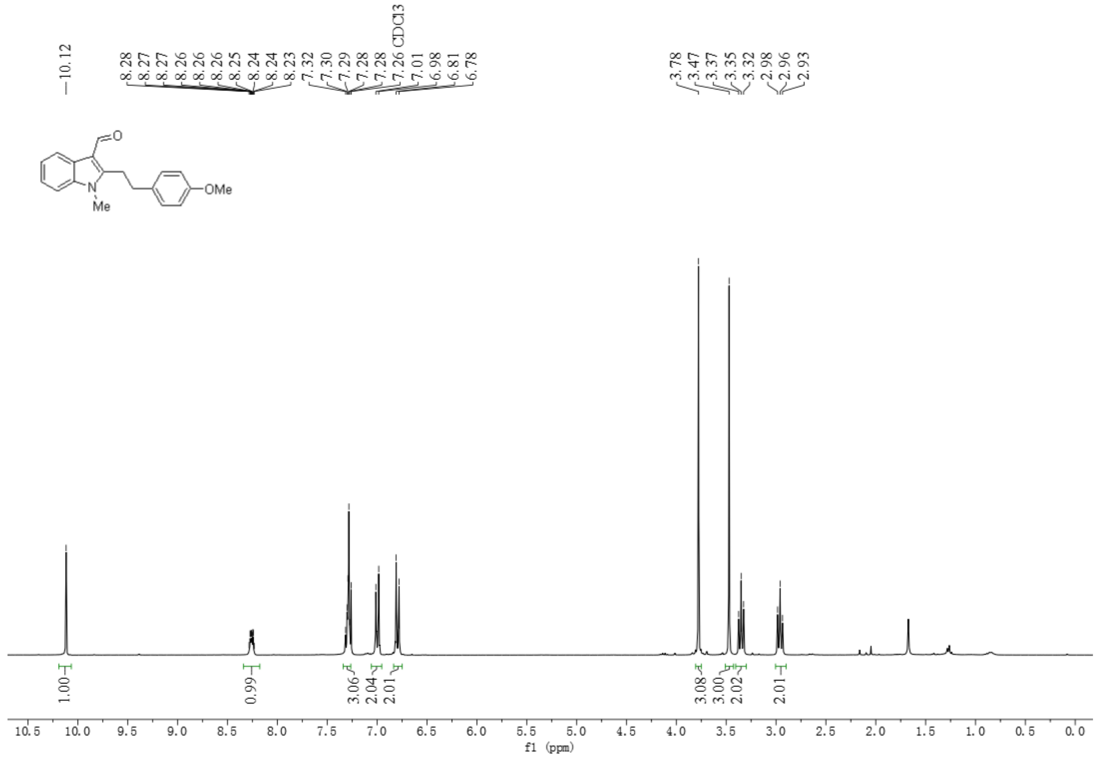

^{13}C NMR spectrum of **73** (75 MHz, $CDCl_3$)

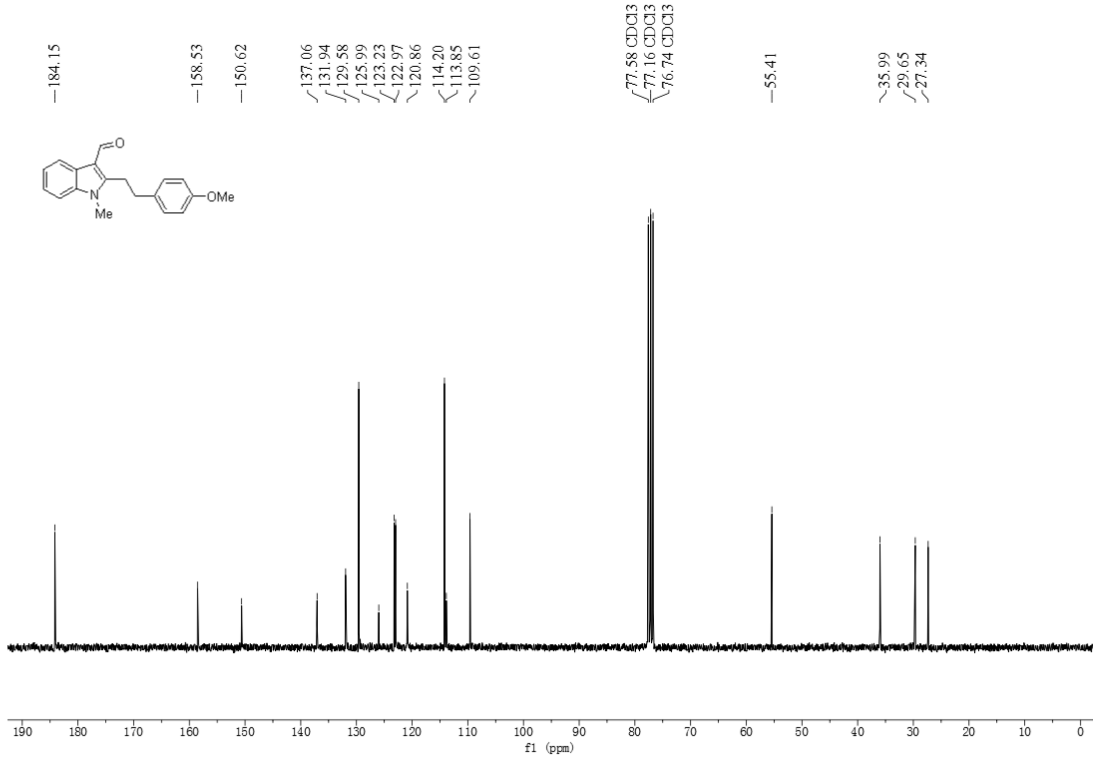

^{1}H NMR spectrum of **74** (300 MHz, $CDCl_3$)

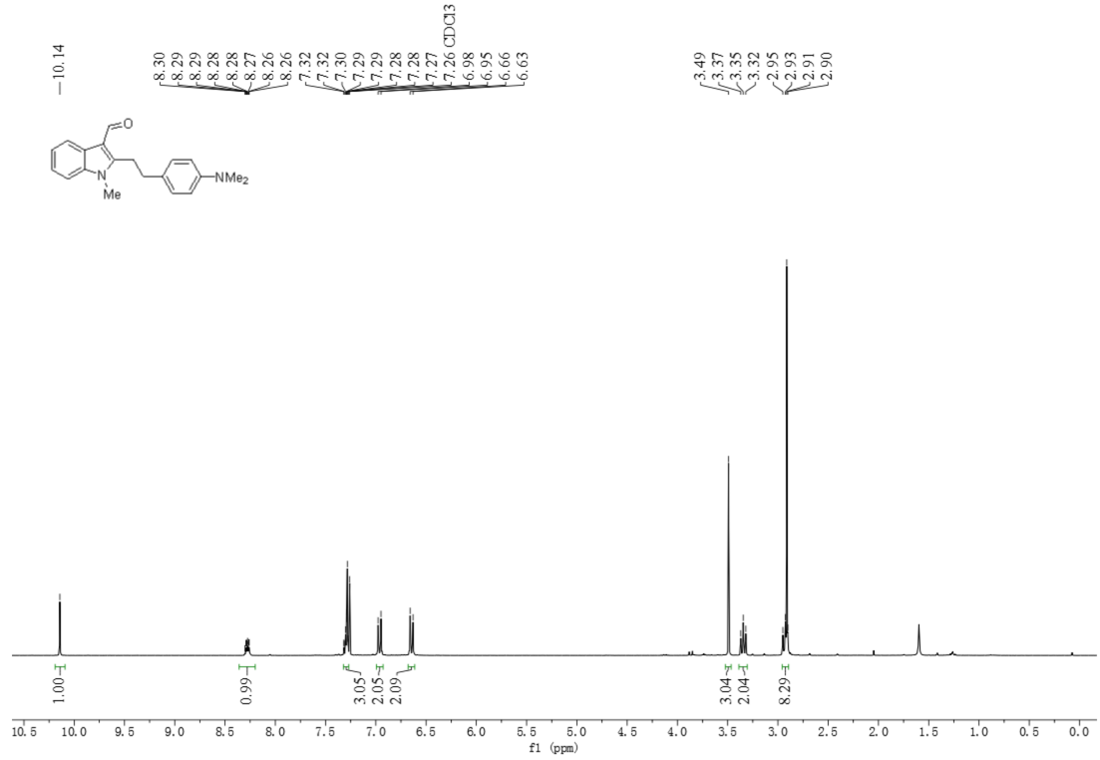

^{13}C NMR spectrum of **74** (75 MHz, $CDCl_3$)

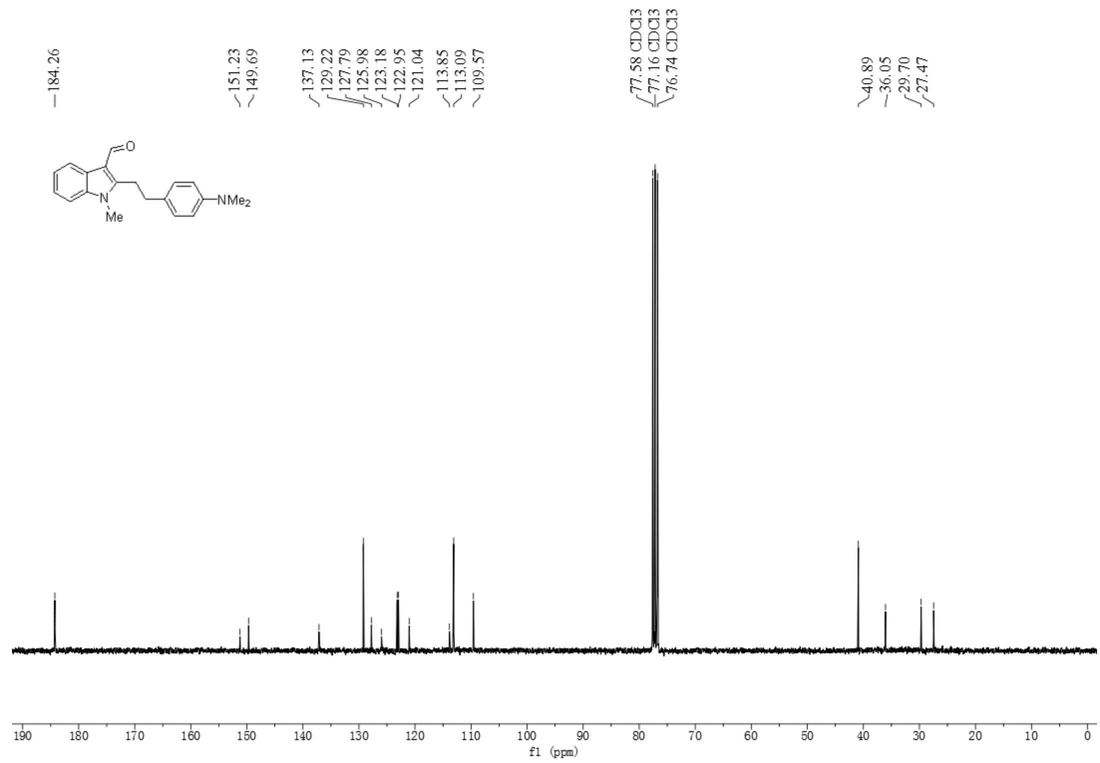

^{1}H NMR spectrum of **75** (300 MHz, $CDCl_3$)

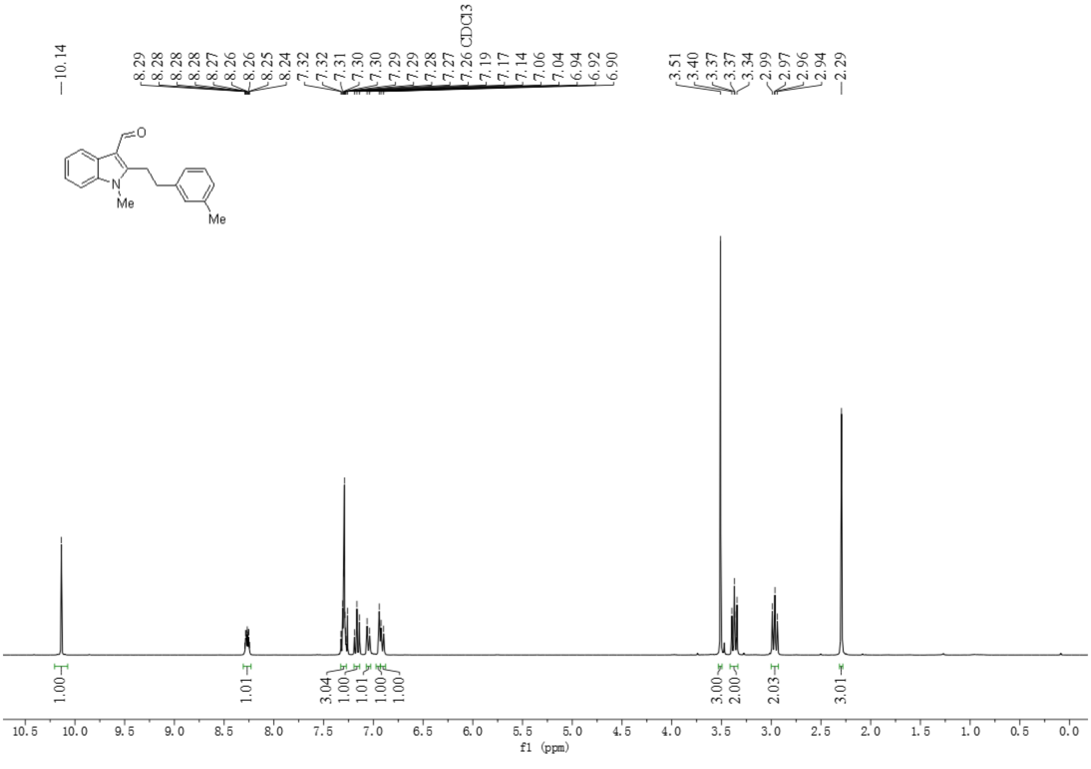

^{13}C NMR spectrum of **75** (75 MHz, $CDCl_3$)

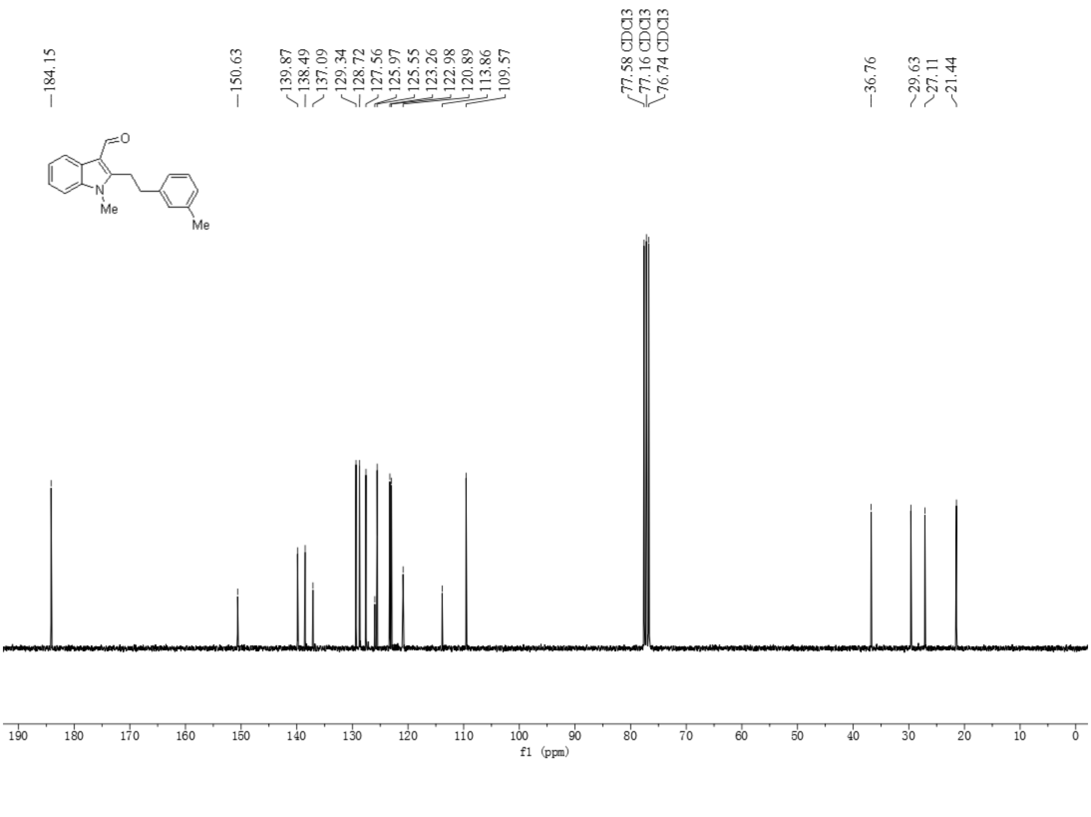

^{1}H NMR spectrum of **76** (300 MHz, $CDCl_3$)

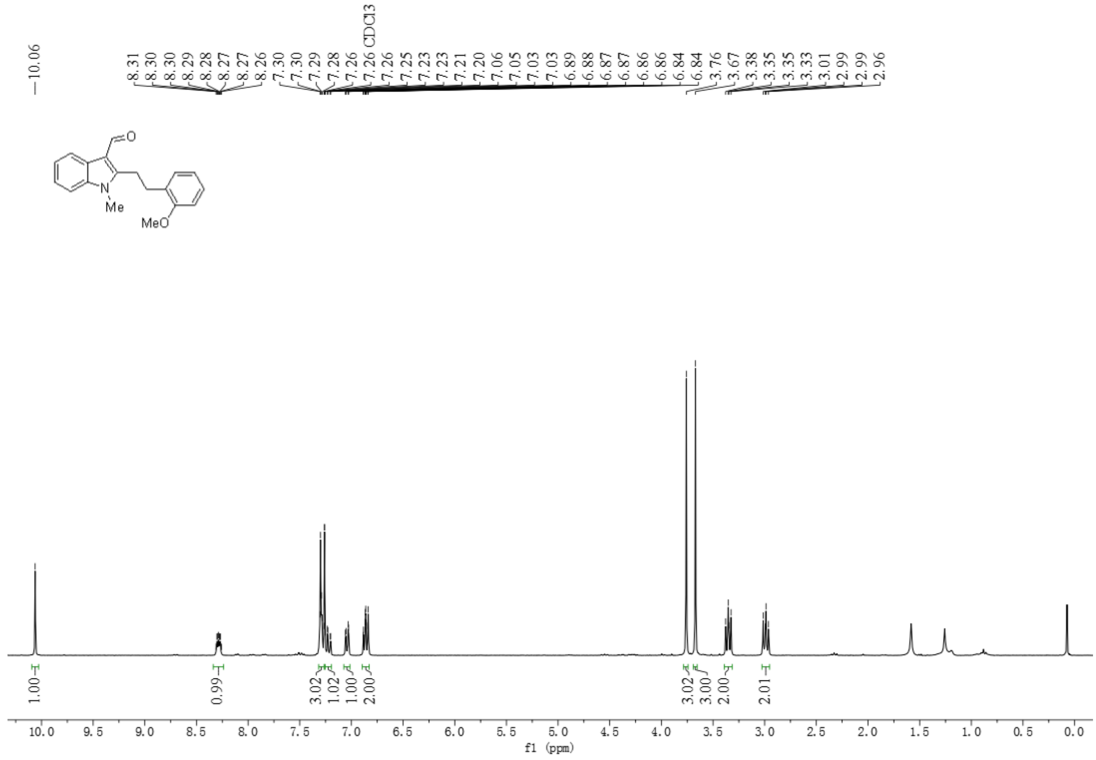

^{13}C NMR spectrum of **76** (75 MHz, $CDCl_3$)

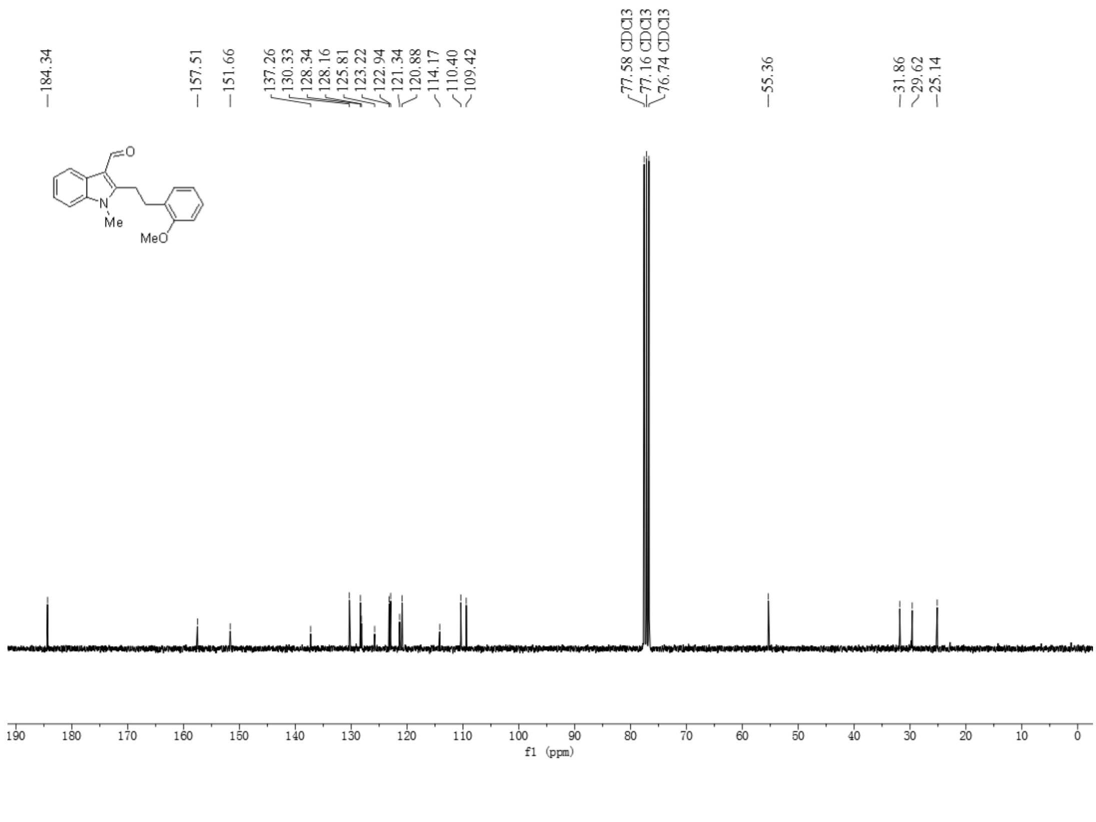

^{1}H NMR spectrum of **77** (300 MHz, $CDCl_3$)

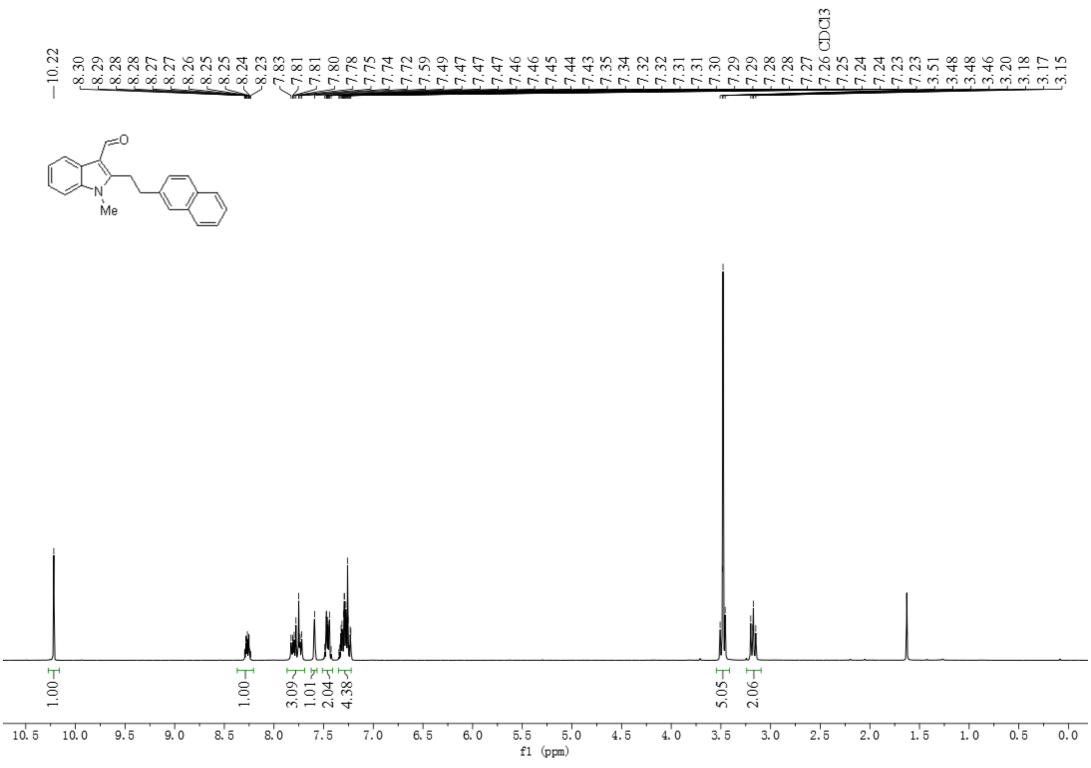

^{13}C NMR spectrum of **77** (75 MHz, $CDCl_3$)

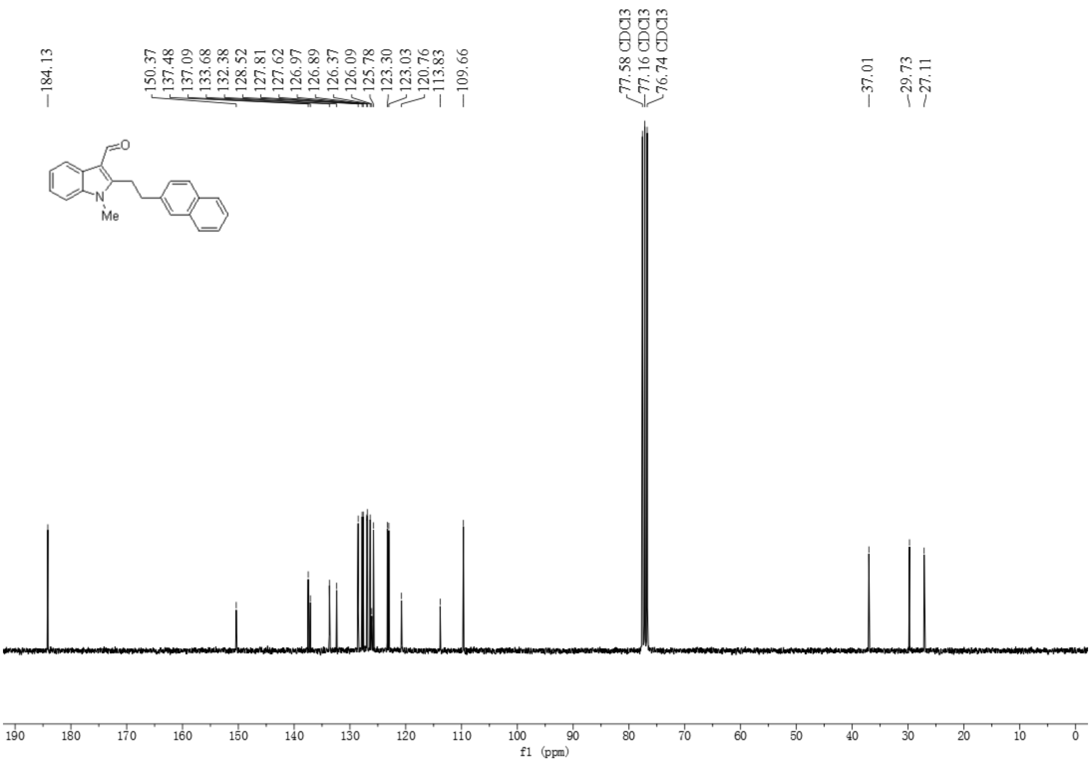

^{1}H NMR spectrum of **78** (300 MHz, $CDCl_3$)

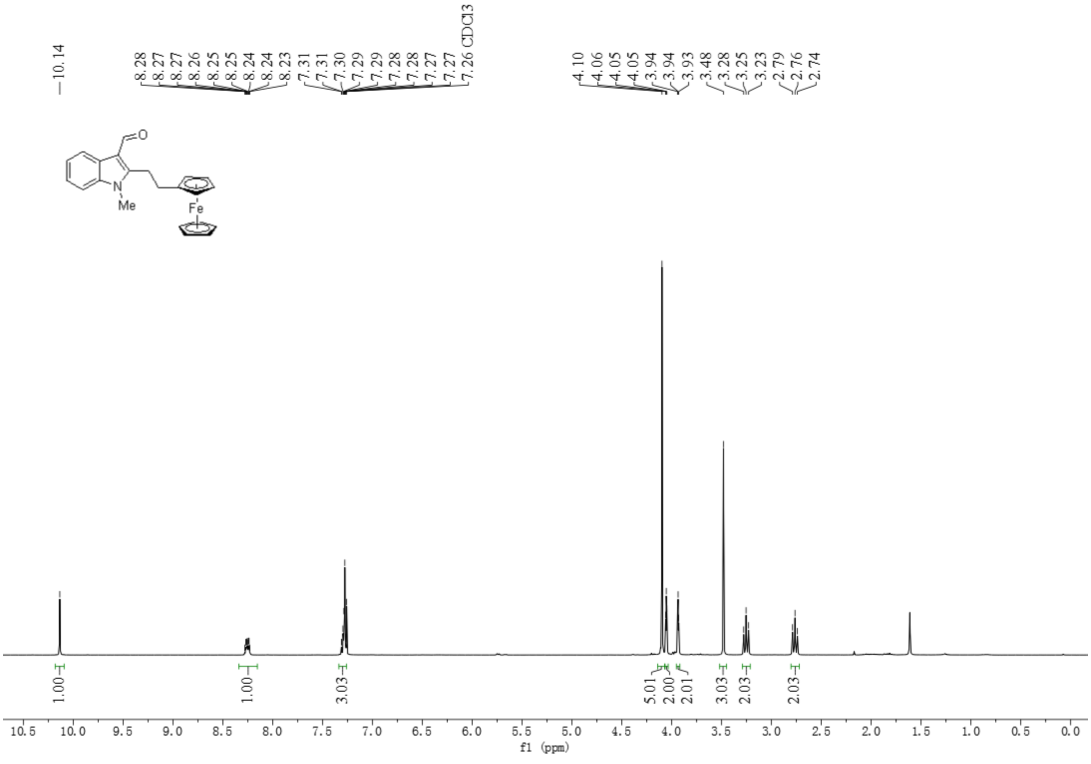

^{13}C NMR spectrum of **78** (75 MHz, $CDCl_3$)

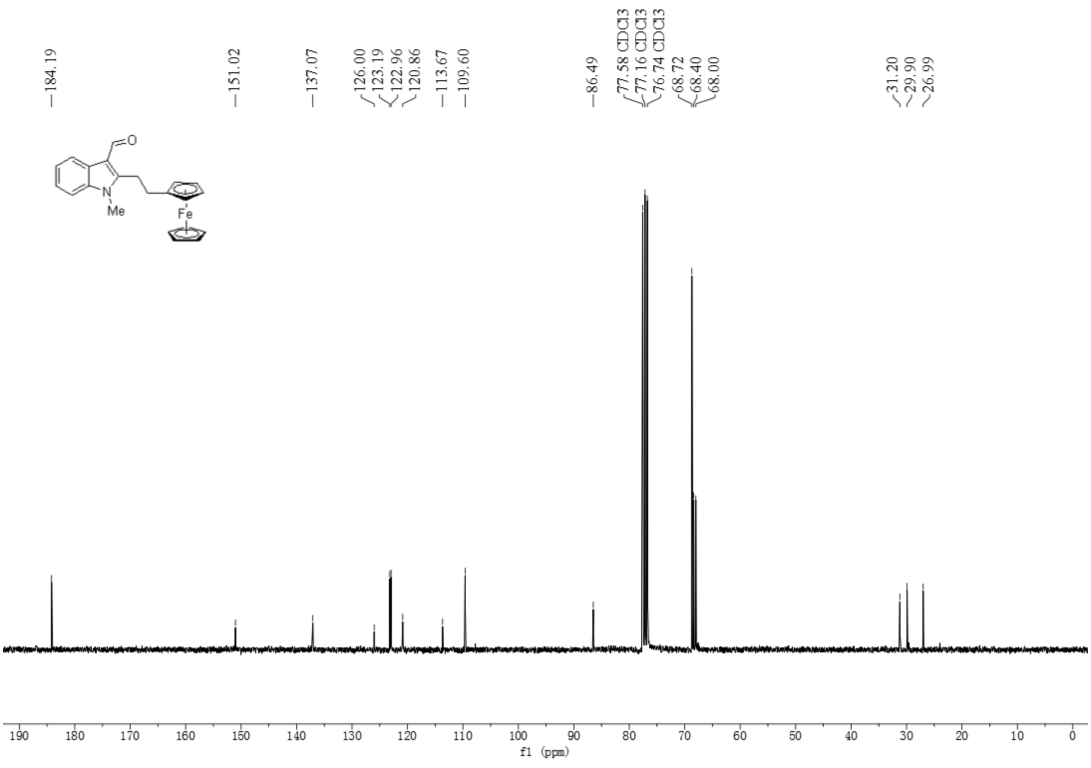

^{1}H NMR spectrum of **79** (300 MHz, $CDCl_3$)

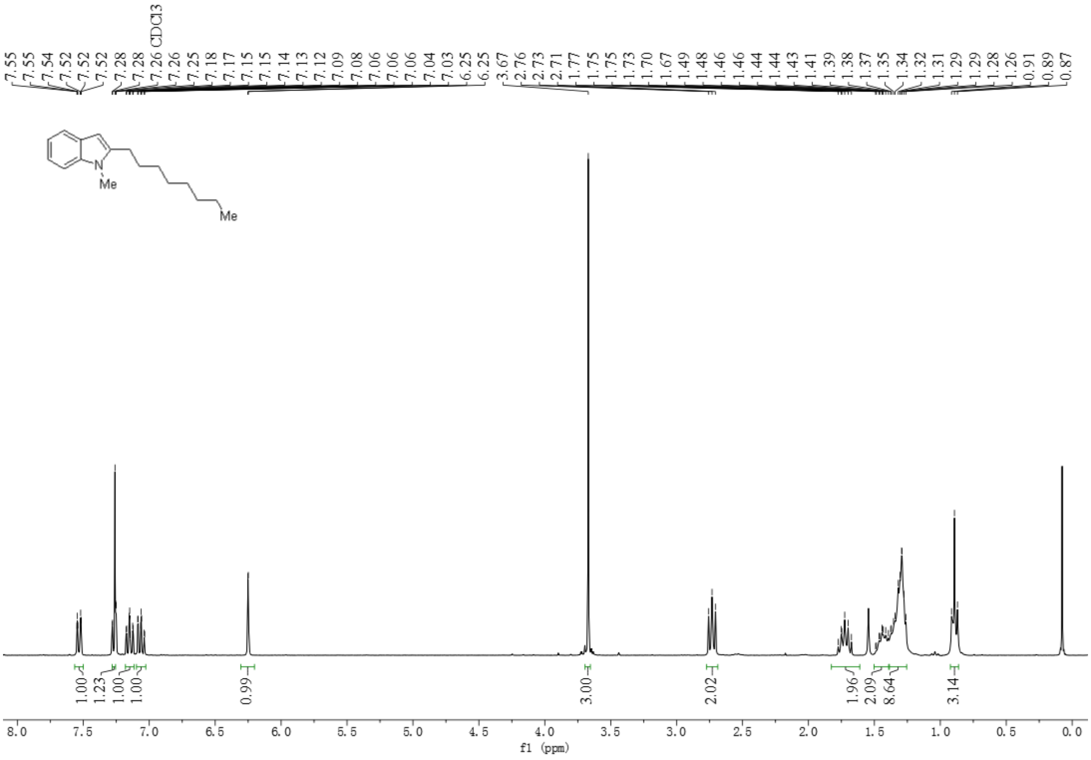

^{13}C NMR spectrum of **79** (75 MHz, $CDCl_3$)

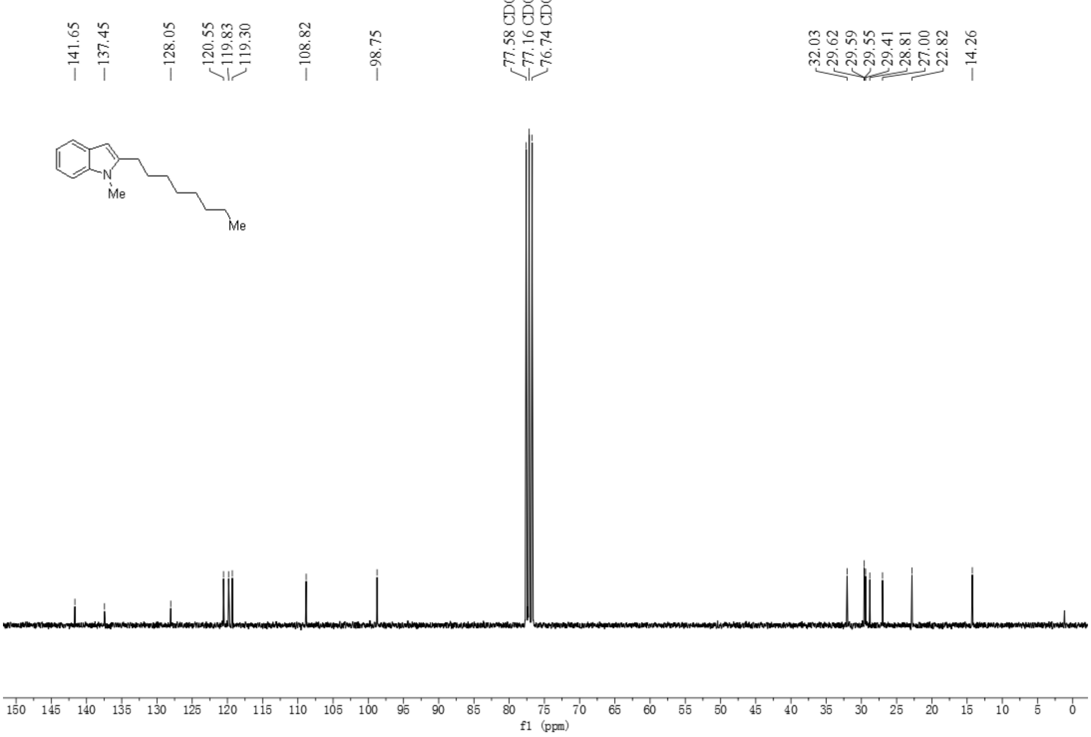

^{1}H NMR spectrum of **80** (300 MHz, $CDCl_3$)

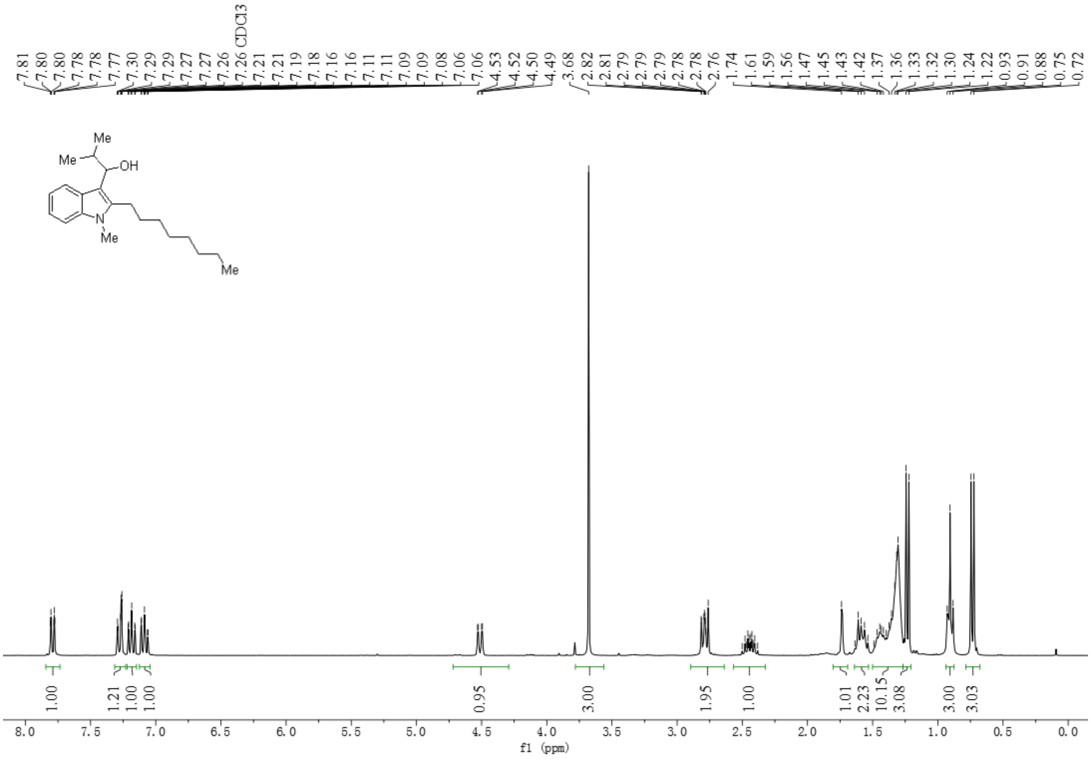

^{13}C NMR spectrum of **80** (75 MHz, $CDCl_3$)

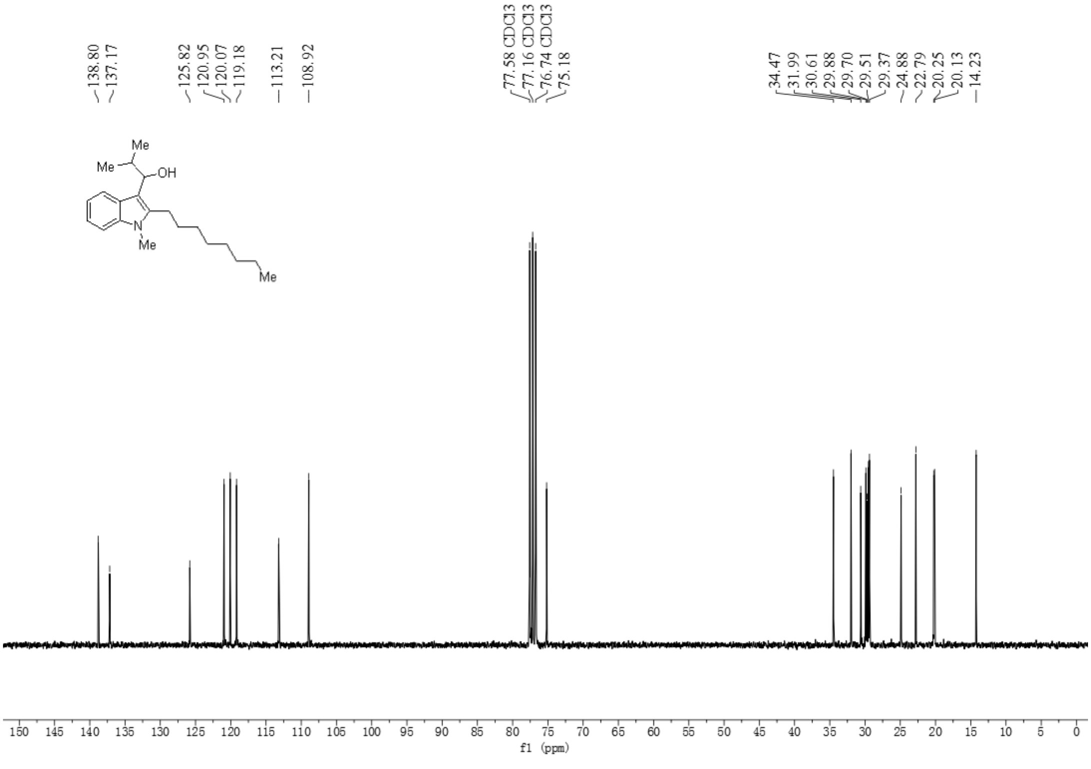

^{1}H NMR spectrum of **81** (300 MHz, $CDCl_3$)

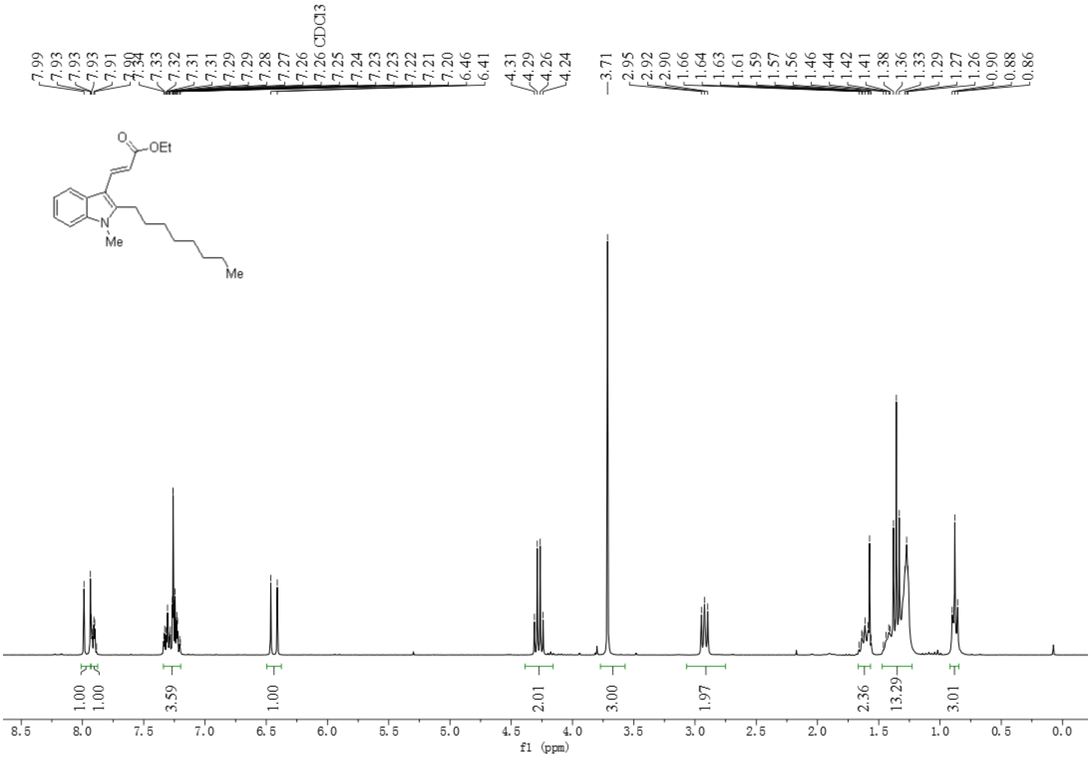

^{13}C NMR spectrum of **81** (75 MHz, $CDCl_3$)

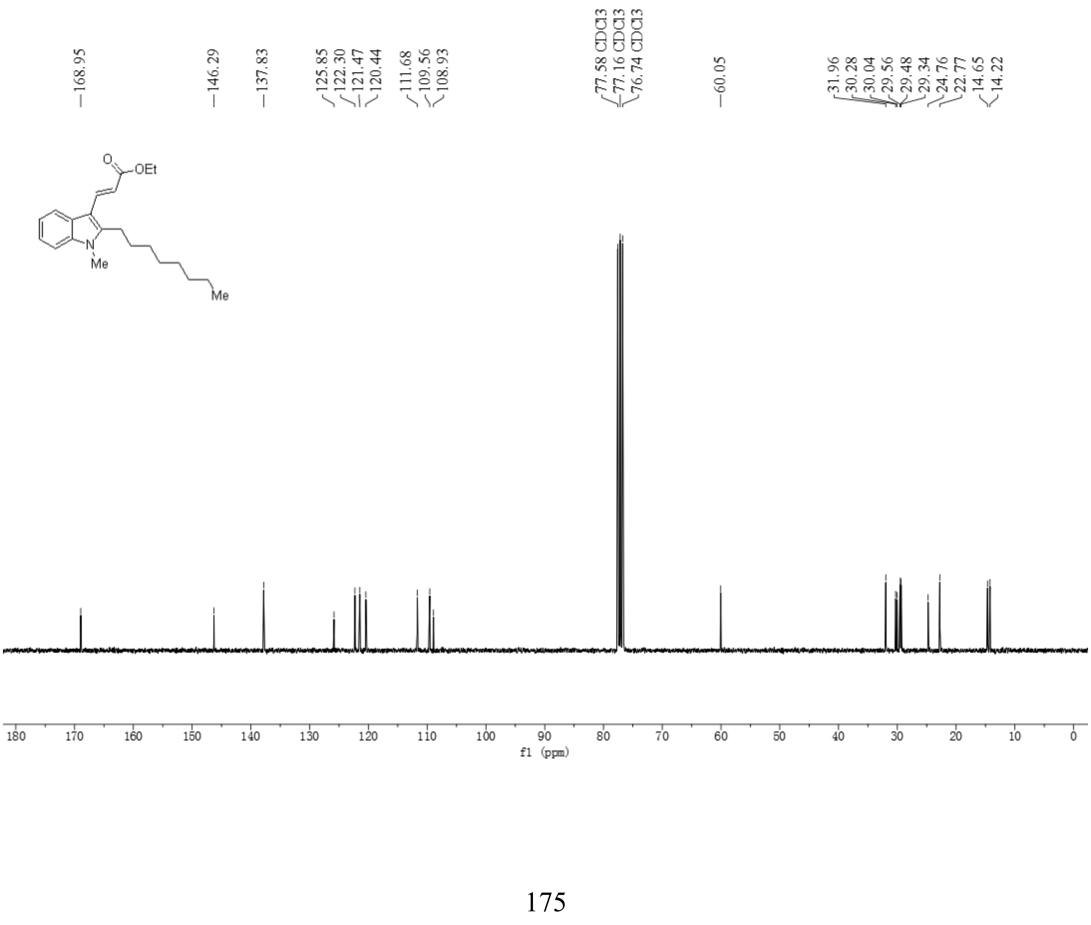

^{1}H NMR spectrum of **82** (300 MHz, $CDCl_3$)

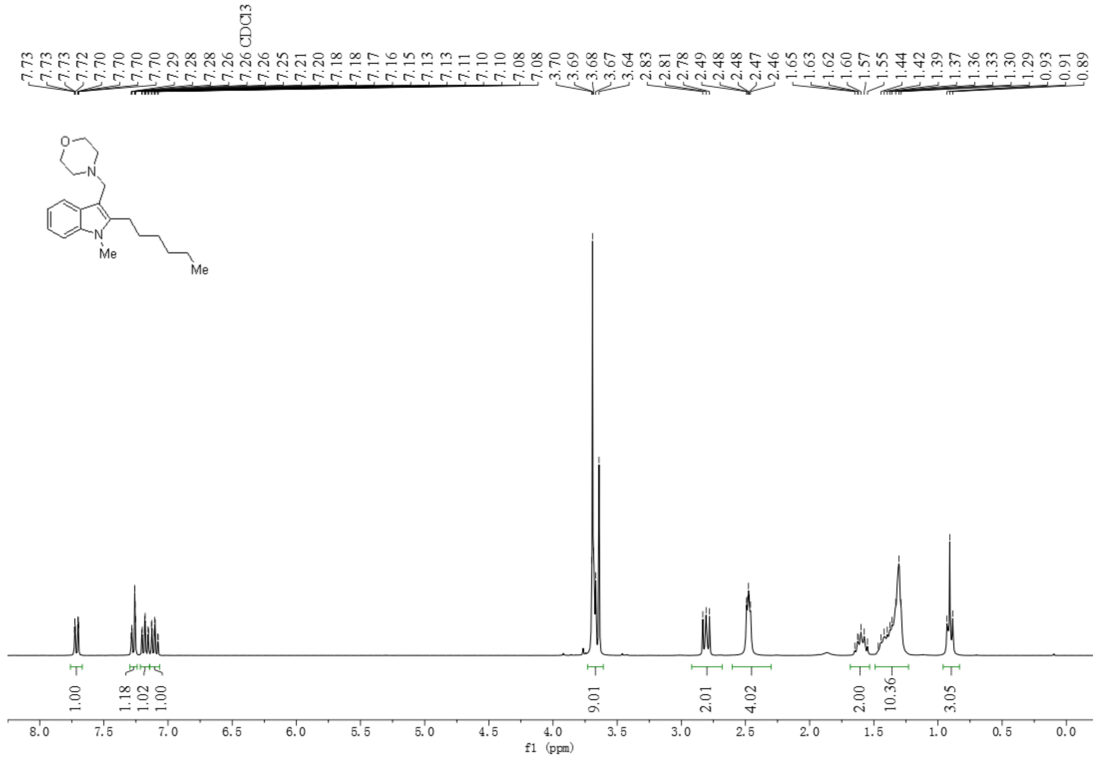

^{13}C NMR spectrum of **82** (75 MHz, $CDCl_3$)

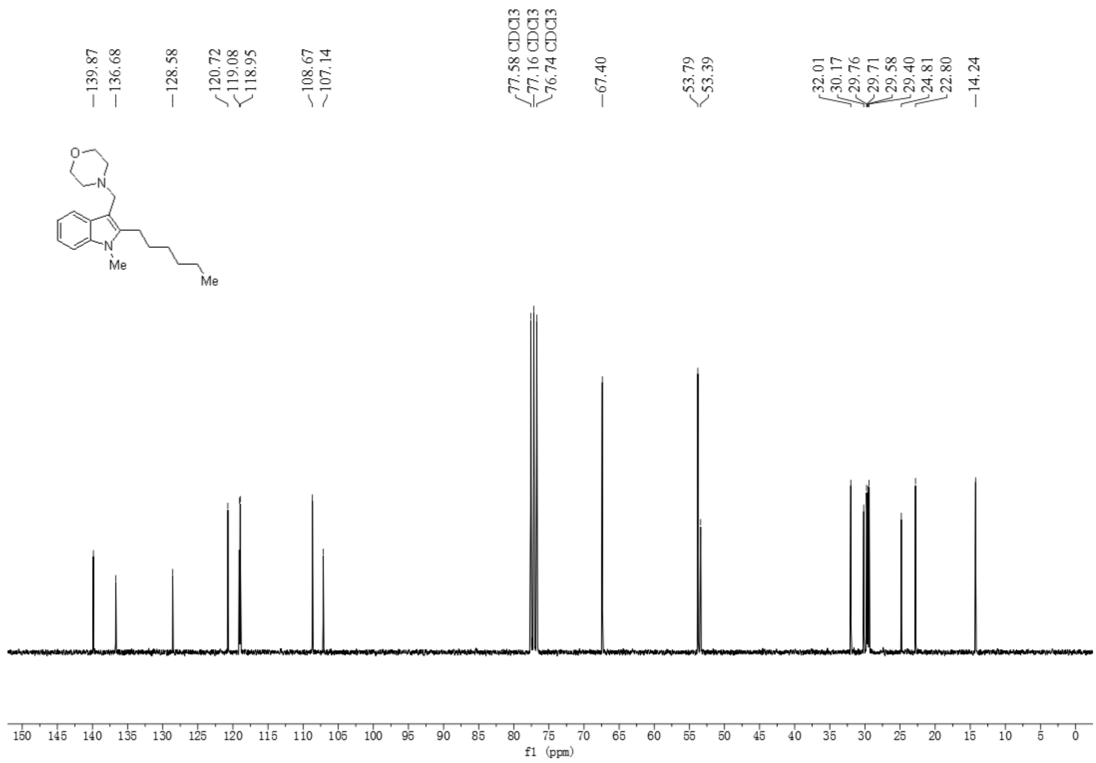

^{1}H NMR spectrum of **83** (300 MHz, DMSO-d_6)

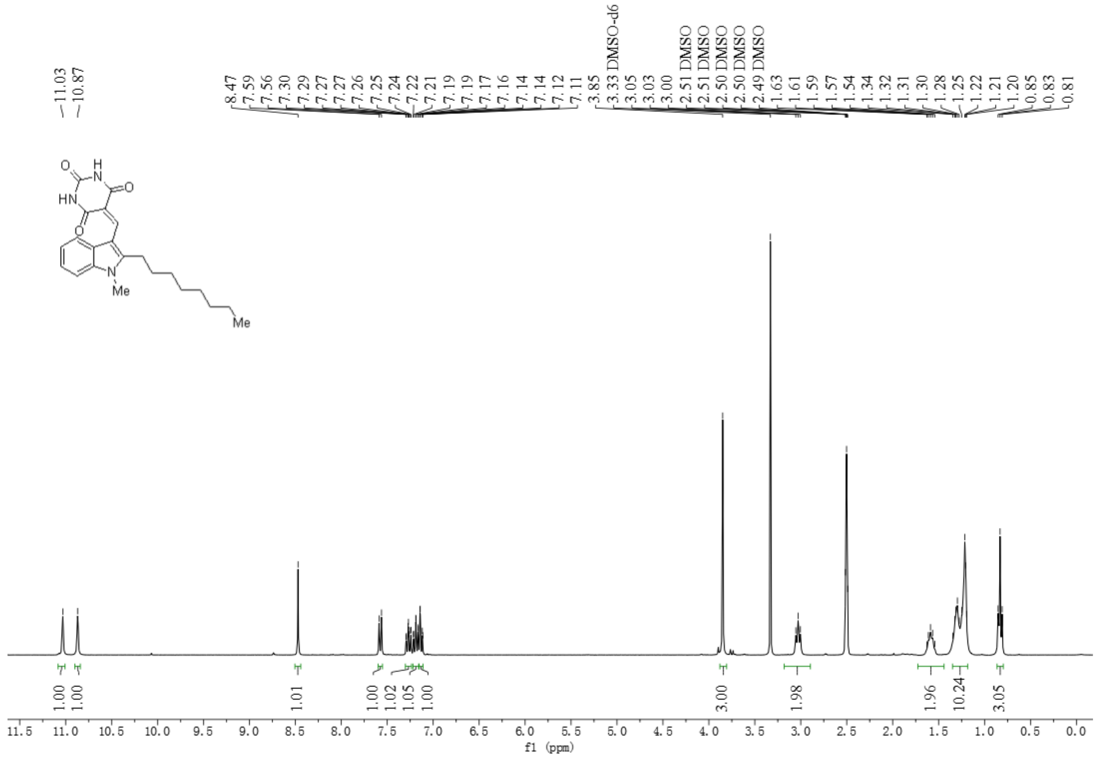

^{13}C NMR spectrum of **83** (75 MHz, DMSO-d_6)

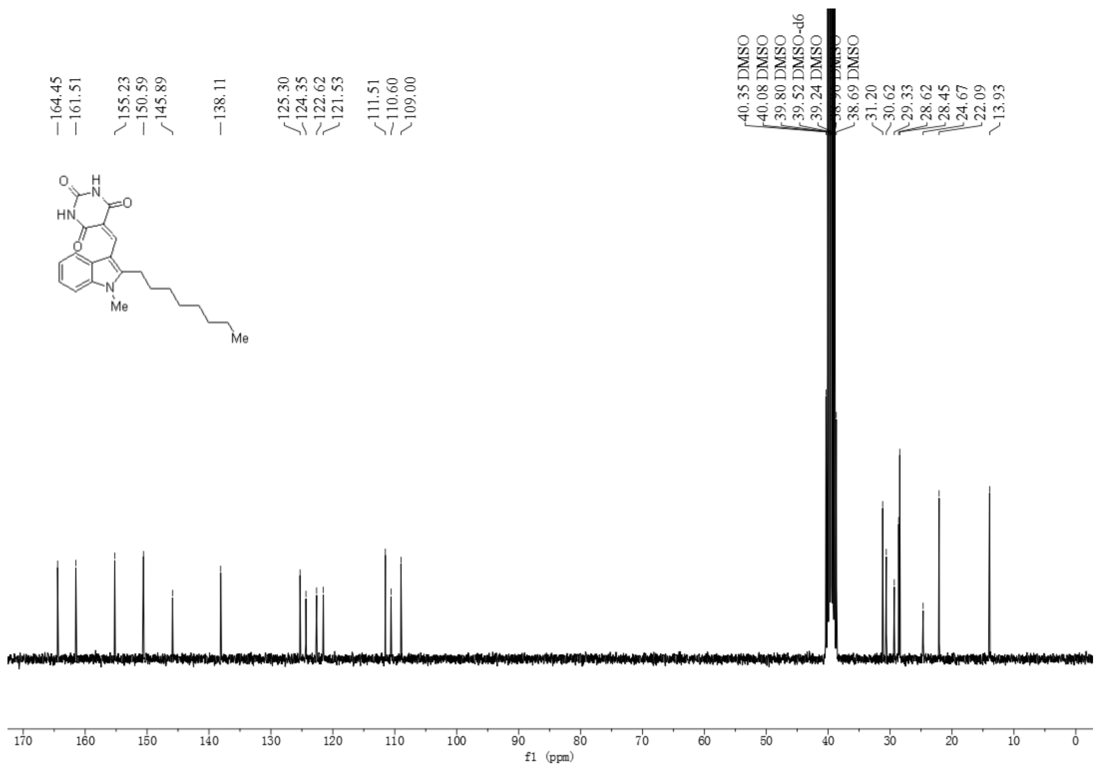

^{1}H NMR spectrum of **84** (300 MHz, $CDCl_3$)

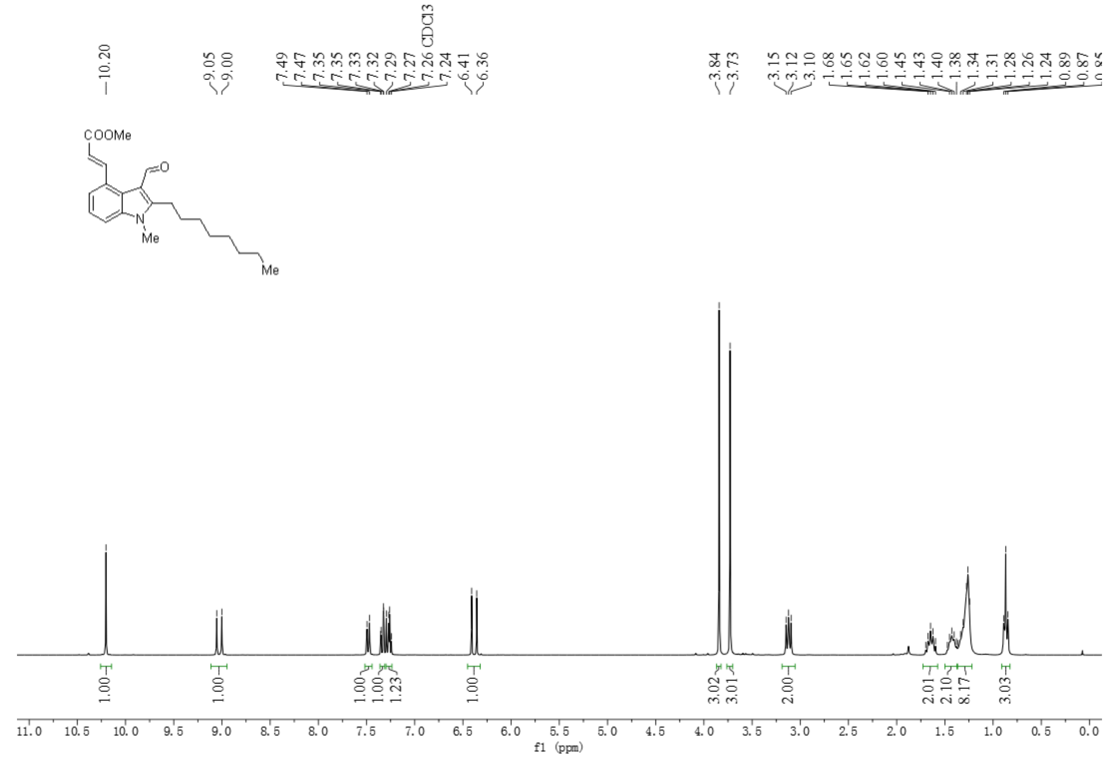

^{13}C NMR spectrum of **84** (75 MHz, $CDCl_3$)

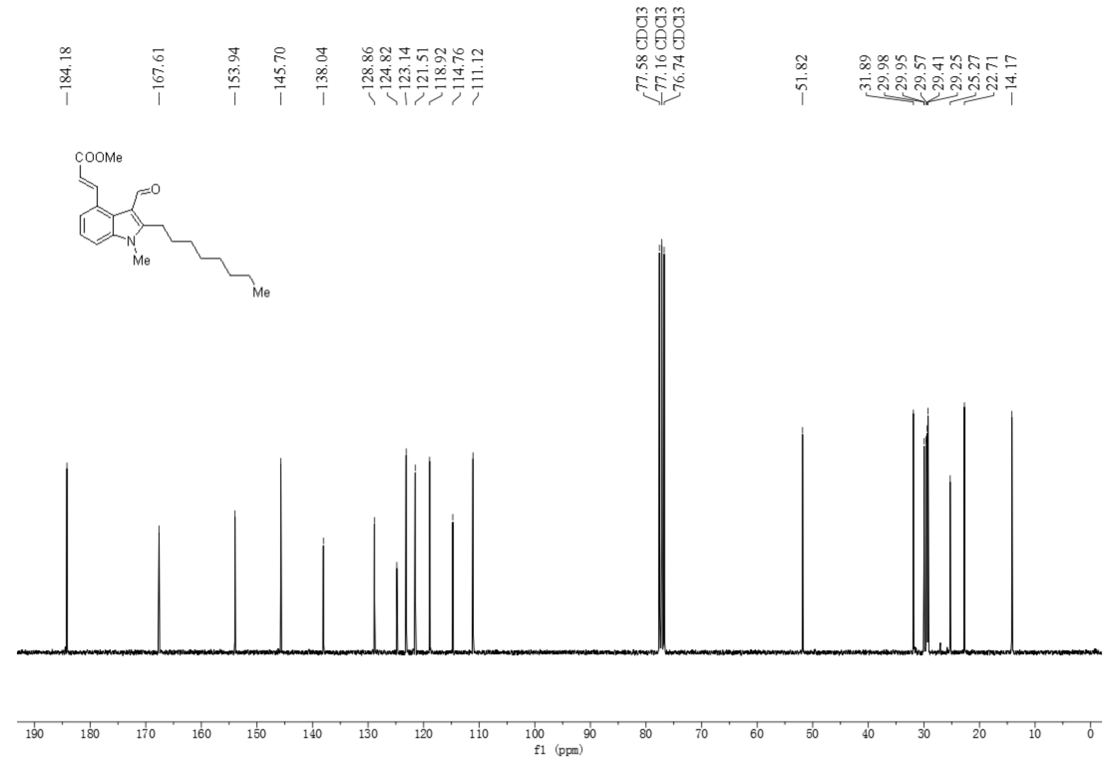

^{1}H NMR spectrum of **85** (300 MHz, $CDCl_3$)

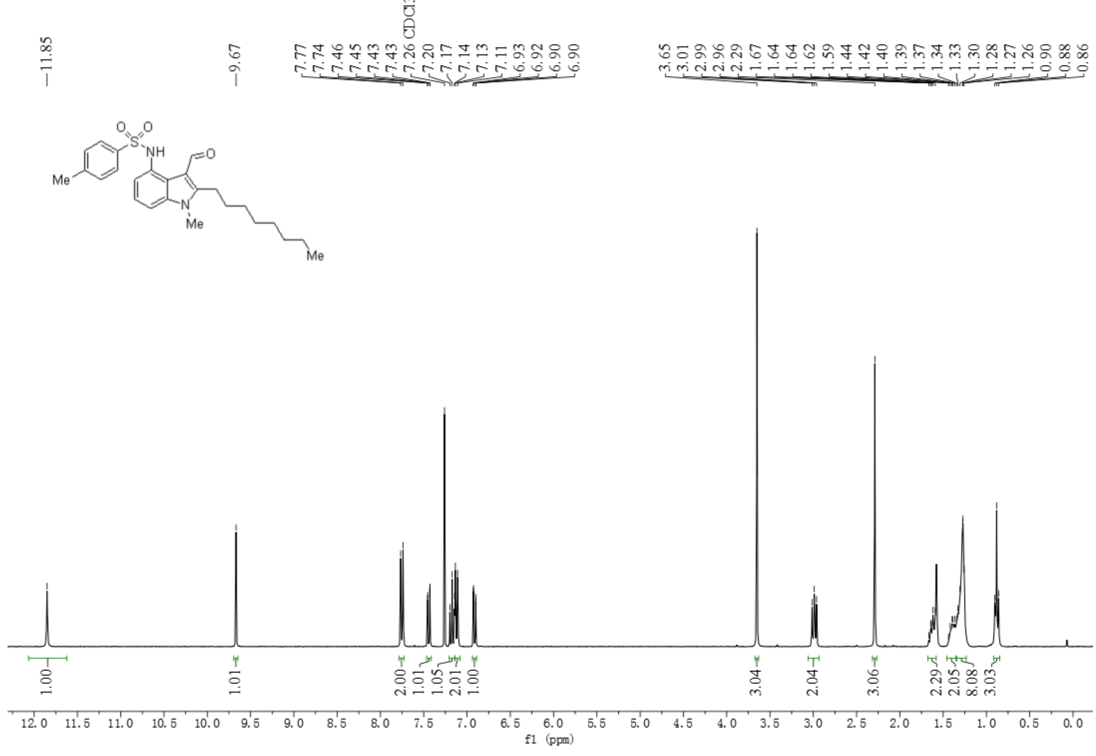

^{13}C NMR spectrum of **85** (75 MHz, $CDCl_3$)

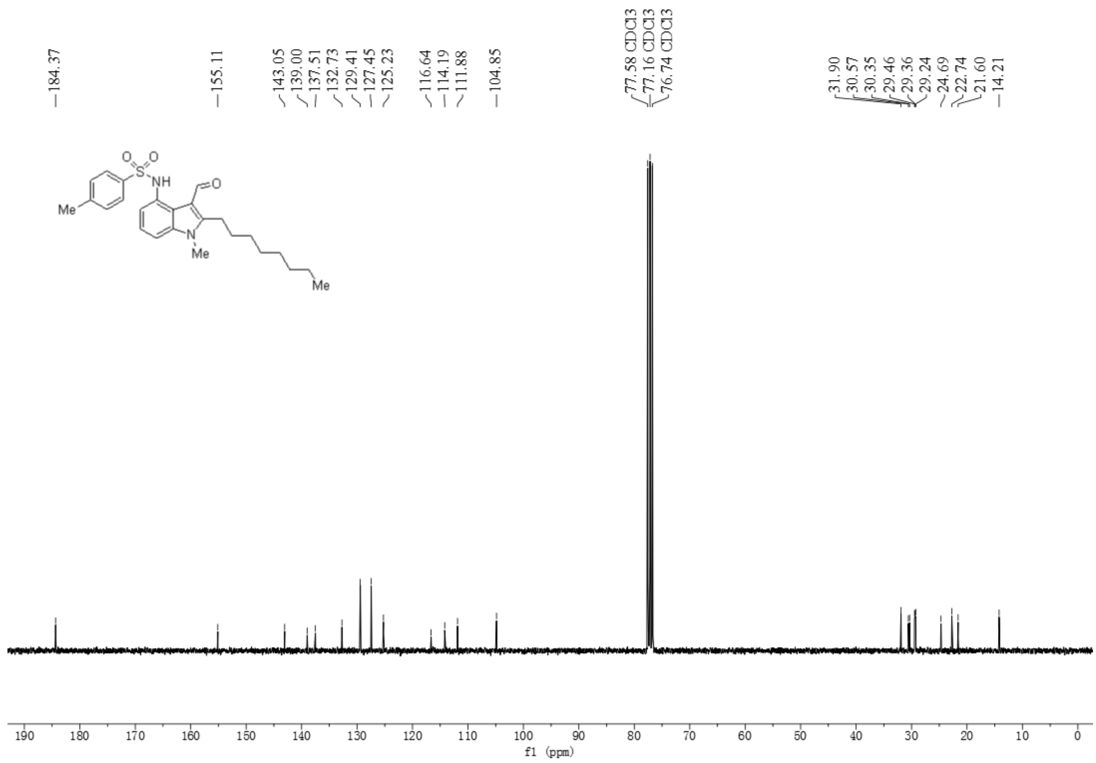

^{1}H NMR spectrum of **86** (300 MHz, $CDCl_3$)

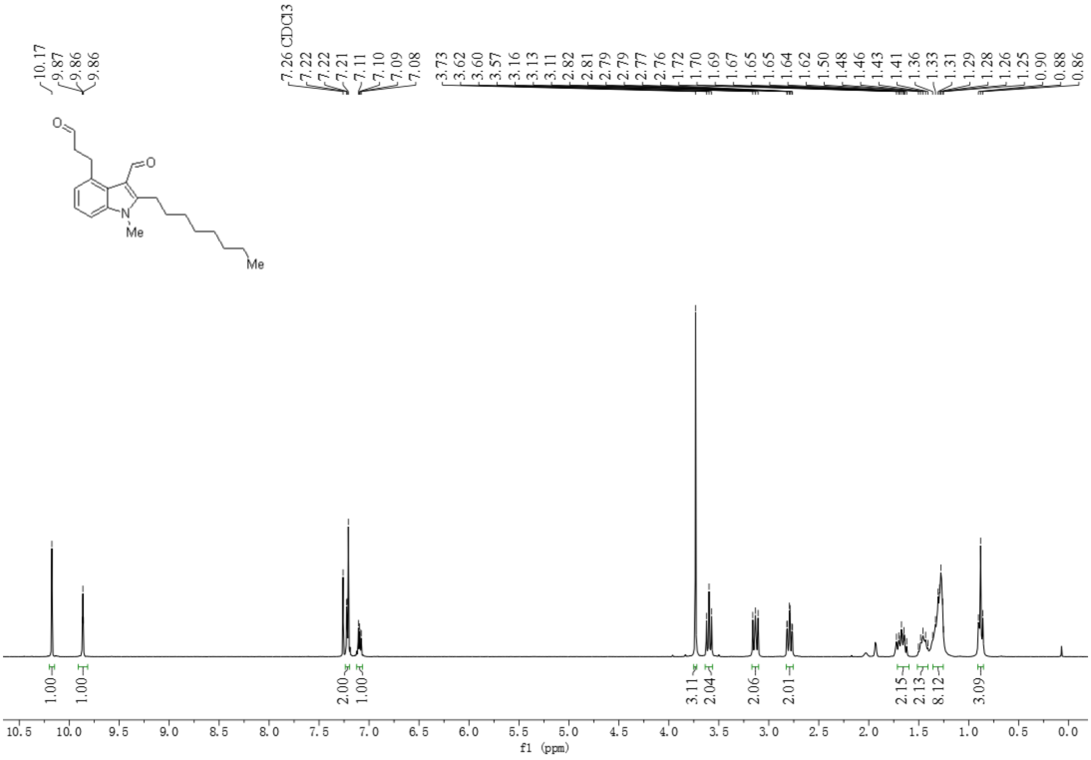

^{13}C NMR spectrum of **86** (75 MHz, $CDCl_3$)

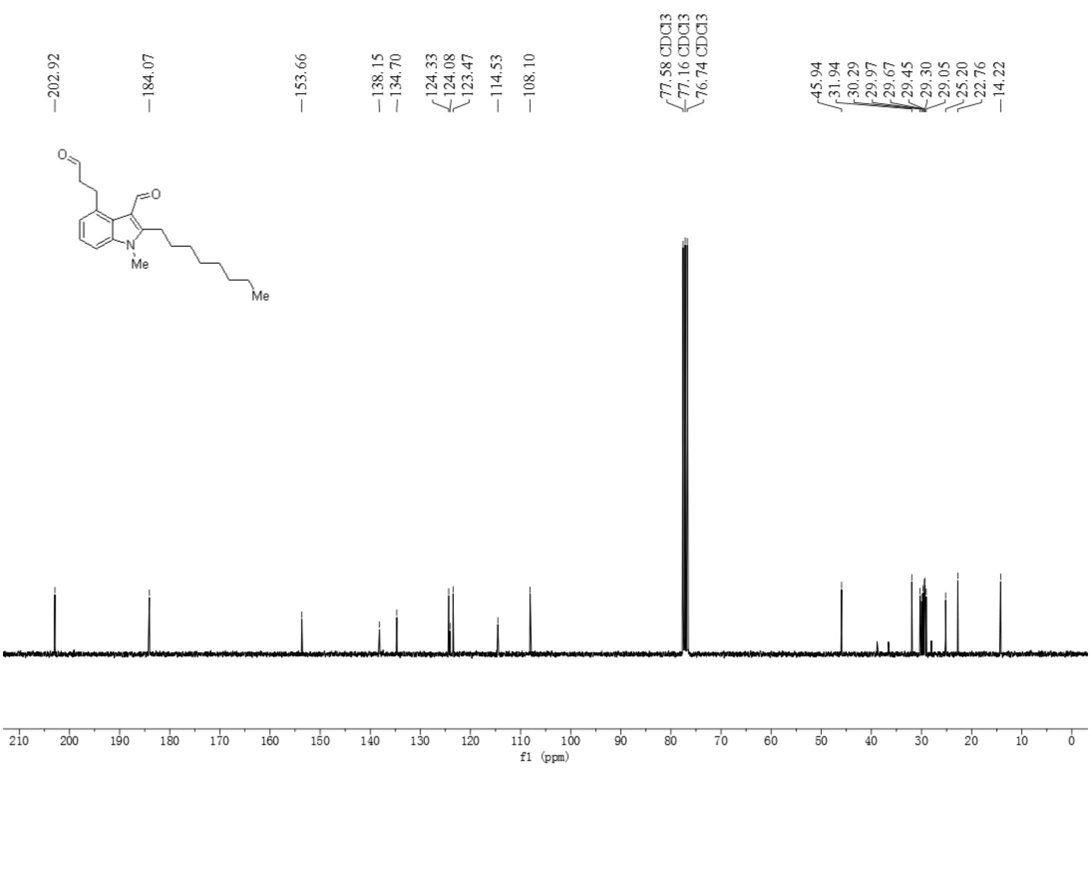

^{1}H NMR spectrum of **87** (300 MHz, $CDCl_3$)

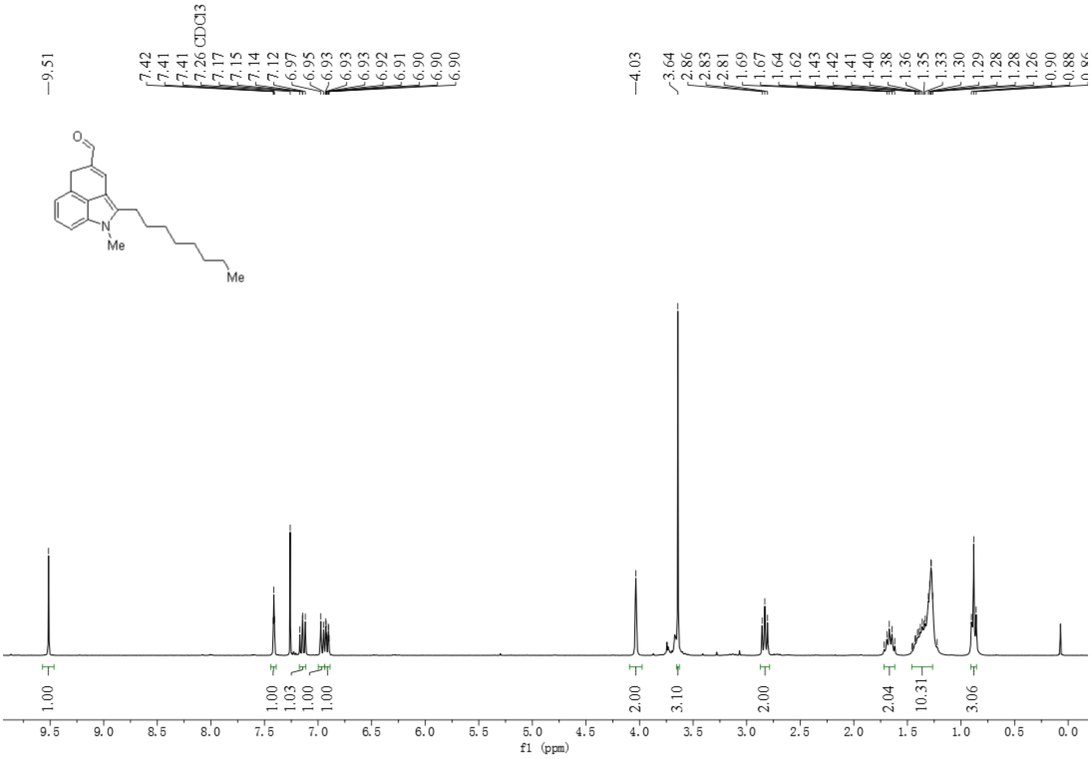

^{13}C NMR spectrum of **87** (75 MHz, $CDCl_3$)

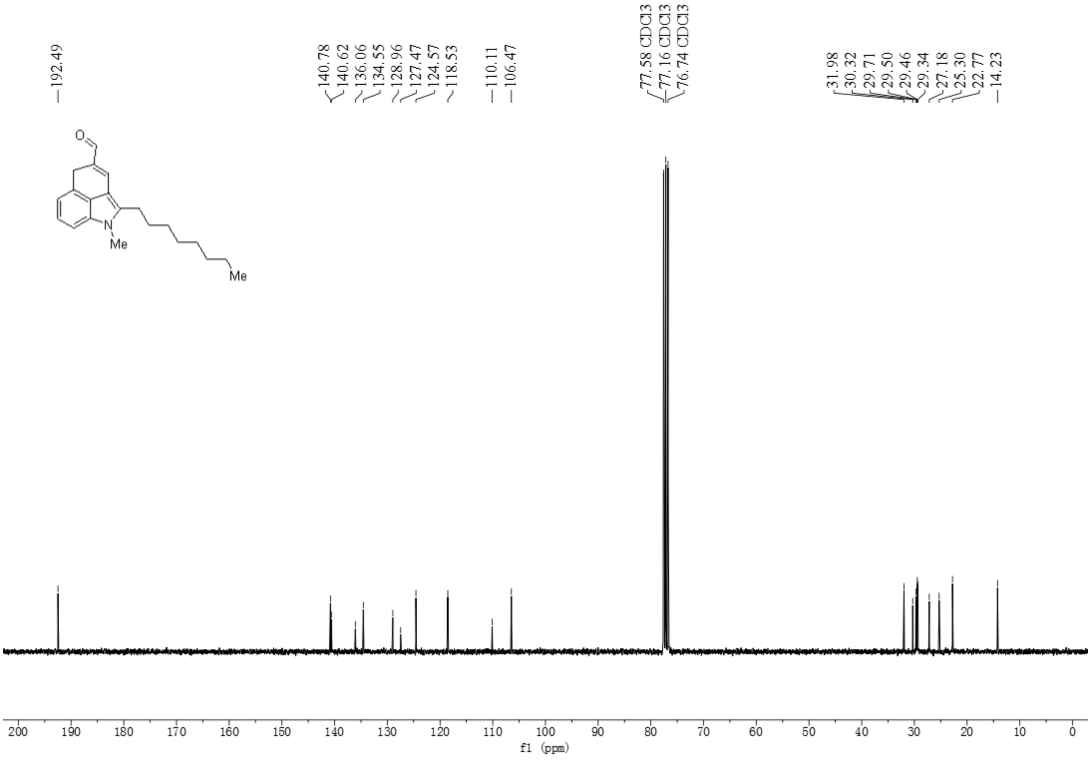

11. References

1. Wong, M. Y., Yamakawa, T. & Yoshikai, N. Iron-catalyzed directed C2-alkylation and alkenylation of indole with vinylarenes and alkynes. *Org. Lett.* **17**, 442−445 (2015).
2. Loup, J. et al. Asymmetric iron-catalyzed C–H alkylation enabled by remote ligand *meta*-substitution. *Angew. Chem. Int. Ed.* **56**, 14197−14201 (2017).
3. Jacob, N., Zaid, Y., Oliveira, J. C. A., Ackermann, L. & Wencel-Delord, J. Cobalt-catalyzed enantioselective C–H arylation of indoles. *J. Am. Chem. Soc.* **144**, 798−806 (2022).
4. Delaude, L., Szypa, M., Demonceau, A. & Noels, A. F. New *in situ* generated ruthenium catalysts bearing *N*-heterocyclic carbene ligands for the ring-opening metathesis polymerization of cyclooctene. *Adv. Synth. Catal.* **344**, 749−756 (2002).
5. Romanov-Michailidis, F., Besnard, C. & Alexakis, A. *N*-Heterocyclic carbene-catalyzed annulation of α-cyano-1,4-diketones with ynals. *Org. Lett.* **14**, 4906–4909 (2012).
6. Lebeuf, R. Hirano, K. & Glorius, F. Palladium-catalyzed C-allylation of benzoins and an NHC-catalyzed three component coupling derived thereof: compatibility of NHC- and Pd-catalysts. *Org. Lett.* **10**, 4243–4246 (2008).
7. Sluijter, S. N., Warsink, S., Lutzc, M. & Elsevier, C. J. Synthesis of palladium(0) and -(II) complexes with chelating bis(*N*-heterocyclic carbene) ligands and their application in semihydrogenation. *Dalton Trans.* **42**, 7365–7372 (2013).
8. Koch, A., Krieck, S., Görls, H. & Westerhausen, M. Alkaline earth metal–carbene complexes with the versatile tridentate 2,6-bis(3-mesitylimidazol-2-ylidene)pyridine ligand. *Organometallics* **36**, 994–1000 (2017).
9. Wu, C.-Y. et al. Ruthenium-catalyzed annulation of alkynes with amides via formyl translocation. *Chem. Commun.* **48**, 3197−3199 (2012).
10. Yamakawa, T. & Yoshikai, N. Alkene isomerization–hydroarylation tandem catalysis: indole C2-alkylation with aryl-substituted alkenes leading to 1,1-diarylalkanes. *Chem. Asian J.* **9**, 1242−1246 (2014).

11. Xu, W. & Yoshikai, N. Highly linear selective cobalt-catalyzed addition of aryl imines to styrenes: reversing intrinsic regioselectivity by ligand elaboration. *Angew. Chem. Int. Ed.* **53**, 14166−14170 (2014).
12. Meyer, S., Orben, C. M., Demeshko, S., Dechert, S. & Meyer, F. Synthesis and characterization of di- and tetracarbene iron(II) complexes with chelating *N*-heterocyclic carbene ligands and their application in aryl Grignard−alkyl halide cross-coupling. *Organometallics* **30**, 6692−6702 (2011).
13. Frisch, M. J. et al. Gaussian 16, Revision A.03, Gaussian, Inc., Wallingford CT (2016).
14. Becke, A. D. Density-functional thermochemistry. III. The role of exact exchange. *J. Chem. Phys*. **98**, 5648−5652 (1993).
15. Lee, C., Yang, W. & Parr, R. G. Development of the Colle-Salvetti correlation-energy formula into a functional of the electron density. *Phys. Rev. B: Condens. Matter Mater. Phys*. **37**, 785−789 (1998).
16. Grimme, S., Ehrlich, S. & Goerigk, L. Effect of the damping function in dispersion corrected density functional theory. *J. Comp. Chem.* **32**, 1456−1465 (2011).
17. Weigend, F. & Ahlrichs, R. Balanced basis sets of split valence, triple zeta valence and quadruple zeta valence quality for H to Rn: Design and assessment of accuracy. *Physical Chemistry Chemical Physics* **7**, 3297–3305 (2005).
18. Tao, J. M., Perdew, J. P., Staroverov, V. N. & Scuseria, G. E. Climbing the Density Functional Ladder: Nonempirical Meta–Generalized Gradient Approximation Designed for Molecules and Solids. *Phys. Rev. Lett.* **91**, 146401 (2003).
19. Staroverov, V. N., Scuseria, G. E., Tao, J. & Perdew, J. P. Comparative assessment of a new nonempirical density functional: Molecules and hydrogen-bonded complexes. *J. Chem. Phys.* **119**, 12129 (2003).
20. Marenich, A. V., Cramer, C. J. & Truhlar, D. G. Universal Solvation Model Based on Solute Electron Density and on a Continuum Model of the Solvent Defined by the Bulk Dielectric Constant and Atomic Surface Tensions. *J. Phys. Chem. B*. **113**, 6378−6396 (2009).
21. Legault, C. Y. CYLview, version 1.0b; Université de Sherbrooke (2009)

(http://www.cylview.org).

22. Guo, J., Minko, Y., Santiago, C. B., Sigman, M. S. Developing comprehensive computational parameter sets to describe the performance of pyridine-oxazoline and related ligands. *ACS Catal.* **7**, 4144–4151 (2017).
23. Bilbrey, J. A., Kazez, A. H., Locklin, J. & Allen, W. D. Exact ligand cone angles. *J. Comput. Chem.* **34,** 1189−1197 (2013).
24. Falivene, L. et al. SambVca 2. a web tool for analyzing catalytic pockets with topographic steric maps. *Organometallics* **35**, 2286−2293 (2016).
