# Supplementary material for: Mechanochemical ligand-controlled regiodivergent hydroarylation of alkenes via iron-catalyzed C−H activation
Source: Nat Commun. 2025 Dec 26;17:150. doi: 10.1038/s41467-025-66806-5 (PMC12774961; doi:10.1038/s41467-025-66806-5)
Supplement: Supplementary file 2 — Description of Additional Supplementary Files [file 41467_2025_66806_MOESM2_ESM.pdf]

Description of Additional Supplementary Files

File Name: Supplementary Data 1

Description: DFT-optimized structures.

File Name: Supplementary Data 2

Description: The Python codes for developing the MLVR model and LOO cross-validation.

File Name: Supplementary Data 3

Description: Data for cross-validation.
